# Multidirectional strain-insensitive stretchable RF electronics

**DOI:** 10.1038/s41467-026-74900-5

**Published:** 2026-06-27

**Authors:** Furong Yang, Senhao Zhang, Jinyao Zhang, Yao Tong, Jun Zhong, Junjie Zheng, Jiawei Li, Yichao Hu, Yangbo Yuan, Jia Zhu, Kai Xu, Cheng Zhang, Huanyu Cheng, Chaoyun Song

**Affiliations:** 1 College of Electronics and Information Engineering, Shenzhen University, Shenzhen, 518060 China; 2 Department of Engineering, King’s College London, London, WC2R 2LS UK; 3 Department of Engineering Science and Mechanics, The Pennsylvania State University, University Park, 16802 USA; 4 Suzhou Institute of Biomedical Engineering and Technology, Chinese Academy of Sciences, Suzhou, 215011 China; 5 School of Materials and Energy, University of Electronic Science and Technology of China, Chengdu, 610054 China; 6 Shanghai Institute of Optics and Fine Mechanics, Chinese Academy of Sciences, Shanghai, 201800 China

**Keywords:** Electrical and electronic engineering, Electronic devices

## Abstract

Stretchable radio-frequency (RF) electronics underpin emerging wearable systems for body-centric communication, continuous health monitoring, and wireless power transfer. However, on-body stretchable antennas undergo multidirectional in-plane strain during natural motion, which detunes resonance and destabilizes wireless links. Existing strain-insensitive designs are typically effective only along prescribed loading directions and often compromise radiation performance. Here, we establish a systematic directional mechano-electromagnetic analysis framework for resonant planar antennas and introduce a dual-port multidirectional strain-insensitive antenna (DP-MSiA), whereby strain-insensitive resonance (shift ≤ 40 MHz at 2.45 GHz) is achieved under up to 45% strain across diverse in-plane directions. Based on the stable resonance and high realized gain of the DP-MSiA, we demonstrate strain-insensitive wireless energy harvesting with rectifiers under in-plane strain of varying direction and magnitude, as well as a strain-robust on-body communication system that sustains stable multimodal health-data transmission during natural motion. Our work opens new opportunities for creating deformation-insensitive electronics and enables integrated functionalities in wearable and embodied systems.

## Abstract (web summary)

Researchers develop a multidirectional strain-insensitive stretchable antenna that maintains stable wireless communication and energy harvesting under large body deformation, enabling reliable wearable health monitoring during natural motion.

## Introduction

Stretchable radio-frequency (RF) electronics promise a seamless human-machine interface for continuous health tracking^1,2^, body-centric communication^3–5^, and on-demand wireless powering^5,6^, overcoming the spatial limits of wired systems and enabling next-generation wearable platforms for long-term, personalized monitoring^7^. However, stretchable RF components inevitably undergo mechanical deformation in daily operation, which perturbs their intrinsically engineered electromagnetic response^8–10^. In particular, the resonant frequency of conventional stretchable antennas is highly sensitive to tensile strain and often exhibits an approximately linear frequency shift, a characteristic that has been widely exploited for strain sensing^11,12^. Nevertheless, such strain-induced frequency shifts are undesirable for communication and wireless power transfer (WPT), because detuning causes impedance mismatch, radiation-pattern distortion, and efficiency loss, ultimately degrading link stability^8–10^.

Strain-induced resonance shifts of resonant antennas are primarily governed by deformation-driven changes in the effective electrical length of the dominant surface-current path^13^. Recent approaches have pursued strain-insensitive resonance through structural design^14,15^ and strain-responsive dielectrics^16^. For example, a three-dimensional arched serpentine-mesh patch has been reported to exhibit only ~2% frequency shift under up to 20% tensile strain by hierarchical unfolding^14^, but integrating such 3D architectures with planar wearable electronics remains challenging^15^. In parallel, material-based strategies using dielectric-elastic elastomer (DEE) substrates have been explored, where the permittivity changes with strain and can partially counteract strain-induced changes in electrical length^17^. However, in practice, the achievable permittivity modulation in DEE substrates remains too small to offset the electrical-length change of a conventional patch under strain^16,17^. Although recent advances in cluster-based synthesis have improved the tunability, the attainable tuning range remains insufficient for complete compensation^17^. Consequently, prior work adopts serpentine patterning to reduce the deformation-driven resonance-frequency shift, making the available permittivity tuning sufficient to achieve strain insensitivity in planar antennas^17^. However, this serpentine patterning entails miniaturization^18–20^ to preserve the target resonance, which can markedly reduce the effective radiation resistance^21–23^ and radiating aperture^24^, thereby degrading radiation efficiency and realized gain^21–24^. These limitations highlight the need for a new strategy that enables strain-insensitive planar antennas while preserving high radiation efficiency.

In resonant patch antennas operating in the fundamental mode, the excited surface-current distribution is primarily oriented along a principal in-plane direction^13^. Stretching parallel to this current orientation increases the effective electrical length and shifts the resonance to a lower frequency^9,13,25,26^, whereas stretching orthogonal to it reduces the effective electrical length via transverse Poisson contraction and shifts the resonance upward^9,25,26^ (Fig. S1). Such direction-dependent shifts are common in wearable operation, where daily motion imposes strains with varying direction and magnitude even at the same on-body location^27–30^. However, systematic analyses of the directional mechano-electromagnetic response of conventional planar antennas under strain remain elusive, along with corresponding multidirectional strain-insensitive design strategies^31^. This motivates a multidirectional strain-insensitive design strategy that preserves both resonance frequency and radiation performance under arbitrary strain orientations.

**Fig. 1:**
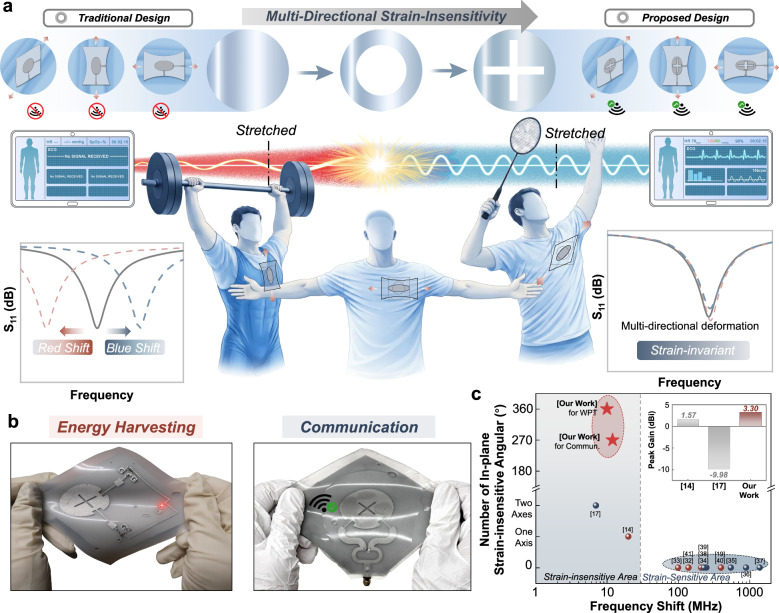
Overview of the dual-port multidirectional strain-insensitive antenna (DP-MSiA). **a** On-body motion induces in-plane stretching with varying directions. Conventional stretchable antennas detune, causing resonance red/blue shifts and mismatch, whereas the proposed symmetry-guided slot loading maintains near-constant resonance under multidirectional stretching. **b** Representative implementations, including integration with rectifiers for wireless energy harvesting and use as a wearable communication antenna under large deformation. **c** Benchmarking against prior stretchable antennas, including strain-insensitive designs, shows a smaller resonance shift, wider-angle strain insensitivity, and higher realized gain under stretching.

Herein, a dual-port multidirectional strain-insensitive stretchable antenna (DP-MSiA) is introduced to maintain stable resonance under arbitrarily oriented in-plane stretching. On-body antennas experience in-plane strain during diverse daily activities, so the strain direction and magnitude can vary continuously in use (Fig. 1a). In conventional stretchable antennas, this direction-varying stretching detunes the antenna, causing the resonance to shift to lower (red shift) or higher (blue shift) frequencies depending on the strain direction. The resulting mismatch weakens the wireless link and can lead to signal dropouts and unreliable reception of physiological data. In contrast, the proposed symmetry-guided slot-loaded design maintains a near-constant resonance under multidirectional strain, enabling more reliable wireless links for continuous physiological monitoring. Representative system-level implementations enabled by this capability include integration with rectifiers for wireless energy harvesting and use in wearable communication under large deformation (Fig. 1b). Moreover, benchmarking the DP-MSiA against prior stretchable antennas shows a smaller resonance shift and strain insensitivity over a broader range of in-plane strain orientations than recently reported designs that are strain-insensitive only along one or two prescribed loading directions (Fig. 1c)^14,17,19,32–41^. The DP-MSiA also maintains a relatively high and stable realized gain under strain, which remains challenging for prior stretchable antennas, including strain-insensitive designs (Table S1). The reported design framework is material-agnostic and can be implemented with diverse stretchable conductors (e.g., liquid metals, Ag nanowires, and graphene) and elastomeric substrates (e.g., PDMS, Ecoflex, SEBS, and hydrogels). Beyond materials, the same symmetry- and mode-guided strategy also applies to a broad class of cavity-type resonant antennas. This multidirectional strain-insensitive operation improves practical reliability and enables straightforward integration into a wide range of wearable components and devices.

## Results and Discussion

### Symmetry-based characteristic-mode framework for multidirectional strain insensitivity

As a feed-independent eigenmode framework that isolates the intrinsic radiation modes set by the radiator geometry and boundary conditions, characteristic mode analysis (CMA)^42^ is exploited to analyze strain-induced resonance shifts. CMA links mechanical strain directly to changes in the fundamental radiation modes. To focus on geometry-driven effects, the classical cavity model is adopted (Notes S1 and S2), with a rotationally symmetric circular patch used as the reference. Its continuous symmetry provides a clean baseline for interpreting degeneracy and strain-induced mode splitting.

**Fig. 2:**
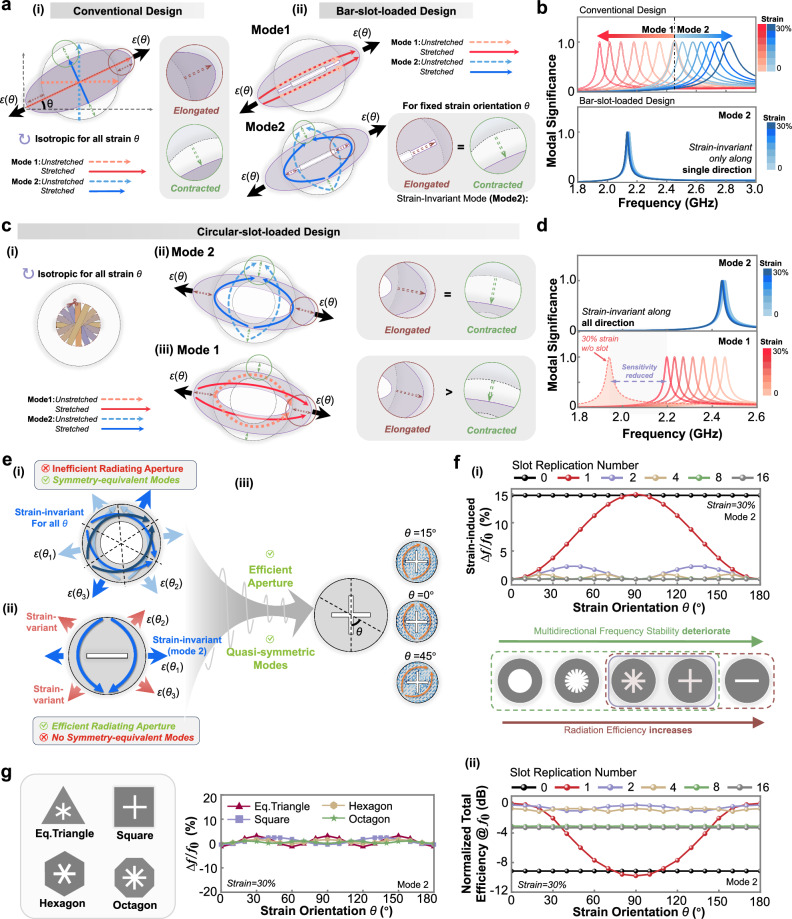
Symmetry-based mode–mechanical coupling and modal compensation for multidirectional strain insensitivity. **a (i)** CMA schematics of the fundamental orthogonal modes of a circular patch (planar cavity) under uniaxial strain applied at an in-plane angle *θ*, and **(ii)** the bar-slot-loaded patch under uniaxial strain applied along the bar-slot length axis. **b** Simulated modal significance versus frequency for Modes 1 and 2 under strain for the circular and bar-slot-loaded patches. **c (i)** Circular-slot topology formed by rotating and replicating the bar-slot motif, and **(ii, iii)** representative Mode 1 and Mode 2 modal current-path. **d** Mode 1 and Mode 2 resonance frequency shift versus uniaxial strain levels (isotropic response for different *θ*). **e** Design trade-off between multidirectional Mode 2 strain invariance and radiation efficiency: **(i)** circular slot, **(ii)** bar slot, and **(iii)** cross slot. **f (i)** Mode 2 multidirectional frequency stability under strain from a sweep of slot replication number, and **(ii)** corresponding total efficiency under the same strain. **g** Extension to polygonal patch geometries showing low strain sensitivity of the strain-orthogonal mode 2.

As shown in Fig. 2a, the arrows schematically trace the dominant modal surface-current path that defines each mode’s effective electrical length and resonance trend. Dashed paths denote the unstrained configuration, whereas solid paths denote the deformed configuration under applied tensile strains, highlighting where the surface-current path lengthens along the tensile axis and shortens due to transverse Poisson contraction. For an ideal circular patch, the fundamental modes form an orthogonal, frequency-degenerate pair due to continuous rotational symmetry^43^ (Fig. 2a–i, dashed arrows). This degenerate pair has no preferred in-plane orientation, and any rotated orthogonal basis is equally valid^13^. However, when uniaxial tensile strain is applied at an in-plane angle *θ*, the deformation breaks rotational symmetry (Fig. 2a–i, semi-transparent ellipse). This loss of rotational freedom locks the mode pair to the principal deformation axes with Mode 1 oriented along the tensile strain (major) axis (solid red arrow) and Mode 2 along the transverse Poisson-contraction (minor) axis (solid blue arrow). As strain increases, Mode 1 shifts to a lower frequency as its effective electrical length increases, whereas Mode 2 follows an opposite frequency shift set by transverse Poisson contraction (Fig. 2b, upper). Because the unstrained circle is isotropic, this baseline response depends on strain magnitude rather than *θ*.

To achieve strain-insensitive changes in the resonance frequency, a slot-enabled mechano-electromagnetic compensation mechanism is introduced. A centered bar slot has a negligible impact on Mode 1, but forces Mode 2 to detour and increases its unstrained effective electrical length (Fig. 2a–ii, dashed blue arrow). Under uniaxial tensile loading applied along the slot-length axis, Mode 1 remains tensile-axis oriented and elongates (Fig. 2a–ii, solid red arrow). Mode 2 follows the slot boundaries (Fig. 2a–ii, blue arrow), with two competing contributions upon stretching relative to the unstrained path. Specifically, the dominant surface-current path lengthens along the tensile direction near the slot but shortens near the patch perimeter due to transverse Poisson contraction. By optimizing the unstrained slot length (Fig. S2 and Note S3), these contributions can be balanced so that the net electrical length of Mode 2 becomes nearly invariant with strain, yielding a strain-insensitive Mode 2 (Fig. 2b, lower). This bar slot, however, breaks rotational symmetry, splits the two unstrained resonances, and defines a preferred axis. As a result, the strain-insensitive condition is directional and holds true only when the strain is applied along the designed axis, namely, the slot length direction.

Multidirectional strain insensitivity requires restoring isotropy while retaining the slot-enabled compensation mechanism. Therefore, the bar-slot pattern is rotated and replicated to form a circular slot topology, followed by rescaling the geometry to place Mode 2 at the target frequency (Fig. 2c–i). Unlike a single bar slot, the circular slot preserves the shape’s rotational symmetry and retains a similar compensation mechanism for Mode 2 (Fig. 2c–ii). A compensation condition tuned for one loading direction therefore extends across *θ*, yielding multidirectional strain insensitivity for Mode 2 (Fig. 2d, upper). Moreover, the circular slot also modifies Mode 1, with the deformation contributions effectively exchanging roles relative to Mode 2. Specifically, its surface-current path lengthens near the patch edge and shortens near the slot (Fig. 2c–iii), leading to partial cancellation (Note S4) that reduces the strain sensitivity of Mode 1 (Fig. 2d, lower).

A practical trade-off emerges between multidirectional Mode 2 strain insensitivity and radiation efficiency. A circular slot (Fig. 2e-i) yields minimal Mode 2 frequency shifts for arbitrary in-plane strain angle *θ* for two reasons: 1) continuous rotational symmetry enables a degenerate continuum of rotated Mode 2 realizations, and 2) the slot-enabled compensation is isotropic, acting similarly for any in-plane loading direction and thereby minimizing the strain-induced frequency shift across *θ*. However, achieving this condition requires a large circular slot size (Fig. S3a, b), which confines the Mode 2 current to a longer, thinner annular path, lowering radiation resistance and increasing conductor loss via current crowding, thus reducing radiation efficiency. By contrast, a single bar slot (Fig. 2e–ii) achieves compensation with a shorter slot (Fig. S3c) and a more efficient radiating aperture, but it breaks the patch’s rotational symmetry. Compensation then becomes orientation-specific rather than robust to *θ*. A cross-slot geometry (Fig. 2e–iii) provides an optimal solution to address the trade-off. Although the cross-slot geometry has only discrete fourfold rotational symmetry, it supports quasi-rotationally symmetric Mode 2 current patterns that have nearly identical resonance frequencies (Fig. S4). Meanwhile, it also exhibits minimal strain-induced frequency shift with low sensitivity to strain angle (Fig. S5), while avoiding the strong annular current confinement imposed by a circular slot.

Further sweeping the slot replication number quantifies multidirectional Mode 2 strain insensitivity (Fig. 2f–i). Without a slot, Mode 2 exhibits a large but nearly *θ*-independent frequency shift, consistent with the isotropy of the circular patch. Introducing a single bar slot breaks this isotropy and introduces strong orientation dependence, with Mode 2 becoming strain-insensitive only near specific strain directions and showing pronounced shifts elsewhere. A qualitative transition occurs for the cross-slot configuration (replication number of 2), where Mode 2 becomes nearly frequency-stable over the full 180° range of strain orientations, indicating that discrete rotational symmetry can recover a quasi-isotropic response. Further increasing the number of rotated slot copies progressively suppresses the remaining *θ*-dependence, approaching the isotropic limit.

Full-wave simulations exciting Mode 2 reveal the corresponding efficiency trend under large strain (Fig. 2f-ii). For the slot-free patch, the Mode 2 frequency shift causes impedance mismatch and yields a nearly *θ*-independent reduction in total efficiency at 30% strain. A single bar slot introduces strong *θ*-dependence in the Mode 2 shift, leading to a pronounced efficiency minimum at *θ* = 90° where frequency detuning is largest. Increasing slot replication suppresses the remaining *θ*-dependence, flattening the efficiency versus *θ*. However, near-omnidirectional Mode 2 stability in higher-replication patterns (N ≥ 8) requires a large slot size (Fig. S6), which increases current crowding and detouring around the slot edges and reduces radiation efficiency, thereby lowering total efficiency. The cross-slot topology achieves high Mode 2 stability across strain angles while retaining higher total efficiency than the more densely replicated patterns in this study, which is therefore selected as the final optimized slot design.

This symmetry-based mechanism is not restricted to circular radiators. Other highly symmetric planar cavities, including the equilateral triangle, square, regular hexagon, and regular octagon, exhibit analogous degenerate orthogonal mode pairs and similar strain-induced frequency responses under uniaxial stretching (Figs. S7 and S8). When slot patterns are designed to preserve the original rotational symmetry while enabling complementary slot lengthening and shortening for the strain-orthogonal mode (Mode 2) (Fig. S9), similarly low strain sensitivity is achieved, as summarized in Fig. 2g.

### Design and mechanical-electromagnetic characterization of DP-MSiA

**Fig. 3:**
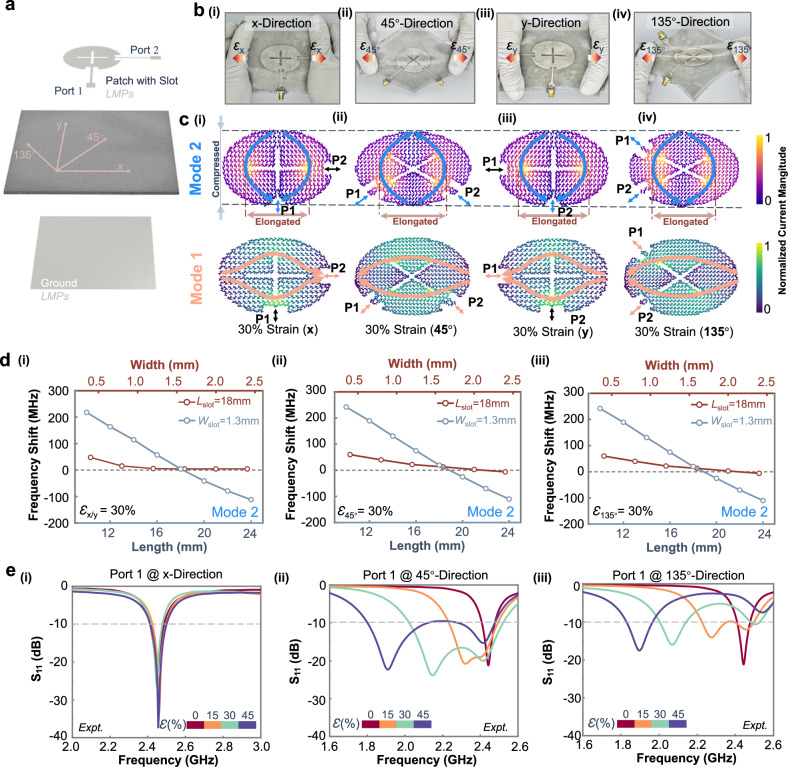
Design and characterization of DP-MSiA. **a** Exploded view of the DP-MSiA being stretched along different directions. **b** Photographs and **c** simulated surface current distributions of the DP-MSiA under representative uniaxial strain applied along (i) x, (ii) 45°, (iii) y, and (iv) 135° directions, illustrating the strain-insensitive (Mode 2) and strain-sensitive (Mode 1) resonances excited by the two ports. The upper row (mode 2) uses the plasma colormap and the lower row (mode 1) uses the viridis colormap to indicate normalized current magnitude, and the overlaid curves trace the dominant effective current path. **d** Simulated strain-induced resonant frequency shift of mode 2 as a function of the slot length (*L*_slot_, blue) and width (*W*_slot_, red) under tensile strain of 30% applied along (i) x/y, (ii) 45°, and (iii) 135° directions. **e** Measured S_11_ of port 1 under tensile strain from 0% to 45% applied along (i) x, (ii) 45°, and (iii) 135° directions.

To achieve multidirectional strain-insensitivity, the strain-insensitive mode (Mode 2) needs to be effectively excited as the strain direction varies. A single feed can efficiently excite only a subset of the desired strain-insensitive modal current distributions as the desired resonance becomes weak when the modal current distribution has a large angular mismatch with the feed line (Fig. S10). This limitation is addressed by implementing a dual-port multidirectional strain-insensitive antenna (DP-MSiA) with two orthogonal ports (Fig. 3), forming the minimum set of in-plane coupling channels needed to excite Mode 2 as the applied tensile strain direction varies. By combining Ecoflex cured in a mold and liquid metal particles (LMPs) via air spraying (Fig. S11), the DP-MSiA features a thin Ecoflex substrate layer (~2 mm) sandwiched between the LMPs-based patch with a cross-shaped slot and a square ground layer (Fig. 3a). It should be noted that, after ultrasonication, the liquid metal particles exhibit a size distribution predominantly in the range of 1 ~ 4 μm (Fig. S12a). However, the transfer process induces partial rupture of the oxide shells, facilitating particle coalescence at the Ecoflex surface and the formation of electrically percolated networks within the LMPs-Ecoflex composites (Fig. S12b). Moreover, the intrinsically stretchable LMPs patterned on Ecoflex also maintain stable high conductivity upon stretching of 300% (Fig. S13) to serve as an effective near-perfect conductor for efficient electromagnetic radiation. Meanwhile, the LMPs trace on Ecoflex exhibit a low modulus (~125 kPa) and remarkable stretchability (>300%) without mechanical failure (Fig. S14). As a result, the fabricated DP-MSiA can withstand tensile strain applied along different directions (Fig. 3b) for conformal integration on irregular 3D surfaces.

The full-wave simulated surface current in Fig. 3c shows that excitation of Modes 1 and 2 is governed by alignment between each port’s feedline axis and the mode’s local surface-current orientation at the feed region. For 30% strain along the *x*-direction (Fig. 3c–i), Port 1 primarily excites Mode 2, whereas Port 2 primarily excites Mode 1. Cross-excitation is weak because the orthogonal mode presents only a small current component along the driven axis at that port. For strain along the y-direction (Fig. 3c–iii), the modal current orientations rotate and the port selectivity swaps, with Port 1 exciting Mode 1 and Port 2 exciting Mode 2. For intermediate strain angles such as 45° and 135° (Fig. 3c–ii, iv), neither mode is aligned or orthogonal to a single feedline axis, so both ports contribute to the excitation. These cases illustrate that the two orthogonal ports provide a complete in-plane excitation basis, so Mode 1 and Mode 2 remain accessible for any strain direction, either by one port alone or a combination of both.

While CMA reveals the intrinsic mode-strain coupling, the practical antenna response is quantified with port-driven full-wave simulations, where the slot length (*L*_slot0_) and width (*W*_slot0_) are swept and the resonance shift Δ*f* under 30% stretching is used as the optimization metric. For the applied tensile strain of 30% along the x- or y-directions, increasing the initial slot length from 10 mm to 18 mm and then to 24 mm progressively reduces the resonance frequency shift Δ*f* of Mode 2 from +218 MHz to approximately 0 and then to −112 MHz (Fig. 3d–i), validating a tunable strain-induced frequency shift from the slot. At the optimized slot length of 18 mm, increasing the slot width from 0.4 mm to 2.4 mm reduces Δ*f* from +48 MHz to nearly zero (+3 MHz) at 1.3 mm and then stabilizes afterwards. These results indicate that overly narrow slots reduce the effective slot length through capacitive bridging between slot edges, leading to insufficient compensation. When the tensile strain of 30% is applied along 45 and 135° (Fig. 3d-ii, iii), the optimal initial slot length increases slightly from 18 to ~ 19 mm. However, the frequency shift Δ*f* is only 20 MHz at 18 mm to cause a negligible difference for practical applications. As a result, the initial slot length of 18 mm and width of 1.3 mm are chosen in the following studies unless specified otherwise (Fig. S15).

The S-parameters (S_11/22_, S_12/21_) of the fabricated DP-MSiA are further measured under tensile loading applied along the four representative in-plane directions (i.e., x, 45°, y, and 135°) to reveal the direction-dependent mechanical-electromagnetic coupling and resonant behavior. The S_12/21_ remains below −10 dB under stretching along x- and y-directions (Fig. S16a). In contrast, the S_12/21_ increases beyond −10 dB at 2.45 GHz (Fig. S16b) under stretching along 45°- and 135°-directions. This difference in S_12/21_ is consistent with the surface-current distributions in Fig. 3c. For stretching along x and y directions, the excited modal current at the opposite (orthogonal) port is nearly orthogonal to that port’s feed axis, giving a near-zero projection and thus negligible inter-port coupling. Under 45° and 135° strain, the modal current orientation rotates relative to the deformed feed line axes. When one port is excited, the induced current at the other port is no longer perpendicular to its feed axis. This introduces a finite current component along that axis and therefore increases inter-port coupling. In addition, the S_11_ of Port 1 exhibits a remarkable strain-insensitive property, with resonance frequency essentially unchanged (a maximum of 12 MHz shift) at 2.45 GHz under tensile strain of up to 45% along the x-direction (Fig. 3e-i), in good agreement with simulation results (Fig. S17). In contrast, Port 2 shows an almost linear redshift in its resonance with increasing x-direction strain (Fig. S18). Due to symmetry, the S-parameter characteristics of Port 1 and Port 2 switch roles upon stretching in the y-direction, with Port 2 exhibiting a maximum frequency shift of only 20 MHz (Fig. S19), consistent with the port-mode selectivity discussed above. Under diagonal stretching along the 45° or 135° directions, each port can be coupled to both modes, and the S_11_ and S_22_ responses exhibit two resonances as strain increases. For Port 1 excitation, one resonance (Mode 1) redshifts with increasing strain, while the other (Mode 2) remains nearly strain-invariant (maximum of 40 MHz shift) near 2.45 GHz (Fig. 3e-ii, iii). The same behavior is observed for Port 2 excitation (Fig. S20) as diagonal stretching is along the symmetric axis of the antenna. Overall, the DP-MSiA exhibits robust strain insensitivity for all four stretching directions, whereas conventional dual-port counterparts without slots suffer from substantial frequency detuning upon stretching (Figs. S21 and S22). A summary plot showing the extracted frequency shift (Δ*f*) versus strain level and stretching direction for both the proposed DP-MSiA and the conventional reference antenna is provided in Fig. S23 and Table S2.

### Multidirectional strain-insensitive RF wireless energy harvester based on DP-MSiA

**Fig. 4:**
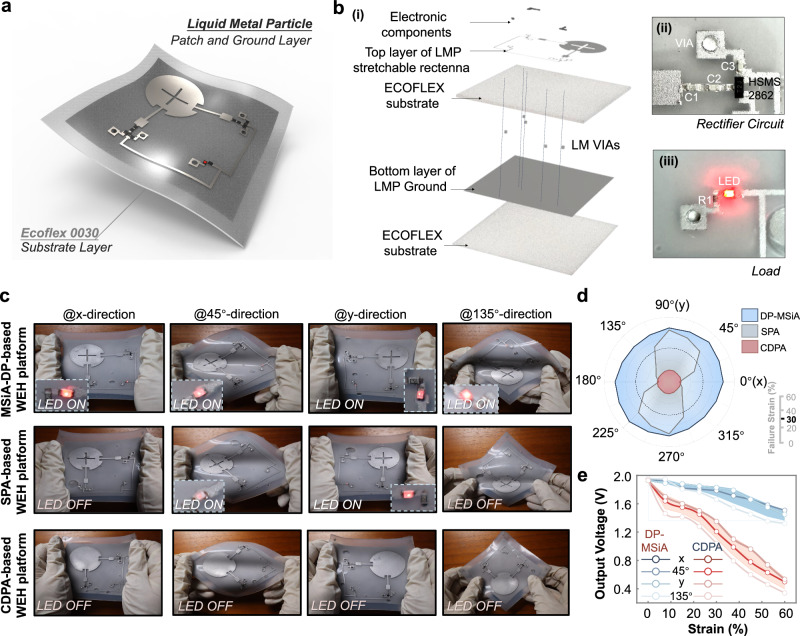
Multidirectional strain-insensitive RF wireless energy harvester based on DP-MSiA. **a** Schematic illustration showing an LED powered by the DP-MSiA-based rectenna. **b** (i) Exploded view of the overall system and top-view photographs of (ii) the rectifier circuit and (iii) the loading part. **c** Photographs of the overall systems powered by DP-MSiA-based, SPA-based, and CDPA-based rectennas under representative uniaxial strains applied along 0, 45, 90, and 135°, along with **d** polar plot of the failure strain for the DP-MSiA-, SPA-, and CDPA-based WEH systems. **e** Comparison of the output voltage between the (slot) DP-MSiA-based and (solid) CDPA-based rectennas as a function of tensile strain increasing from 0% to 60% applied along four representative directions. The pink/blue shaded regions represent the error bands of the DP-MSiA- and CDPA-based WEH systems.

To further demonstrate the practical functionality of wireless powering, the DP-MSiA is integrated with two identical lumped-element rectifying circuits at two ports to convert harvested RF energy into a regulated voltage output separately. The resulting two direct current (DC) outputs are further combined at a common output node to power a light-emitting diode (LED), yielding a standalone far-field wireless energy-harvesting (WEH) platform (Fig. 4a and S24). Due to the excellent solderability of the LMPs-based traces, the electronic components (e.g., resistor, capacitor, LED, and Schottky diode) are soldered using stretchable conductive silver paste (Fig. 4b). The via holes (2 mm in thickness), with transferred LMPs coating on the walls to enhance interfacial adhesion with the subsequently injected pristine liquid metal (Fig. S25), form a stable electrical connection between the LMPs-based top layer of stretchable rectenna circuits and the bottom layer of ground.

The performance characterization of the DP-MSiA-based WEH platform under tensile strains along four representative directions (0°, 45°, 90°, and 135°) reveals comprehensive directional coverage (Fig. 4c). The LED powered by the DP-MSiA-based WEH platform maintains continuous illumination under ~30% stretching along all directions, attributed to the multidirectional strain-insensitivity. To investigate the contribution of each port in the dual-port architecture, the rectifying circuit is only connected to one port of the DP-MSiA, while the other port is left open, forming an equivalent single-port antenna (SPA)-based WEH platform. In this single-port configuration, LED illumination is only sustained under ~30% stretching along 45° and 90° directions. These results show that the multidirectional strain insensitivity is provided by the dual orthogonal feeds. It is also noteworthy that inter-port coupling, evidenced by the elevated S_21_ response under diagonal stretching (Fig. S16), does not compromise the wireless energy-harvesting efficiency when the rectifier outputs are properly isolated. This observation results from the fact that the RF power coupled into either port is locally rectified before jointly contributing to the common DC load. In comparison, the conventional dual-port antenna (CDPA)-based WEH platform (Fig. S26) fails to provide a stable power supply to the LED even under a small tensile strain of ~ 5% applied in any direction. The degradation of the slot-free configuration in power transfer results from strain-induced frequency shift, which simultaneously reduces the radiation efficiency (Fig. S27) and leads to significant port-impedance variation at 2.45 GHz (Fig. S28) for increased mismatch between the antenna and the rectifier.

The failure strain (defined as the maximum strain to maintain LED illumination) is also measured for all three WEH platforms, with eight representative in-plane stretching directions (Fig. 4d). An additional four directions are included to increase the angular resolution to 22.5° for a more detailed performance comparison. The CDPA-based WEH platform exhibits a limited failure strain of <10% at all tested directions, which is much smaller than the 30% needed for typical wearable deformations. Moreover, the SPA-based platform meets the strain requirement only at 45° and 90° directions. By contrast, the DP-MSiA-based platform features a failure strain of at least 45% along all tested directions, which supports a (quasi-)omnidirectional (360°) strain-insensitivity within the tensile strain from 0 to 40%.

For quantitative characterization, the rectenna output across a 160-ohm load is evaluated in a free-space, boresight-aligned, polarization-matched configuration at a transmitter-receiver separation of 1 m using a 3 dBm signal generator, a 30 dB power amplifier, and a commercial transmitting patch antenna with a gain of 19 dBi, corresponding to an estimated received RF power at the rectenna of *P*_r_ = 15.08 dBm. The output voltage is measured as a function of tensile strain from 0% to 60% along the four representative directions (Fig. 4e). Different from the pronounced voltage drop from 2.0 V to ~0.5 V with increasing strain from 0% to 60% in the CDPA-based WEH platform, the DP-MSiA-based WEH platform maintains a stable output voltage up to ~40% stretching (followed by a slight drop to ~1.6 V upon 60% stretching). The measured voltages are further converted to DC output power and normalized RF-to-DC efficiency, with the efficiency values referenced to the estimated received RF power in the unstrained state to provide a common basis for comparison across different strain states (Fig. S29). Specifically, the DP-MSiA-based platform maintains a high normalized RF-to-DC efficiency above 40% at 60% strain, while that of the CDPA-based platform decreases to only around 5% at 60% strain, depending on the stretching direction. The results confirm the importance of the cross-shaped slot structure and dual-port configuration in achieving multidirectional, strain-insensitive RF energy harvesting. In addition, cyclic stretching tests show that the WEH platform retains stable functionality after repeated deformations to illuminate the LED (Fig. S30a). It exhibits only a minor decrease in output voltage across a 160-ohm load after 600 stretching cycles along the x-axis followed by 600 additional cycles along the y-axis (Fig. S30b).

### Design and characterization of SP-MSiA for stable communication

**Fig. 5:**
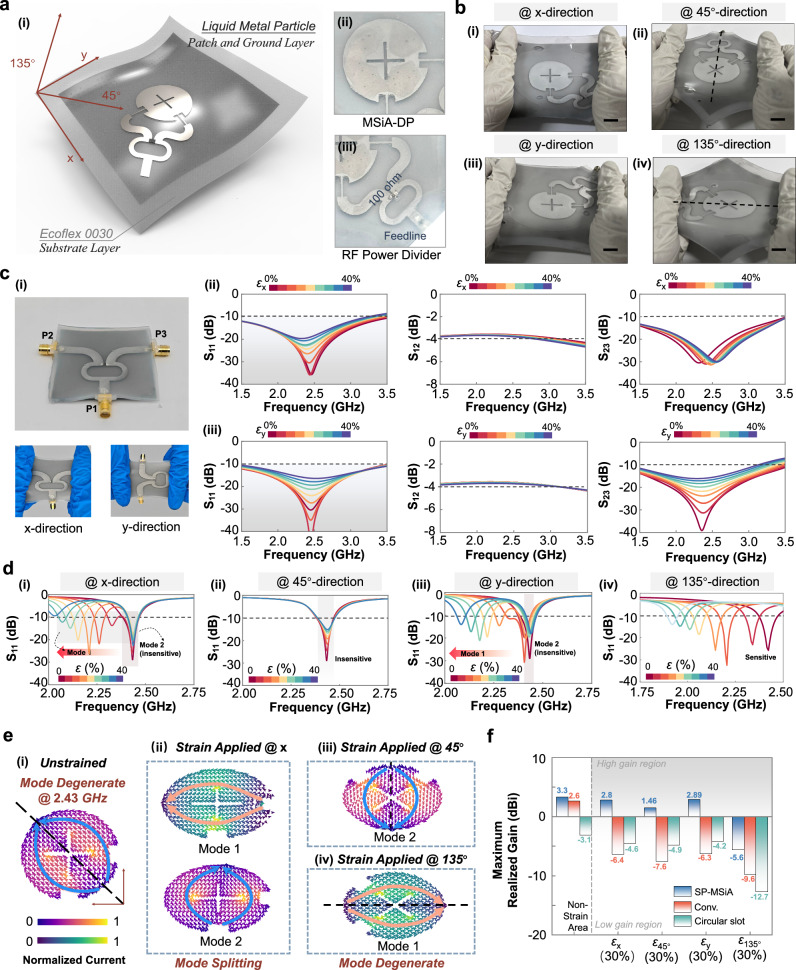
Multidirectional strain-insensitive communication system based on SP-MSiA. **a** (i) Schematic showing Ecoflex-LMPs-based SP-MSiA that integrates (ii) a DP-MSiA and (iii) a 2.45 GHz RF power divider (shown in optical photographs), with different stretching directions labeled. **b** Photographs of the SP-MSiA with representative uniaxial strain applied along (i) x, (ii) 45°, (iii) y, and (iv) 135° directions. **c** (i) Photographs of the fabricated standalone stretchable Wilkinson power divider (PD) before (top) and after (bottom) stretching. (ii) Measured S-parameters (S_11_, S_12_, and S_23_) of the standalone stretchable Wilkinson PD under tensile strain applied along the (ii) x-direction and (iii) y-direction. **d** Measured S_11_ of the SP-MSiA under tensile strain of 0 − 40% applied along (i) x, (ii) 45°, (iii) y, and (iv) 135° directions. **e** Simulated surface-current distributions showing (i) the unstrained, in-phase excitation of a superposition of two orthogonal modes, (ii) uniaxial stretching breaking the degeneracy and splitting the modes into Mode 1 (strain-aligned) and Mode 2 (strain-orthogonal), (iii) the excited Mode 2 pattern (strain-orthogonal) under stretching along the 45° direction, and (iv) the excited Mode 1 pattern (strain-aligned) under stretching along the 135° direction, with Viridis/Plasma colormap marking the strain-sensitive Mode 1 and insensitive Mode 2, respectively. **f** Measured maximum realized gain of the SP-MSiA, the conventional stretchable antenna and circular-slot stretchable antenna under tensile strain of 30% applied along (i) x, (ii) 45°, (iii) y, and (iv) 135° directions.

Although a parallel rectifying configuration can be implemented with the dual-port structure, the dual-port output cannot directly interface with commercial communication chips that require a single-ended input. Therefore, two orthogonal feeds of the DP-MSiA are combined using a Wilkinson power divider (PD) to obtain a single-port, multidirectional, and strain-insensitive antenna (SP-MSiA) (Fig. 5a). Specifically, the quarter-wave branches of PD combine the two port signals in phase to present a matched output. Meanwhile, the isolation resistor (100 ohms in a 50-ohm system) is connected between the two branches of the Wilkinson PD. The generated reflections from any significant impedance mismatch at one port (due to strain-induced geometric symmetry breaking) are mainly terminated in this resistor rather than coupling into the other port. As a result, port-to-port isolation is improved to stabilize the radiation characteristics. The antenna is designed in a planar microstrip using the same LMPs-based conductive layer to ensure mechanical compliance and consistent stretchability (Fig. 5b), exhibiting strain-insensitive characteristics. To experimentally validate the strain tolerance of the feeding network, a standalone stretchable Wilkinson PD is fabricated (Fig. 5c-i) and measured. The S-parameters (S_11_, S_12_, and S_23_) of the fabricated standalone PD remain largely stable across the 2.4 – 2.48 GHz ISM band under uniaxial stretching along both the x- and y-directions, with only minor variations observed as the strain increases (Fig. 5c-ii and iii). These results confirm the stable RF performance of the stretchable PD under stretching, providing a reliable feeding network for the subsequent single-port antenna design.

Feeding the dual ports of DP-MSiA through a symmetric PD feedline network structure ensures in-phase excitation at both ports of the patch, since the electrical path length from the PD input to each port is identical. However, such an in-phase feeding of the symmetric circular patch excites a superposition of the degenerate mode pair, whose relative modal amplitudes and the effective input impedance become highly sensitive to small geometric or manufacturing deviations. Minor fabrication tolerances can therefore shift the impedance and resonance and degrade measured performance relative to idealized simulations. Therefore, a slight geometric asymmetry in the form of an elliptical patch is introduced (Fig. S31) to split the two modes into distinct resonant frequencies within the band of interest (ISM band: 2.40–2.48 GHz), so the SP-MSiA behaves as a quasi-single-mode radiator around each resonance, enabling more robust impedance matching in the presence of fabrication errors/variations. As a result, two closely spaced modes are generated, with the surface current mainly oriented along the major axis at 2.40 GHz (mode 1) and the minor axis at 2.45 GHz (mode 2) (Fig. S32). The two modes are sufficiently close in frequency that they merge into a single resonance at 2.43 GHz (Fig. 5d) in the unstrained condition. This merging is visualized in the simulated current distribution (Fig. 5e-i), which exhibits a composite pattern from a linear combination of the two orthogonal modes excited at each port. As indicated by the measured S_11_ in Fig. 5d-i, stretching applied along the x-direction (major axis) splits the single resonance into two. Unlike the dual-port cases in Fig. 3c, the PD-fed configuration combines the excitations from both ports at a single input, so the measured S_11_ reflects the superposed responses of Modes 1 and 2. Consistent with the mode trends in Fig. 3, the frequency of mode 1 decreases (or red-shifts) to ~ 2.05 GHz with increasing tensile strain along the x-direction, whereas mode 2 remains nearly unchanged at ~2.45 GHz. Similarly, stretching applied along the minor axis (y-direction) also induces the mode splitting in frequency. Mode 1 is still strain-sensitive and red-shifts from 2.43 to ~2.10 GHz upon stretching, whereas mode 2 remains strain-insensitive and negligibly shifts down to ~2.40 GHz at low strain due to mode coupling, followed by recovery to 2.43 GHz with further increasing strain as the two resonances decouple (Fig. 5d-iii). Nevertheless, such a configuration ensures that mode 2 is effectively excited and remains within the desired ISM band under stretching applied along the x- or y-direction to maintain stable wireless communication.

Under stretching along the x- or y-direction, the strain-coupled modes (Modes 1 and 2) undergo different changes in effective electrical length and therefore split into two distinct resonances. In contrast, the symmetry of the SP-MSiA is preserved under diagonal (45°/135°) stretching, so the PD-feedline excitation continues to drive a superposition of the degenerate mode pair and the response remains a single merged resonance, as confirmed by the measured S-parameters (Fig. 5d-ii, iv). The simulated currents show that under 45° stretching the response is dominated by the strain-orthogonal mode (Mode 2) (Fig. 5e-iii), and the effective electrical length remains essentially unchanged due to slot-patch compensation, yielding a strain-insensitive resonance near 2.43 GHz. By contrast, under 135° stretching the response is dominated by the strain-aligned mode (Mode 1) (Fig. 5e-iv), increasing the effective electrical length and producing the observed redshift. For benchmarking, a conventional unslotted two-port patch antenna operating at 2.45 GHz is fabricated and integrated with the same PD as a reference (Fig. S33). The reference antenna exhibits pronounced resonance shifts for stretching along any direction (Fig. S34) due to strain-induced electrical length changes (Fig. S35). Although the SP-MSiA remains strain-sensitive under 40% stretching along the 135° direction, its resonance shift of ~0.50 GHz is significantly smaller than that of ~0.65 GHz from the unslotted reference, due to the geometric self-compensation discussed in the previous sections. Both the SP-MSiA and the unslotted reference antenna maintain broadly similar radiation patterns under strain. However, the SP-MSiA exhibits much higher maximum realized gains of 2.8, 1.46, and 2.89 dBi under 30% stretching along the x, 45°, and y directions, which are significantly higher than those (−6.4, −7.6, and −6.3 dBi) provided by the conventional counterpart (Fig. 5f and S36). Moreover, the realized gain of −5.6 dBi from the SP-MSiA under stretching along the strain-sensitive 135° direction is also higher than that of −9.6 dBi from the conventional counterpart due to its smaller resonance-frequency shift. As a complementary metric to realized gain, the absolute radiation efficiency of the SP-MSiA in the undeformed state is −4.55 dB, which is close to that of the conventional no-slot patch antenna (−4.3 dB), and remains stable under representative strain levels (Fig. S37). A similar trend is also observed for the circular-hole strain-invariant design (Fig. S38). Specifically, the circular-hole antenna maintains good strain-insensitive resonance behavior under stretching along the x-, 45°-, and y-directions, but still exhibits a pronounced frequency shift under stretching along the 135° direction. However, despite this partial directional strain tolerance, its maximum realized gain remains much lower than that of the cross-slot SP-MSiA in all cases (Fig. 5f), owing to its lower radiation efficiency. Therefore, the cross-slot design offers a more favorable overall trade-off between directional-strain robustness and radiation performance. Nonetheless, the substantially enhanced communication stability of the SP-MSiA under multidirectional stretching paves the way for the practical implementation of stretchable antennas in wearable electronics.

To further evaluate practical wearable conditions, we also examine biaxial stretching, out-of-plane bending, and on-body loading. Under 30% biaxial strain with intersection angles (i.e., the angle between the two applied biaxial stretching directions) of 180°, 150°, 120°, and 90°, the SP-MSiA exhibits resonance shifts of 7.5, 27.5, 46.9, and 221.1 MHz, respectively, which are smaller than those of the reference antenna (270.6, 88.0, 136.4, and 244.2 MHz) (Fig. S39). However, as the intersection angle decreases to 90°, the frequency-stability advantage of the SP-MSiA diminishes because the slot-patch compensation becomes less effective as the patch is stretched near isotropically, increasing the overall patch size and decreasing the resonance frequency. Such a condition is more stringent than most practical wearable deformation states, where biaxial strains are generally unequal in magnitude and not orthogonal. Moreover, measurements under out-of-plane bending conditions show only slight changes in resonance frequency and S_11_ (Fig. S40). These results indicate that the proposed antenna maintains stable impedance behavior under representative wearable deformation scenarios.

### End-to-end wireless communication performance through SP-MSiA

**Fig. 6:**
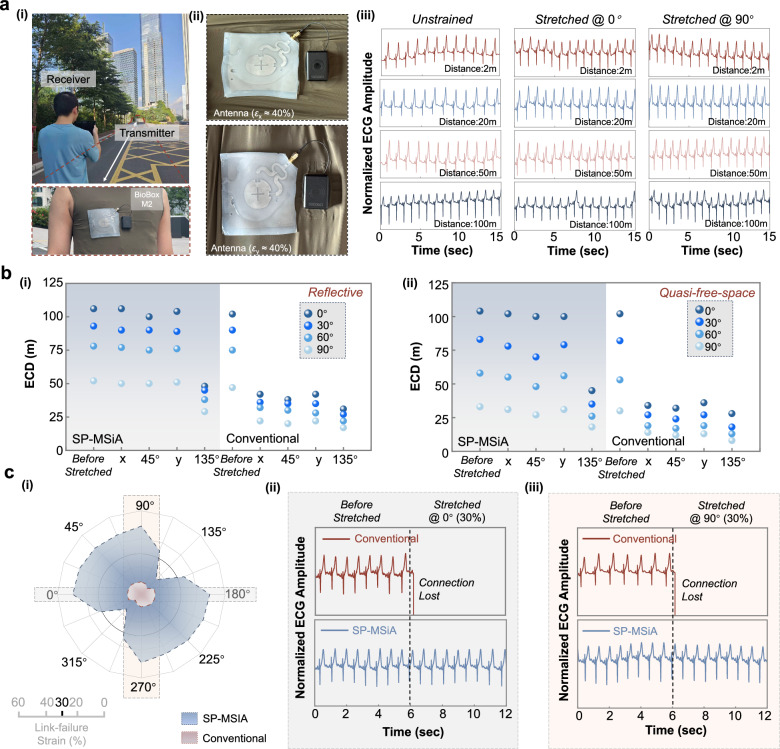
End-to-end wireless communication performance evaluation of SP-MSiA. **a** (i) Experimental setup of the SP-MSiA integrated with a BLE-based wireless ECG monitoring system (BioBox M2) through an IPEX-SMA coaxial feedline. (ii) Photograph of SP-MSiA under 40% stretching along x and y directions. (iii) Received ECG signals at different distances (2–100 m) before (left) and after stretching of 30% along 0° (middle) and 90° (right). **b** Comparison of effective communication distance (ECD) between the SP-MSiA and the conventional stretchable antenna before and after 30% stretching applied along different directions under (i) reflective and (ii) quasi-free-space conditions. **c** (i) Polar plot of link-failure strain for the SP-MSiA and the conventional stretchable antenna. Representative ECG waveforms before and after 30% stretching applied along the (ii) 0° and (iii) 90° directions.

Unlike the free-space WEH validation, the BLE communication study is conducted in a wearable configuration by mounting the SP-MSiA on a smart T-shirt, thereby enabling direct evaluation of communication robustness under practical on-body deformation. To assess the on-body safety of the proposed antenna for wearable operation, SAR analysis is conducted to confirm low SAR values, consistent with the effective shielding provided by the ground plane (Fig. S41). Furthermore, both simulations (Fig. S42a, b) and wearable measurements (Fig. S42c) confirm that the proposed antenna is minimally affected by body loading, because the ground plane effectively shields the radiator from lossy biological tissue, suppresses near-field coupling, thereby preserving the resonance and impedance matching under practical wearable conditions. With a thin stretchable nanogel adhesive applied to the antenna ground plane, the SP-MSiA is bonded to the right pectoral region of a smart T-shirt with embedded electrocardiogram (ECG) electrodes and stretchable interconnects. Connected by an IPEX-to-SMA coaxial cable (Fig. S43a), the SP-MSiA functions as the transmitting antenna in the Bluetooth low energy (BLE)-based commercial wireless ECG monitoring system (BioBox M2). The transmitted signals from the integrated system are received by a commercial tablet computer (RedMi Pad 2) for real-time signal processing and visualization. Such a system setup allows the evaluation of the end-to-end wireless communication performance by positioning the receiver at various distances from the transmitter in an outdoor line-of-sight (LoS) environment, representing a practical wearable use case (Fig. 6a–i). The received ECG signals remain consistently stable as the effective communication range increases from 2 m to 100 m (Fig. 6a–ii, left), even after stretching along the x- and y-directions (Fig. 6a–ii, middle and right). In contrast, the trace antenna (Fig. S43b) supports stable communication only up to ~32 m because of its lower gain compared with the patch antenna. It is worth noting that the high receiver sensitivity of BLE (typically ≈ −92 dBm at 500 kbps, Packet Error Rate < 20.8%) can still show short-range communication (~5 m) by using the IPEX-SMA RF cable itself as a weak radiator (without an antenna).

Furthermore, systematic measurement and comparison of the effective communication distances (ECD) required to sustain a stable wireless link are conducted to highlight the superior and salient features of the reported SP-MSiA relative to the reference antennas. To this end, the testing scenarios consider several representative environments to reflect the diverse real-world conditions, including an indoor reflective line-of-sight (R-LoS) condition and an outdoor quasi-free-space line-of-sight (QFS-LoS) condition. The former preserves the direct path with multipath from surrounding surfaces, whereas the latter has negligible reflections. Meanwhile, due to inevitable human motion during daily activities, the antenna of the on-body transmitter/receiver is rarely perfectly aligned with the link boresight; thus, even small rotations can lead to off-boresight (OB) angles. As a result, a range of off-boresight angles (30°, 60°, and 90°) is investigated (Fig. S44) to provide a comprehensive assessment of the antenna communication distance.

The comparison of the ECD between the SP-MSiA and the conventional reference antenna before and after 30% stretching along different directions is summarized in Fig. 6b, demonstrating the performance difference under the indoor R-LoS (Fig. 6b–i) and outdoor QFS-LoS (Fig. 6b–ii) conditions with different OB angles. Under both indoor LoS and outdoor QFS-LoS conditions, both the SP-MSiA and the conventional reference patch antenna achieve a maximum ECD of >100 m before stretching, because the cross-shaped slot still maintains the high gain of the solid patch. However, the ECD of both antennas before stretching decreases with the increasing OB angle, dropping from a peak of > 100 m at 0° OB angle to the lowest of 50 m at 90° OB angle in reflective condition (Fig. 6b–i), as the link deviates from the antenna main lobe. On the other hand, the body increasingly obstructs the direct path as the OB angle increases, and the link has to rely on weak reflected paths and diffuse multipaths. In addition, when the OB angle between the transmitter and receiver is 90° in an open outdoor setting (QFS condition), the ECD further drops to ~30 m because the quasi-free-space environment lacks sufficient reflectors to redirect energy from the transmitter antenna main lobe to the receiver, resulting in a much weaker received signal (Fig. 6b–ii).

Compared with the reference antenna, the ECD of the SP-MSiA under multidirectional tensile strain conditions exhibits significant stability, owing to its multidirectional strain-insensitive RF characteristics. Specifically, the SP-MSiA preserves a nearly unchanged ECD even when 30% stretching is applied along x, 45°, and y directions under both reflective and QFS conditions, compared with the unstretched state. In comparison, the reference antenna exhibits a significant degradation, with the maximum (0°-OB)/minimum (90°-OB) ECD reduced from 102/47 m to 42/22 (x-stretching), 38/20 (45°-stretching), and 42/22 m (y-stretching) under reflective conditions (Fig. 6b–i). Meanwhile, the minimum (90°-OB) ECD under QFS conditions also drops sharply from 30 m (unstretched) to 14 (x-direction), 12 (45°-direction), and 13 m (y-direction). Under stretching along the 135° direction, the maximum (0°-OB)/minimum (90°-OB) ECD of 48/29 m from the SP-MSiA is also significantly larger than 17/12 m from the reference antenna under reflective conditions. Similarly, under QFS conditions, the SP-MSiA achieves a higher maximum/minimum (0°-OB/90°-OB) ECD (45/18 m) than the reference antenna (28/8 m).

To further analyze the directional dependence of the strain-insensitive performance, the maximum (or link-failure) strain to support stable data communication between transmitter and receiver is measured along varying in-plane axes spaced 22.5° apart at the maximum ECD distance under LoS conditions. The SP-MSiA can sustain a link-failure strain of up to 30% for an overall angular coverage of 270° (except 135° ± 22.5° or 315° ± 22.5°) (Fig. 6c–i, blue). The SP-MSiA can still withstand a tensile strain of up to ~15% to maintain a stable link, even along the most strain-sensitive directions (135°). In contrast, the reference antenna loses the link under a small strain of ~ 5%, regardless of the stretching directions (Fig. 6c-i, orange). The sustained stable wireless transmission from the SP-MSiA provides the integrated system with uninterrupted ECG recordings even under 30% stretching along the x- and y-directions (Fig. 6c–ii, iii), in direct contrast to the abrupt signal dropout experienced by the system with the reference antenna. Furthermore, the cyclic mechanical reliability of the communication platform is also evaluated under repeated stretching to ~50% strain, showing only minor changes in S_11_ (Fig. S45a) and maintaining a maximum communication distance above 100 m even after 600 cycles (Fig. S45b).

### Real-world applications of SP-MSiA-based wearable electronics

**Fig. 7:**
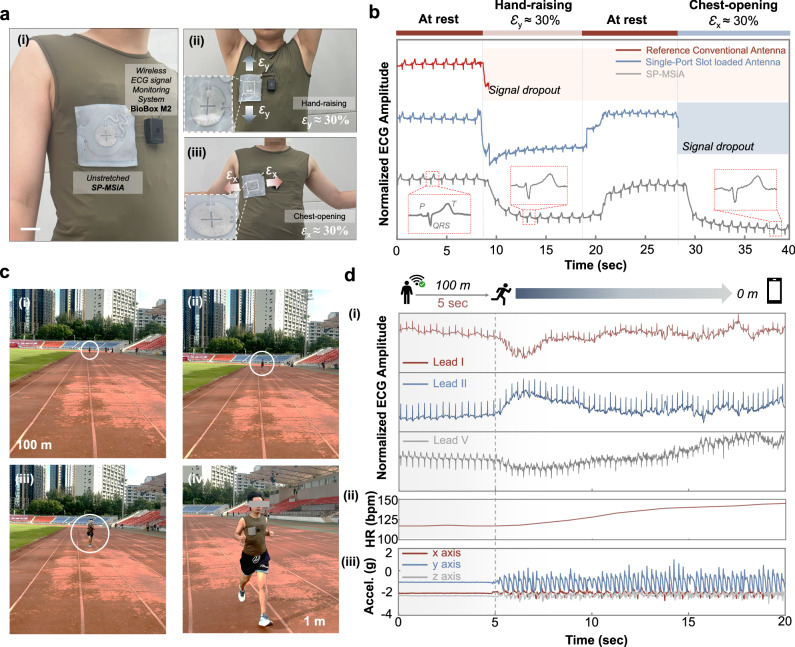
Real-world applications of SP-MSiA-based wearable electronics. **a** (i) On-body experimental setup of the SP-MSiA integrated with a wireless ECG monitoring system on the right pectoral region. Photographs showing the antenna under (ii) hand-raising (*ε*_*y*_ ≈ 30%) and (iii) chest-opening (*εₓ* ≈ 30%) motions. **b** Comparison of the wireless ECG signals recorded by the systems based on SP-MSiA, single-port slot-loaded antenna, and reference antenna before and during hand-raising and chest-opening movements. **c** Photographs of the outdoor running test showing a volunteer moving from 100 m to 1 m. **d** Real-time monitoring of (i) three-lead ECG, (ii) heart rate (HR), and (iii) triaxial acceleration signals during running.

In practical wearable applications, the complexity of human motion induces multidirectional dynamic deformation at the skin-device or cloth/device interfaces, even within the same skin region (Fig. 7a–i). The integrated device system undergoes inevitable yet distinct directional deformations during various body movements in daily activities, such as hand raising (*ε*_*y*_ ≈ 30%) (Fig. 7a–ii) and chest opening (*εₓ* ≈ 30%) (Fig. 7a–iii). Both the systems based on reference and single-port slot-loaded antennas exhibit pronounced signal dropouts when subjected to hand-raising (*ε*_*y*_ ≈ 30%) or chest-opening (*εₓ* ≈ 30%) motions, indicating severe strain-induced frequency detuning and link failure (Fig. 7b, red and blue). In contrast, the SP-MSiA allows the integrated system to stably and clearly distinguish the P wave, QRS complex, and T wave of ECG signals both before and during motions, demonstrating robust wireless communication capability under dynamic deformations (Fig. 7b, gray).

Outdoor field evaluations further confirm the stable communication capability of the integrated system based on the SP-MSiA during rapid and dynamic body movements over long communication distances, as the volunteer is running from 100 m toward the receiver (Fig. 7c). The continuously recorded ECG, heart rate (HR), and triaxial acceleration signals during exercise show the difference between pre- and in-exercise states, as evidenced by the transition from the progressively increased HR from 117 to 145 bpm, a stable baseline to periodic motion-induced acceleration, and weak motion-induced noise in the robust ECG waveforms (Fig. 7d). These results also demonstrate the feasibility of the integrated RF device system based on the multidirectional strain-insensitive SP-MSiA for robust and reliable sensing, paving the way toward the next-generation, body-conformal wireless RF wearable systems.

In summary, this study presents a dual-port multi-directional strain-insensitive stretchable antenna (DP-MSiA) featuring a cross-shaped slot at the center of the circular patch, in which the effective electrical length remains invariant under multidirectional strain through engineered current redistribution. By leveraging the intrinsic, significant mechanical-electromagnetic coupling feature of the DP-MSiA, further integration of two rectifying circuits for wireless energy harvesting (DP-MSiA-based WEH rectenna) and a power divider (SP-MSiA) for data transmission achieves stable and multidirectional strain-insensitive wireless power and end-to-end communication, respectively. In addition, the integration of the SP-MSiA with a wearable physiological monitoring system provides a proof-of-concept demonstration of stable health information tracking under direction-dependent deformation induced by dynamic body motions and over a wide range (>100 m) of wireless operation. The demonstrated results also open new opportunities in self-powered sensing nodes for soft body area networks or smart Internet of Things. The simple yet effective electrical-length compensation design strategy proposed in this work lays a robust foundation for developing stable and efficient wireless RF stretchable electronics, providing new design insights for other deformable systems demanding robust wireless functionality.

## Methods

### Materials

Gallium (99.99%) and indium (99.995%) were purchased from Luciteria, USA. Anhydrous ethanol (200 proof, ≥99.5%) was provided by Koptec (Decon Labs Inc., USA). Ecoflex 00-30 was obtained from Smooth-On, USA. Polyethylene terephthalate (PET) film and adhesive polyimide (PI) film were provided by 3 M, USA.

### Fabrication and preparation of liquid metal particles (LMPs) ink

LMPs ink was prepared by tip sonication (NE-1000Z, Fangxu Inc.) of 2.3 g pristine liquid metal with a Ga/In weight ratio of 74.5/25.5 in 20 mL anhydrous ethanol, using a sonicator operated at 40% power and 20 kHz frequency in pulse mode (2 s on / 4 s off) for 5 min.

### Fabrication of the DP-MSiA and SP-MSiA

The fabrication of DP-MSiA/SP-MSiA started with patterning an adhesive PI film (30 μm thick) attached to a 3D-printed ABS mold (WeNext, featuring a 150 × 150 × 2 mm recessed rectangular cavity) into the designed patch pattern by an ultraviolet laser (LPKF Laser U4, power: 0.4 μJ; speed: 2000 mm s^−1^; wavelength: 355 nm). Subsequently, LMPs ink was air-sprayed onto the 3D-printed mold placed on a 50 °C hot plate by an airbrush (MeiPen-116, 0.8 mm nozzle diameter) with a loading of 1 μL/mm^2^, where the elevated temperature facilitated ethanol evaporation for uniform LMPs coating. Meanwhile, the ground layer was fabricated using the same process, except that the PI mask was attached onto a PET film (70 μm thick) instead of the 3D printed mold. After manual removal of the excessive part in the PI masks, Ecoflex precursors (base-to-curing agent ratio of 1:1) were cast into the recessed cavity of the mold until fully filled, after which the ground layer was laminated on top of the mold. After curing at room temperature for 2 hours, peeling off the PET film and the released polymer substrate layer from the 3D-printed mold transferred LMPs-based Patch and Ground layer onto the top and bottom surface of Ecoflex substrate layer. Finally, the SubMiniature version A (SMA) connectors were subsequently soldered onto the two ports using conductive silver paste (XRK-8000C), followed by curing at 120 °C for 5 min to establish stable electrical contacts. A 100-ohm resistor was also soldered onto the SP-MSiA using the same conductive silver paste.

### Fabrication of the DP-MSiA-based WEH platform

The fabrication process of the DP-MSiA-based WEH platform followed a similar procedure to that of the DP-MSiA, except for slight modification of the mold material/shape and the ground integrated configuration for the introduced deep (2 mm) via interconnections. Specifically, a CNC-machined aluminum (WeNext) mold was modified by introducing several cylindrical pillars with a height of 2.2 mm, slightly exceeding the recessed depth of the mold to prevent the Ecoflex precursor covering the pillar tops, thereby ensuring electrical connection between the top patch and the ground layer. As for the ground layer, the LMPs-based ground pattern was first formed by spraying the LMPs ink through a PI mask onto a glass substrate, followed by spin-coating 8 g of Ecoflex precursor (500 rpm, 10 s) over the patterned surface and subsequent curing and peeling off from the glass substrate to form the Ecoflex-transferred LMPs layer. Next, the liquid metal side of the pre-fabricated Ecoflex-LMPs Ground layer was placed onto the mold (containing the LMP-patch pattern and uncured Ecoflex). Releasing the entire structure from the mold after Ecoflex curing formed the integrated DP-MSiA-based WEH platform. Moreover, all the electronic components (i.e., resistor, capacitor, LED, and Schottky diode) were also soldered using the same conductive silver paste with surface mount technology (SMT) stencils.

### Finite element analysis simulation

The mechanical deformation in the antennas was simulated in COMSOL Multiphysics 6.1, with the solid mechanics physics interface in the structural mechanics module employed for geometrically nonlinear static analysis. A shell model with a thickness of 10 µm was chosen for the thin patch in the simulation. The Neo-Hookean model with Young’s modulus of 125 kPa and Poisson’s ratio of 0.49 was applied for Ecoflex. The boundary conditions were applied with one edge fixed and the opposite edge prescribed displacement to impose the target tensile strain. The antennas before and after deformation were imported into CST Studio Suite 2023 for simulation. CMA was first performed using the Multilayer Solver with the CMA source type (no driven ports). The Ecoflex substrate was modeled as lossless with *ε*_r_ = 3.06 (tan *δ* = 0), and the patch was assigned as a perfect electrical conductor (PEC), consistent with the standard lossless CMA formulation. The ground was not modeled but idealized as an infinite PEC ground by imposing an electric-wall boundary (*E*_t_ = 0). The CMA sweep (1-4 GHz) extracted modal significance and eigencurrent distributions versus strain magnitude and direction. Full-wave frequency-domain simulations were then conducted on the corresponding driven antenna models with the inset feed and feedlines. The patch and ground were assigned EGaIn conductivity (*σ* = 1 × 10^5^ S/m) and Ecoflex was modeled with *ε*_r_ = 3.06 and tan *δ* = 0.02. Radiation (open and added space) boundaries were applied, with 50-ohm ports exciting the structure. Adaptive meshing with local refinement at feed/slot edges was used. A 1-4 GHz sweep was performed to obtain S-parameters, Z-parameters, and surface-current distributions. The power divider was simulated similarly.

### Characterization of mechanical-electromagnetic properties

The stress-strain curve of Ecoflex-LMPs composite was measured by the M230pro (YiGao) at a tensile speed of 30 mm/min. Strain-dependent S-parameters were measured using a custom-built nonmagnetic and nonconductive stretcher (Fig. S46a) connected to a VNA (T5260A, Transcom Instruments, 50 kHz-6 GHz) calibrated by the open-short-load-through method to the SMA reference plane. The antenna was clamped at both ends, and uniaxial strain was applied by increasing the clamp separation with side adjustment screws. The tensile strain was defined as (*L*_1_−*L*_0_)/*L*_0_, where *L*_0_ and *L*_1_ are the characteristic gauge lengths of the patch (Fig. S46b) along the loading direction before and after stretching, respectively. The strain level was verified from direct length measurement after each stretching step. Direction-dependent measurements were performed by aligning the loading axis with the x, 45°, y, and 135° directions (Fig. S46c). Unless otherwise noted, these measurements were conducted in free space without textile, phantom, or on-body loading.

An open-short-load-through (SOLT) calibration was conducted to set the measurement reference plane at the SMA connector. For single-port antennas, the antenna was soldered to a single SMA connector and connected to one VNA port via a coaxial cable. For dual-port antennas, two SMA connectors were used to connect each port to one VNA port, enabling simultaneous acquisition of S-parameters (S_11_, S_12_/S_21_, S_22_) as the antenna was stretched to different strain levels. The radiation patterns of the SP-MSiA and its non-slotted counterpart, both before and after stretching, were measured in a conventional far-field anechoic chamber. For the stretched cases, the antenna samples were pre-strained to the desired tensile levels and fixed in place at the center of the chamber using adhesive tape on a low-permittivity foam support. The antenna under test remained stationary, while a directional horn antenna (receiving probe) mounted on a motorized ring rotated to scan the angular range. Polarization alignment between the antenna under test and the horn was maintained for all measurements. The received signal was recorded at each angular position to obtain the radiation patterns under different strain conditions.

The strain-dependent measurements of the proposed antenna were conducted in free space because the performance difference between free-space and wearable conditions is small. As confirmed by the additional on-tissue, on-phantom, and representative on-body/on-garment results (Fig. S42), body loading causes only slight resonance shifts and negligible degradation in impedance matching, mainly due to the shielding effect of the ground plane. Therefore, the free-space stretching measurements provide a reasonable and controlled approach for evaluating the intrinsic effect of mechanical deformation on the antenna response.

### Demonstrations of RF energy harvesting

For the LED-illumination demonstration, an RF signal at 2.45 GHz with an output power of 3 dBm was generated using a tunable signal generator (SMA100B, Rohde & Schwarz) connected to a power amplifier and a commercial transmitting wall-mounted patch antenna (2.4 G, Aihua). The three wireless energy harvesting systems were positioned 1 m from the transmitter. The rectennas were tested under a 90° off-boresight orientation to reduce the received RF power, making the effect of mechanical deformation more distinguishable. The rectenna output was directly connected to an LED as the load, with the LED state under different stretching conditions visually recorded.

For the quantitative WEH experiment using a 160-ohm load, the experiment was performed using a 3 dBm signal generator followed by a 30 dB power amplifier, corresponding to a power of approximately 33 dBm delivered to the transmitting antenna. A commercial transmitting antenna with a gain of approximately 19 dBi was used, and the transmitting and receiving antennas were arranged in boresight alignment with matched linear polarization at a separation distance of 1 m. The rectenna output was connected to a fixed 160-ohm load resistor, and the DC output voltage across the load was measured under different strain levels and stretching directions. The measured output voltage was then converted into DC output power according to *P*_DC_ = *V*^2^/*R*, where *R* = 160 ohm. The normalized RF-to-DC efficiency was further calculated as *P*_DC_/*P*_r,0_, where *P*_r,0_ is the estimated RF power received by the rectenna in the unstrained state.

### Demonstrations of end-to-end far-field wireless communication of SP-MSiA

The wireless communication end-to-end performance of the SP-MSiA was evaluated by a BLE-based commercial ECG monitoring kit (BioBox M2, Sibet CAS). The kit comprised a smart T-shirt for ECG signal capture, a portable acquisition box for signal processing with a 500 Hz sampling rate and 0.1-100 Hz bandpass filter and wireless transmission, and a tablet computer for real-time signal reception, heart rate calculation, and display. An IPEX connector was reserved on the acquisition box for connecting the measured antennas bonded on the surface of the smart T-shirt by an SMA-IPEX coaxial cable. The transmitting antenna was deformed by manually stretching the smart T-shirt to control the deformation magnitude and direction. Moreover, the strain values were approximately estimated using a simple gauge-length method based on the patch dimension measured before and after deformation. The received signals were then recorded by placing the receiver tablet at various distances from the transmitter to measure the effective communication distances (ECD) and link-failure strain.

## Supplementary information

Supplementary Information

Transparent Peer Review file

## Source data

Source Data

## Data Availability

All data supporting the findings of this study are available within the article and its supplementary files. Any additional requests for information can be directed to and will be fulfilled by the corresponding authors. Source data are provided with this paper.
