# Supplementary material for: Multidirectional strain-insensitive stretchable RF electronics
Source: Nat Commun. 2026 Jun 27;17:8041. doi: 10.1038/s41467-026-74900-5 (PMC13454596; doi:10.1038/s41467-026-74900-5)
Supplement: Supplementary file 1 [file 41467_2026_74900_MOESM1_ESM.pdf]

Supporting Information for

Multidirectional strain-insensitive stretchable RF electronics

Furong Yang[#], Senhao Zhang[#,*], Jinyao Zhang, Yao Tong, Jun Zhong, Junjie Zheng, Jiawei Li, Yichao Hu, Yangbo Yuan, Jia Zhu, Kai Xu, Cheng Zhang[*], Huanyu Cheng[*], Chaoyun Song[*]

[#]These authors contributed equally to this work. (**Furong Yang** and **Senhao Zhang)**
*To whom correspondence should be addressed. E-mail: smz5628@psu.edu (**S.Z.**), czhangseu@foxmail.com (**C.Z.**), huanyu.cheng@psu.edu (**H.C.**), chaoyun.song@kcl.ac.uk (**C.S.**)

This file includes:
Notes S1 to S5
Figure S1 to S46
Tables S1 and S2
References 1 to 29

Note S1. Classical cavity model for microstrip patch antennas

The classical cavity model views a microstrip patch as a thin cavity of height h, bounded by a perfect electric conductor on the bottom (the ground plane) and another on top (the patch) [1-3]. The substrate and conductor are assumed lossless, and the ground plane is taken as infinite so that finite-ground effects and dielectric/conductor losses are neglected, and the resonance is governed primarily by the patch geometry. At the radiating edges of the patch (top conductor), electromagnetic fields spread into the air instead of stopping abruptly, storing extra electric energy. This fringing makes the patch act slightly longer than its physical length and thus a lower resonant frequency [4,5].

Rectangular patch: For a classical rectangular patch of length L and width W on a substrate with relative permittivity ε_r and thickness h, the dominant mode is TM_{10}. The fringing at the radiating edges makes the patch electrically longer than its physical length to yield an effective electrical length (L_{eff}):

$$L_{\text{eff}} = L + 2\Delta L, \tag{1.1}$$

where the fringing length ΔL is expressed as[4-6]

$$\Delta L \approx 0.412 \, \frac{(\varepsilon_{\text{eff}} + 0.3)\left(\frac{W}{h} + 0.264\right)}{(\varepsilon_{\text{eff}} - 0.258)\left(\frac{W}{h} + 0.8\right)} h, \tag{1.2}$$

with ε_{eff} as the effective permittivity:

$$\varepsilon_{\text{eff}} \approx \frac{\varepsilon_r+1}{2} + \frac{\varepsilon_r-1}{2}\left(1+\frac{12h}{W}\right)^{-\frac{1}{2}}. \tag{1.3}$$

Consider the speed of light as a constant c, the fundamental resonance frequency (f_r) is then given as:

$$f_r \approx \frac{c}{2\, L_{\text{eff}}\sqrt{\varepsilon_{\text{eff}}}}. \tag{1.4}$$

Circular patch: For a circular patch of radius a, the dominant resonance is the TM_{11} mode (often written as TM^z_{11} in the cylindrical coordinate system)[5,7]. Fringing makes the disk electrically larger than its physical radius, which is captured by an effective radius a_{eff} and approximated as[7,8]

$$a_{\text{eff}} = a\sqrt{1 + \frac{2h}{\pi a\, \varepsilon_r}\left[\ln\left(\frac{\pi a}{2h}\right) + 1.7726\right]}. \tag{1.5}$$

The TM_{11} resonance frequency is then expressed as[7,8]:

$$f_r \approx \frac{X'_{11}\, c}{2\pi\, a_{\text{eff}}\sqrt{\varepsilon_{\text{eff}}}}, \tag{1.6}$$

where X'_{11} is the first root of $J'_1(x) = 0$ with $X'_{11} \approx 1.8412$ as the boundary condition for the circular TM_{11} cavity mode.

By expressing the TM_{11} resonance condition in terms of the guided wavelength as:

$$\lambda_g = \frac{c}{f_r\sqrt{\varepsilon_{\text{eff}}}}, \tag{1.7}$$

Eq. (1.6) can be written as

$$2\pi a_{\mathrm{eff}} = \mathrm{X}'_{11}\lambda_{\mathrm{g}}. \tag{1.8}$$

With the equivalent half-wavelength cavity length identified as $L_{\mathrm{eff,\,circ}} = \lambda_{\mathrm{g}}/2$, the effective electrical length of the circular patch is

$$L_{\mathrm{eff,circ}} = \frac{\pi a_{\mathrm{eff}}}{\mathrm{X}'_{11}}. \tag{1.9}$$

Note S2. Thin-substrate limit and sensitivity of the resonant electrical length

When the substrate thickness h is much smaller than the lateral dimensions of the patch (width W for a rectangular patch and diameter $D = 2a$ for a circular patch), the resonance conditions can be simplified considerably.

Rectangular patch: The factor $1/\sqrt{1+12h/W}$ in Eq. (1.3) approaches unity to give $\varepsilon_{\mathrm{eff}} \approx \varepsilon_{\mathrm{r}}$. For $W/h \gg 10$ in our design, $(W/h + 0.264)/(W/h + 0.8)$ also approaches unity to simplify Eq. (1.3) as

$$\Delta L \approx 0.412 \frac{(\varepsilon_{\mathrm{r}}+0.3)}{(\varepsilon_{\mathrm{r}}-0.258)} h. \tag{2.1}$$

Since $h \ll L$, the fringing length becomes negligible ($2\Delta L \ll L$) to result in $L_{\mathrm{eff}} = L$. Substituting these approximations into Eq. (1.4) gives the resonance frequency as:

$$f_{\mathrm{r}} \approx \frac{c}{2\, L_{\mathrm{eff}} \sqrt{\varepsilon_{\mathrm{eff}}}} = \frac{c}{2\,(L+2\Delta L)\sqrt{\varepsilon_{\mathrm{eff}}}} \approx \frac{c}{2L\sqrt{\varepsilon_{\mathrm{eff}}}}, \tag{2.2}$$

Thus, the sensitivity $\partial \ln f_{\mathrm{r}} / \partial \ln L$ becomes -1.

Circular patch: For $h \ll a$ in our design, a_{eff} in Eq. (1.5) approaches a and the resonance frequency in Eq. (1.6) can be expressed as:

$$f_{\mathrm{r}} \approx \frac{X'_{11}\, c}{2\pi\, a_{\mathrm{eff}}\sqrt{\varepsilon_{\mathrm{eff}}}} \approx \frac{X'_{11}\, c}{2\pi\, a\sqrt{\varepsilon_{\mathrm{eff}}}} = \frac{X'_{11}\, c}{\pi\, D\sqrt{\varepsilon_{\mathrm{eff}}}}. \tag{2.3}$$

Thus, the sensitivity $\partial \ln f_{\mathrm{r}} / \partial \ln a$ also becomes -1, and the effective electrical length can be expressed as:

$$L_{\mathrm{eff,circ}} = \frac{\pi a_{\mathrm{eff}}}{X'_{11}} \approx \frac{\pi a}{X'_{11}} = \frac{\pi D}{2X'_{11}}. \tag{2.4}$$

Note S3. Strain-invariant condition for circular patch antennas with a slot

In the conventional case for a circular patch antenna operating in fundamental mode TM_{11}, its effective electrical length is given by Eq. (2.4). A central slot of unstrained length L_{slot0} (aligned with the x-direction) and width W_{slot0} perturbs the surface current and forces a detour around the slot. This increases the electrical path by an amount proportional to L_{slot0} as:

$$L_{eff} = \frac{\pi}{2X_{11}}\left(D_y + \alpha L_{slot0}\right), \tag{3.1}$$

where $\alpha \in (0,1]$[9–11] is the effectiveness factor. It becomes 1 when the slot fully interrupts current flow and decreases when current partially bridges the slot capacitively.

Under uniaxial stretching along the x-direction:

With small-strain linearization (i.e., only the first-order term)[12-15] and a nearly incompressible elastomer ($v \approx 0.49$ - 0.50 for Ecoflex[16]), uniaxial tensile strain ε_x applied along the x-direction results in increased slot length (aligned with x-direction) and decreased D_y (perpendicular to the x-direction) due to Poisson contraction:

$$D_y(\varepsilon_x) \approx D_0(1\text{-}v\varepsilon_x), \tag{3.2}$$

$$L_{slot}(\varepsilon_x) \approx L_{slot0}(1+\varepsilon_x), \tag{3.3}$$

where D_0 is the unstrained diameter.

Substituting Eq. (3.2) and Eq. (3.3) into (3.1) and expanding to first order gives

$$L_{eff}(\varepsilon_x) = \underbrace{\frac{\pi}{2X_{11}}(D_0 + \alpha L_{slot0})}_{\text{Unstrained}} + \frac{\pi}{2X_{11}}\varepsilon_x(\alpha L_{slot0} - vD_0). \tag{3.4}$$

Because $f_{11} \propto L_{eff}^{-1}$, the fractional frequency shift (or normalized relative frequency change) becomes

$$\left.\frac{\Delta f}{f}\right|_x \approx -\frac{\alpha L_{slot0} - vD_0}{D_0 + \alpha L_{slot0}}\,\varepsilon_x. \tag{3.5}$$

In Eq. (3.5), the numerator expresses the compensation from slot elongation ($+\alpha L_{slot0}\varepsilon_x$) and Poisson contraction ($-vD_0\varepsilon_x$) of the patch diameter.

The first-order strain-invariant condition is achieved when the fractional frequency shift is zero, which gives:

$$L_{slot0} = \frac{vD_0}{\alpha}. \tag{3.6}$$

Under this condition, Poisson's contraction exactly offsets slot elongation, keeping L_{eff} constant as the initial condition, achieving strain-invariance for small strain.

Under uniaxial stretching along the y-direction:

The patch diameter along the y-direction increases and the slot shortens due to the Poisson effect:

$$D_y(\varepsilon_y) \approx D_0(1 + \varepsilon_y), \tag{3.7}$$

$$L_{slot}(\varepsilon_y) \approx L_{slot0}(1 - \nu\varepsilon_y). \tag{3.8}$$

Repeating the same derivation as in the above for the uniaxial stretching along the x-direction gives

$$L_{slot0} = \frac{D_0}{\alpha\nu}. \tag{3.9}$$

For $\nu \approx 0.5$ and $\alpha \leq 1$, this would require $L_{slot0} \gtrsim 2D_0$, geometrically impossible for a central slot. Thus, full first-order invariance under stretching along the *y*-direction cannot be achieved with a single-slot topology, though partial compensation still occurs.

Validity of the linear approximation: second-order effects:

The derivations above are based on small-strain linearization. As the applied uniaxial strain increases, geometric nonlinearity becomes more significant. To assess the validity of the linear approximation, general second-order expansions of the geometric change are introduced [12]:

$$D(\varepsilon_x) \approx D_0(1\text{-}\nu\varepsilon_x + k_D\varepsilon_x{}^2), \tag{3.10}$$

$$L_{slot}(\varepsilon_x) \approx L_{slot,0}(1+\varepsilon_x+k_L\varepsilon_x{}^2), \tag{3.11}$$

where k_D and k_L are dimensionless coefficients capturing geometric and material nonlinearity. Substituting into Eq. (3.4) gives:

$$L_{eff}{}^{(2)}(\varepsilon_x) \approx \frac{\pi}{2X_{11}}\left[D_0+\alpha L_{slot,0}+\varepsilon_x(\alpha L_{slot,0}\text{-}\nu D_0)+\varepsilon_x{}^2(k_D D_0+\alpha k_L L_{slot,0})\right]. \tag{3.12}$$

Thus, the relative error in the total electrical length when using the linear approximation as in Eq. (3.4) is then:

$$\frac{|\Delta L|}{L_{eff}(0)} \approx \frac{L_{eff}{}^{(2)}(\varepsilon_x)\text{-}L_{eff}{}^{(1)}(\varepsilon_x)}{L(0)} = \varepsilon_x^2\,\frac{|k_D D_0+\alpha k_L L_{slot,0}|}{D_0+\alpha L_{slot,0}}. \tag{3.13}$$

For an ideal incompressible isotropic block under uniaxial tension, the standard kinematics give $\nu = 1/2$ and $k_D = 3/8$ for the lateral contraction [12][15], while the axial stretching is exactly $1+\varepsilon_x$, so $k_L=0$ [12][15]. For a tensile strain of 30%, $\varepsilon_x=0.3$ and thus $\varepsilon_x^2=0.09$, and the resulting relative error is on the order of 3% when D_0 and $L_{slot,0}$ are of similar magnitude. Thus, the second-order correction remains only a few percent even at 30% stretching, indicating that the linear approximation is sufficiently accurate for the strain range considered in this work. By substituting $\varepsilon_x = 0.10, 0.20, 0.30, 0.40$, and 0.45, the estimated second-order error is approximately 0.25%, 1.0%, 2.25%, 4.0%, and 5.1%, respectively. Since the resonant frequency scales approximately as $f \propto L_{eff}^{-1}$, these results indicate that the deviation from the first-order strain-invariant condition remains small throughout the experimentally studied range. Thus, although the strain-invariant condition is formally derived at the undeformed state through first-order linearization, the second-order correction remains only a few percent even at 45% stretching, indicating that this condition remains a reliable approximation across the full experimental strain range considered in this work.

Note S4. Partial slot-patch complementation for the tensile strain applied parallel to the current path direction

For tension applied along the y-direction, the first-order strain-invariant condition derived in Note S3 would require a central slot with $L_{slot0} \geq 2D_0$, which is not geometrically feasible for a slot confined within the patch. Full cancellation is therefore unattainable with a single central slot. Nevertheless, any finite slot introduces a Poisson-driven shortening of the slot as the patch expands along the y-direction, which counteracts part of the increase in electrical length from the radius increase, so partial compensation always occurs. The analysis below quantifies this reduction relative to the unslotted counterpart.

Unslotted circular patch

For an unslotted circular patch in the TM_{11} mode, according to the Eqs. (2.3) and (3.7), the frequency shift is:

$$\Delta f = \frac{X'_{11}\, c}{\pi D_0 (1+\varepsilon_y)\sqrt{\varepsilon_r}} - \frac{X'_{11}\, c}{\pi D_0 \sqrt{\varepsilon_r}}, \tag{4.1}$$

so the relative resonance frequency shift follows the first-order linear approximation

$$\frac{\Delta f}{f}\Big|_{y,\text{no slot}} = \frac{1}{1+\varepsilon_y} - 1 \approx -\varepsilon_y, \tag{4.2}$$

indicating that the frequency decreases proportionally to the applied strain.

Slot-loaded circular patch

Similarly, for a circular patch with a single central slot of length $L_{slot0} = a_0 = D_0/2$ aligned along the x-direction, combining Eqs. (2.3), (3.7), and (3.8) gives the first-order fractional frequency shift:

$$\frac{\Delta f}{f}\Big|_{y,\text{slot}, L_{slot0}=a_0} \approx -\frac{D_0 - \alpha v L_{slot0}}{D_0 + \alpha L_{slot0}}\varepsilon_y = -\frac{1-\alpha v/2}{1+\alpha/2}\varepsilon_y. \tag{4.3}$$

Here, the term $\alpha v/2$ in the numerator captures the partial compensation arising from Poisson-driven shortening of the slot as the radius increases, which reduces the net increase in electrical length.

Finally, the sensitivity ratio along the y-direction between the slotted and unslotted designs is compared via a slope-reduction factor **R**, defined as:

$$\mathbf{R} = \frac{\frac{\Delta f}{f}\Big|_{y,\text{ slot}}}{\frac{\Delta f}{f}\Big|_{y,\text{ no slot}}} = \frac{1 - \frac{\alpha v}{2}}{1 + \frac{\alpha}{2}},\ 0 < \alpha \leq 1,\ 0 < v < 0.5, \tag{4.4}$$

which quantifies how much the slot reduces the sensitivity along the y-direction.

Note S5. Extend the strain-invariant characteristic for higher strain levels.

In **Note S3**, strain invariance at the onset of stretching is obtained by setting the initial-condition relation $L_{slot0} = vD_0/a$ under stretching along the x-direction. That result is derived from the undeformed state $\varepsilon = 0$, which yields zero initial slope of the fractional frequency shift. Extending this invariance to a specific finite strain level ε' in the typical operating range can preserve strain-insensitive behaviour at higher strain levels.

After imposing the instantaneous invariance condition $L_{slot}(\varepsilon) = vD_y(\varepsilon)/a$ at the target strain ε'_x, solving for the required initial slot length gives:

$$L_{slot0}\left(\varepsilon'_x\right) = \frac{vD_0}{\alpha}\,\frac{1 - v\,\varepsilon'_x}{1 + \varepsilon'_x}. \tag{5.1}$$

Equivalently, for a chosen L_{slot0}, the strain at which the response becomes strain-invariant is given as:

$$\varepsilon'_x = \frac{\frac{vD_0}{\alpha} - L_{slot0}}{L_{slot0} + \frac{v^2 D_0}{\alpha}}. \tag{5.2}$$

Initially, since $L_{slot0}(\varepsilon'_x) = \frac{vD_0}{\alpha}\frac{1 - v\,\varepsilon'_x}{1 + \varepsilon'_x} < \frac{vD_0}{\alpha}$, the slot under-compensates at very small strain, and the frequency rises slightly. As the strain increases, slot length L_{slot0} increases, and at ε'_x, the strain-invariant condition $L_{slot0} = vD_0/a$ is met, producing a local plateau. For ε_x just above ε'_x, slot elongation and Poisson-induced contraction are comparable, so their effects nearly cancel, and the effective electrical length remains approximately constant over a finite strain range. Practically, for the tensile strain along the x-direction from 0 to ca. 50%, this produces the observed slight rise, flat, and slight fall in the resonance frequency, but the frequency variations are confined to a very narrow range (<1%) around the designed value and are thus quasi-strain invariant. It should be noted that only at very large strains, slot elongation dominates and the antenna operates in slot mode, leading to a more noticeable downward shift.

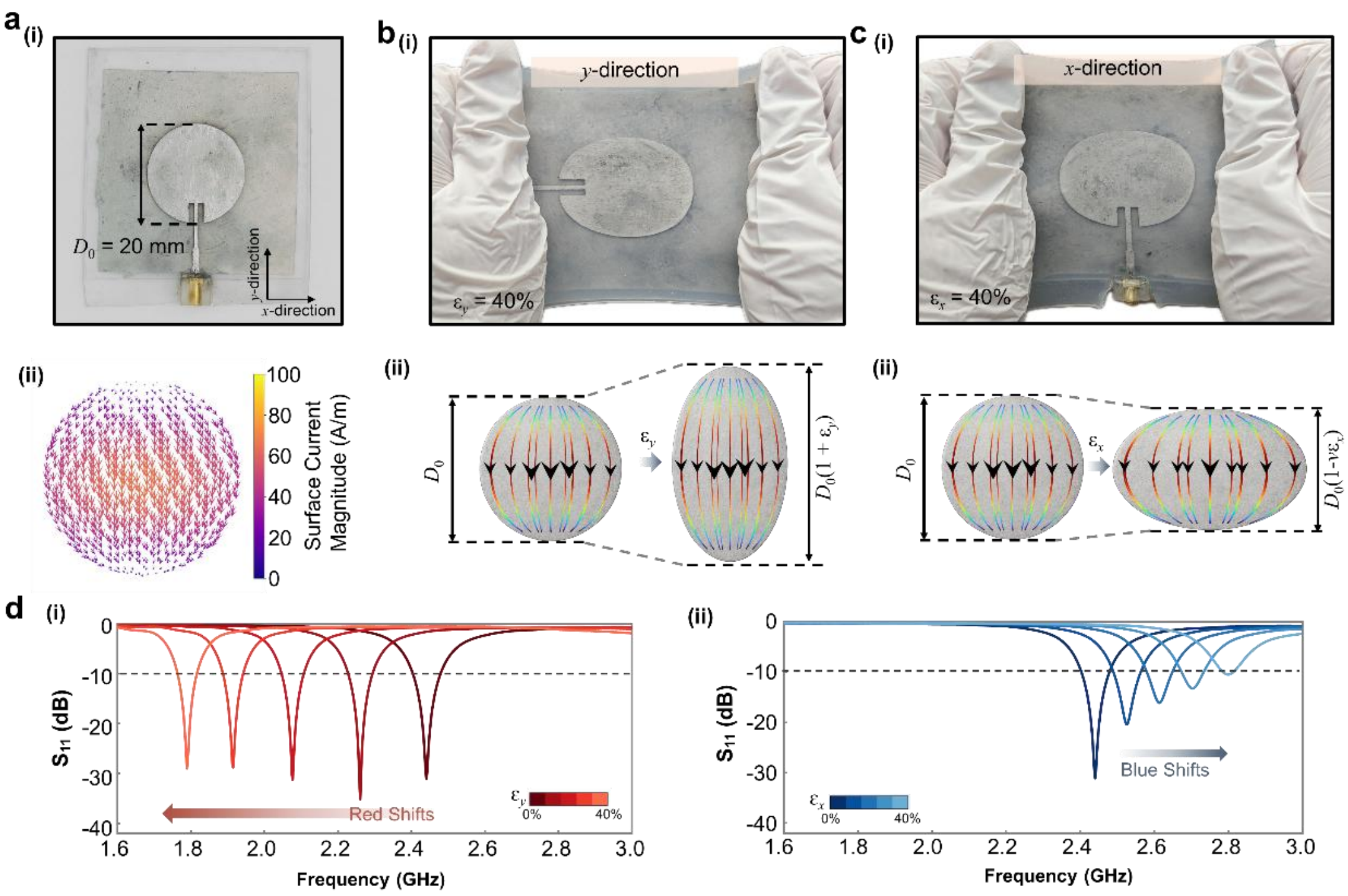

Figure S1. (i) Photographs and (ii) simulated surface currents (TM_{11}) of a conventional circular patch antenna (2.45 GHz) **(a)** before and **(b,c)** after under tensile strain applied along the **(b)** y- and **(c)** x-directions, along with **(d)** the measured S_{11} curve changes upon stretching along the (i) y- and (ii) x-directions.

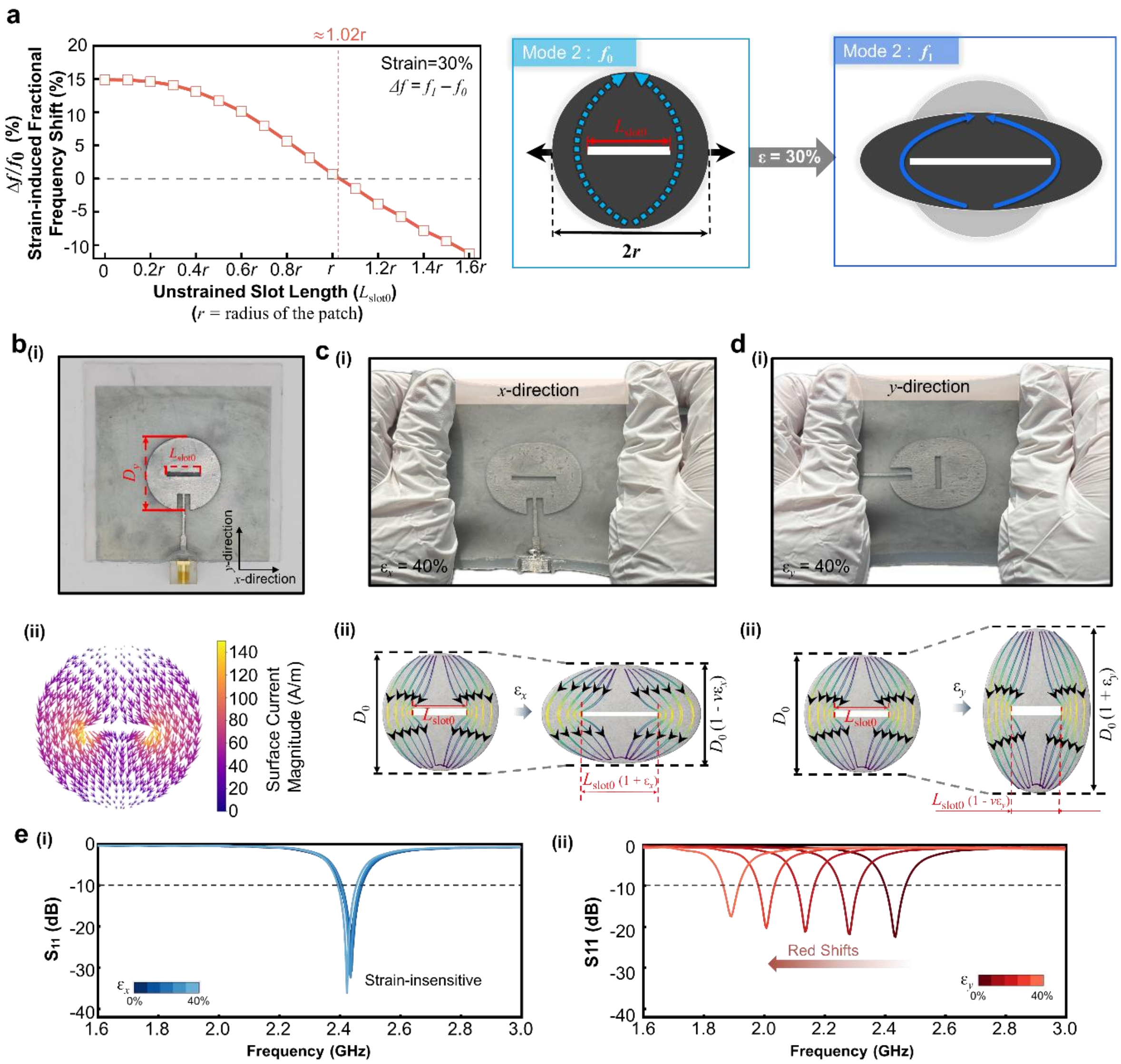

Figure S2. **(a)** Simulated fractional frequency shift $\Delta f/f_0$ versus unstrained slot length L_{slot0} under ε = 30% (showing the optimized L_{slot0} for strain-insensitive Mode 2). (i) Photographs and (ii) simulated surface currents (TM_{11}) of the optimized circular slot-loaded patch antenna (scaled to 2.45 GHz) **(b)** before and after under tensile strain applied in the **(c)** x- and **(d)** y-directions, along with **(e)** measured S_{11} curve changes upon stretching along the (i) x- and (ii) y-directions.

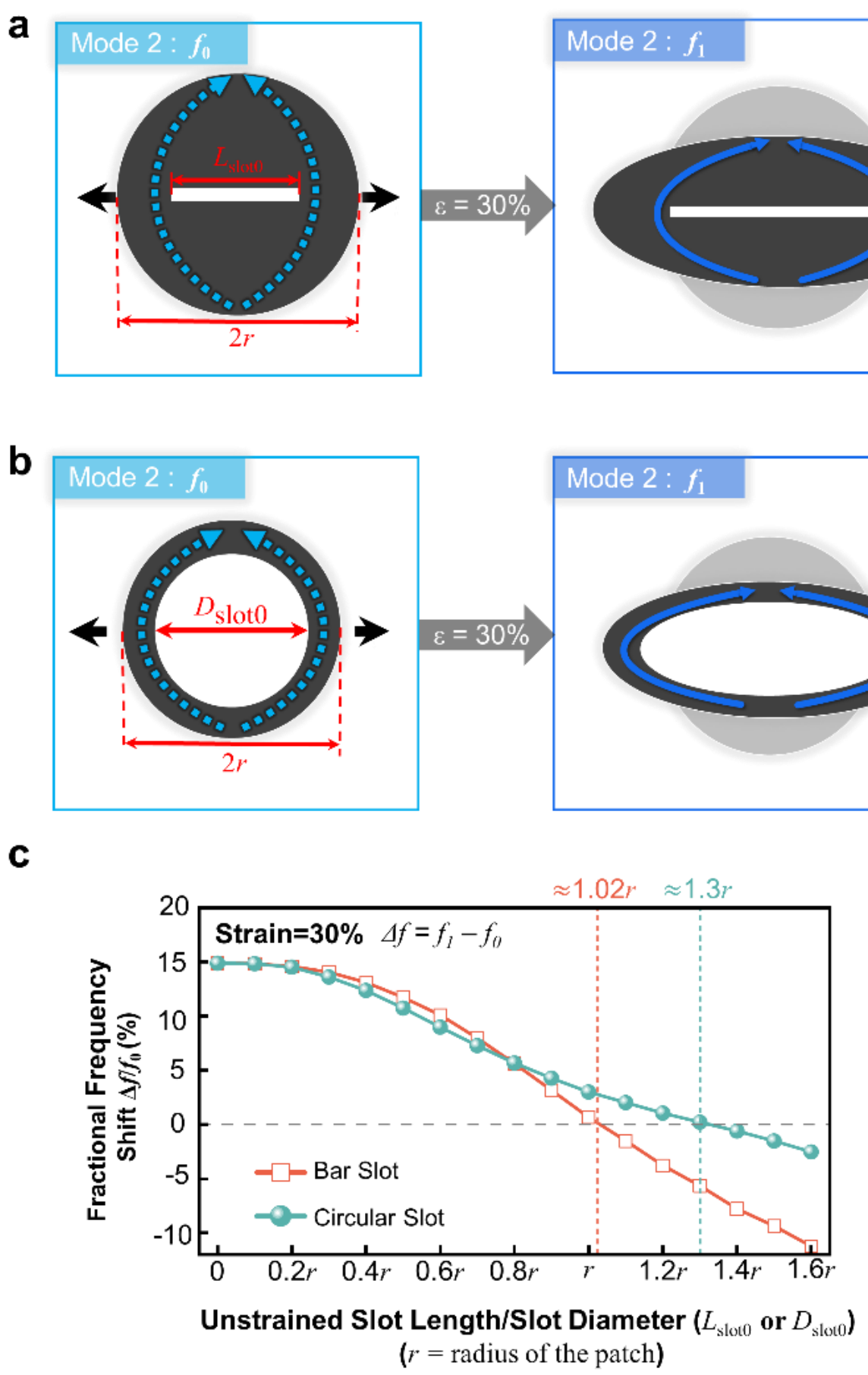

Figure S3. Comparison of bar and circular-slot designs for Mode 2. **(a)** Mode 2 schematic under strain for a bar slot with unstrained length L_{slot0}. **(b)** Mode 2 schematic under strain for a circular slot with unstrained diameter D_{slot0}. **(c)** Simulated Mode 2 fractional frequency shift $\Delta f/f_0$ versus normalized slot size for straight- and circular-slot designs, indicating the optimized values (≈1.02r and ≈1.3r, respectively).

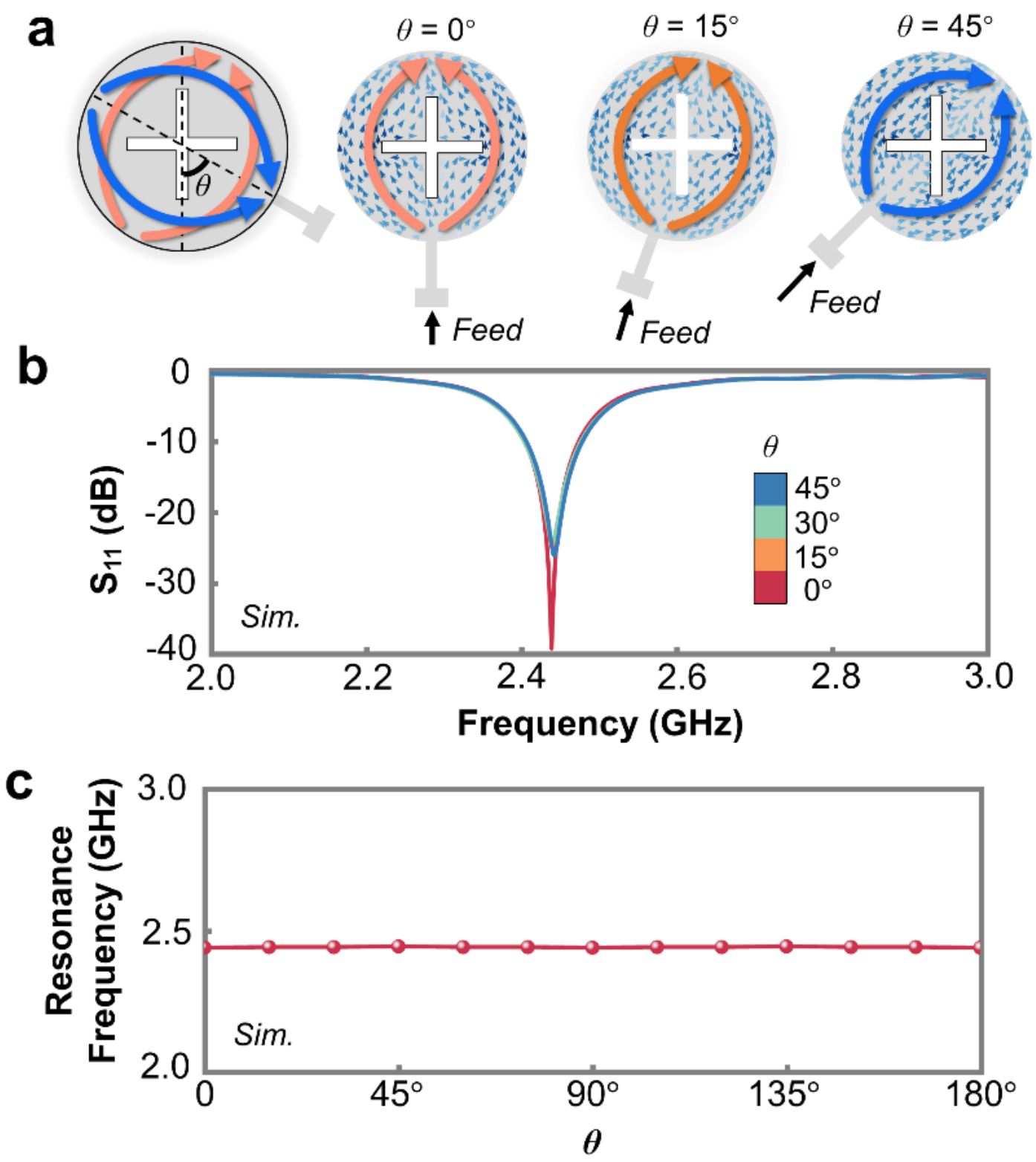

Figure S4. Quasi-rotationally symmetric Mode 2 behavior of the cross-slot design in the unstrained state. **(a)** Simulated Mode 2 surface-current distributions for representative in-plane orientation angles θ (0°, 15°, and 45°), where θ denotes the orientation of the excited Mode 2 current pattern measured from the y-axis. **(b)** Simulated S_{11} responses for θ = 0°, 15°, 30°, and 45°. **(c)** Extracted Mode 2 resonance frequency versus θ from 0° to 180°, showing nearly identical resonance frequencies across feed orientations.

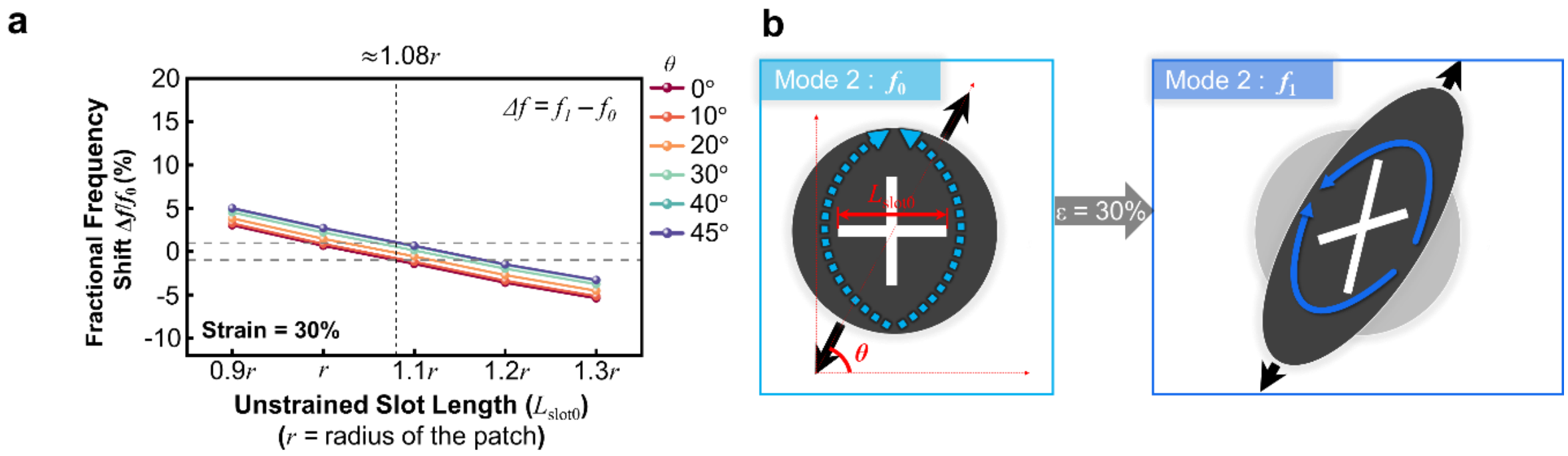

Figure S5. Orientation dependence of the Mode 2 strain-induced frequency response for cross-slot designs. **(a)** Simulated $\Delta f/f_0$ versus in-plane strain orientation for the cross-slot design. **(b)** Mode 2 schematics for the cross-slot design at $\theta = 60°$.

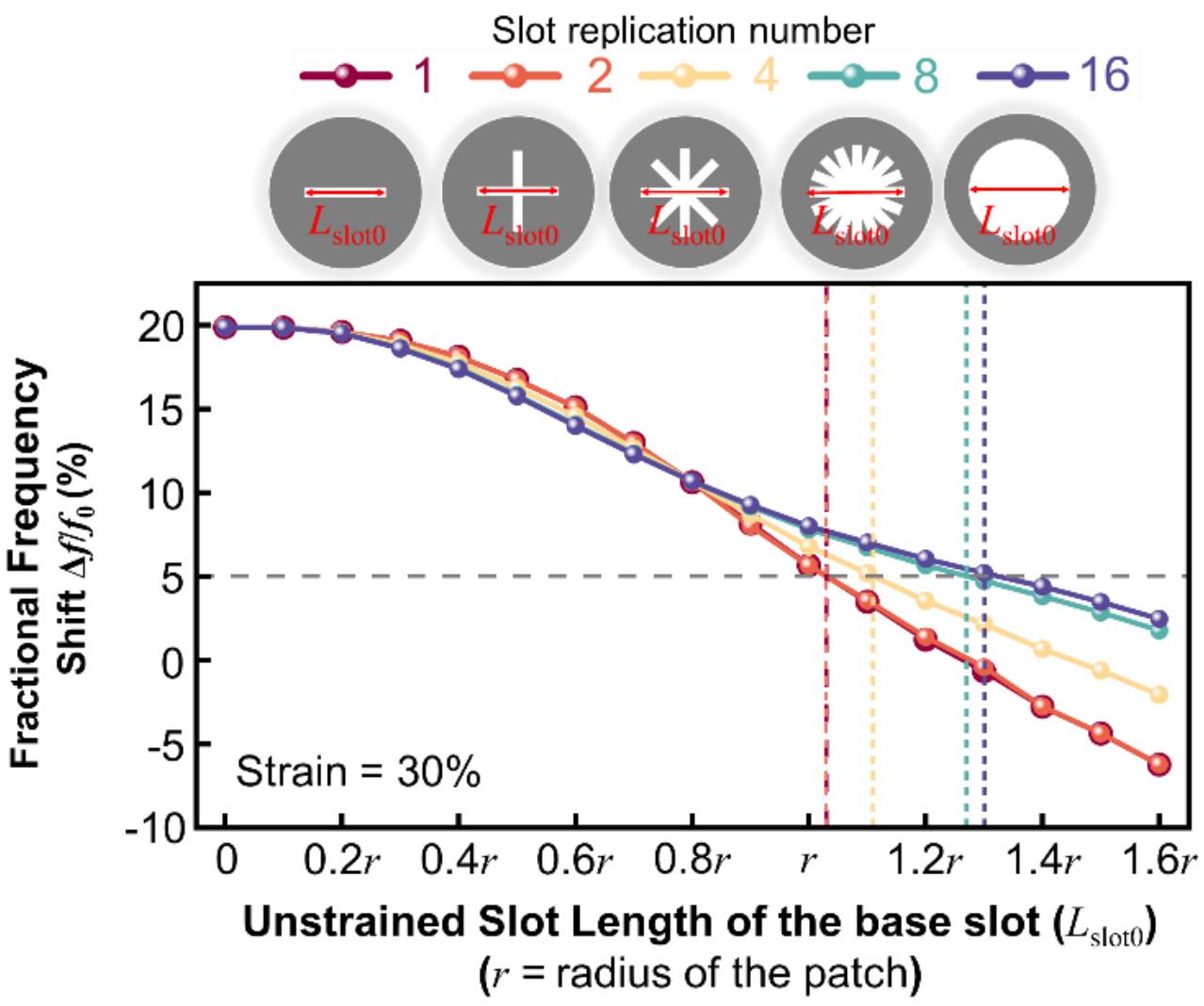

Figure S6. Simulated Mode 2 strain-induced fractional frequency shift $\Delta f/f_0$ at $\varepsilon = 30\%$ as a function of the unstrained length of the base slot (L_{slot0}), for different slot replication numbers. The base slot is a single bar-slot element that is replicated and rotated to form the multi-slot pattern. Vertical dashed lines indicate the L_{slot0} values that yield zero $\Delta f/f_0$ for each replication number.

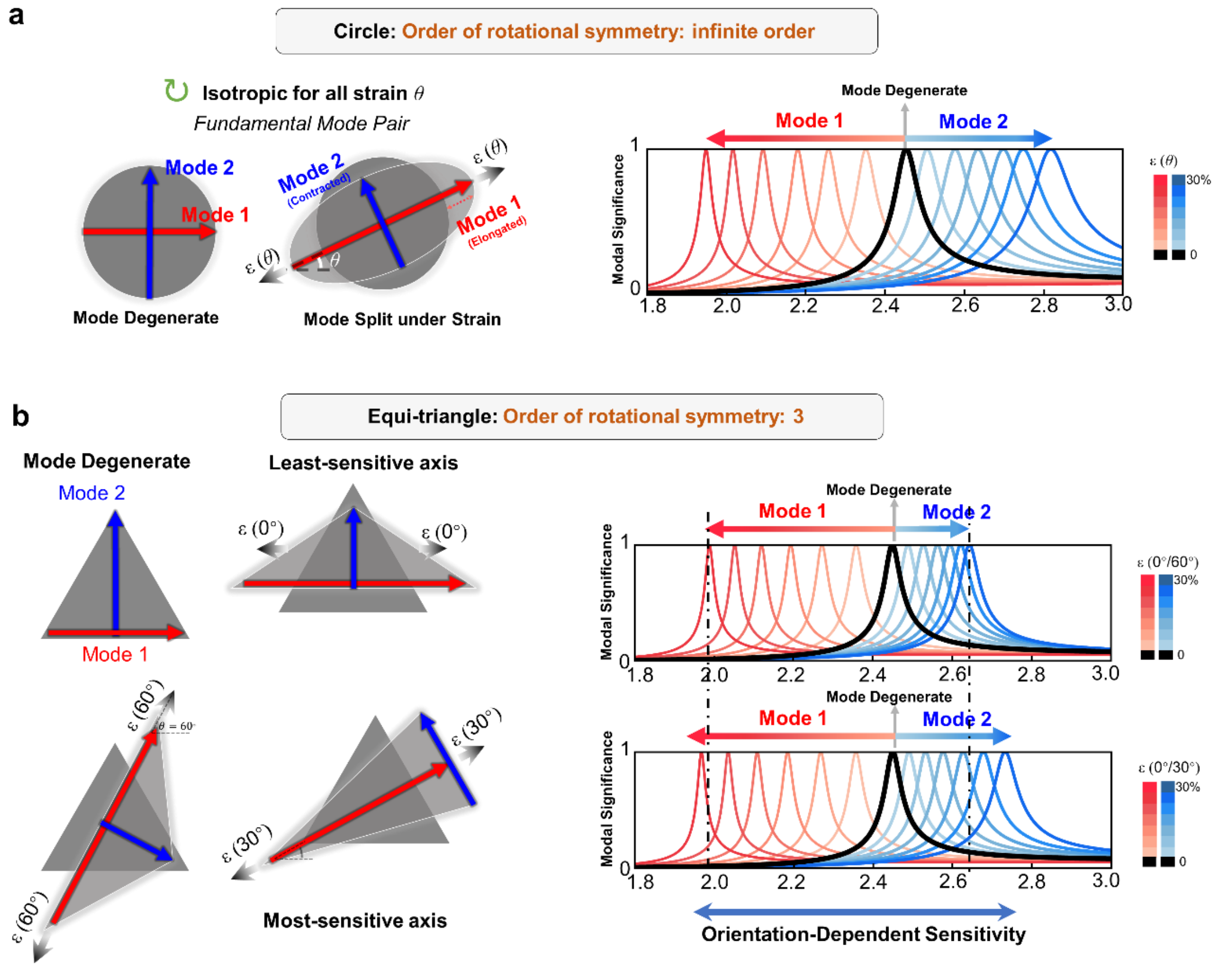

Figure S7. Simulated modal-significance versus frequency for a **(a)** circular patch and **(b)** an equilateral-triangle patch under uniaxial strain $\varepsilon(\theta)$ ranging from 0% to 30% in 5% increments. The circle exhibits strain-direction-independent mode splitting due to its infinite rotational symmetry. The equilateral triangle exhibits strain-direction-dependent splitting due to its lower rotational symmetry order.

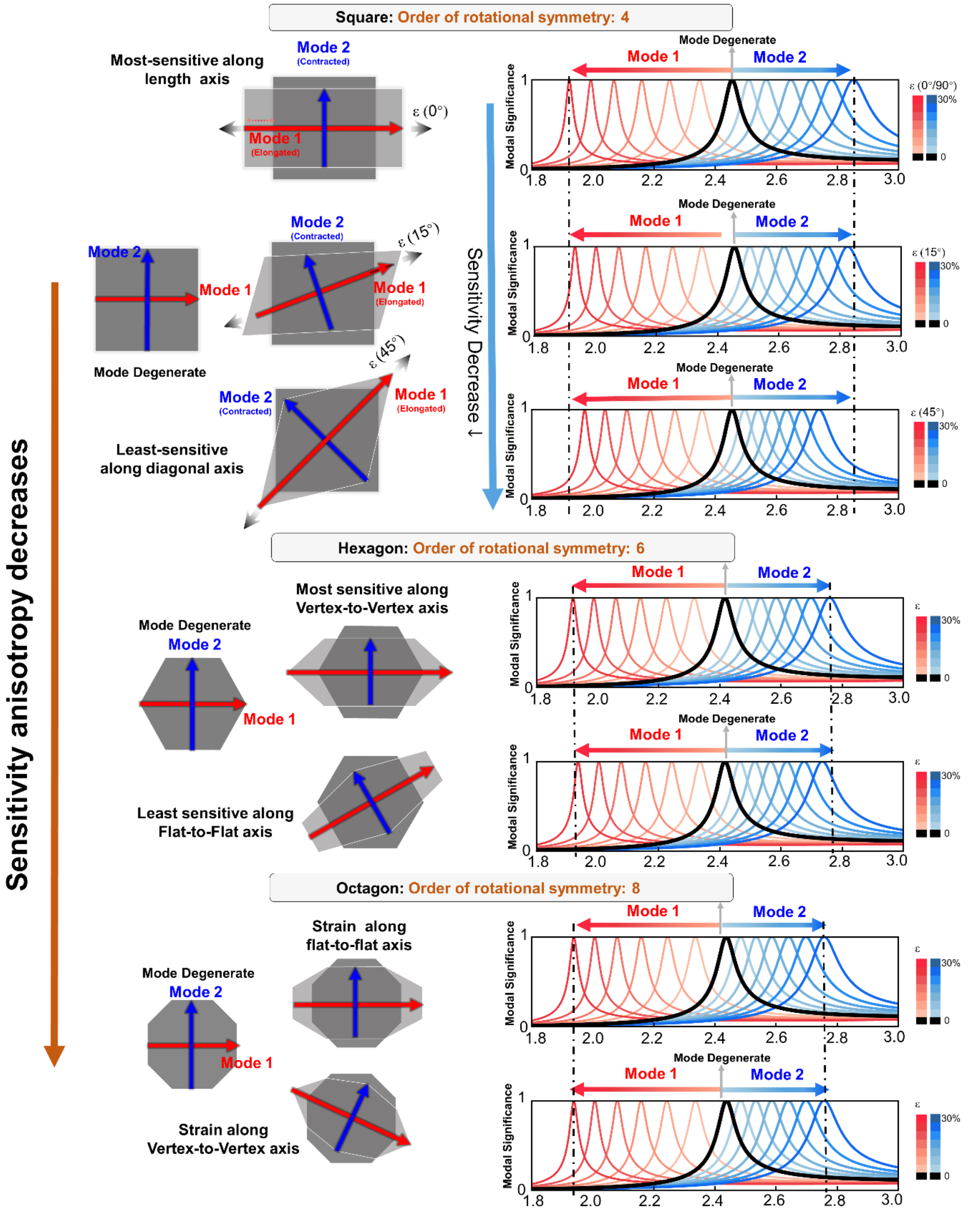

Figure S8. Simulated modal-significance spectra for a square, a regular hexagon, and a regular octagon cavity under uniaxial strain $\varepsilon(\theta)$ ranging from 0% to 30% in 5% increments. These cavities exhibit analogous orthogonal modes and strain-induced splitting. The strain-direction sensitivity anisotropy decreases as the rotational symmetry order increases.

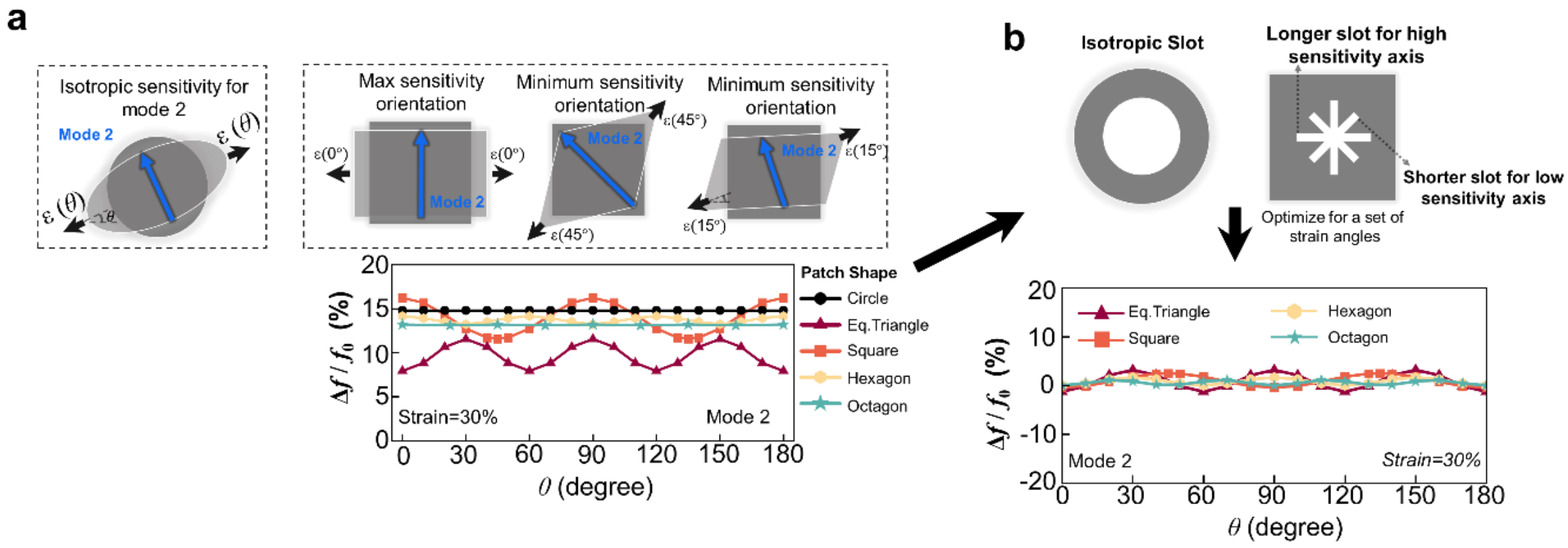

Figure S9. Symmetry-guided slot design for reducing strain-orthogonal mode (Mode 2) sensitivity under uniaxial strain. **(a)** Simulated strain-induced fractional frequency shift $\Delta f/f_0$ of Mode 2 versus strain orientation θ for different cavity shapes at $\varepsilon = 30\%$, illustrating isotropic response for the circular cavity and orientation-dependent response for polygonal cavities. **(b)** Slot-pattern design concept to suppress Mode 2 frequency shift by keeping the original rotational symmetry of the cavity while enabling complementary slot lengthening and shortening across strain orientations, yielding reduced $\Delta f/f_0$ versus θ at $\varepsilon = 30\%$.

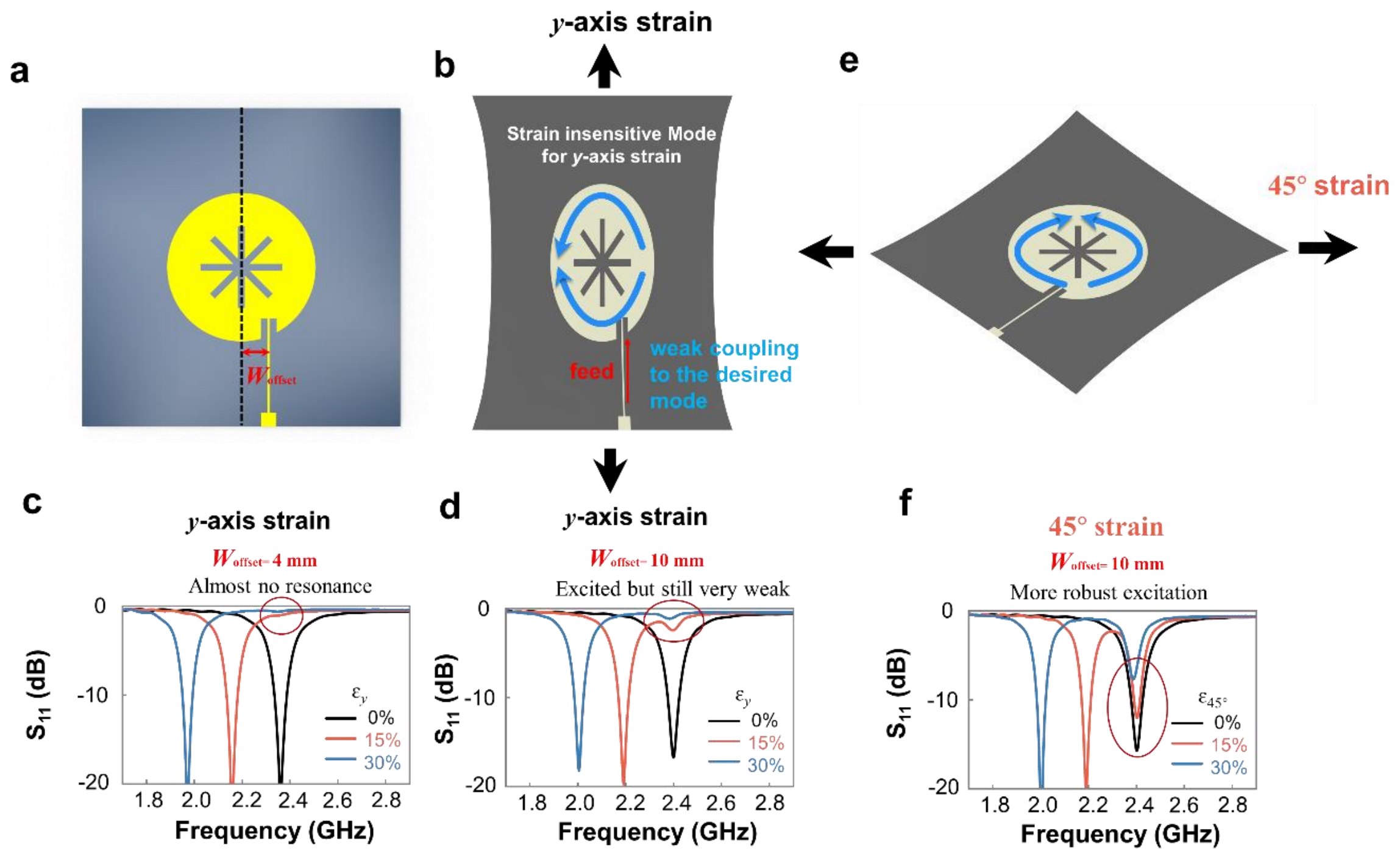

Figure S10. Limitation of single-feed excitation for multidirectional strain-insensitive operation. **(a)** Offset-fed single-port antenna design with feed offset (W_{offset}). **(b)** Schematic showing weak coupling of the strain-insensitive mode under y-axis stretching because it is orthogonal or nearly orthogonal to the feed direction. Simulated reflection responses showing that the desired mode is absent for **(c)** a small offset, only weakly excited for **(d)** a large offset under y-direction strain. **(e)** Schematic of the 45° stretching direction. **(f)** Simulated reflection responses for W_{offset} =10 mm under 45°-direction strain, where the desired mode becomes more clearly excited.

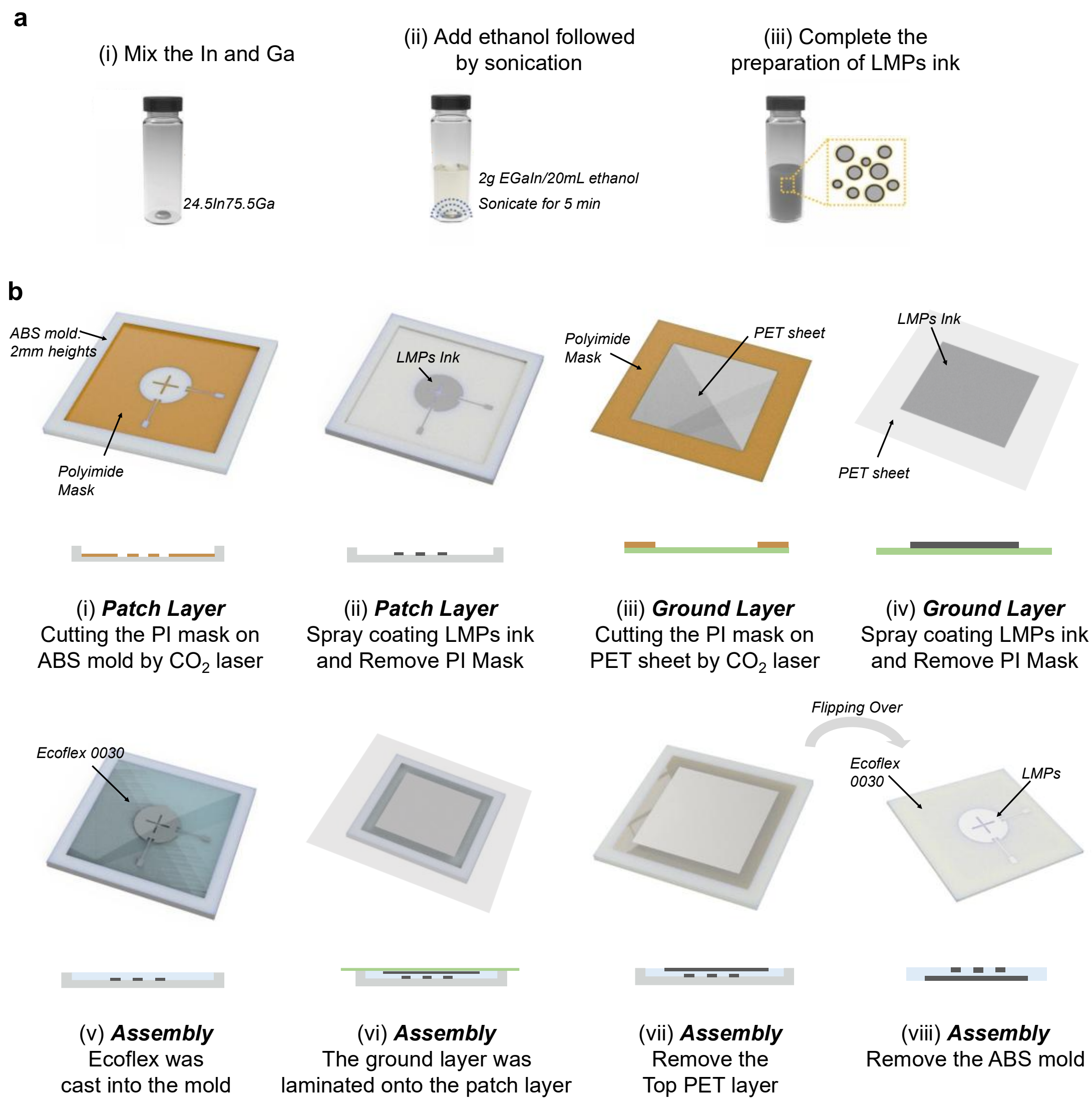

Figure S11. Schematic showing the fabrication process of Ecoflex-LMPs composite-based stretchable dual-port multidirectional strain-insensitive antenna (DP-MSiA): from **(a)** preparation of liquid metal particle (LMPs) ink to **(b)** fabrication of the (i, ii) patch layer, (iii, iv) ground plane layer, followed by (v-viii) assembly.

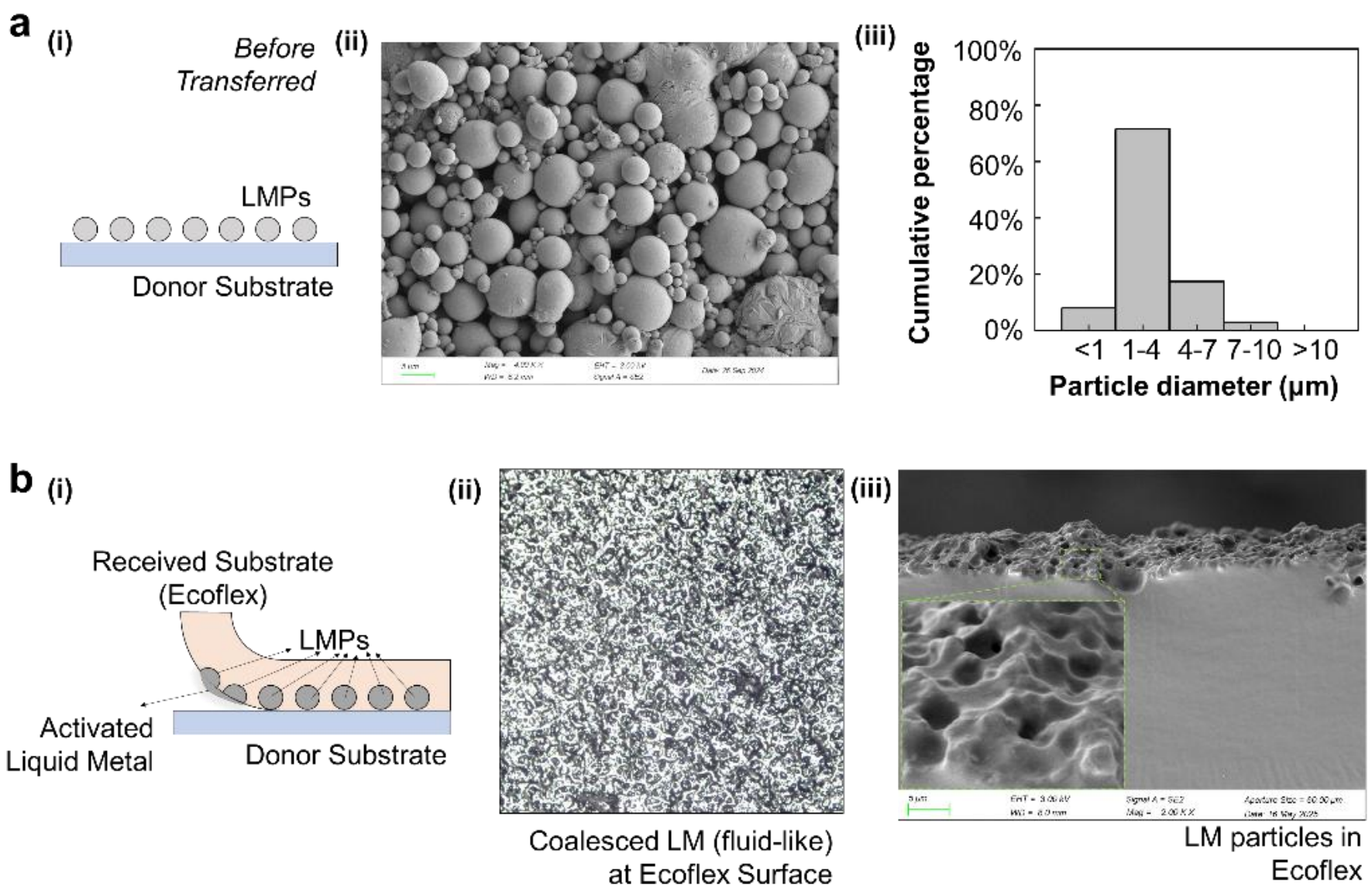

Figure S12. Microstructure evolution of liquid metal particles (LMPs) before and after transfer. (a) (i) Schematic illustration of LMPs on the donor substrate prior to transfer. **(ii)** SEM image and **(iii)** particle size distribution of LMPs after ultrasonication. **(b) (i)** Schematic illustration of the transfer process from the donor substrate to the Ecoflex substrate. **(ii)** Optical image showing the coalesced, fluid-like morphology of liquid metal at the Ecoflex surface. **(iii)** Cross-sectional SEM image revealing liquid metal particles embedded near the Ecoflex surface, forming interconnected conductive pathways.

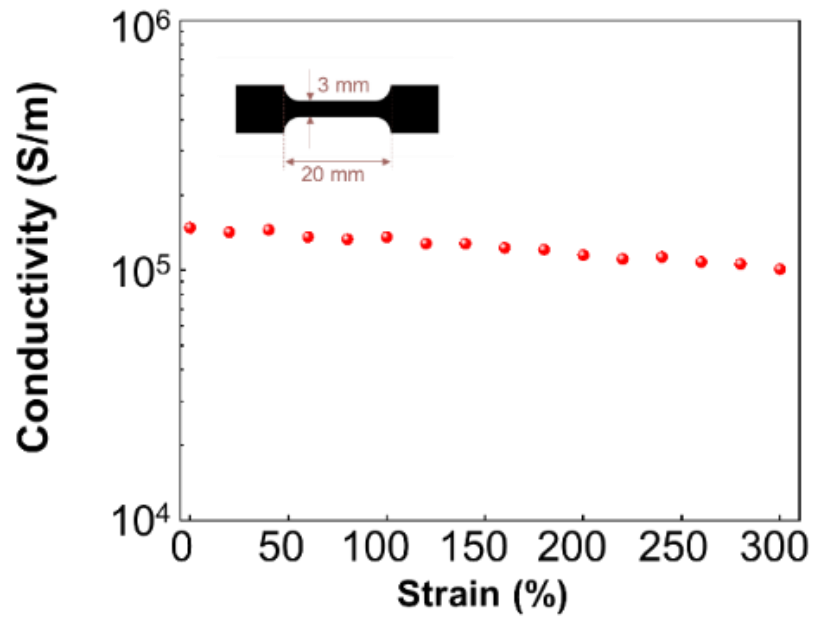

Figure S13. Conductivity–strain curve of the LMPs in a standard dumbbell shape (width of 3 mm in the middle) printed on the Ecoflex00-30 substrate.

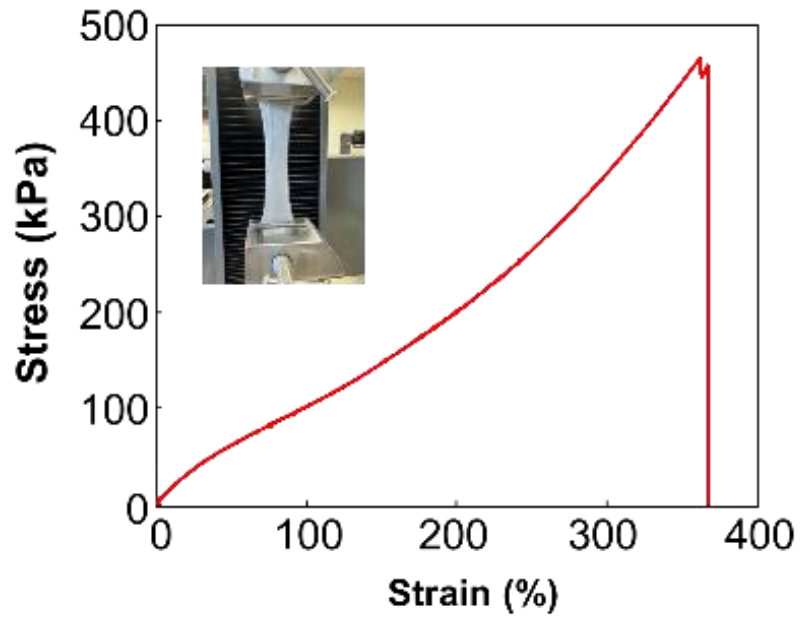

Figure S14. Uniaxial tensile stress-strain curve of the LMPs trace printed on the Ecoflex00-30 substrate.

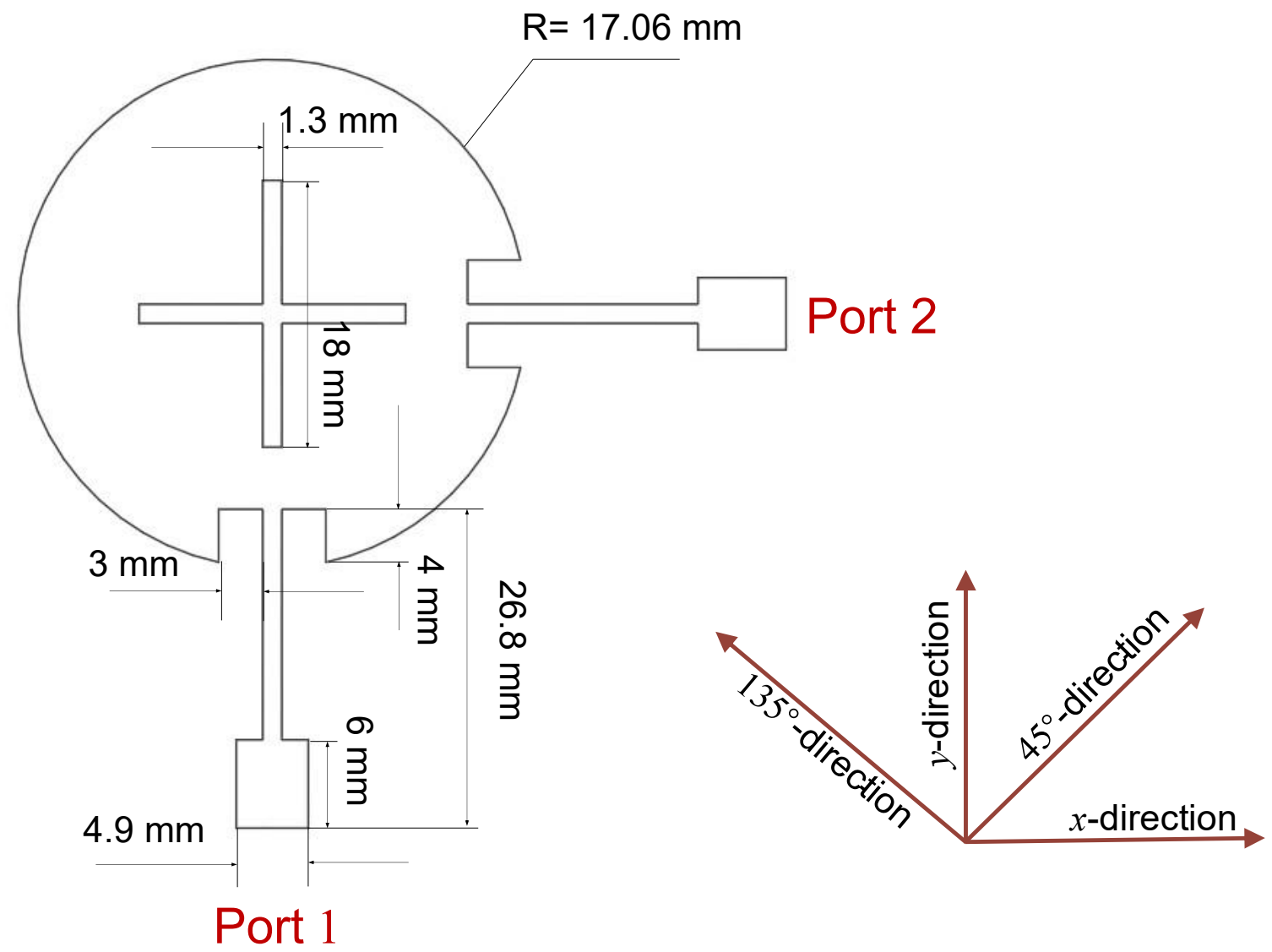

Figure S15. Geometrical dimensions of Ecoflex-LMPs composite-based stretchable dual-port multidirectional strain-insensitive antenna (DP-MSiA).

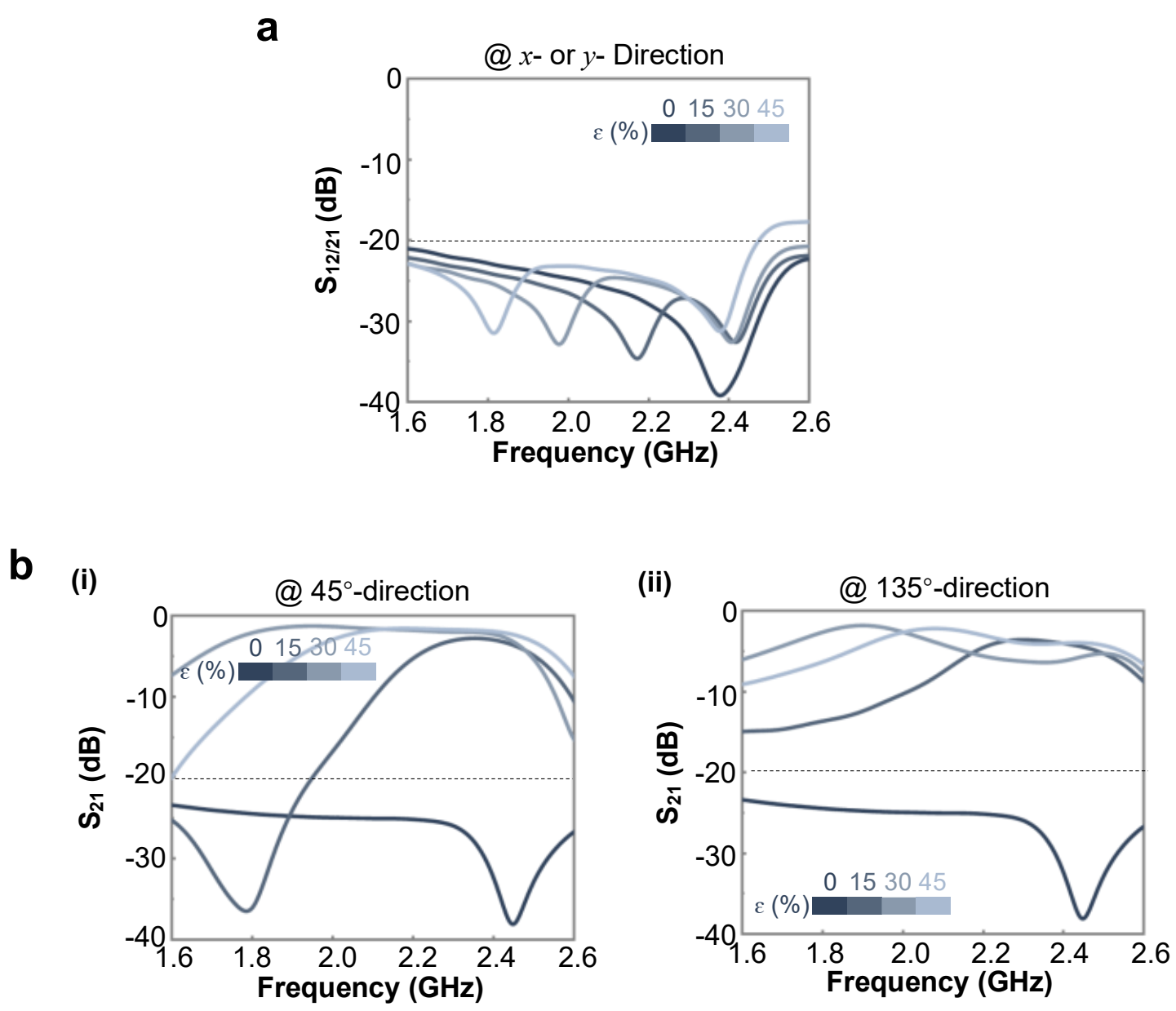

Figure S16. Measured $S_{12/21}$ of DP-MSiA under stretching of 0, 15, 30, and 45% along **(a)** x/y, **(b)** (i) 45°, and (ii) 135° directions.

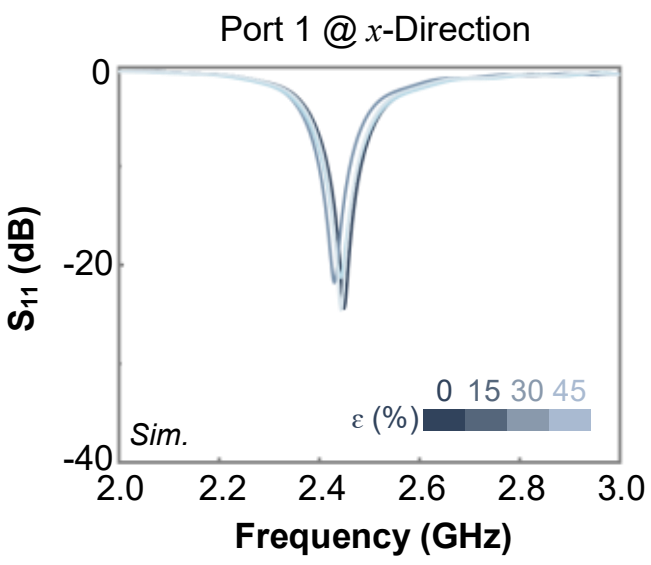

Figure S17. Simulated S_{11} from port 1 of the DP-MSiA under stretching of 0, 15, 30, and 45% along the x-direction.

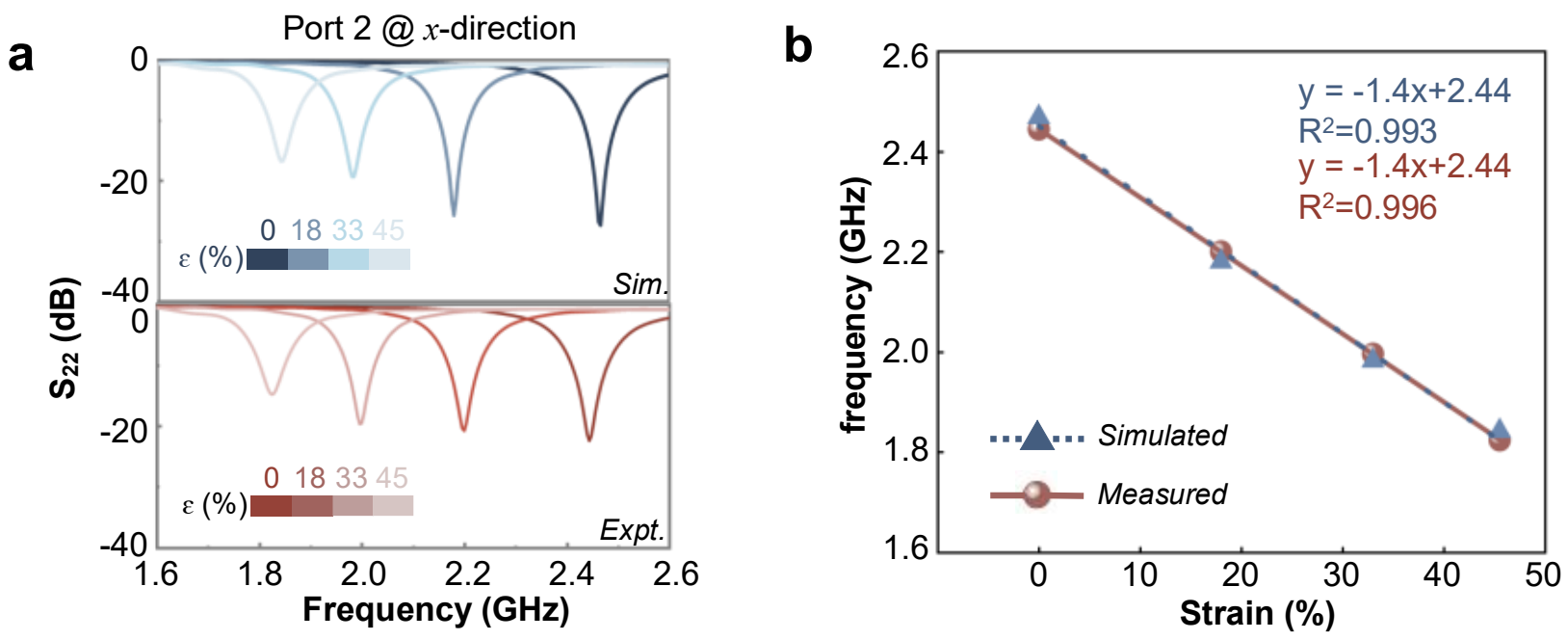

Figure S18. **(a)** Measured (red) and simulated (blue) S_{22} from port 2 of the DP-MSiA under stretching of 0, 18, 33, and 45% along the x-direction, and **(b)** the corresponding comparison between measured (red) and simulated (blue) resonance frequency as a function of the applied tensile strain along the x-direction.

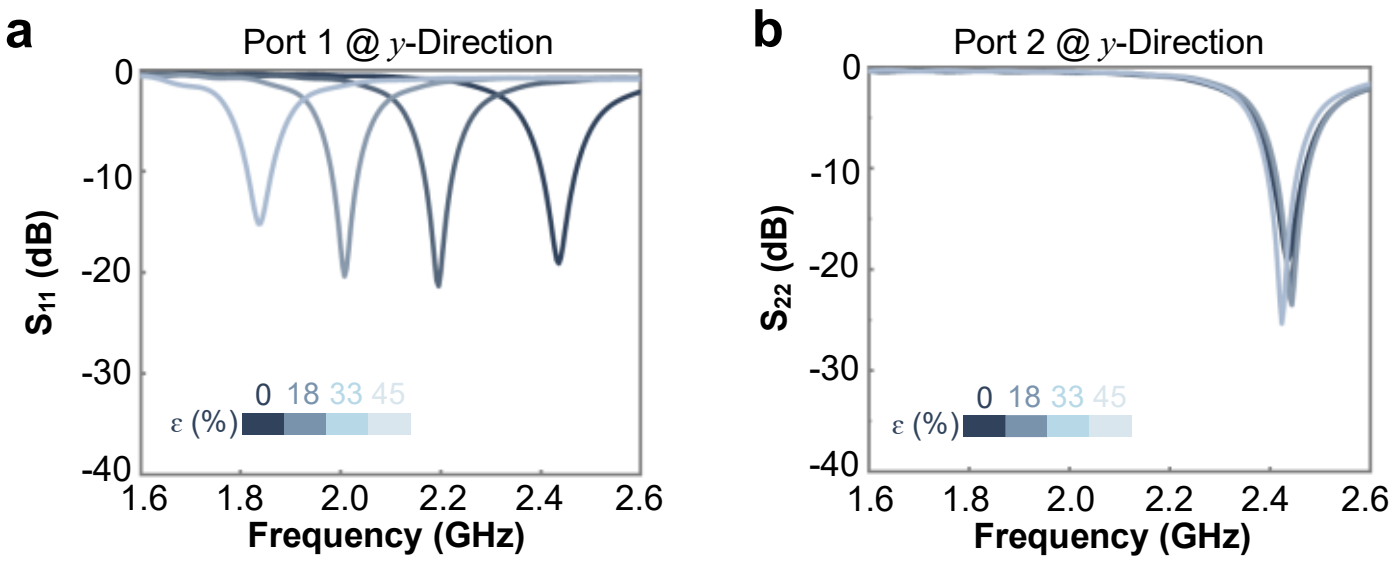

Figure S19. Measured **(a)** S_{11} from port 1 and **(b)** S_{22} from port 2 under stretching of 0, 18, 33, and 45% along the y-direction.

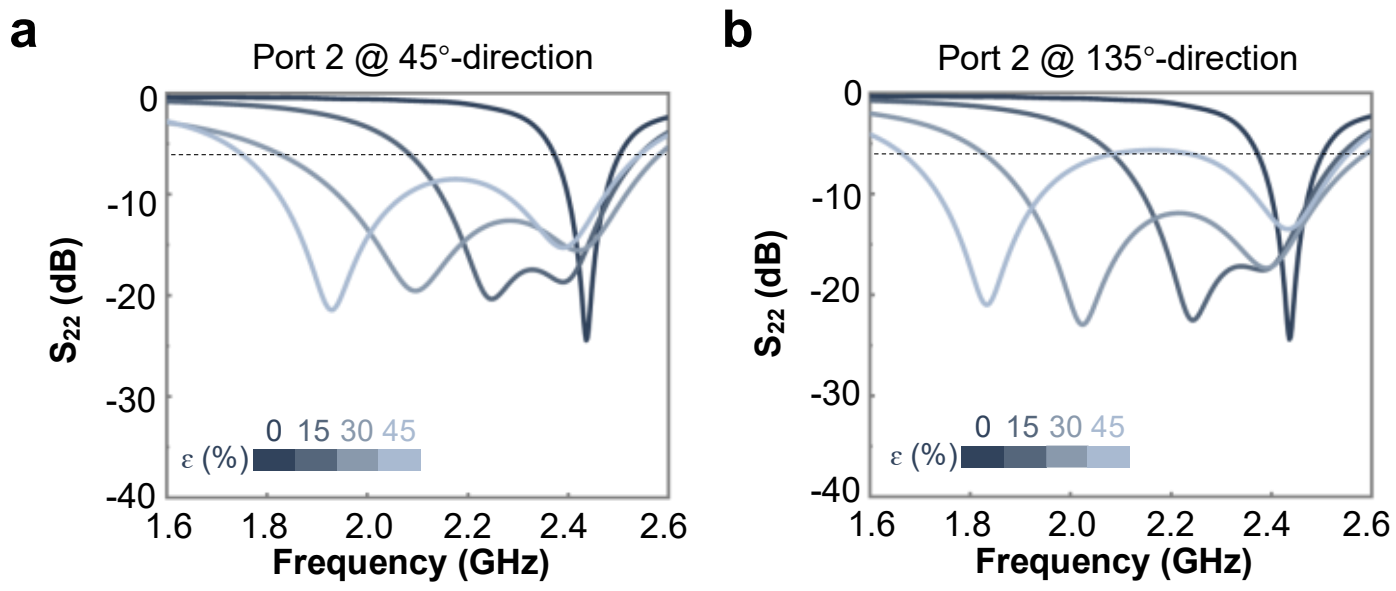

Figure S20. Measured S_{22} of DP-MSiA under stretching of 0, 15, 30, and 45% along **(a)** 45°-direction and **(b)** 135°-direction.

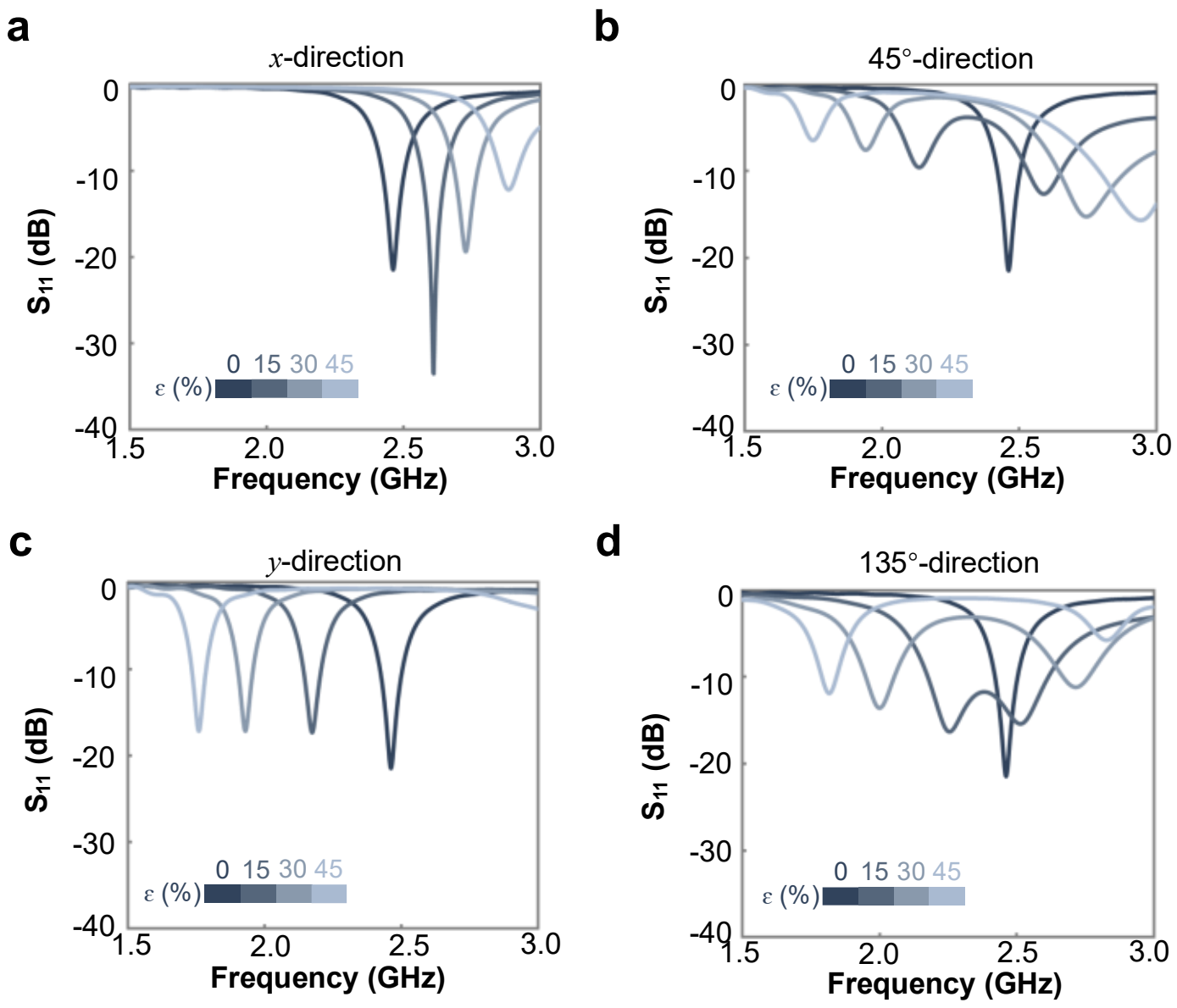

Figure S21. Measured S_{11} of conventional dual-port antenna without slot under stretching of 0, 15, 30, and 45% along **(a)** x, **(b)** 45°, **(c)** y, and **(d)** 135° directions.

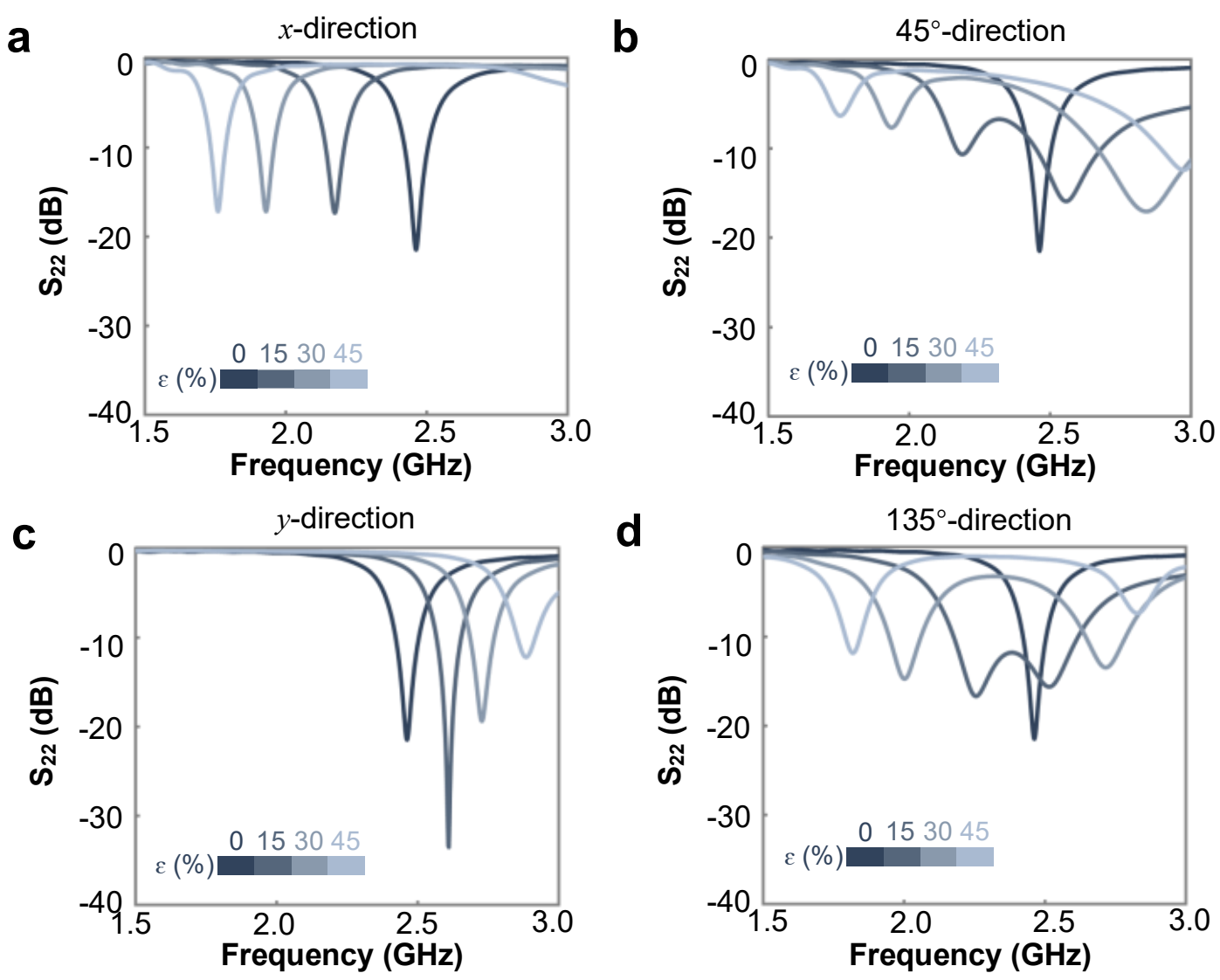

Figure S22. Measured S_{22} of conventional dual-port antenna without slot under stretching of 0, 15, 30, and 45% along **(a)** x, **(b)** 45°, **(c)** y, and **(d)** 135° directions.

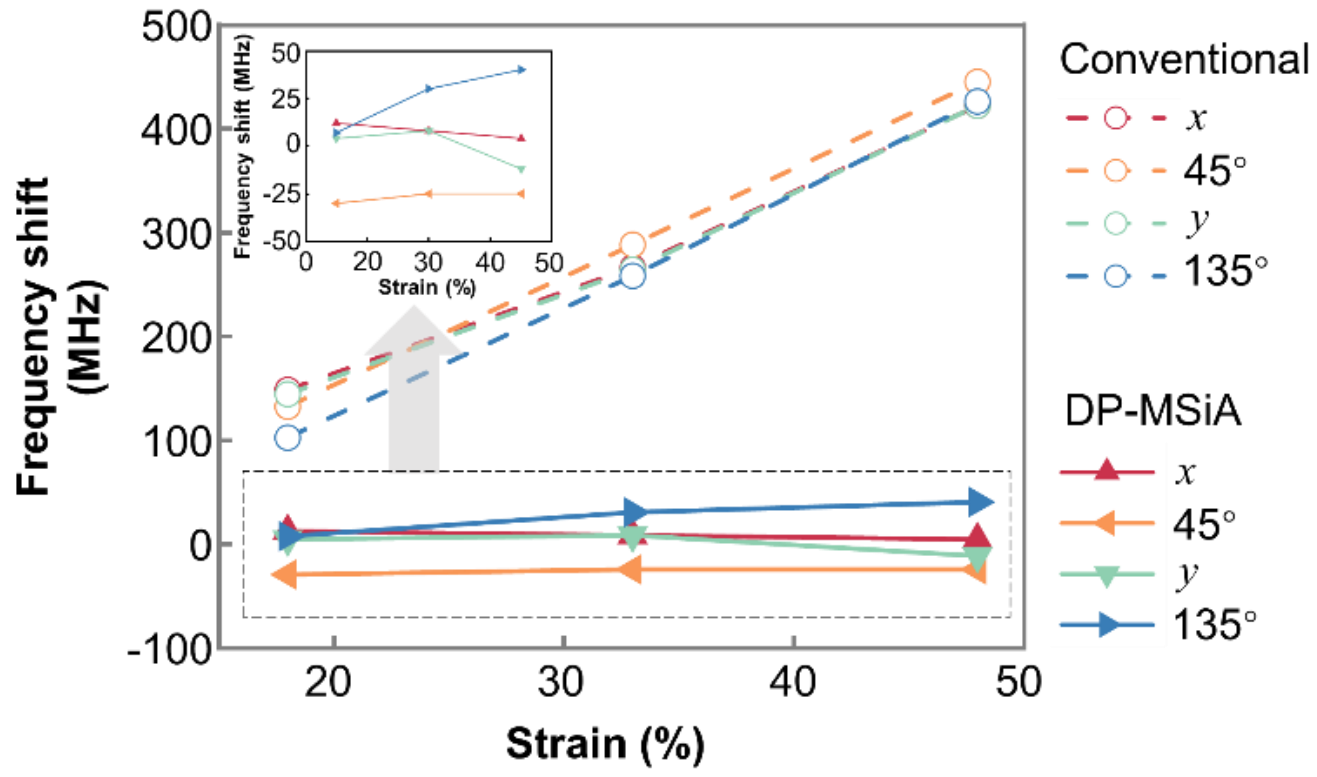

Figure S23. Extracted frequency shift (Δ*f*) of the proposed DP-MSiA and the conventional antenna as a function of strain level under four representative in-plane stretching directions (x, 45°, y, and 135°).

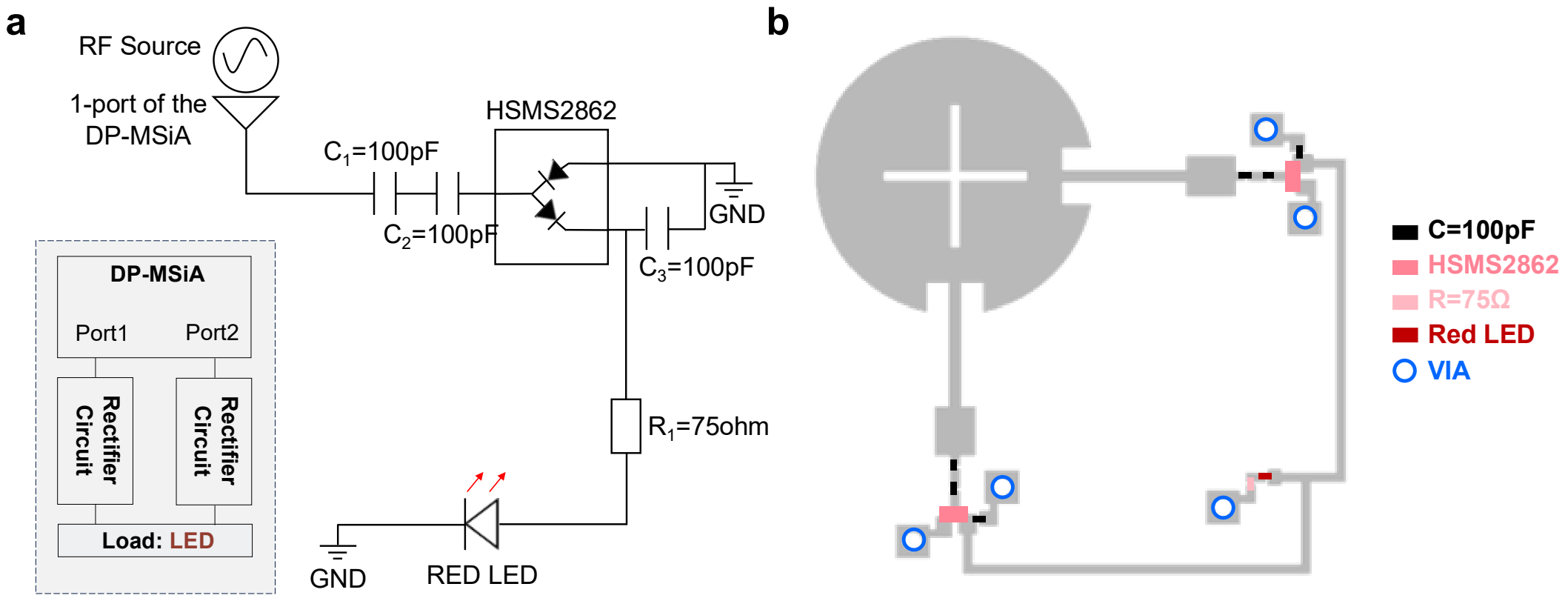

Figure S24. **(a)** Schematic configuration and **(b)** component layout for the DP-MSiA-based wireless energy-harvesting (WEH) system.

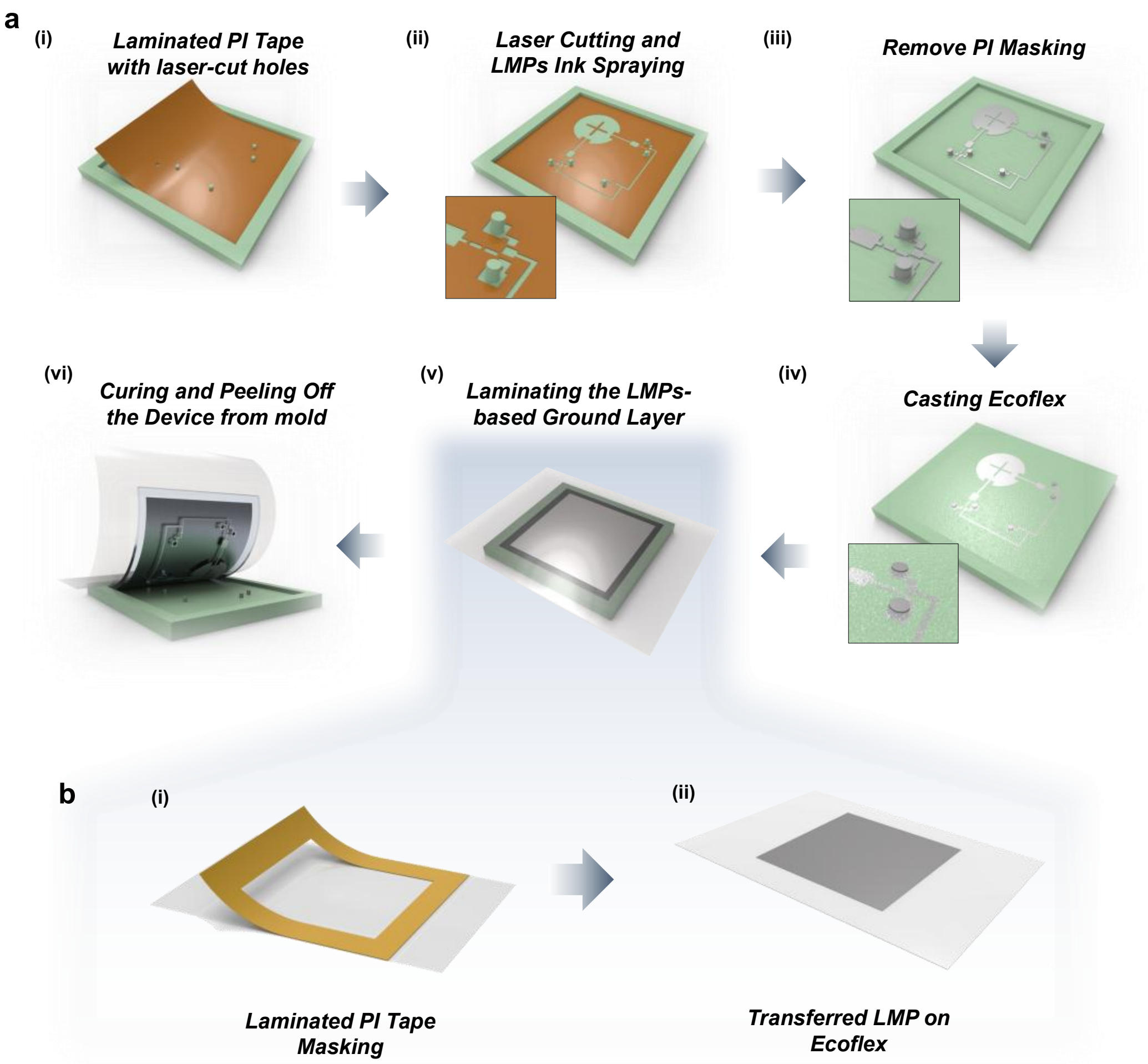

Figure S25. Schematic showing the fabrication processes of **(a)** DP-MSiA-based wireless energy-harvesting (WEH) system and **(b)** the ground layer of transferred LMPs on Ecoflex.

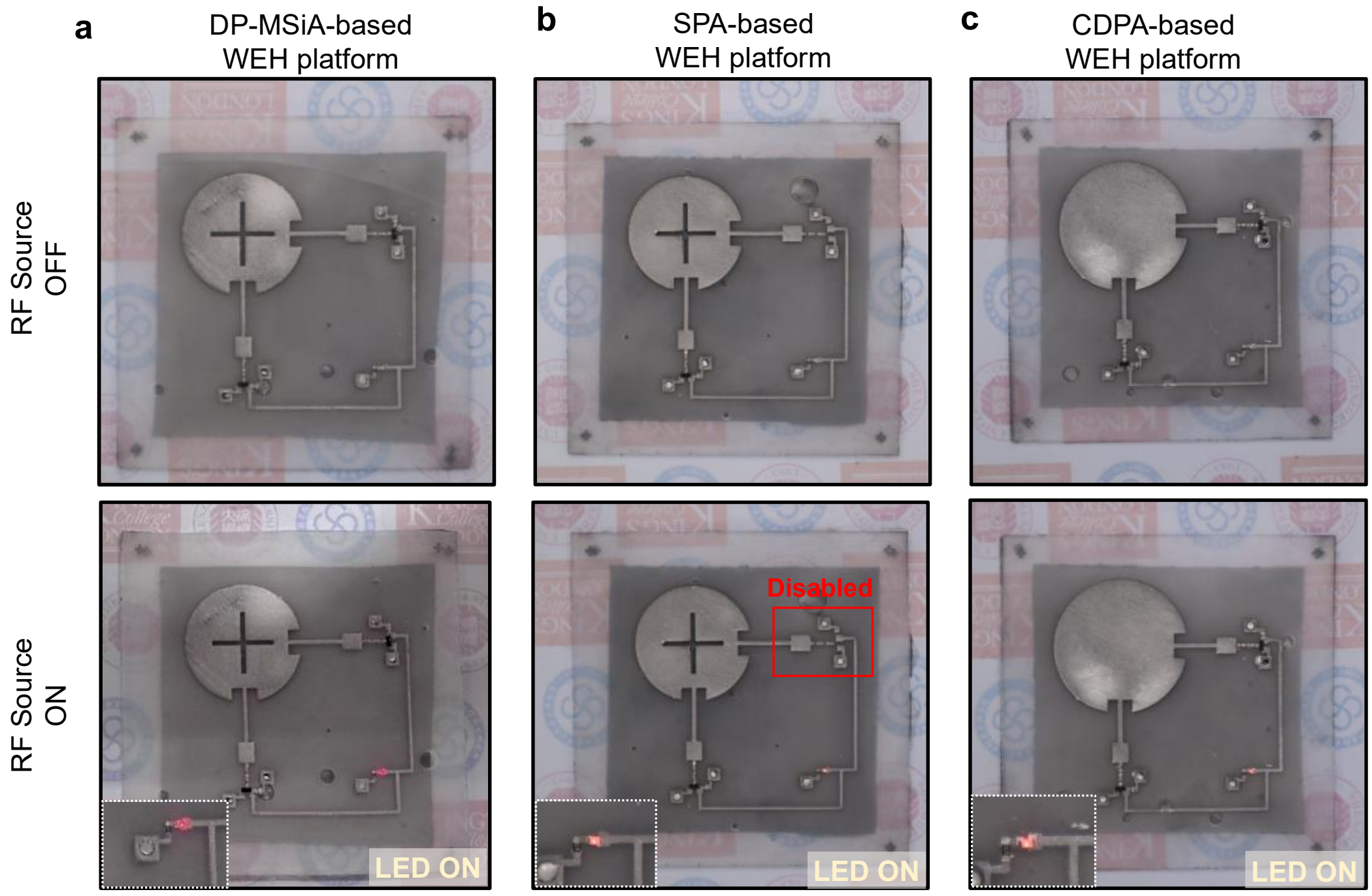

Figure S26. Photographs showing the LED status powered by **(a)** DP-MSiA-, **(b)** SPA-, and **(c)** CDPA-based rectennas with RF source turned OFF (top) and ON (bottom).

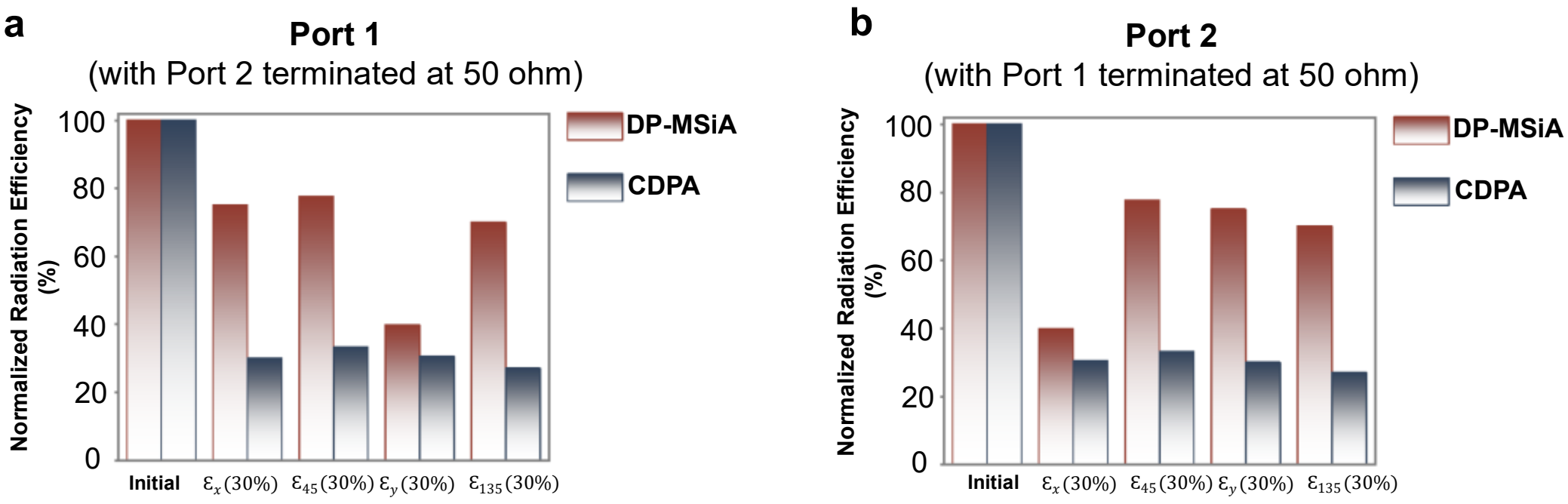

Figure S27. Simulated radiation efficiency (normalized by that before stretching) of the DP-MSiA and CDPA under 30% tensile strain along various directions: **(a)** mode excited by Port 1 with Port 2 terminated at 50 Ω, and **(b)** mode excited by Port 2 with Port 1 terminated at 50 Ω.

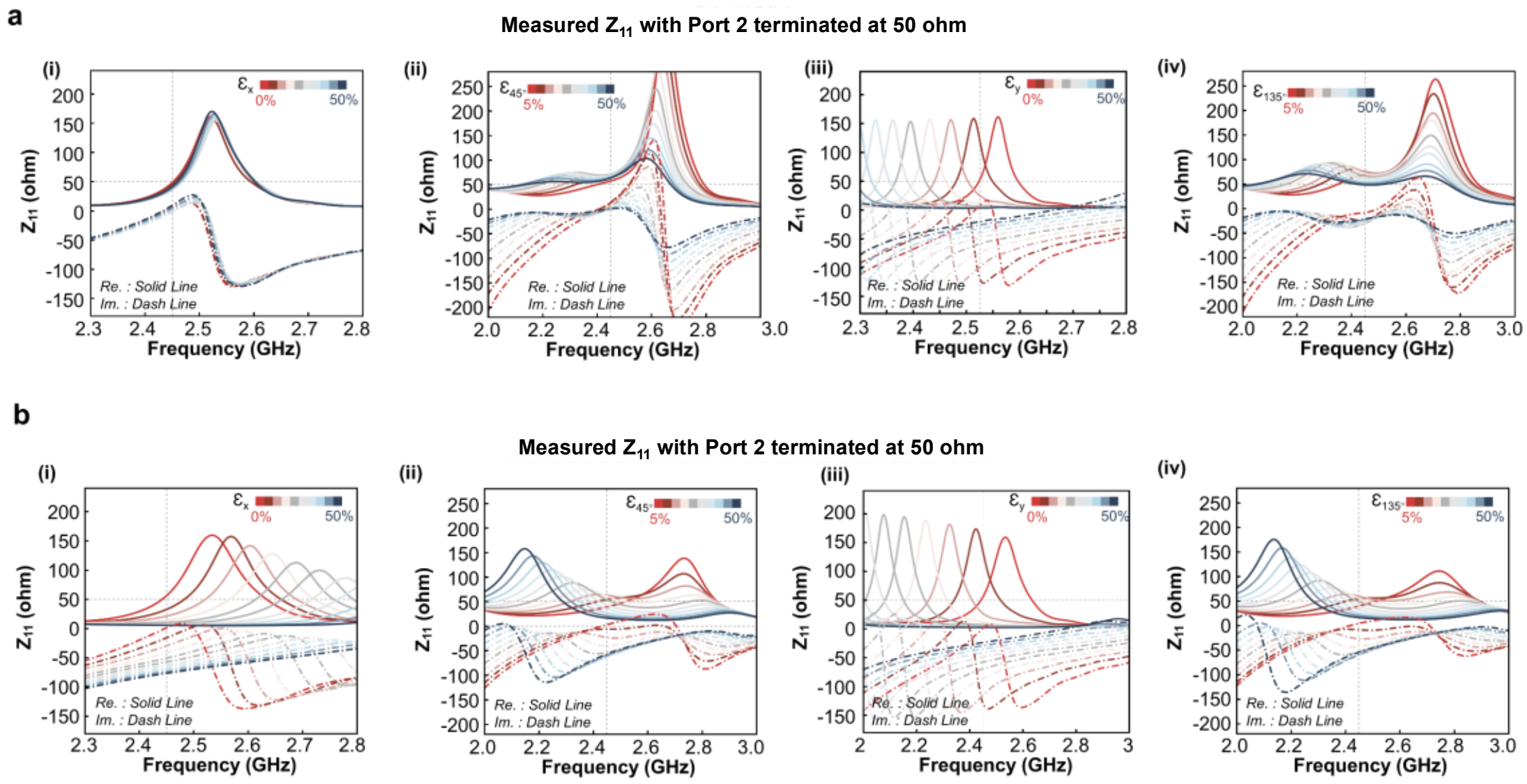

Figure S28. Simulated Z_{11} of **(a)** DP-MSiA and **(b)** CDP under stretching from 0 to 50% at a step size of 5%, along (i) x, (ii) 45°, (iii) y, and (iv) 135° directions.

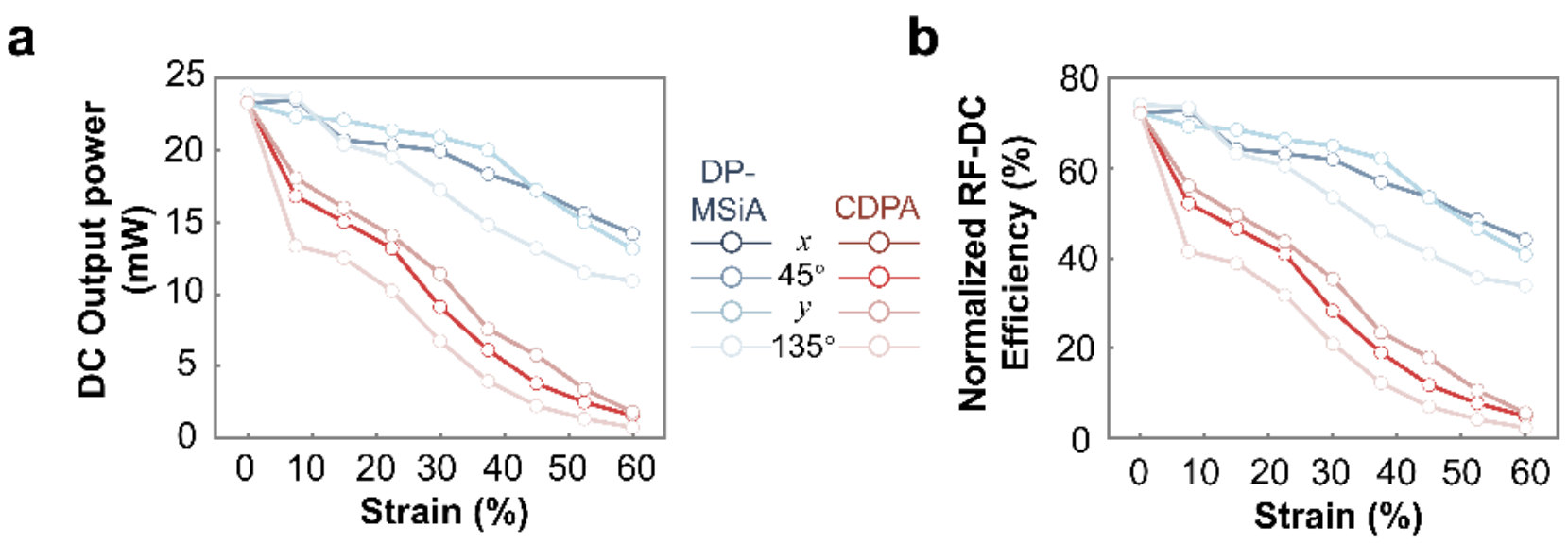

Figure S29. Quantitative WEH performance comparison between the proposed DP-MSiA and the conventional CDPA under multidirectional stretching. **(a)** DC output power measured across the 160-ohm load as a function of strain**.** **(b)** Estimated normalized RF-to-DC efficiency as a function of strain. Results are shown for four representative in-plane stretching directions (x, 45°, y, and 135°).

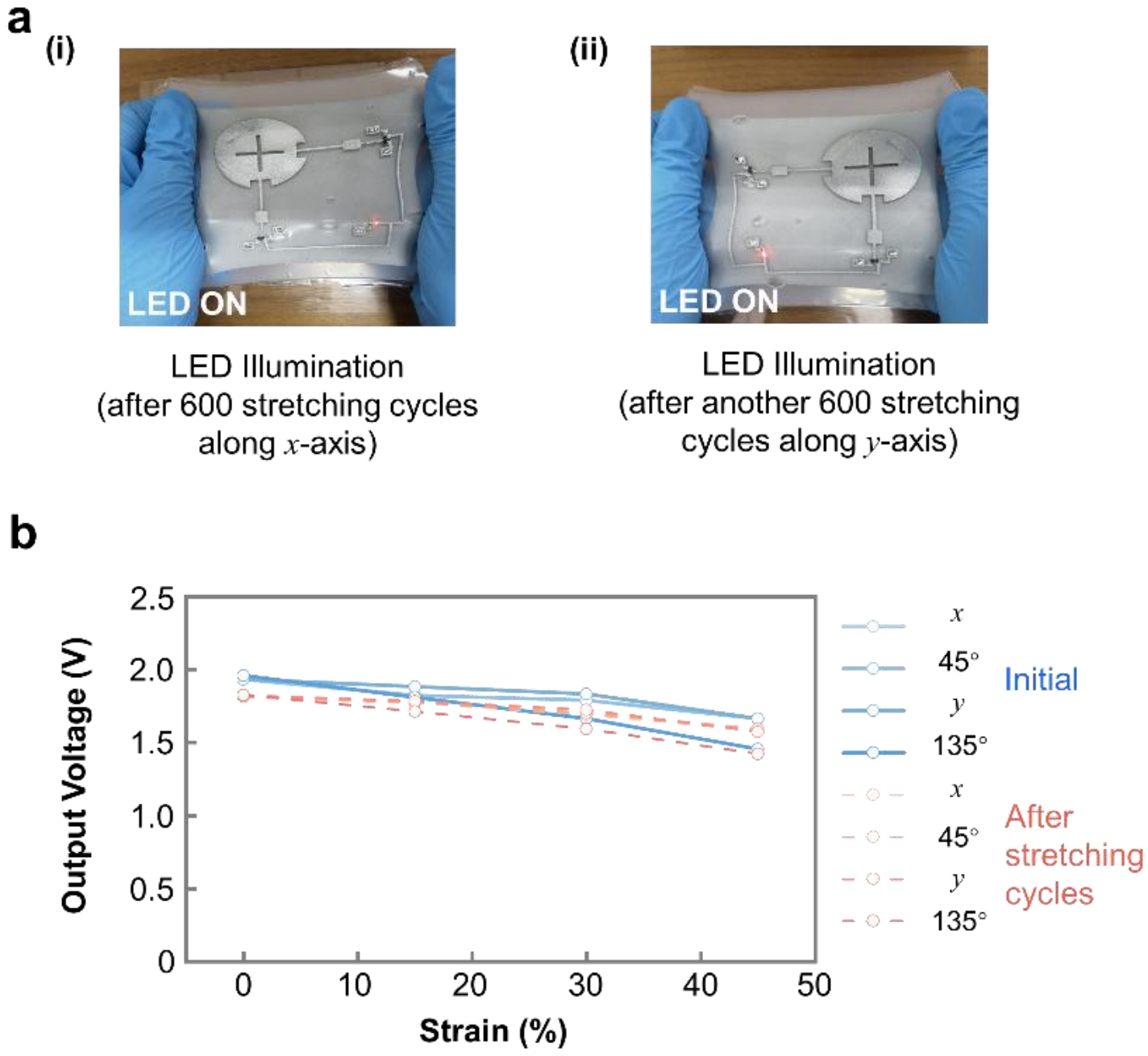

Figure S30. Cyclic stretching characterization of the DP-MSiA-based WEH system. (a) Photographs of LED illumination after cyclic stretching along the (i) x-axis and (ii) y-axis. **(b)** Output voltage versus strain measured along the x, 45°, y, and 135° directions before and after cyclic stretching.

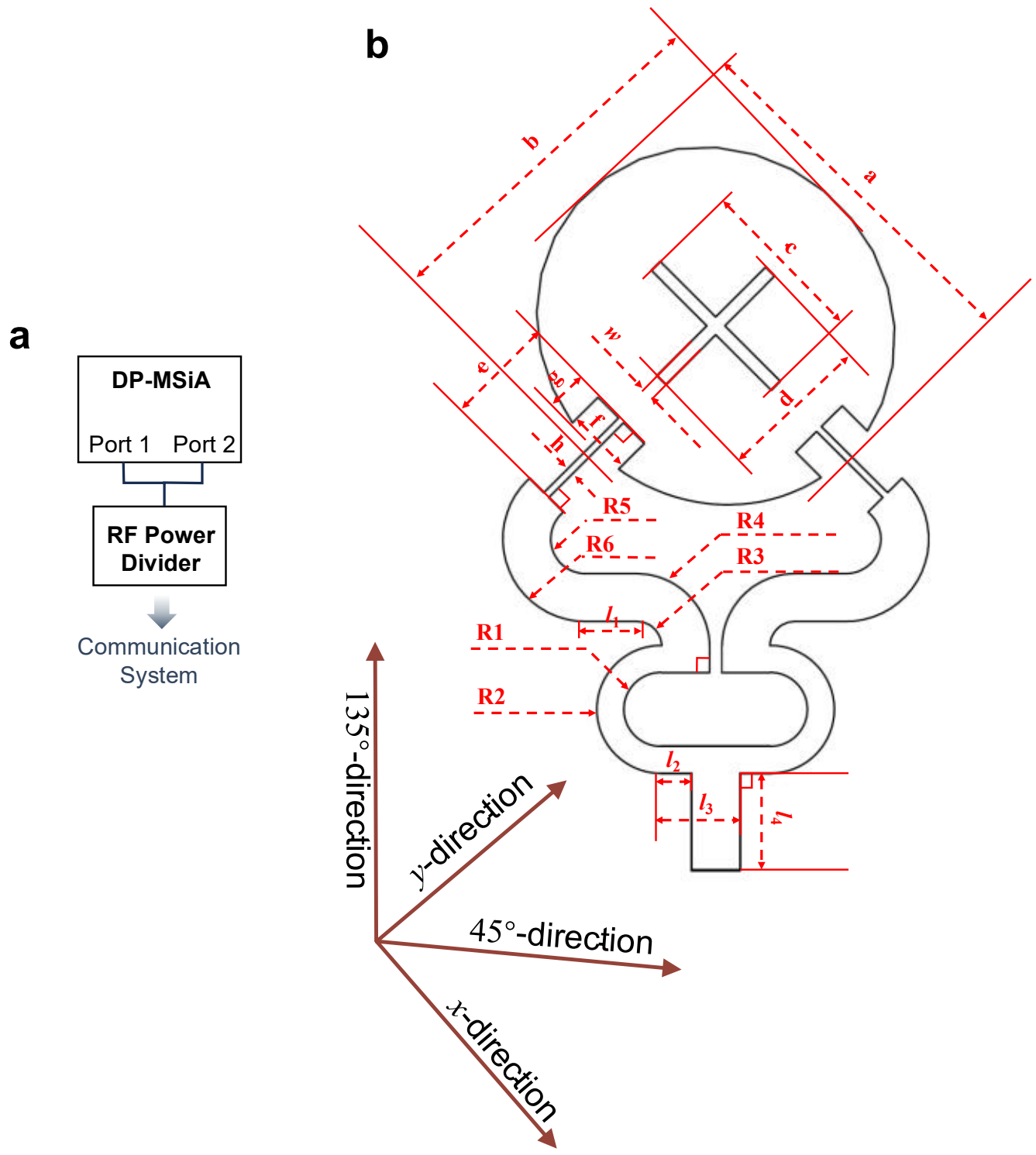

Parameter	Size (mm)
a	38.5
b	36.5
c	17.3
d	15.7
e	10.8
f	6.7
g	3.7
h	0.7
w	1.3
R1	3.8
R2	6.5
R3	2.5
R4	7.4
R5	3
R6	7.9
l_1	5.3
l_2	3.1
l_3	8.1
l_4	9.9

Figure S31. **(a)** Schematic configuration and **(b)** geometrical dimensions of the single-port, multidirectional, and strain-tolerant antenna (SP-MSiA) based on the RF power divider.

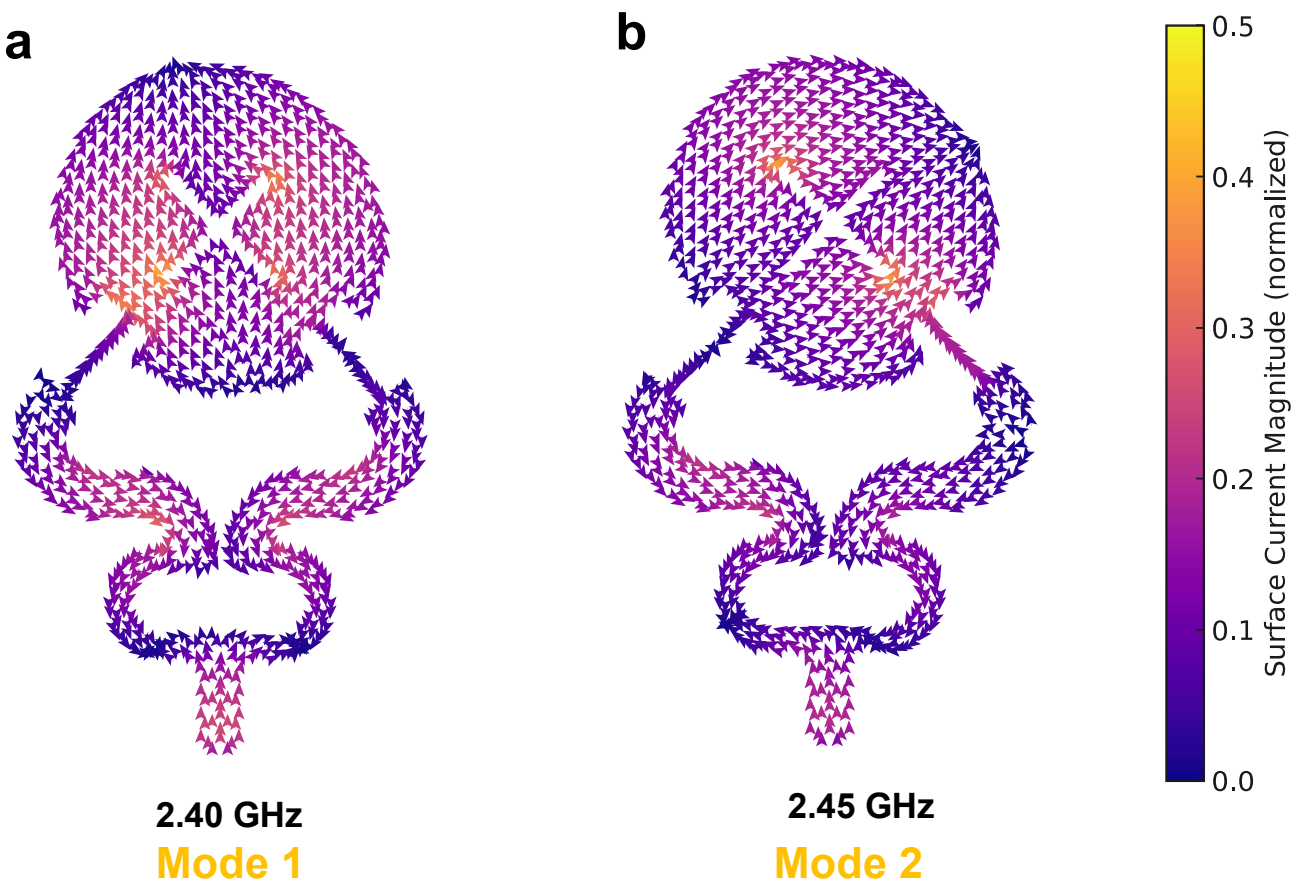

Figure S32. Simulated surface current distribution of SP-MSiA at **(a)** 2.40 GHz (Mode 1, major-axis-dominant) and **(b)** 2.45 GHz (Mode 2, minor-axis-dominant).

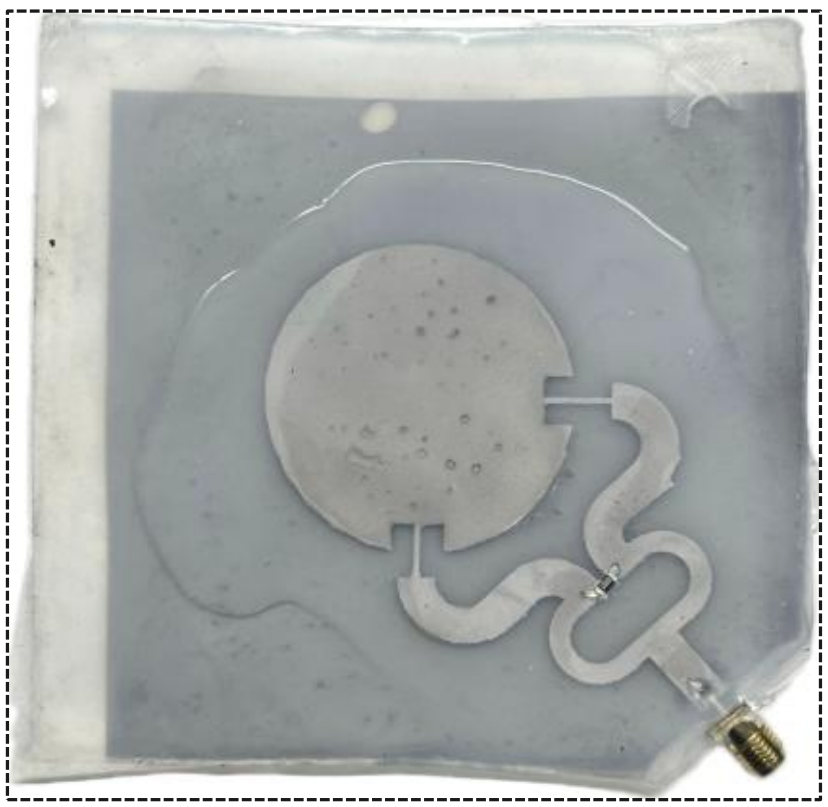

Figure S33. Photograph of a no-slot single-input antenna system with a power-divider-fed two-branch patch radiator.

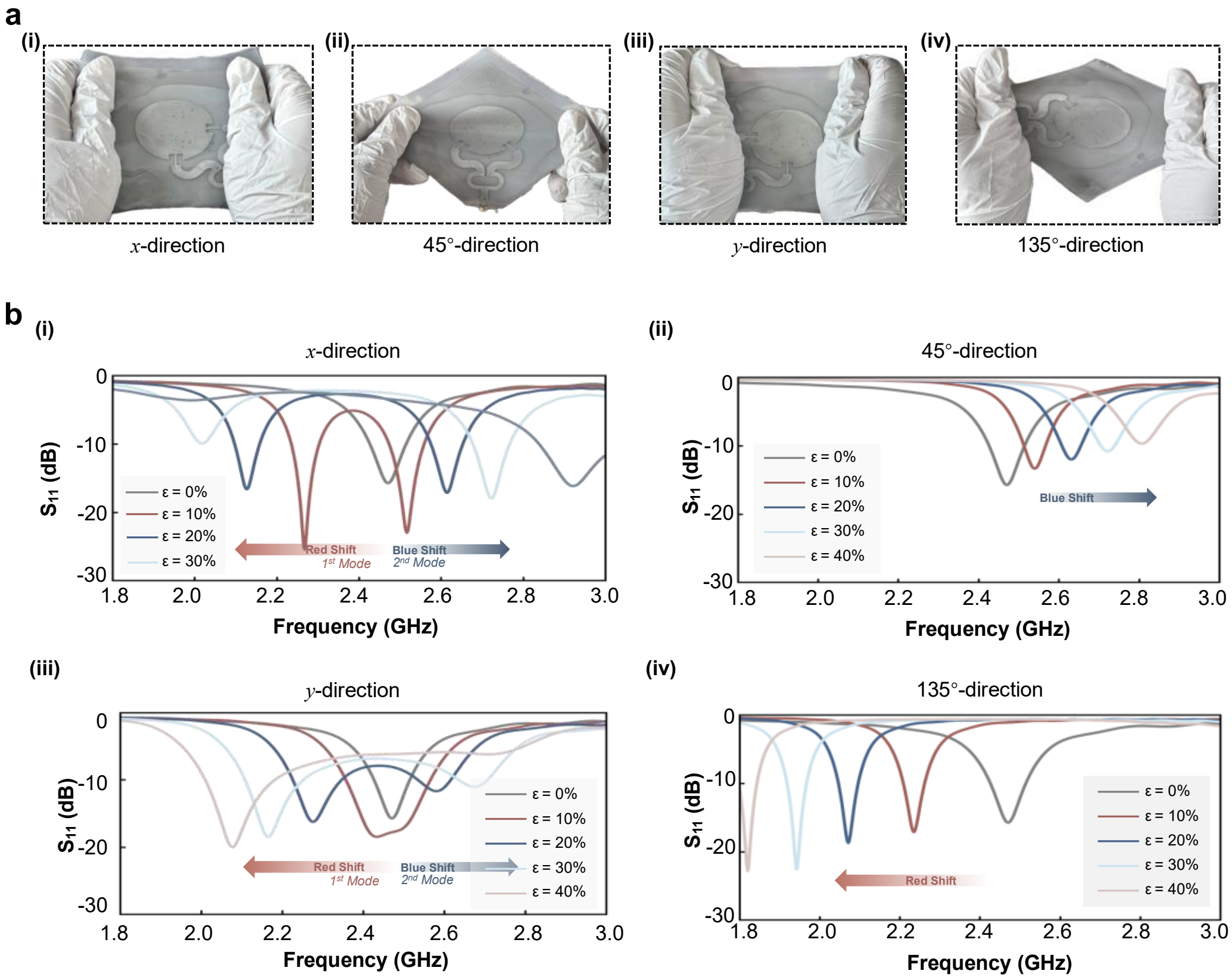

Figure S34. **(a)** Photograph and **(b)** measured S_{11} of the conventional dual-port antenna (without slot) with power divider under tensile strain of 0, 10, 20, 30, and 40% applied along (i) x, (ii) 45°, (iii) y, and (iv) 135° directions.

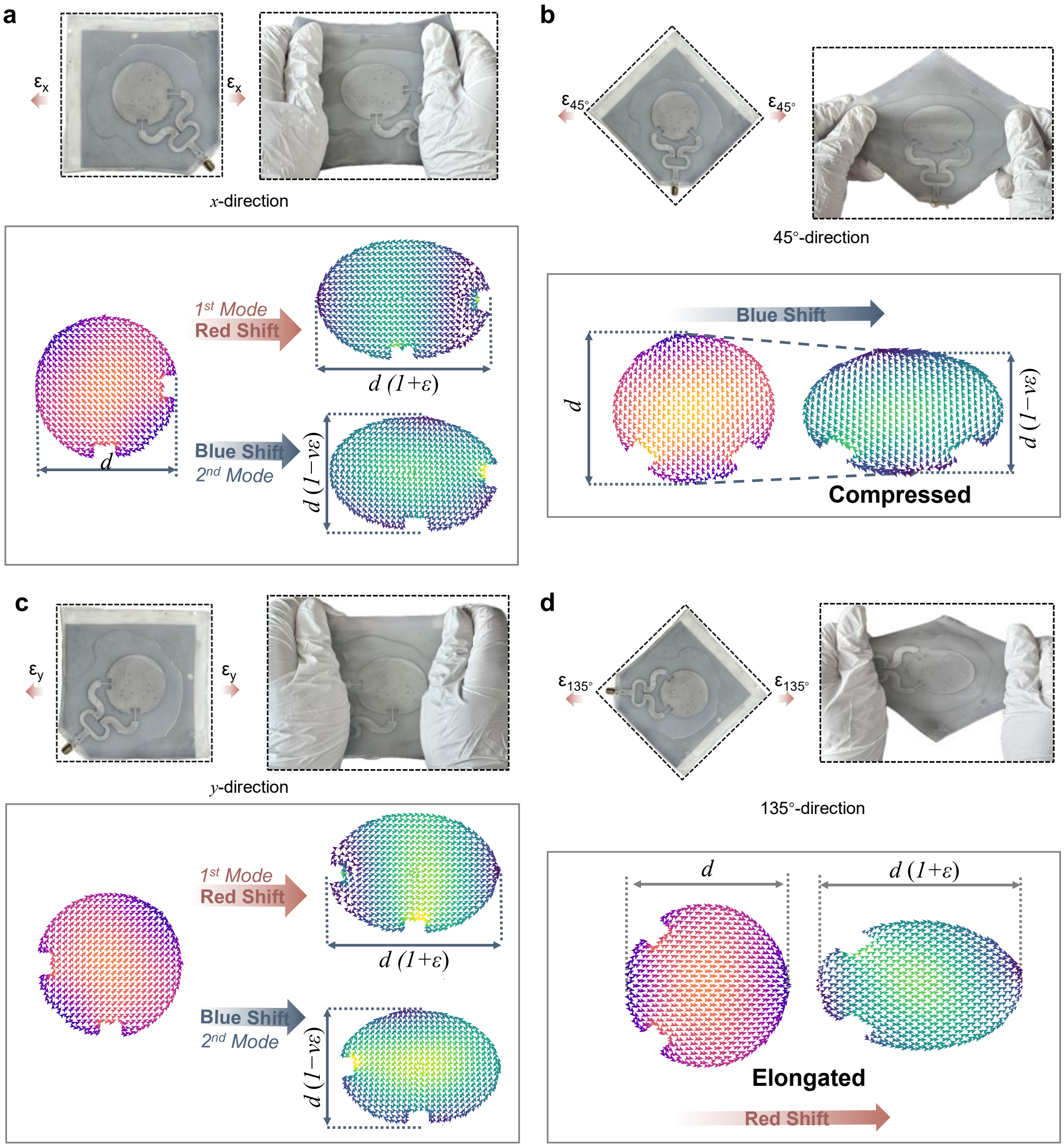

323

324 **Figure S35.** Photographs (top) and simulated surface current distributions (bottom) of the
325 conventional dual-port antenna (without slot) with power divider under tensile strain of 0, 10, 20, 30,
326 and 40% applied along **(a)** x, **(b)** 45°, **(c)** y, and **(d)** 135° directions.

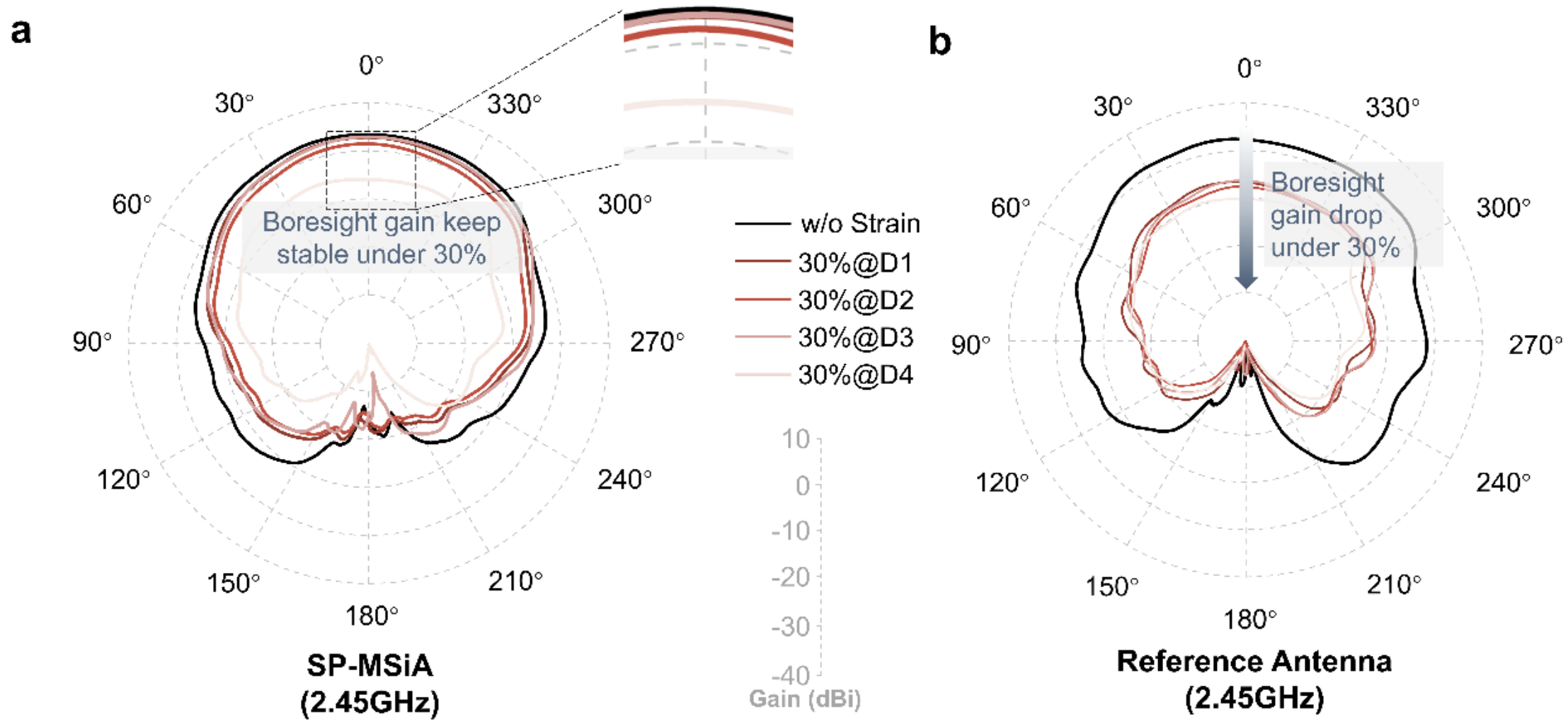

Figure S36. Radiation patterns of the **(a)** SP-MSiA and **(b)** conventional dual-port antenna (without slot) with power divider under tensile strain of 30% applied along x, 45°, y, and 135° directions.

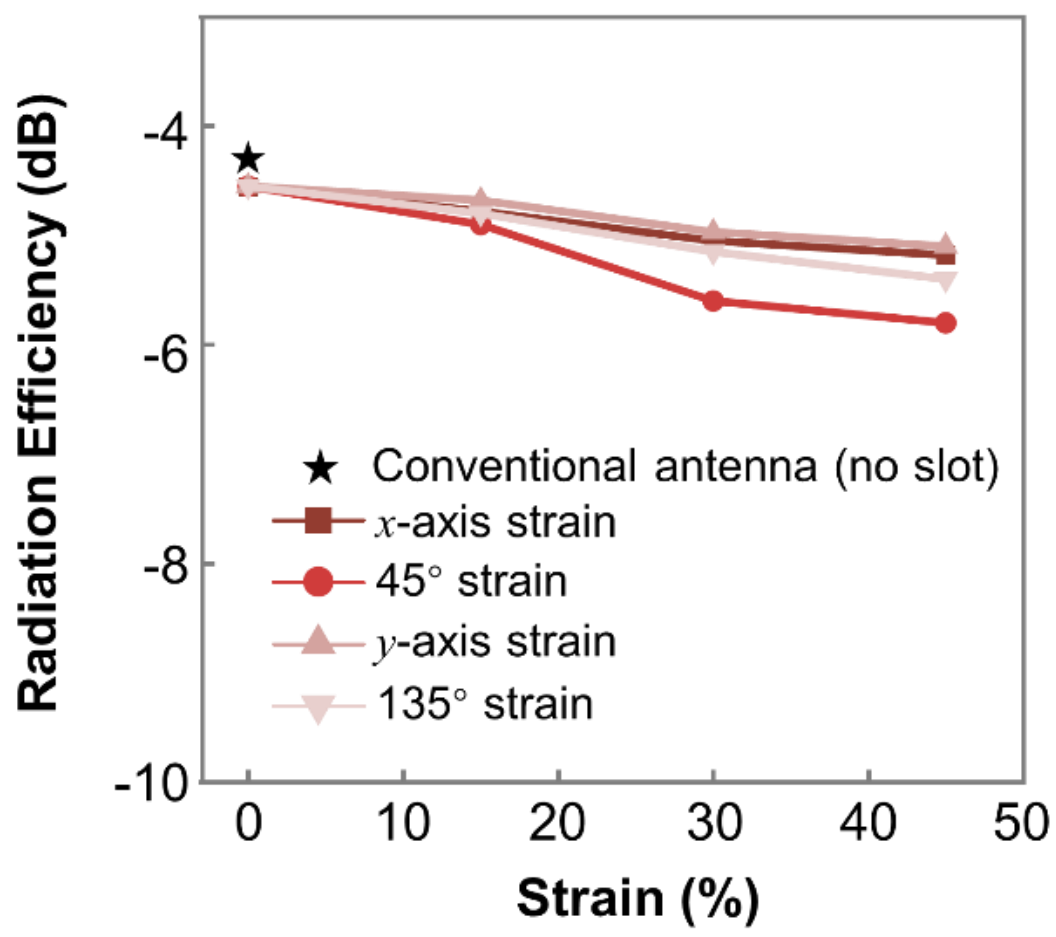

Figure S37. Absolute radiation efficiency of the SP-MSiA as a function of strain level under four representative in-plane stretching directions (x, 45°, y, and 135°).

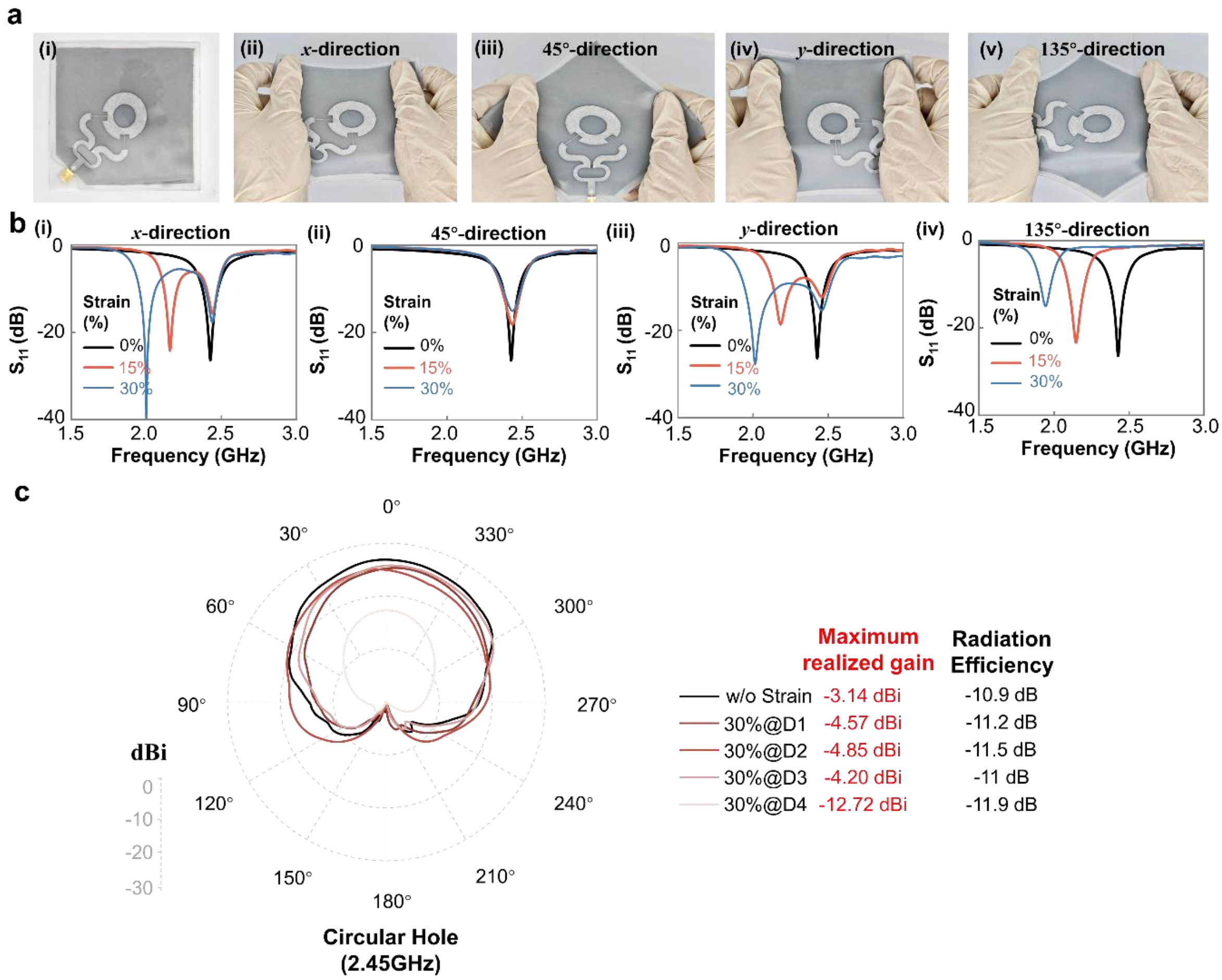

Figure S38. **(a)** Photographs of the circular-hole strain-invariant antenna (i) before and under stretching along the (ii) x, (iii) 45°, (iv) y, and (v) 135° directions. **(b)** Measured S-parameters (S_{11}) of the circular-hole strain-invariant antenna under applied strain (15% and 30%) along the (i) x, (ii) 45°, (iii) y, and (iv) 135° directions. **(c)** Measured radiation patterns and maximum realized gain of the circular-hole strain-invariant antenna before and after 30% strain applied along the x, 45°, y, and 135° directions.

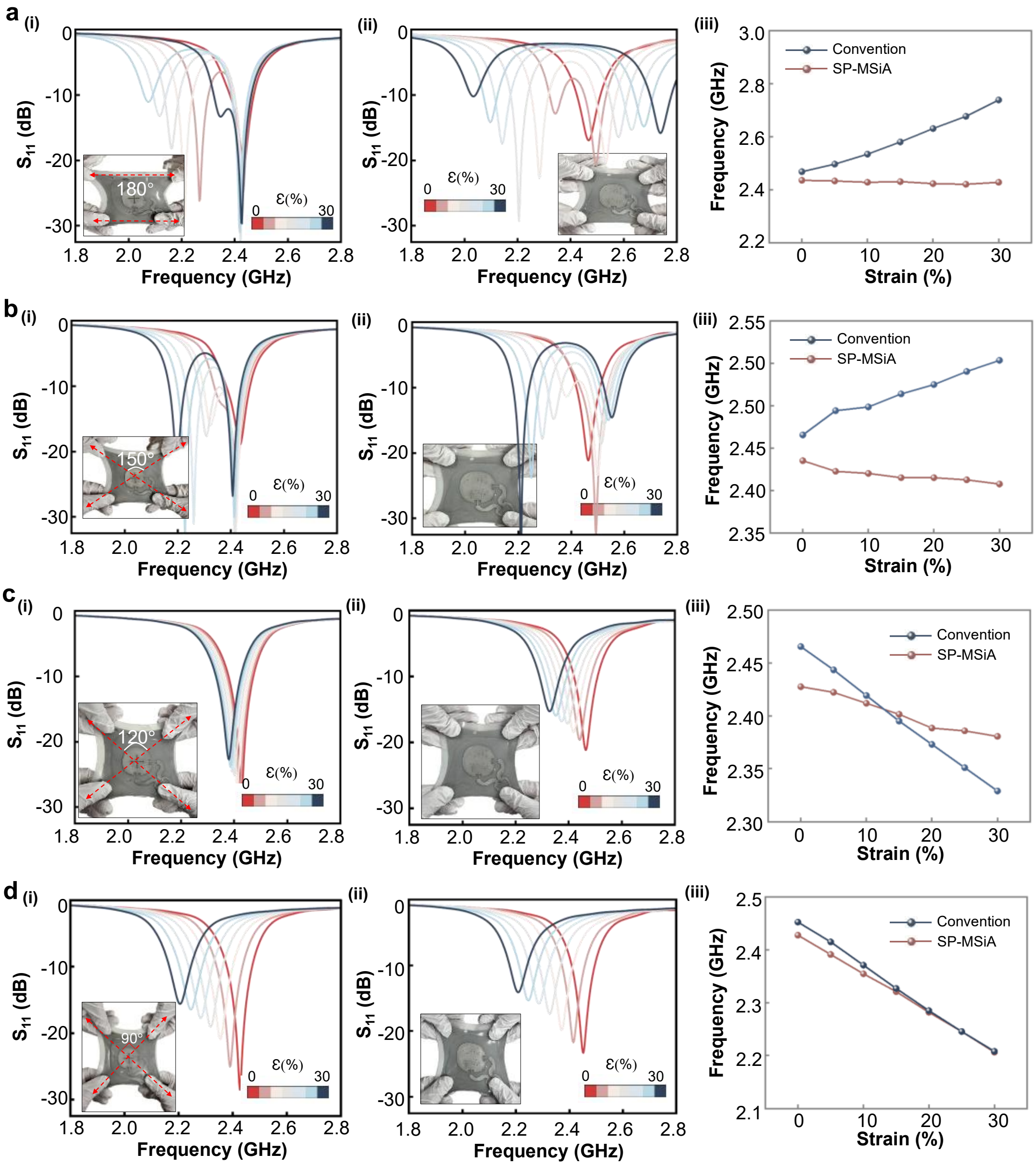

Figure S39. Measured S_{11} of the (i) SP-MSiA and (ii) reference conventional antenna under dual biaxial tensile strain of 0, 5, 10, 15, 20, 25, and 30% with four representative inter-axis angles of **(a)** 180° (parallel), **(b)** 150°, **(c)** 120°, and **(d)** 90°, along with (iii) the corresponding extracted resonance frequency. The insets show photographs of the SP-MSiA and the reference conventional antenna under biaxial strain.

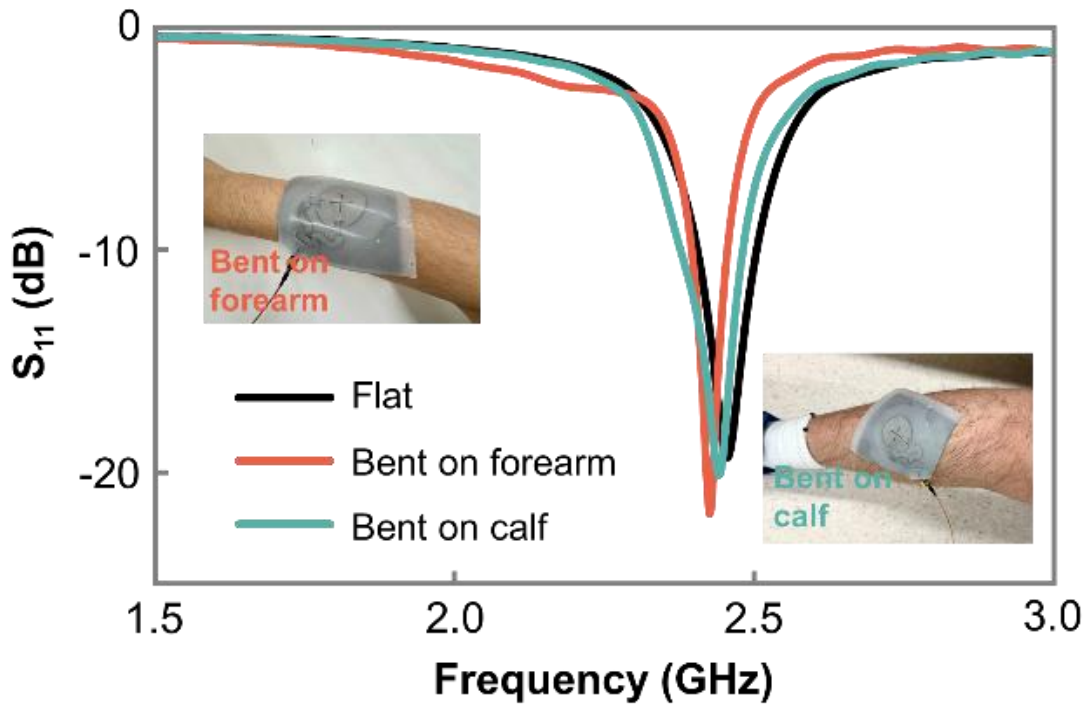

Figure S40. Measured S_{11} of the SP-MSiA in the flat state and under bending on the forearm and calf.

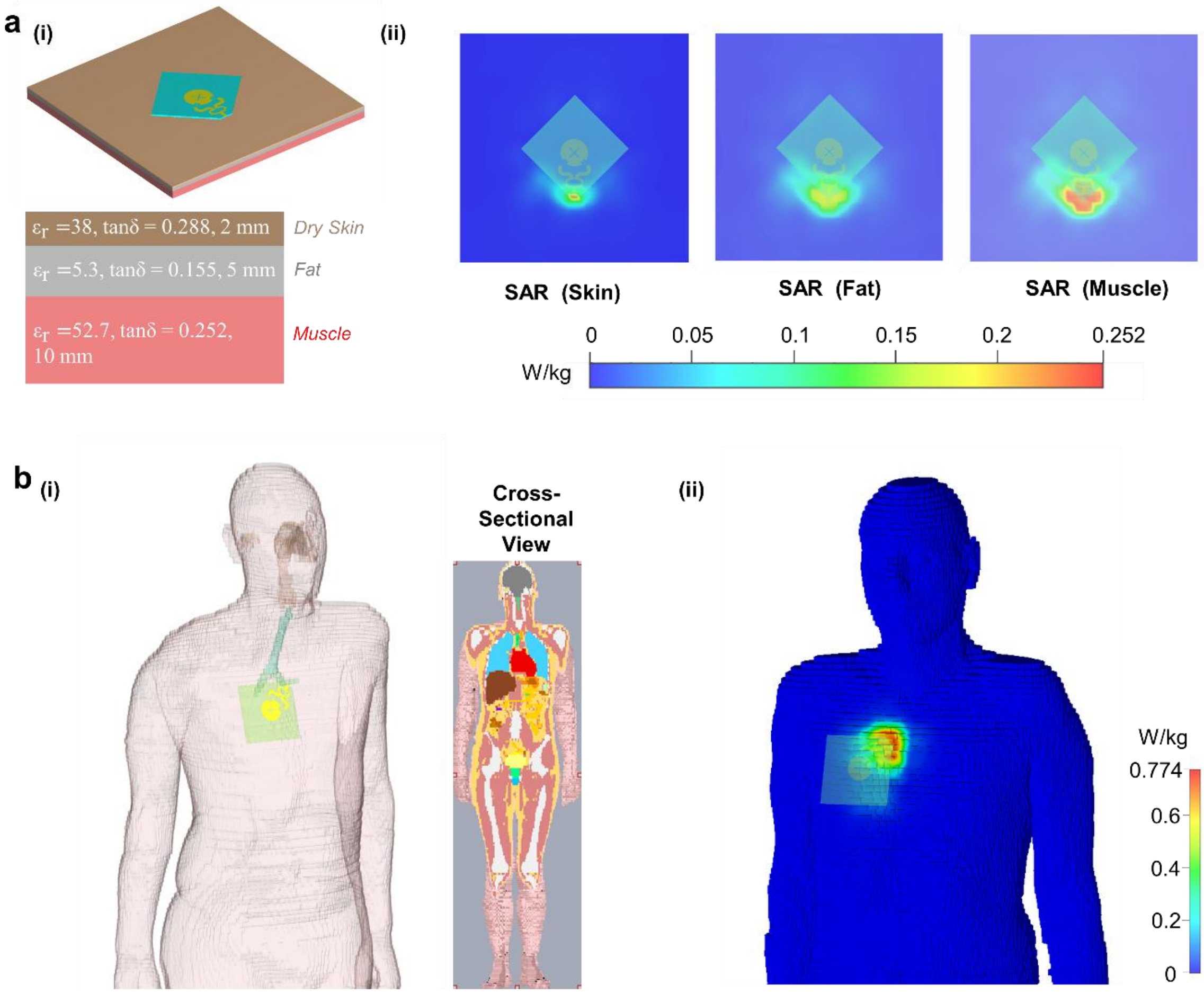

Figure S41. (i) Schematic model and (ii) simulated SAR distribution of the proposed SP-MSiA on **(a)** a simplified three-layer tissue model (consisting of dry skin, fat, and muscle) and **(b)** a realistic "Gustav" human voxel model.

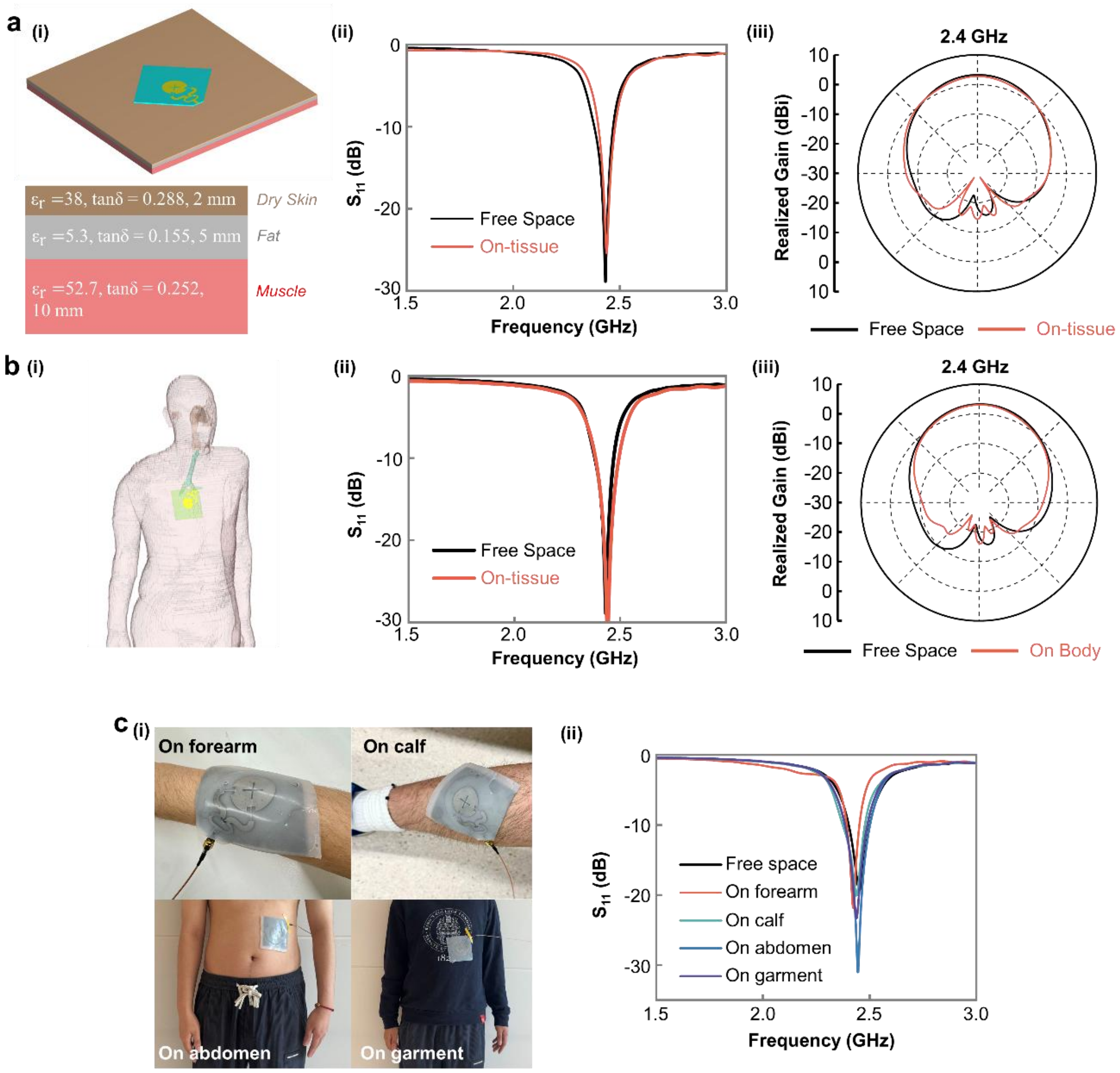

Figure S42. Simulated and measured body-loading effects of the DP-MSiA. **(a)** (i) Three-layer tissue model, simulated (ii) S_{11}, and (iii) realized gain patterns in free space and on tissue. **(b)** (i) "Gustav" human voxel model, simulated (ii) S_{11}, and (iii) realized gain patterns in free space and on body. **(c)** (i) Wearable configurations on the forearm, calf, abdomen, and garment, and (ii) corresponding measured S_{11} responses.

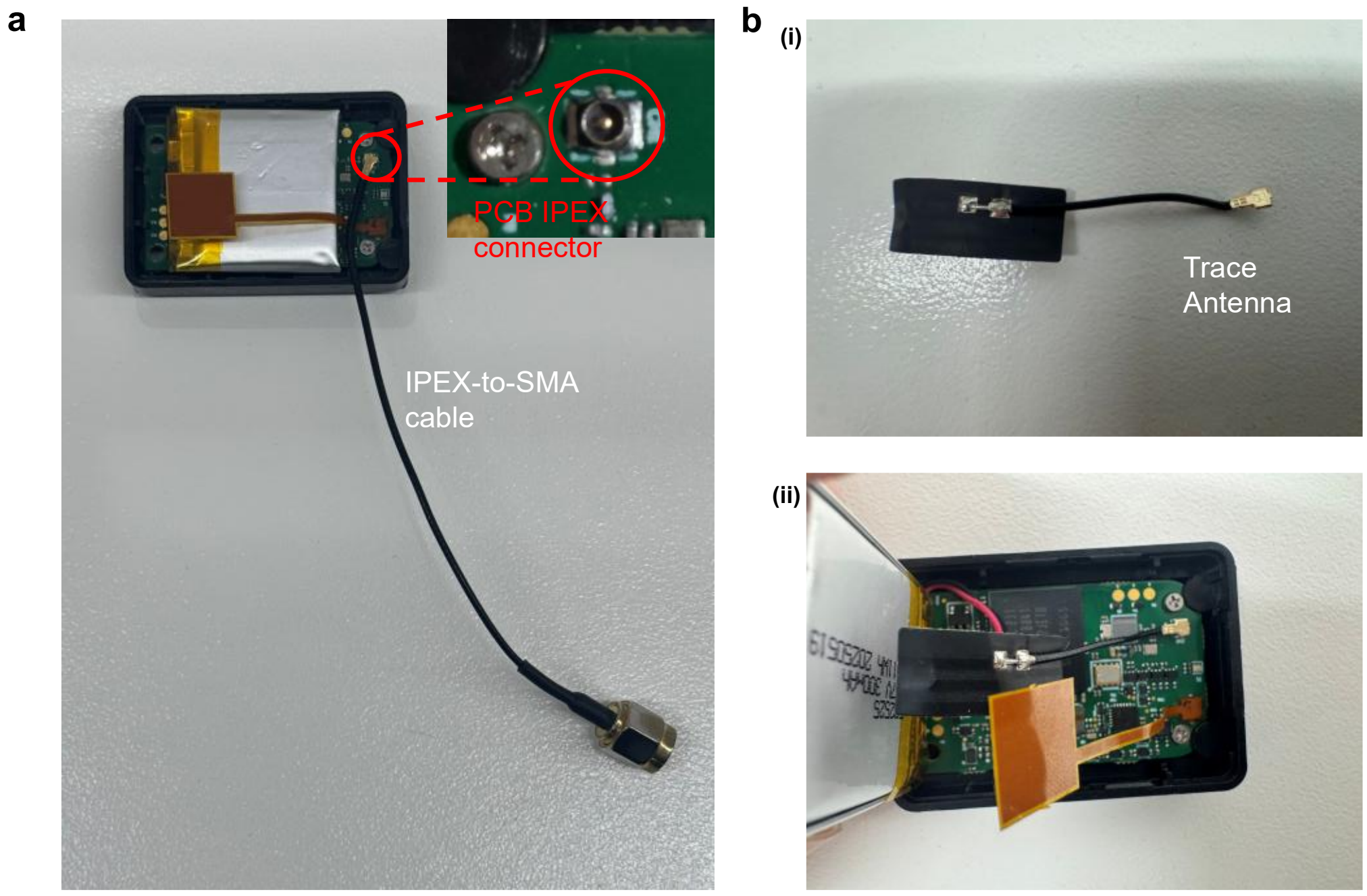

Figure S43. **(a)** Photograph of the IPEX-to-SMA cable for connecting SP-MSiA with the commercial wireless ECG monitoring system (BioBox M2). **(b)** Photograph of (i) the trace antenna and (ii) the connection with BioBox M2.

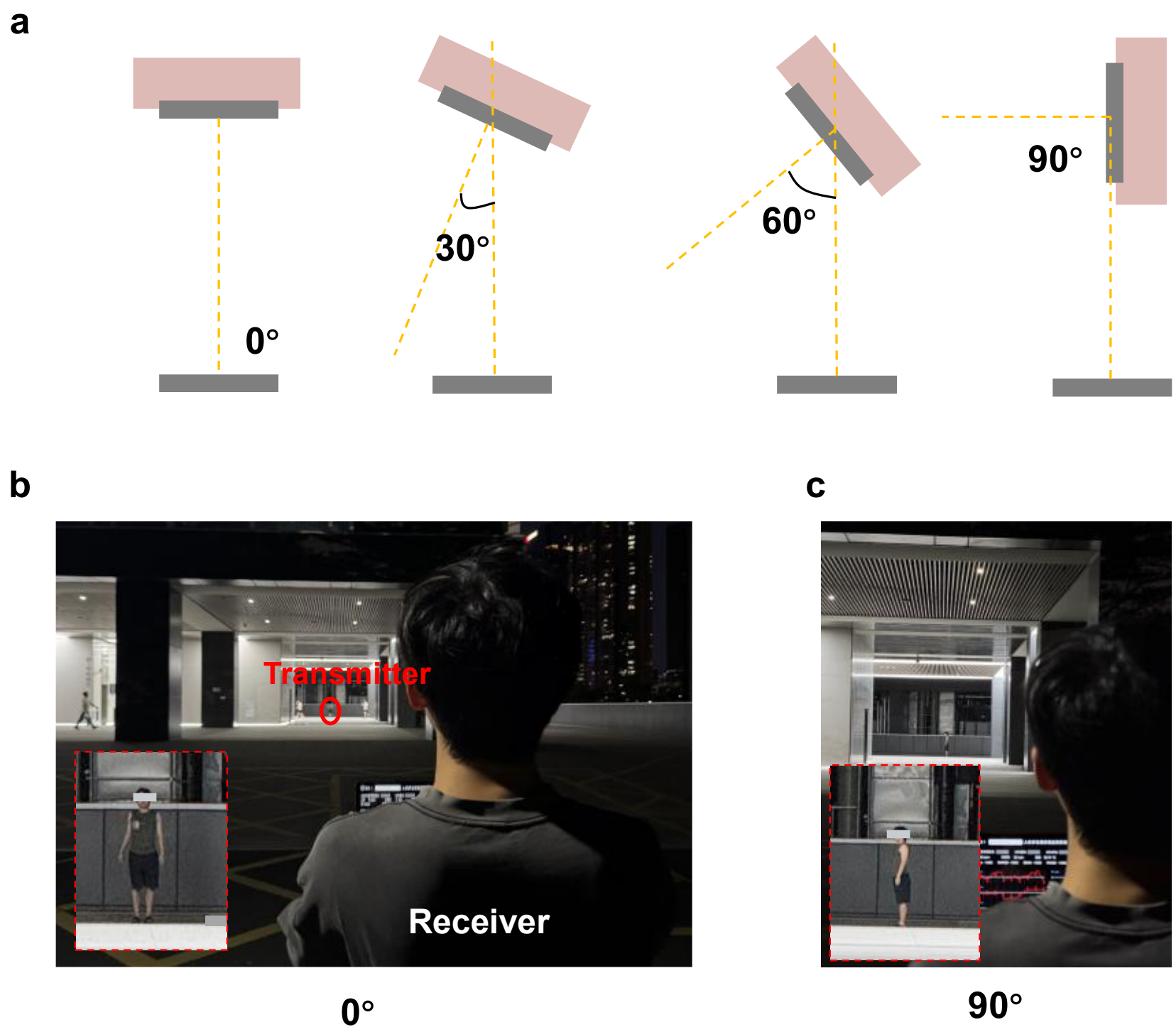

Figure S44. **(a)** Schematic showing the test setup with various off-boresight (OB) angles to measure the effective communication distance, along with the photographs under reflective conditions at OB angles of **(b)** 0° and **(c)** 90°.

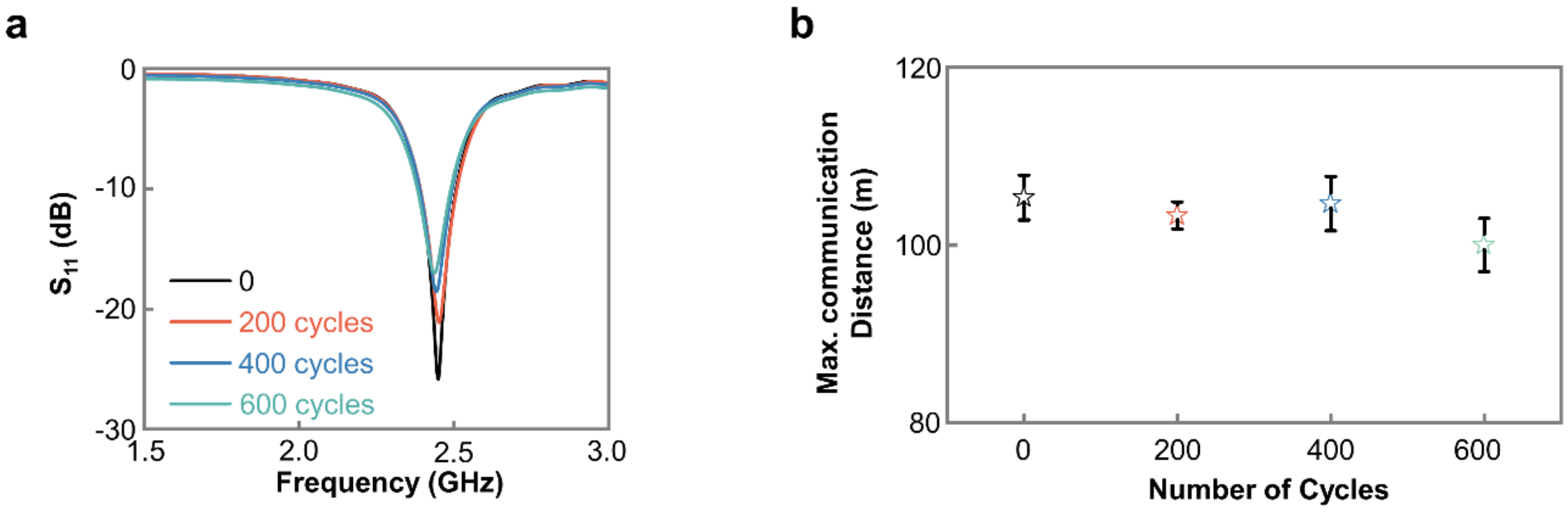

Figure S45. Cyclic stretching characterization of the SP-MSiA. **(a)** Measured S_{11} of the SP-MSiA after 200, 400, and 600 stretching cycles. **(b)** Maximum communication distance of the SP-MSiA as a function of stretching cycle number. Error bars represent the standard deviation from four repeated measurements.

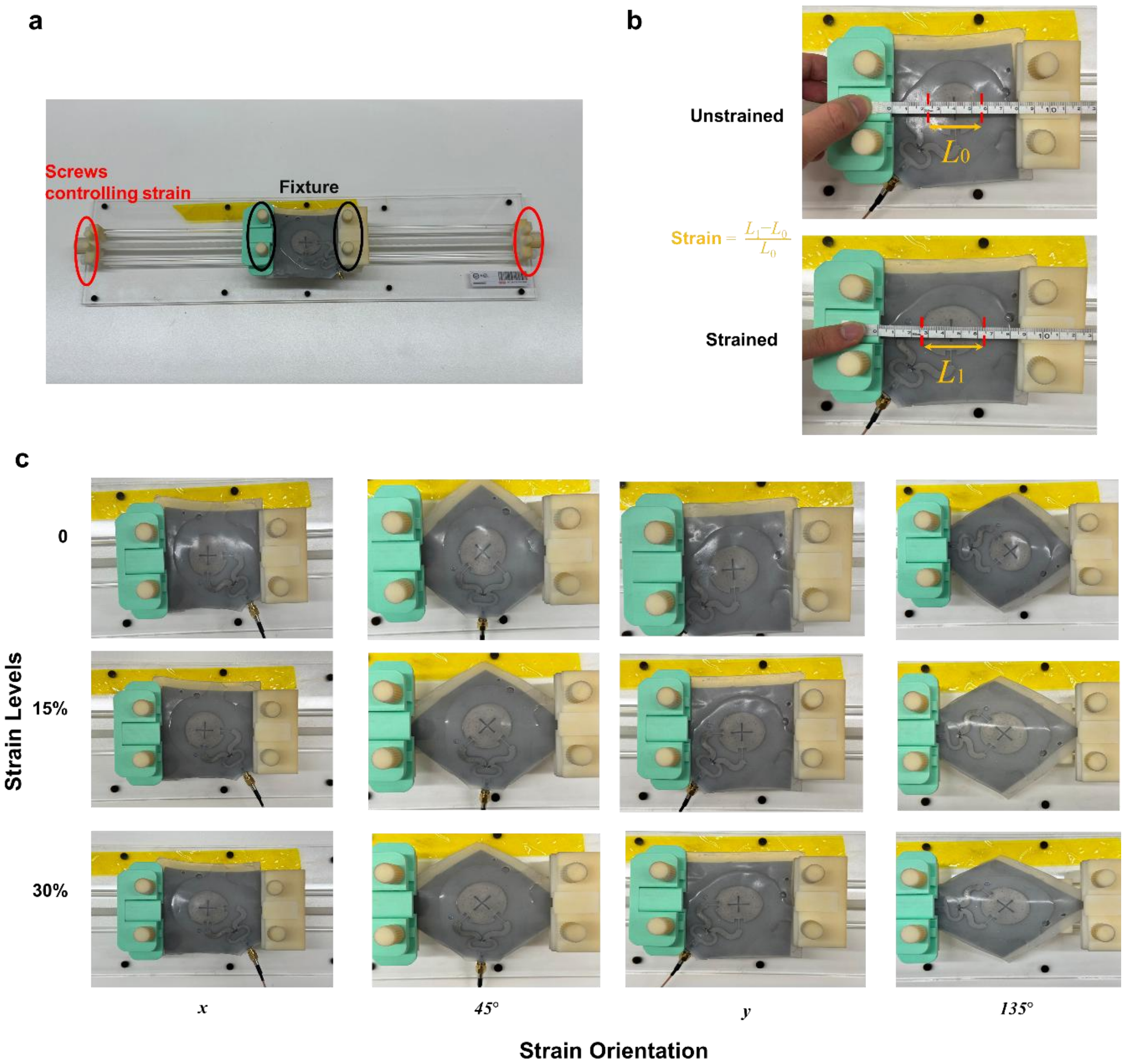

Figure S46. **(a)** Experimental setup of the custom-built nonmagnetic and nonconductive stretcher for strain-dependent antenna measurements. **(b)** Illustration of the strain calculation method, where strain is determined from the change in the characteristic patch length as $(L_1–L_0)/L_0$. **(c)** Photographs of the antenna under different loading directions (x, 45°, y, and 135°) and strain levels (0%, 15%, and 30%).

Table S1. Comparison of the reported and existing advanced stretchable antennas

Ref	Applied Strain (%)	Frequency Shift (MHz)	Resonance Frequency (GHz)	Strain Insensitive Angular Coverage	Peak Gain (dBi)	Materials	Antenna Type
[17]	20	140	2.2	0	/	LIG+Ecoflex	Meander Dipole
[18]	30	400	1.7	0	/	Copper+Solaris	Meander Dipole
[19]	30	100	2.32	0	/	Ti/Au+PDMS	Meander Patch
[20]	40	210	3.4	0	/	Silver-plated knitted textile+ Nylon/Ecoflex	Serpentine Patch
[21]	32	550	2.2	0	/	Copper+ Solaris	Serpentine Patch
[22]	120	900	1.6	0	/	EGaIn+ PDMS/Ecoflex	Dipole
[23]	80	1400	2.4	0	/	EGaIn+ Ecoflex/PDMS	Dipole
[24]	22.2	250	1.85	0	/	EGaIn+ PDMS	Dipole
[25]	30	230	2.5	0	/	Copper+PDMS	Monopole
[26]	100	400	1.5	0	/	$Ti_3C_2T_x$ MXene/ SWNT+ Latex	Dipole
[27]	50	140	1.7	0	/	Silver nanoparticles+ SEBS elastomer	Dipole
[28]	20	20	1.8	single axis	1.57 dBi	Copper+Ecoflex	3D-Arced Serpentine Microstrip Patch

[29]	30	7	2.4	two axes	-9.975 dBi	silver nanoflakes +dielectro-elastic elastomer	Serpentine Microstrip Patch
**SP-MSiA (This work)**	**40**	**12**	**2.45**	**270° (three axes)**	**3.3 dBi**	**EGaIn+Ecoflex**	**Microstrip Patch**
**DP-MSiA (This work)**	**50**	**< 40**	**2.45**	**360° (all axes)**	**3.3 dBi**	**EGaIn+Ecoflex**	**Microstrip Patch**

Table S2. Resonance frequency shifts of the DP-MSiA under different levels and orientations of uniaxial strain.

Orientation / Strain	*x* direction	45° direction	*y* direction	135° direction
15%	12 MHz	-30 MHz	4 MHz	7 MHz
30%	8 MHz	-25 MHz	8 MHz	30 MHz
45%	4 MHz	-25 MHz	-12 MHz	40 MHz

References:

[1] Y. Lo, D. Solomon, and W. Richards, Theory and experiment on microstrip antennas[J]. **IEEE transactions on Antennas and Propagation**, 27(2): 137-145 (2003).

[2] K. Carver, J. Mink, Microstrip antenna technology[J], **IEEE transactions on antennas and propagation**, 29(1): 2-24 (1981).

[3] D.M. Pozar, Microstrip antennas[J], **IEEE transactions on antennas and propagation**, 80(1): 79-91 (1980).

[4] E. Hammerstad, O. Jensen, Accurate models for microstrip computer-aided design[J], **1980 IEEE MTT-S Int. Microwave Symp. Digest**, 407–409 (1980).

[5] R. Garg, Microstrip antenna design handbook[M], **Artech House** (2001).

[6] M. Kirschning, R.H. Jansen, N.H.L. Koster, Accurate model for open-end effect of microstrip lines[J], ***Electronics Letters***, 17(3): 123–125 (1981).

[7] C.A. Balanis, Antenna theory: analysis and design[M], **John Wiley & Sons** (2016).

[8] L. Shen, et al., Resonant frequency of a circular disc, printed-circuit antenna[J], **IEEE Transactions on Antennas and Propagation**, 25(4): 595–596 (1977).

[9] D.M. Pozar, D.H. Schaubert (Eds.), The analysis and design of microstrip antennas and arrays[M], **New Jersey, USA** (1995).

[10] D.M. Pozar, A review of aperture-coupled microstrip antennas: history, operation, development, and applications[M], **Univ. of Massachusetts at Amherst** (1996).

[11] R.N. Simons, Coplanar waveguide circuits, components, and systems[M], **Wiley** (2004).

[12] R.W. Ogden, Non-linear elastic deformations[M], **Courier Corporation** (1997).

[13] G.A. Holzapfel, Nonlinear solid mechanics: a continuum approach for engineering science[M], **Wiley** (2002).

[14] J.E. Marsden, T.J.R. Hughes, Mathematical foundations of elasticity[M], **Courier Corporation** (1994).

[15] L.R.G. Treloar, The physics of rubber elasticity[M], **Oxford Univ. Press** (1975).

[16] D.R. Darby, Z. Cai, and C.R. Mason et al., Modulus and adhesion of Sylgard 184, Solaris, and Ecoflex 00-30 silicone elastomers with varied mixing ratios[J], **Journal of Applied Polymer Science**, 139(25): e52412 (2022).

[17] J. Zhu, Z. Hu, and C. Song, et al., Stretchable wideband dipole antennas and rectennas for RF energy harvesting[J], **Materials Today Physics**, 18: 100377 (2021).

[18] T. Chang, Y. Tanable, and C.C. Wojcik et al., A general strategy for stretchable microwave antenna systems using serpentine mesh layouts[J], **Advanced Functional Materials**, 27(46): 1703059 (2017).

[19] S.I. Park, D.S. Brenner, and G. Shin et al., Soft, stretchable, fully implantable miniaturized optoelectronic systems for wireless optogenetics[J], **Nature Biotechnology**, 33(12): 1280–1286 (2015).

[20] F. Nikbakhtnasrabadi, H.E. Matbouly, and M. Ntagios et al., Textile-based stretchable microstrip antenna with intrinsic strain sensing[J], **ACS Applied Electronic Materials**, 3(5): 2233–2246 (2021).

[21] Y. Kim, A. Basir, and R. Herbert et al., Soft materials, stretchable mechanics, and optimized designs for body-wearable compliant antennas[J], **ACS Applied Materials & Interfaces**, 12(2): 3059–3067 (2019).

[22] M. Kubo, X. Li, and C. Kim et al., Stretchable microfluidic radiofrequency antennas[J], **Advanced Materials**, 22: 1–10 (2010).

[23] B. Yao, X. Xu, and Q. Zhang et al., Highly stretchable and mechanically tunable antennas based on three-dimensional liquid metal network[J], **Materials Letters**, 270: 127727 (2020).

[24] J. So, J. Thelen, and A. Qusba et al., Reversibly deformable and mechanically tunable fluidic antennas[J], **Advanced Functional Materials**, 19(22): 3632–3637 (2009).

[25] A.M. Hussain, F.A. Ghaffar, and S. Park et al., Metal/polymer based stretchable antenna for constant frequency far-field communication in wearable electronics[J], **Advanced Functional Materials**, 25(42): 6565–6575 (2015).

[26] Y. Li, X. Tian, and S. Gao et al., Reversible crumpling of 2D titanium carbide (MXene) nanocoatings for stretchable electromagnetic shielding and wearable wireless communication[J], **Advanced Functional Materials**, 30(5): 1907451 (2020).

[27] Z. Liu, F. Wan, and L. Mou et al., A general approach for buckled bulk composites by combined biaxial stretch and layer-by-layer deposition and their electrical and electromagnetic applications[J], **Advanced Electronic Materials**, 5(4): 1800817 (2019).

[28] J. Zhu, S. Zhang, and N. Yi et al., Strain-insensitive hierarchically structured stretchable microstrip antennas for robust wireless communication[J], **Nano-Micro Letters**, 13(1): 108 (2021).

[29] S.H. Kim, A. Basir, and R. Avila et al., Strain-invariant stretchable radio-frequency electronics[J], **Nature**, 629: 1047–1054 (2024)
