# Supplementary material for: Multidirectional strain-insensitive stretchable RF electronics
Source: Nat Commun. 2026 Jun 27;17:8041. doi: 10.1038/s41467-026-74900-5 (PMC13454596; doi:10.1038/s41467-026-74900-5)
Supplement: Supplementary file 2 [file 41467_2026_74900_MOESM2_ESM.pdf]

nature portfolio

# Peer Review File

# Multidirectional strain-insensitive stretchable RF electronics

Corresponding Author: Professor Huanyu Cheng

**This file contains all reviewer reports in order by version, followed by all author rebuttals in order by version.**

Version 0:

Reviewer comments:

Reviewer #1

(Remarks to the Author)
This manuscript proposes a stretchable planar antenna concept intended to mitigate resonance detuning under multidirectional in-plane strain by combining symmetry-based slot loading with a dual-port architecture, supported by characteristic-mode analysis and experimental S-parameter characterization, and further demonstrated in (i) a dual-port rectenna for wireless energy harvesting and (ii) a Wilkinson-divider-fed single-port configuration for BLE-based wearable ECG transmission. The idea is timely and the system-level demonstrations are relevant to wearable communications; however, several critical points remain insufficiently supported—most notably the lack of a clearly documented strain/VNA measurement setup and strain quantification method, inconsistent/unclear on-body (or phantom) loading conditions across WEH vs BLE validations, missing experimental verification of the Wilkinson feed network under strain, limited durability evidence for rigid interfaces/components under large (especially diagonal) deformation, and ambiguity in the quantitative headline resonance-shift claim—therefore I recommend major revision.

Major comments:

1. The manuscript states that strain-dependent S-parameters were measured using a “custom-built nonmagnetic and nonconductive stretcher” and a VNA with SOLT calibration to the SMA reference plane, but it does not provide a photo or schematic of the measurement setup nor a reproducible protocol describing how the applied strain (e.g., 10%, 20%, 30%, 45%) was defined and verified. Because the central claim of the work relies on direction-resolved resonance stability under controlled deformation, the authors should clearly describe the strain quantification procedure (e.g., gauge length, clamp spacing, marker-based length change, or another metric), the method used to align and maintain the strain axis (x, 45°, y, 135°), and the associated measurement uncertainty. In addition, since the work targets wearable antennas, it should be clarified whether these measurements were conducted in free space, on cloth, or on-body/phantom conditions, as body loading can significantly affect resonance and impedance matching.

2. The validation conditions used for the WEH platform (DP-MSiA) and the BLE communication platform (SP-MSiA) are not clearly aligned. The WEH demonstration is described as a far-field rectenna experiment with a commercial transmitting patch antenna placed 1 m away, but it is unclear whether the receiving rectenna was measured in free space or under on-body/garment conditions. In contrast, the BLE communication demonstration explicitly attaches the antenna to a smart T-shirt and evaluates link performance under garment stretching. Since the manuscript emphasizes wearable applications, the authors should clarify the measurement conditions used for both DP-MSiA and SP-MSiA and ideally provide a comparison between free-space and on-body (or phantom) antenna performance under representative strain levels to ensure that the two demonstrations are evaluated under consistent and realistic wearable scenarios.

3. The wireless energy-harvesting validation relies primarily on LED illumination and the output voltage measured across a 160-Ω load, together with a “failure strain” definition; while these demonstrations are visually compelling, they do not provide a reproducible, quantitative rectenna/WPT assessment. Please report the transmit conditions in a form that enables replication and fair comparison (e.g., PA output power or delivered power to the Tx antenna, Tx antenna gain, polarization/alignment, and the resulting EIRP or estimated incident power density/field strength at 1 m), because the current description (“3 dBm signal generator + amplifier”) is insufficient to infer the RF exposure at the receiver. In addition, please include RF-to-DC conversion performance (e.g., DC output power and/or PCE versus incident power or received power) and its variation versus strain/direction; at minimum, converting the reported Vout across 160 Ω to Pout (=$V^2$/R) and defining a voltage/power-based “failure” criterion (rather than an LED-threshold-dependent criterion) would substantially improve rigor and comparability.

4. The SP-MSiA configuration critically relies on the Wilkinson power divider, and the manuscript claims that the PD maintains nearly unchanged S-parameters under stretching. However, this claim is not sufficiently supported by experimental data. Because the Wilkinson divider is a quarter-wave network whose electrical properties may change under mechanical deformation, it is important to experimentally validate its strain tolerance. The authors should therefore provide measured S-parameters (e.g., S11, S21, and isolation) of the power divider under representative strain levels and directions to substantiate the claim that the feed network remains electrically stable under deformation.

5. The wearable system incorporates rigid interfaces and discrete components (e.g., SMA connectors, the isolation resistor in the Wilkinson divider, and multiple components in the rectifier circuit) attached using conductive silver paste. Under large deformation, particularly diagonal stretching (e.g., 45°), these rigid–soft interfaces may experience stress concentration and potential mechanical failure. However, the manuscript does not present any durability or cyclic stretching tests. The authors should include at least a basic fatigue test (e.g., hundreds or thousands of cycles at representative strain levels) and report the resulting changes in S11, WEH output voltage, or communication performance to demonstrate that the antenna and associated circuitry maintain stable operation under repeated mechanical deformation.

6. In the "real-world applications" demonstration (Fig. 7), the manuscript labels the hand-raising motion as approximately $\varepsilon y \approx 30\%$ and the chest-opening motion as approximately $\varepsilon x \approx 30\%$. However, the manuscript does not explain how these strain values were determined during human motion. Without a quantitative measurement method (e.g., marker-based distance change, simple gauge-length estimation, or video-based tracking), it is difficult to verify the stated strain levels. The authors should either provide a quantitative method used to estimate these strain values or soften the claim by describing these motions as qualitative large deformations with approximate strain estimates.

7. The Abstract states that the DP-MSiA exhibits a resonance shift of ≤20 MHz at 2.45 GHz under up to 45% strain across multiple in-plane directions. However, other sections of the manuscript describe the strain-invariant resonance behavior using percentage-based values (e.g., approximately 1.8%), which may exceed 20 MHz depending on how the resonance is defined. The authors should clarify the method used to extract the resonance frequency and provide a consistent definition across the manuscript. A summary table or plot showing the extracted resonance shift versus strain and direction would help ensure that the headline claim is fully supported by the presented data.

Minor comments:

1. In the Supplementary Information, Fig. S28 refers to a "two-port patch antenna with the power divider," which may be confusing because a power-divider-fed antenna effectively operates as a single-input system even if the radiator has two feeds. The authors should clarify the terminology to distinguish between the radiator feed configuration and the overall system input configuration.

2. The manuscript and Supplementary Information appear to use the terms "SP-MSiA" and "IP-MSiA" interchangeably in several locations. To avoid confusion for readers, the authors should ensure that the terminology is consistent throughout the manuscript and that each acronym is clearly defined.

3. Several figures would benefit from improved visual clarity, particularly in terms of color contrast, line thickness, and consistency between legend labels and plotted curves. Improving these graphical elements would make the results easier to interpret and better highlight the key comparisons presented in the manuscript.

4. The description of prior work in the Introduction appears to have inconsistencies in the explanations of References 16 and 17. The current text suggests that the roles or characteristics of these references may have been interchanged or inaccurately summarized. The authors should carefully re-check the descriptions of Refs. 16 and 17 and revise the corresponding sentences to ensure that each reference is accurately represented.

Reviewer #2

(Remarks to the Author)
This manuscript reports a multidirectional strain-insensitive stretchable RF antenna system based on a symmetry-guided slot design and dual-port excitation strategy. By leveraging a characteristic-mode-based mechano-electromagnetic framework, the authors achieve near-constant resonance under large in-plane tensile strains across various orientations. The proposed DP-MSiA maintains a frequency shift of ≤20 MHz at 2.45 GHz under up to 45% strain, and is further integrated into wireless energy harvesting and on-body communication systems. The antenna demonstrates stable power transfer and ECG data transmission during body motions for wearable RF electronics. Here are a few concerns listed below.

1. The study claims "multidirectional" or "360° strain-insensitive" behavior. Does this strictly apply to uniaxial strain only, or to all deformation states including biaxial stretching?

2. The slot-enabled compensation appears optimized at a specific strain level (e.g., 30%). Is the strain-invariant condition theoretically valid across the entire strain range (0–45%), or only near the design point?

3. The study emphasizes preserved radiation performance. Could the authors provide absolute radiation efficiency values under representative strain levels to quantitatively support this claim?

4. Is the dual-port architecture fundamentally required for multidirectional strain insensitivity, or could a properly designed single-feed antenna achieve comparable robustness?

5. The improved communication stability is demonstrated experimentally. Could the authors clarify whether this improvement mainly results from reduced frequency detuning, or radiation-pattern stability?

Reviewer #3

(Remarks to the Author)
This manuscript presents a DP-MSiA and SP-MSiA designed to minimize the frequency shift and maximize the radiation performance under multidirectional mechanical in-plane strain. The authors comprehensively analyzed the effect of antenna geometry on the signal performance under the mechanical deformation in multi-directions, such as the presence of slots, the number of slots, slot geometry and antenna shapes. Additionally, wireless communication and ECG monitoring were demonstrated as proof-of-concept, showing the outstanding performance compared to the conventional antenna. While the overall originality and experimental validations are outstanding, additional discussions are needed before publication.
1. This antenna is intended for wearable devices mounted onto the human body. The authors should show the effect of human body tissue on the antenna's performance even though there is a ground layer.
2. Since the antenna operates in RF, specific absorption rate (SAR) should be studied to evaluate human tissue safety.
3. The authors should clearly show the microstructure of LMP layer with liquid metal particle size.
4. The authors assume the electrical conductivity of the patch and ground layers as σ = 3.6×106 S/m which is for bulk EGaIn, not for particles. Therefore, a proper conductivity value must be considered in their simulations. Plus, please provide the unit "S/m" on the manuscript.
5. Although liquid metal and Ecoflex are naturally very soft and stretchable materials, the mechanical reliability under cyclic loading should be presented to guarantee the long term use.
6. Considering the signal weakness at 135 deg, people may still think a circular hole rather than the cross-slot is more beneficial even though the overall efficiency is lower. The authors should clearly show the performance comparison between the circular hole and cross-slot in figure 5f.
7. The authors assume that the device is mechanically deformed only in-plane. However, the device is subject to bending and bi-axial stretching when it is located on the textile or human body. The effect of those mechanical deformations on the antenna performance would further improve the completeness of the manuscript.
8. In Fig. 2f (i), slot number 8 is invisible.

Version 1:

Reviewer comments:

Reviewer #1

(Remarks to the Author)
The authors have thoroughly addressed the reviewers' concerns through substantial revisions and additional experimental validations. The revised manuscript now provides clear strain-measurement methodology, body-loading and SAR analysis, cyclic durability evaluation, quantitative WEH characterization, and experimental validation of the Wilkinson power divider under deformation. The technical novelty and experimental completeness are strong, and the manuscript is now suitable for publication in its present form.

Reviewer #2

(Remarks to the Author)
The authors have addressed all the concerns.

Reviewer #3

(Remarks to the Author)
All of my concerns have been addressed.

**Open Access** This Peer Review File is licensed under a Creative Commons Attribution 4.0 International License, which permits use, sharing, adaptation, distribution and reproduction in any medium or format, as long as you give appropriate credit to the original author(s) and the source, provide a link to the Creative Commons license, and indicate if changes were made.
In cases where reviewers are anonymous, credit should be given to 'Anonymous Referee' and the source.
The images or other third party material in this Peer Review File are included in the article's Creative Commons license, unless indicated otherwise in a credit line to the material. If material is not included in the article's Creative Commons license and your intended use is not permitted by statutory regulation or exceeds the permitted use, you will need to obtain permission directly from the copyright holder.
To view a copy of this license, visit https://creativecommons.org/licenses/by/4.0/

***<u>Reviewer 1's comments and authors' responses</u>***

*This manuscript proposes a stretchable planar antenna concept intended to mitigate resonance detuning under multidirectional in-plane strain by combining symmetry-based slot loading with a dual-port architecture, supported by characteristic-mode analysis and experimental S-parameter characterization, and further demonstrated in (i) a dual-port rectenna for wireless energy harvesting and (ii) a Wilkinson-divider-fed single-port configuration for BLE-based wearable ECG transmission. The idea is timely and the system-level demonstrations are relevant to wearable communications; however, several critical points remain insufficiently supported—most notably the lack of a clearly documented strain/VNA measurement setup and strain quantification method, inconsistent/unclear on-body (or phantom) loading conditions across WEH vs BLE validations, missing experimental verification of the Wilkinson feed network under strain, limited durability evidence for rigid interfaces/components under large (especially diagonal) deformation, and ambiguity in the quantitative headline resonance-shift claim—therefore I recommend major revision.*

**Our response:**

Thank you for your thoughtful and constructive comments. We acknowledge the concerns you have raised regarding the experimental setup, validation consistency, and clarity of key claims. These issues are important, and we have carefully revised the manuscript to address them. In the following, we provide a point-by-point response and describe how each of these concerns has been resolved.

*Major comments:*

*1. The manuscript states that strain-dependent S-parameters were measured using a "custom-built nonmagnetic and nonconductive stretcher" and a VNA with SOLT calibration to the SMA reference plane, but it does not provide a photo or schematic of the measurement setup nor a reproducible protocol describing how the applied strain (e.g., 10%, 20%, 30%, 45%) was defined and verified. Because the central claim of the work relies on direction-resolved resonance stability under controlled deformation, the authors should clearly describe the strain quantification procedure (e.g., gauge length, clamp spacing, marker-based length change, or another metric), the method used to align and maintain the strain axis (x, 45°, y, 135°), and the associated measurement uncertainty. In addition, since the work targets wearable antennas, it should be clarified whether these measurements were conducted in free space, on cloth, or on-body/phantom conditions, as body loading can significantly affect resonance and impedance matching.*

**Our response:**

Thank you for this valuable comment. We agree that a clear description of the stretching setup and strain quantification procedure is important for reproducibility. In the revised manuscript, we have added a photograph/schematic of the custom-built stretching fixture and expanded the experimental protocol for strain-dependent measurements. Specifically, the antenna under test was mounted on a custom-built nonmagnetic and nonconductive stretching fixture and held at a fixed position by two clamps/fixtures, as shown in **Figure S46a**. The applied tensile strain was controlled by adjusting the side screws/knobs of the stretcher, which gradually increased the separation between the two fixtures while maintaining the sample in a well-defined loading configuration. The applied strain was quantified from the change in the characteristic length of the antenna patch along the loading direction. As illustrated in **Figure S46b**, the initial gauge length before stretching was defined as $L_0$ in the unstrained state, and the corresponding length after stretching was defined as $L_1$. The tensile strain was then calculated as

$$\text{Strain} = \frac{L_1 - L_0}{L_0}.$$

During the stretching process, the patch length was continuously monitored using a ruler as the screws were gradually adjusted to increase the applied strain, and the strain level was determined from the measured length difference before and after stretching. In this way, the strain level could be accurately adjusted and verified over the range from 0% to 45%. To investigate directional strain sensitivity, the antenna was mounted in four different orientations using the stretching fixture so that the applied tensile axis was aligned with the $x$, 45°, $y$, and 135° directions, respectively, as shown in **Figure S46c**. For each direction, the sample was first fixed in the corresponding orientation, and then the same stretching and length-based strain quantification procedure was applied. This ensured that the strain axis was well defined and maintained throughout the measurement. We have added the following information in the revised manuscript: (1) the structure of the stretcher, (2) the definition and verification of the applied strain using $L_0$ and $L_1$, and (3) the method used to realize the four loading directions. These additions improve the reproducibility of the measurement procedure and clarify the direction-resolved, strain-dependent S-parameter measurements.

In addition, we would like to clarify that the strain-dependent S-parameter measurements were conducted in **free-space conditions**, rather than on-cloth or on-body during stretching. To address the reviewer's concern regarding wearable operation and possible body-loading

effects, we further investigated the proposed antenna under body-loading conditions using both simulations and experimental measurements.

Firstly, as shown in **Figure S42a-i**, the antenna was analyzed using a simplified multilayer tissue model consisting of skin, fat, and muscle. The results in **Figures S42a-ii** and **iii** show that the reflection coefficient and realized gain pattern under on-tissue conditions remain very close to those in free space, with only a slight frequency shift and negligible performance degradation. Furthermore, the antenna was evaluated using the anatomically realistic "Gustav" human voxel model in CST Studio Suite (**Figure S42b-i**), with the antenna placed on the chest to emulate a practical wearable scenario. Compared with the simplified layered tissue model, this phantom provides a more representative assessment of body-loading effects. As shown in **Figures S42b-ii** and **iii**, the simulated reflection coefficient under on-phantom conditions remains close to that in free space, with only a slight resonance shift, while the realized gain pattern is only minimally affected. These simulated results confirm that body proximity has little influence on the proposed antenna performance.

Finally, we have added additional experimental measurements of the reflection coefficient ($S_{11}$) of the proposed antenna under several representative wearable conditions (e.g., placing the antenna on the forearm, calf, abdomen, and garment, photographs shown in **Figure S42c-i**), with the corresponding $S_{11}$ responses provided in **Figure S42c-ii**. The measured $S_{11}$ responses are very similar to those in air, with nearly unchanged resonance frequencies and no obvious deterioration in impedance matching.

In summary, the minimal body-loading effect is mainly attributed to the presence of the ground plane. This is a common characteristic of patch-type antennas, where the ground plane acts as a shield between the radiating element and the lossy human tissue, thereby reducing near-field coupling and helping preserve the resonance and impedance matching. Therefore, the free-space stretching measurements provide a reasonable and controlled approach for evaluating the intrinsic effect of mechanical deformation on the antenna response. Thus, we have added the information in the revised manuscript.

**Our modification to the manuscript:**

"……

**2.5 End-to-end wireless communication performance through SP-MSiA**

Unlike the free-space WEH validation, the BLE communication study is conducted in a

wearable configuration by mounting the SP-MSiA on a smart T-shirt, thereby enabling direct evaluation of communication robustness under practical on-body deformation. To assess the on-body safety of the proposed antenna for wearable operation, SAR analysis is conducted to confirm low SAR values, consistent with the effective shielding provided by the ground plane (**Figure S41**). Furthermore, both simulations (**Figures S42ab**) and wearable measurements (**Figure S42c**) confirm that the proposed antenna is minimally affected by body loading, because the ground plane effectively shields the radiator from lossy biological tissue, suppresses near-field coupling, and thereby preserving the resonance and impedance matching under practical wearable conditions. With a thin stretchable nanogel adhesive applied to the antenna ground plane, the SP-MSiA is bonded to the right pectoral region of a smart T-shirt with embedded electrocardiogram (ECG) electrodes and stretchable interconnects. **……"**

**"……**

***Characterization of mechanical-electromagnetic properties:***

The stress-strain curve of Ecoflex-LMPs composite was measured by the M230pro (YiGao) at a tensile speed of 30 mm $min^{-1}$. Strain-dependent S-parameters were measured using a custom-built nonmagnetic and nonconductive stretcher **(Figure S46a)** connected to a VNA (T5260A, Transcom Instruments, 50 kHz–6 GHz) calibrated by the open-short-load-through method to the SMA reference plane. The antenna was clamped at both ends, and uniaxial strain was applied by increasing the clamp separation with side adjustment screws. The tensile strain was defined as $(L_1 - L_0)/L_0$, where $L_0$ and $L_1$ are the characteristic gauge lengths of the patch **(Figure S46b)** along the loading direction before and after stretching, respectively. The strain level was verified from direct length measurement after each stretching step. Direction-dependent measurements were performed by aligning the loading axis with the *x*, 45°, *y*, and 135° directions **(Figure S46c)**. Unless otherwise noted, these measurements were conducted in free space without textile, phantom, or on-body loading.

An open-short-load-through (SOLT) calibration was conducted to set the measurement reference plane at the SMA connector. **……"**

**"……**Polarization alignment between the antenna under test and the horn was maintained for all measurements. The received signal was recorded at each angular position to obtain the radiation patterns under different strain conditions.

The strain-dependent measurements of the proposed antenna were conducted in free space due to the small performance difference between free-space and wearable conditions.

As confirmed by the additional on-tissue, on-phantom, and representative on-body/on-garment results **(Figure S42)**, body loading causes only slight resonance shifts and negligible degradation in impedance matching, mainly due to the shielding effect of the ground plane. Therefore, the free-space stretching measurements provide a reasonable and controlled approach for evaluating the intrinsic effect of mechanical deformation on the antenna response. **……**"

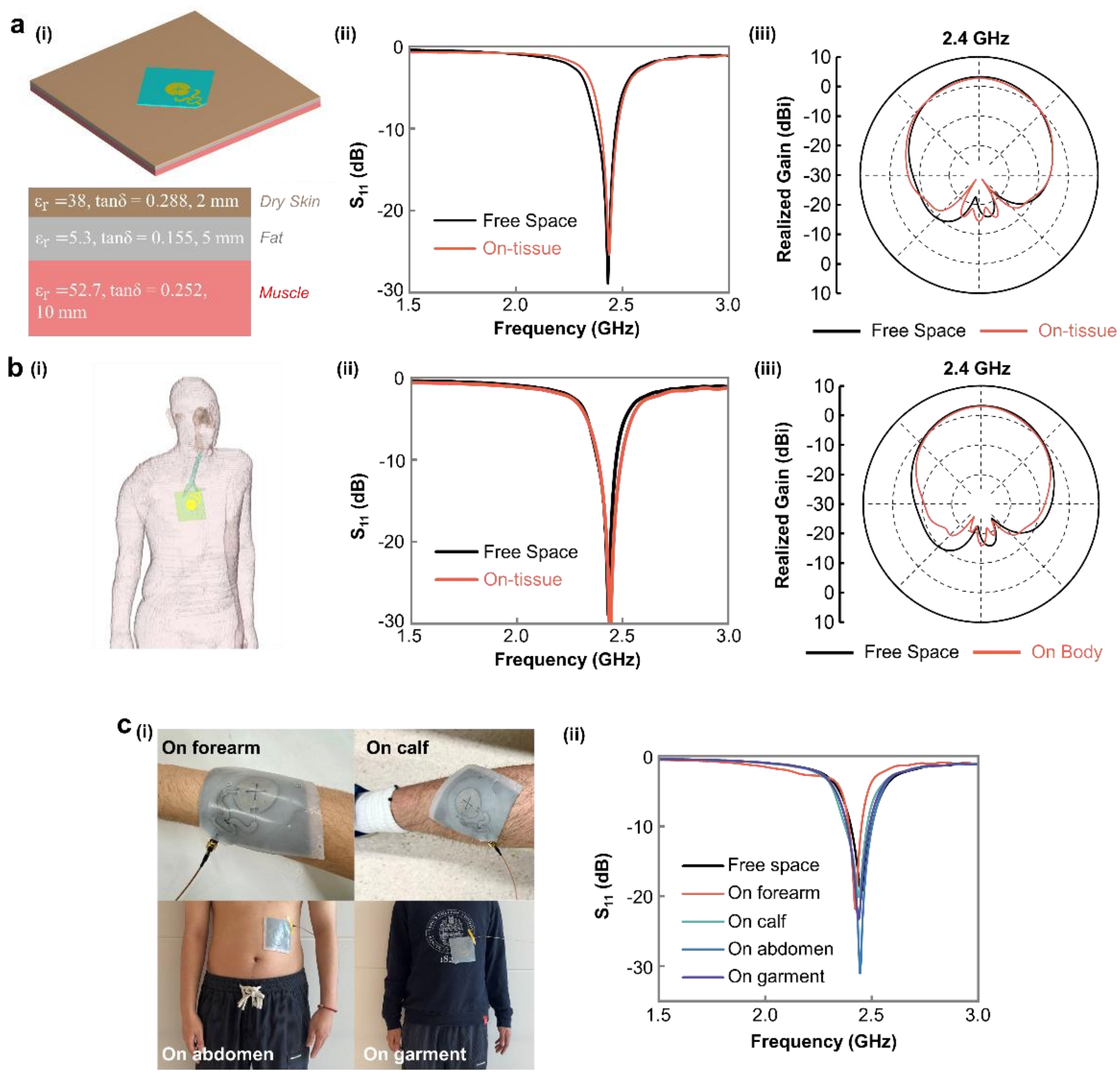


**Figure S42. Simulated and measured body-loading effects of the DP-MSiA.** **(a)** (i) Three-layer tissue model, simulated (ii) $S_{11}$, and (iii) realized gain patterns in free space and on tissue. **(b)** (i) "Gustav" human voxel model, simulated (ii) $S_{11}$, and (iii) realized gain patterns in free space and on

the body. **(c)** (i) Wearable configurations on the forearm, calf, abdomen, and garment, and (ii) corresponding measured $S_{11}$ responses.

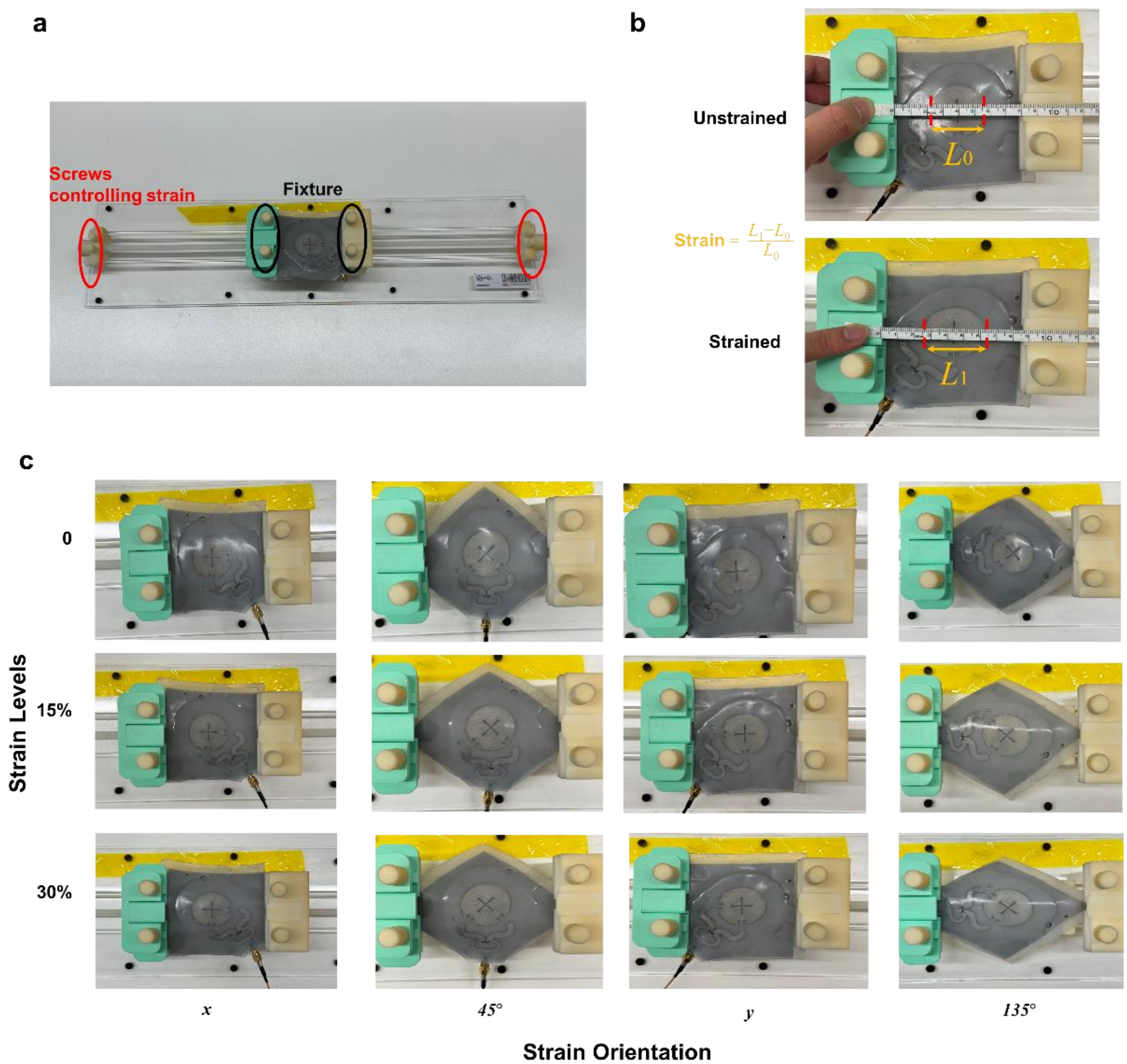


**Figure S46.** **(a)** Experimental setup of the custom-built nonmagnetic and nonconductive stretcher for strain-dependent antenna measurements. **(b)** Illustration of the strain calculation method, where strain is determined from the change in the characteristic patch length as $(L_1$-$L_0)/L_0$. **(c)** Photographs of the antenna under different loading directions ($x$, 45°, $y$, and 135°) and strain levels (0%, 15%, and 30%).

*2. The validation conditions used for the WEH platform (DP-MSiA) and the BLE communication platform (SP-MSiA) are not clearly aligned. The WEH demonstration is described as a far-field rectenna experiment with a commercial transmitting patch antenna placed 1 m away, but it is unclear whether the receiving rectenna was measured in free space or under on-body/garment conditions. In contrast, the BLE communication demonstration explicitly attaches the antenna to a smart T-shirt and evaluates link performance under garment stretching. Since the manuscript emphasizes wearable applications, the authors should clarify the measurement conditions used for both DP-MSiA and SP-MSiA and ideally provide a comparison between free-space and on-body (or phantom) antenna performance under representative strain levels to ensure that the two demonstrations are evaluated under consistent and realistic wearable scenarios.*

**Our response:**

Thank you for this important comment. We agree that the validation conditions of the DP-MSiA-based WEH platform and the SP-MSiA-based BLE communication platform should be more clearly distinguished. In our experiments, the WEH platform was measured in air (free-space condition) rather than under on-body or garment-mounted conditions, because this experiment was designed to experimentally verify the strain-insensitive RF response of the antenna and rectifying system under controlled excitation, using LED illumination as a direct physical indicator of stable antenna performance under stretching. In contrast, the BLE communication platform was specifically evaluated in a wearable configuration by attaching the antenna to a smart T-shirt and examining its performance under stretching, since this experiment was intended to demonstrate the practical applicability of the proposed system in wearable communication applications. This distinction has been clarified in the revised manuscript in both the main text and the experimental section.

To further address the reviewer's other concern, we additionally investigated the body-loading effects of the DP-MSiA through both simulations and measurements. The $S_{11}$ and radiation efficiency results consistently show that the proposed antenna exhibits only minimal body-loading effects, with only slight resonance shifts and negligible impedance degradation relative to the free-space condition. These findings highlight the critical role of the ground-backed patch configuration in wearable operation, as it effectively shields the radiator from the lossy human body and suppresses near-field coupling. Therefore, the antenna performance is well preserved under body-mounted conditions, which further supports the wearable applicability of both the communication and wireless energy harvesting demonstrations.

**Our modification to the manuscript:**

“……

**2.5 End-to-end wireless communication performance through SP-MSiA**

Unlike the free-space WEH validation, the BLE communication study is conducted in a wearable configuration by mounting the SP-MSiA on a smart T-shirt, thereby enabling direct evaluation of communication robustness under practical on-body deformation. To assess the on-body safety of the proposed antenna for wearable operation, SAR analysis is conducted to confirm low SAR values, consistent with the effective shielding provided by the ground plane (**Figure S41**). Furthermore, both simulations (**Figures S42ab**) and wearable measurements (**Figure S42c**) confirm that the proposed antenna is minimally affected by body loading, because the ground plane effectively shields the radiator from lossy biological tissue, suppresses near-field coupling, and thereby preserves the resonance and impedance matching under practical wearable conditions. With a thin stretchable nanogel adhesive applied to the antenna ground plane, the SP-MSiA is bonded to the right pectoral region of a smart T-shirt with embedded electrocardiogram (ECG) electrodes and stretchable interconnects. **……”**

“……

***Characterization of mechanical-electromagnetic properties:***

**……**Direction-dependent measurements were performed by aligning the loading axis along the *x*, 45°, *y*, and 135° directions. Unless otherwise noted, these measurements were conducted in free space without textile, phantom, or on-body loading. **……** Polarization alignment between the antenna under test and the horn was maintained for all measurements. The received signal was recorded at each angular position to obtain the radiation patterns under different strain conditions.

The strain-dependent measurements of the proposed antenna were conducted in free space due to the small performance difference between free-space and wearable conditions. As confirmed by the additional on-tissue, on-phantom, and representative on-body/on-garment results, body loading causes only slight resonance shifts and negligible degradation in impedance matching **(Figure S42)**, mainly due to the shielding effect of the ground plane. Therefore, the free-space stretching measurements provide a reasonable and controlled approach for evaluating the intrinsic effect of mechanical deformation on the antenna response. **……”**

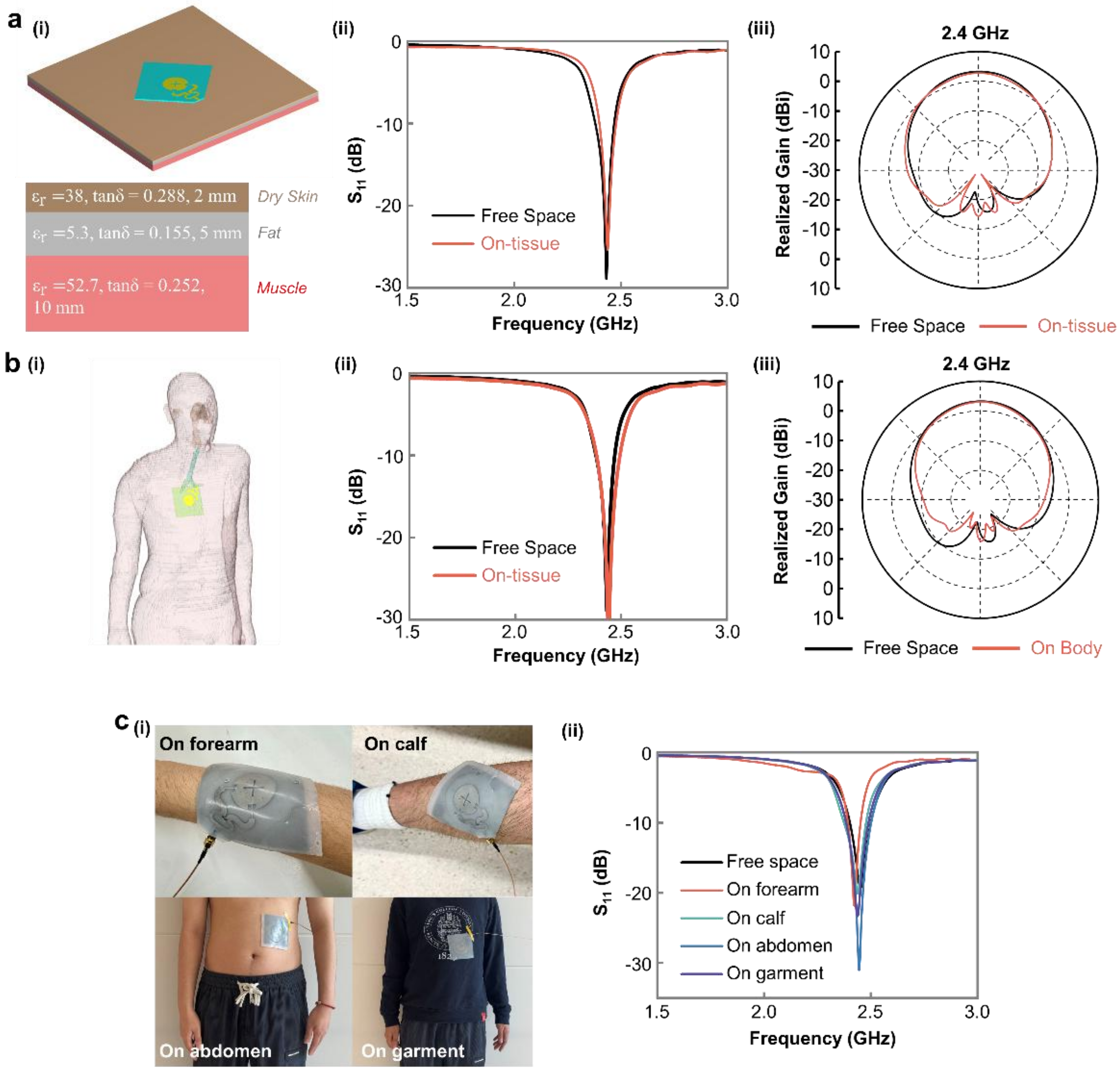


**Figure S42. Simulated and measured body-loading effects of the DP-MSiA.** **(a)** (i) Three-layer tissue model, simulated (ii) $S_{11}$, and (iii) realized gain patterns in free space and on tissue. **(b)** (i) "Gustav" human voxel model, simulated (ii) $S_{11}$, and (iii) realized gain patterns in free space and on the body. **(c)** (i) Wearable configurations on the forearm, calf, abdomen, and garment, and (ii) corresponding measured $S_{11}$ responses.

*3. The wireless energy-harvesting validation relies primarily on LED illumination and the output voltage measured across a 160-Ω load, together with a "failure strain" definition; while these demonstrations are visually compelling, they do not provide a reproducible, quantitative rectenna/WPT assessment. Please report the transmit conditions in a form that enables*

*replication and fair comparison (e.g., PA output power or delivered power to the Tx antenna, Tx antenna gain, polarization/alignment, and the resulting EIRP or estimated incident power density/field strength at 1 m), because the current description ("3 dBm signal generator + amplifier") is insufficient to infer the RF exposure at the receiver. In addition, please include RF-to-DC conversion performance (e.g., DC output power and/or PCE versus incident power or received power) and its variation versus strain/direction; at minimum, converting the reported Vout across 160 Ω to Pout (=V²/R) and defining a voltage/power-based "failure" criterion (rather than an LED-threshold-dependent criterion) would substantially improve rigor and comparability.*

**Our response:**

Thank you for this important and constructive comment. We would like to clarify that the LED-illumination experiment was included primarily as an intuitive and application-oriented demonstration, to visually reflect the practical benefit of the strain-insensitive antenna design for energy capture under mechanical deformation. Therefore, this result should be regarded as a qualitative demonstration rather than a quantitative performance metric. For a more rigorous evaluation, we further conducted measurements using a 160-ohm load, which allowed quantitative extraction of the DC output voltage. However, we agree that the wireless energy-harvesting (WEH) demonstration should be described in a more quantitative and reproducible manner. In response, we have revised the manuscript to report the transmit conditions more explicitly. Specifically, for the WEH experiment quantitatively measured using 160-ohm load, the experiment was performed using a 3 dBm signal generator followed by a 30 dB power amplifier, corresponding to a power of approximately 33 dBm delivered to the transmitting antenna. A commercial transmitting antenna with a gain of approximately 19 dBi was used, and the transmitting and receiving antennas were arranged in boresight alignment with matched linear polarization at a separation distance of 1 m.

In addition, we have added the corresponding quantitative analysis by converting the measured output voltage across the 160-ohm load into DC output power ($P_{out} = V^2/R$) and normalized RF-to-DC efficiency. We have further compared their variations under different strain levels and stretching directions. Specifically, the DC output power ($P_{out} = V^2/R$) and normalized RF-to-DC efficiency were calculated as follows:

The estimated received RF power was calculated using the Friis transmission equation,

$$P_r = P_t + G_t + G_r - L_{FS},$$

where $P_t$ is the power delivered to the transmitting antenna, $G_t$ and $G_r$ are the gains of the transmitting and receiving antennas, respectively, and $L_{FS}$ is the free-space path loss. At 2.45 GHz and 1 m, the free-space path loss is

$$L_{FS} = 20\log_{10}\left(\frac{4\pi R}{\lambda}\right) \approx 40.22\ \text{dB}$$

Using $P_t$ =33 dBm, $G_t$ =19 dBi, and $G_r$ =3.3 dBi, the estimated received power at the receiving antenna is

$$P_r = (33 + 19 + 3.3 - 40.22)\ \text{dBm} = 15.08\ \text{dBm} = 32.2\ \text{mW}$$

In addition, the output voltage measured across the 160-ohm load has now been converted to DC output power using

$$P_{DC} = \frac{V_{out}^2}{R}.$$

To enable consistent comparison of energy-harvesting performance across different strain states, we further used the estimated received RF power in the unstrained state as a common reference for normalization. Accordingly, this quantity should be interpreted as a normalized RF-to-DC performance metric referenced to the unstrained condition, rather than the true power conversion efficiency (PCE). Specifically, the normalized RF-to-DC efficiency was calculated as:

$$\text{Normalized RF-to-DC efficiency} = \frac{P_{DC}}{P_{r,0}},$$

where both $P_{DC}$ and $P_{r,0}$ are expressed in mW, and $P_{r,0}$ denotes the estimated received power at the receiving antenna in the unstrained state. The converted DC output power and normalized RF-to-DC efficiency as functions of strain are presented in **Figure S29**.

Regarding the "failure" criterion, although a voltage- or power-based "failure" criterion under a fixed 160-ohm load is more quantitative than an LED-based criterion, it still depends inherently on both the load impedance and the input-power level, and is therefore not universal. The purpose of this figure was not to establish a universal failure criterion or to isolate the intrinsic performance of the rectifier itself, but rather to illustrate that the performance difference between the proposed strain-insensitive antenna and the strain-sensitive counterpart can become very pronounced under certain practical operating conditions. In this sense, the figure serves as an application-oriented demonstration rather than a universal benchmark, which we believe is difficult to define for stretchable rectennas. For this reason, in the revised manuscript, we have added $P_{DC}$ and normalized RF-to-DC efficiency under the fixed 160-ohm load as quantitative comparison metrics, and we also report the corresponding source conditions as suggested by the reviewer, while the LED-illumination result is retained as a practical functional demonstration. We believe these revisions could provide a more reproducible and quantitative rectenna/WPT assessment and allow a fairer comparison with related studies.

**Our modification to the manuscript:**

“

······By contrast, the DP-MSiA-based platform features a failure strain of at least 45% along all tested directions, which supports a (quasi-)omnidirectional (360°) strain-insensitivity within the tensile strain from 0 to 40%.

For quantitative characterization, the rectenna output across a 160-ohm load is evaluated in a free-space, boresight-aligned, polarization-matched configuration at a transmitter-receiver separation of 1 m using a 3 dBm signal generator, a 30 dB power amplifier, and a commercial transmitting patch antenna with a gain of 19 dBi, corresponding to an estimated received RF power at the rectenna of $P_r$ =15.08 dBm. The output voltage is measured as a function of tensile strain from 0% to 60% along the four representative directions (**Figure 4e**). Different from the pronounced voltage drop from 2.0 V to ~0.5 V with increasing strain from 0% to 60% in the CDPA-based WEH platform, the DP-MSiA-based WEH platform maintains a stable output voltage up to ~40% stretching (followed by a slight drop to ~1.6 V upon 60% stretching). The measured voltages are further converted to DC output power and normalized RF-to-DC efficiency, with the efficiency values referenced to the estimated received RF power in the unstrained state to provide a common basis for comparison across different strain states (**Figure S29**). Specifically, the DP-MSiA-based platform maintains a high normalized RF-to-DC efficiency above 40% at 60% strain, while that of the CDPA-based platform decreases to only around 5% at 60% strain, depending on the stretching direction. The results confirm the importance of the cross-shaped slot structure and dual-port configuration in achieving multidirectional, strain-insensitive RF energy harvesting. ······”

“······

***Demonstrations of RF energy harvesting:*** For the LED-illumination demonstration, an RF signal at 2.45 GHz with an output power of 3 dBm was generated using a tunable signal generator (SMA100B, Rohde & Schwarz) connected to a power amplifier and a commercial transmitting wall-mounted patch antenna (2.4 G, Aihua). The three wireless energy harvesting systems were positioned 1 m from the transmitter. The rectennas were tested under a 90° off-boresight orientation to reduce the received RF power, making the effect of mechanical deformation more distinguishable. The rectenna output was directly connected to an LED as the load, with the LED state under different stretching conditions visually recorded.

For the quantitative WEH experiment using a 160-ohm load, the experiment was

performed using a 3 dBm signal generator followed by a 30 dB power amplifier, corresponding to a power of approximately 33 dBm delivered to the transmitting antenna. A commercial transmitting antenna with a gain of approximately 19 dBi was used, and the transmitting and receiving antennas were arranged in boresight alignment with matched linear polarization at a separation distance of 1 m. The rectenna output was connected to a fixed 160-ohm load resistor, and the DC output voltage across the load was measured under different strain levels and stretching directions. The measured output voltage was then converted into DC output power according to $P_{DC} = V^2/R$, where $R$ = 160 ohm. The normalized RF-to-DC efficiency was further calculated as $P_{DC}/P_{r,0}$, where $P_{r,0}$ is the estimated RF power received by the rectenna in the unstrained state. ……"

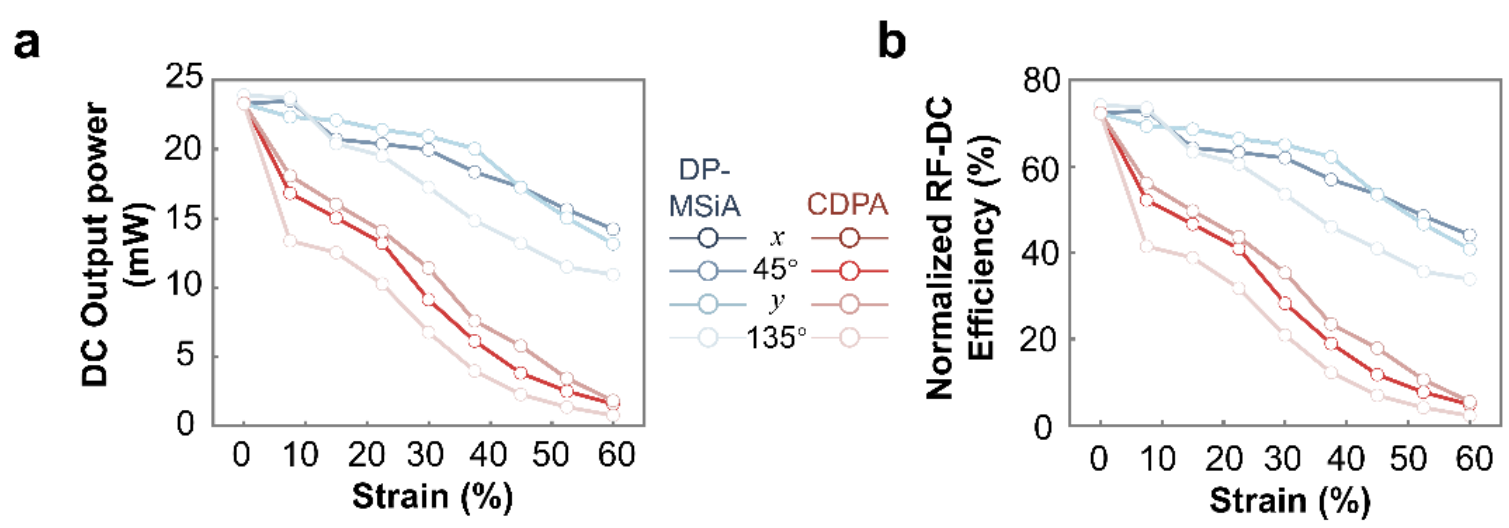


**Figure S29. Quantitative WEH performance comparison between the proposed DP-MSiA and the conventional CDPA under multidirectional stretching.** **(a)** DC output power measured across the 160-ohm load as a function of strain. **(b)** Estimated normalized RF-to-DC efficiency as a function of strain. Results are shown for four representative in-plane stretching directions (*x*, 45°, *y*, and 135°).

*4. The SP-MSiA configuration critically relies on the Wilkinson power divider, and the manuscript claims that the PD maintains nearly unchanged S-parameters under stretching. However, this claim is not sufficiently supported by experimental data. Because the Wilkinson divider is a quarter-wave network whose electrical properties may change under mechanical deformation, it is important to experimentally validate its strain tolerance. The authors should therefore provide measured S-parameters (e.g.,* $S_{11}$*,* $S_{21}$*, and isolation) of the power divider under representative strain levels and directions to substantiate the claim that the feed network remains electrically stable under deformation.*

**Our response:**

Thank you for this constructive comment. We agree that, because the SP-MSiA relies on a Wilkinson power divider (PD) as its feeding network, the electrical stability of the PD under mechanical deformation should be experimentally verified. In response, we fabricated a standalone stretchable Wilkinson PD and added its measured S-parameters under applied strain to the revised manuscript.

As shown in **Figure 5c-i**, the fabricated PD was characterized as a three-port device, with Port 1 serving as the input and Ports 2 and 3 as the two output branches. To evaluate directional strain tolerance, the standalone PD was stretched under representative loading conditions along the *x*- and *y*-directions, corresponding to the two principal deformation modes of the feed network. The measured results are shown in **Figure 5c-ii** and **-iii**. Specifically, **Figure 5c-ii** presents the **measured** $S_{11}$, $S_{12}$, and $S_{23}$ of the PD under tensile strain applied along the ***x* direction**, while **Figure 5c-iii** shows the corresponding **measured** results under strain applied along the ***y* direction**. The strain varied from **0% to 40%**, as indicated in the figure. From the results, it can be seen that the transmission characteristic of the power divider remains highly stable under deformation. In particular, the measured $S_{12}$ stays close to the expected power-division level of above -4 dB over the investigated strain range for both the *x*- and *y*-direction loading cases, with only very small variation. In addition, the measured input matching $S_{11}$ and output isolation $S_{23}$ preserve their overall responses around the operating band, although minor changes in resonance depth and frequency can be observed under stretching. These variations are limited and do not alter the fundamental power-division behavior of the feed network.

**Our modification to the manuscript:**

"**……**The antenna is designed in a planar microstrip using the same LMPs-based conductive layer to ensure mechanical compliance and consistent stretchability (**Figure 5b**), exhibiting strain-insensitive characteristics. To experimentally validate the strain tolerance of the feeding network, a standalone stretchable Wilkinson PD is fabricated (**Figure 5c-i**) and measured. The S-parameters ($S_{11}$, $S_{12}$, and $S_{23}$) of the fabricated standalone PD remain largely stable across the 2.4-2.48 GHz ISM band under uniaxial stretching along both the *x*- and *y*-directions, with only minor variations observed as the strain increases (**Figures 5c-ii** and **iii**). These results confirm the stable RF performance of the stretchable PD under stretching, providing a reliable feeding network for the subsequent single-port antenna design.

Feeding the dual ports of DP-MSiA through a symmetric PD feedline network structure ensures in-phase excitation at both ports of the patch, since the electrical path length from the

PD input to each port is identical. **……"**

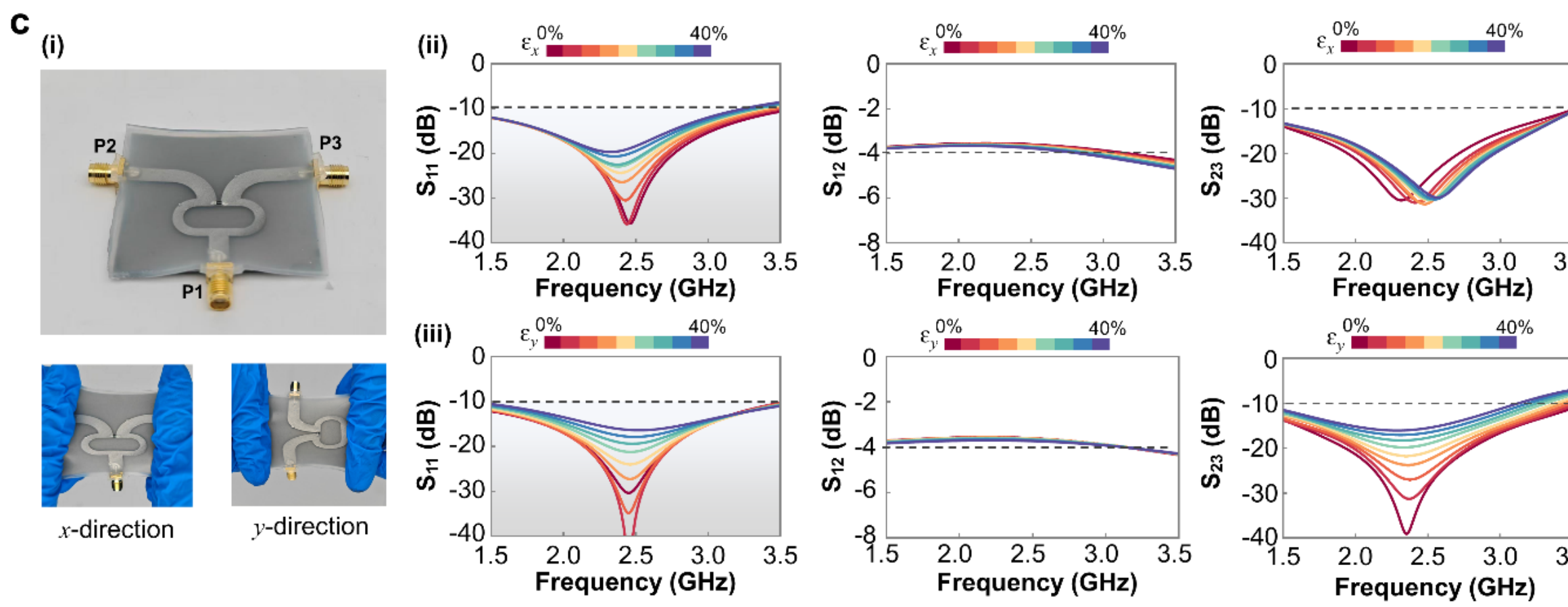


**Figure 5.** …… **(b)** Photographs of the SP-MSiA with representative uniaxial strain applied along (i) *x*, (ii) 45°, (iii) *y*, and (iv) 135° directions. **(c)** (i) Photographs of the fabricated standalone stretchable Wilkinson power divider (PD) before (top) and after (bottom) stretching. (ii) Measured S-parameters ($S_{11}$, $S_{12}$, and $S_{23}$) of the standalone stretchable Wilkinson PD under tensile strain applied along the (ii) *x*-direction and (iii) *y*-direction. **(d)** Measured $S_{11}$ of the SP-MSiA under tensile strain of 0 - 40% applied along (i) *x*, (ii) 45°, (iii) *y*, and (iv) 135° directions. ……**"**

*5. The wearable system incorporates rigid interfaces and discrete components (e.g., SMA connectors, the isolation resistor in the Wilkinson divider, and multiple components in the rectifier circuit) attached using conductive silver paste. Under large deformation, particularly diagonal stretching (e.g., 45°), these rigid–soft interfaces may experience stress concentration and potential mechanical failure. However, the manuscript does not present any durability or cyclic stretching tests. The authors should include at least a basic fatigue test (e.g., hundreds or thousands of cycles at representative strain levels) and report the resulting changes in $S_{11}$, WEH output voltage, or communication performance to demonstrate that the antenna and associated circuitry maintain stable operation under repeated mechanical deformation.*

**Our response:**

Thank you for this important comment. We agree that the mechanical durability of the rigid-soft interfaces and the discrete-component interconnections should be evaluated under repeated deformation, since these regions may be susceptible to stress concentration and

possible failure under large strain. In response, we performed cyclic stretching fatigue tests to evaluate the stability of both the communication platform and the WEH platform under repeated mechanical loading.

For the communication platform, a cyclic stretching test was carried out using 45° diagonal stretching. During the test, the antenna was manually stretched and released repeatedly, so that the evaluation simultaneously probed the cyclic durability of the stretchable antenna structure and the mechanical reliability of the rigid-soft connection, including the SMA/antenna interface. In each cycle, the antenna was stretched to approximately 50% tensile strain and then released to the undeformed state. The $S_{11}$ response was measured in the undeformed state after 0, 200, 400, and 600 cycles. In addition, the communication performance was evaluated in terms of the maximum communication distance after cyclic loading. For each cycle condition, the communication-distance measurement was repeated four times, and the results are presented as mean ± standard deviation with error bars. As shown in **Figure S45a,** only a minor variation in the $S_{11}$ response was observed after repeated deformation. Moreover, as shown in **Figure S45b**, the maximum communication distance remained above 100 m after 600 cycles, with only a small change from the initial state, confirming stable operation of the communication platform under repeated mechanical deformations.

For the WEH platform, we further examined the fatigue tolerance of the integrated rectifying system using two separate evaluations: (i) LED illumination and (ii) output voltage measured across a 160-Ω load. In each cycle, the device was stretched to approximately 50% tensile strain and then released to the undeformed state.

As a direct demonstration of practical functionality, the same WEH device was first evaluated by LED illumination under the same RF exposure conditions after cyclic loading. The stretching tests were performed sequentially along the *x*-axis and then the *y*-axis, since these deformation modes load the main interconnect regions containing the rigid components and their joints. The device remained capable of illuminating the LED after 600 stretching cycles along the *x*-axis and after 600 additional stretching cycles along the *y*-axis, as shown in **Figure S30a**, indicating preserved operational capability after repeated deformation. For the 160-ohm-load demonstration, the output voltage was measured after the cyclic stretching tests were completed. After stretching cycles, under the same RF exposure conditions, the output voltage was then measured across a 160-ohm load under representative strain levels applied along four directions (*x*, *y*, 45°, and 135°). It can be found that only a minor drop in output voltage was observed relative to the initial state, as shown in **Figure S30b**. For

example, at the highest measured strain, the output voltage decreased from about 1.7 V to about 1.5 V, indicating that the WEH platform maintained stable functionality after repeated deformations. This confirms that the integrated rectifier and its rigid-soft interconnections remained mechanically and electrically stable after repeated deformations.

**Our modification to the manuscript:**

"……The results confirm the importance of the cross-shaped slot structure and dual-port configuration in achieving multidirectional, strain-insensitive RF energy harvesting. In addition, cyclic stretching tests show that the WEH platform retains stable functionality after repeated deformations to illuminate the LED (**Figure S30a)**. It exhibits only a minor decrease in output voltage across a 160-ohm load after 600 stretching cycles along the *x*-axis, followed by 600 additional cycles along the *y*-axis (**Figure S30b)**.

……"

"……The sustained stable wireless transmission from the SP-MSiA provides the integrated system with uninterrupted ECG recordings even under 30% stretching along the *x*- and *y*-directions (**Figures 6c-ii, iii**), in direct contrast to the abrupt signal experienced by the system with the reference antenna. Furthermore, the cyclic mechanical reliability of the communication platform is also evaluated under repeated stretching to ~50% strain, showing only minor changes in $S_{11}$ (**Figure S45a**) and maintaining a maximum communication distance above 100 m even after 600 cycles (**Figure S45b**).

**2.6 Real-world applications of SP-MSiA-based wearable electronics**

……"

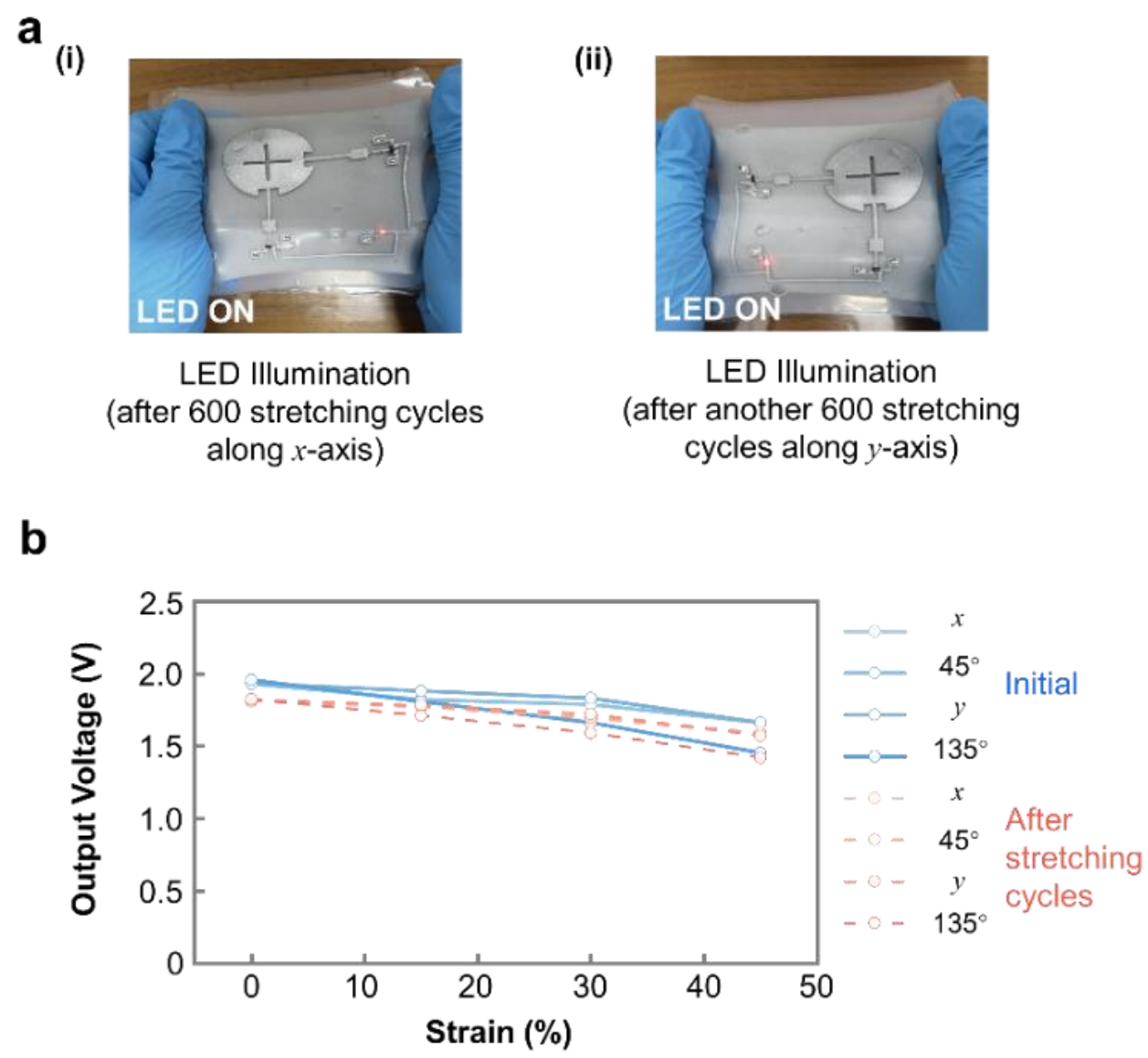


**Figure S30. Cyclic stretching characterization of the DP-MSiA-based WEH system. (a)** Photographs of LED illumination after cyclic stretching along the (i) *x*-axis and (ii) *y*-axis. **(b)** Output voltage versus strain measured along the *x*, 45°, *y*, and 135° directions before and after cyclic stretching.

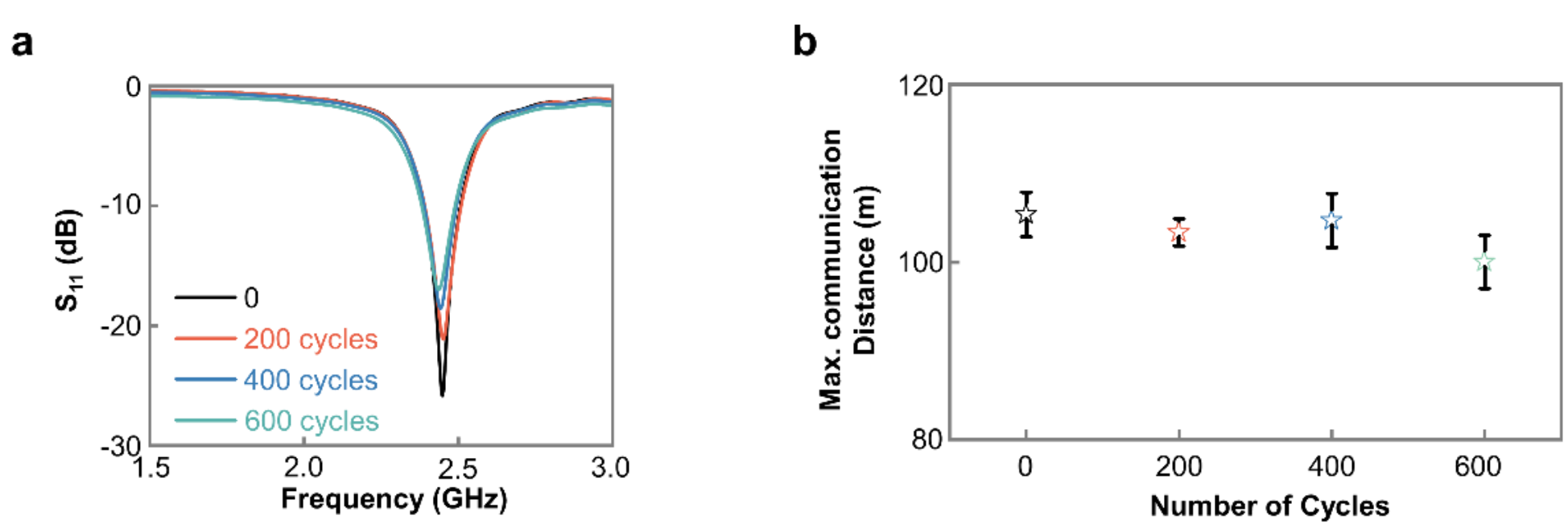


**Figure S45. Cyclic stretching characterization of the SP-MSiA. (a)** Measured $S_{11}$ of the SP-MSiA after 200, 400, and 600 stretching cycles. **(b)** Maximum communication distance of the SP-MSiA as a function of the stretching cycle number.

*6. In the "real-world applications" demonstration (Fig. 7), the manuscript labels the hand-raising motion as approximately $\varepsilon_y \approx 30\%$ and the chest-opening motion as approximately $\varepsilon_x \approx 30\%$. However, the manuscript does not explain how these strain values were determined during human motion. Without a quantitative measurement method (e.g., marker-based distance change, simple*

*gauge-length estimation, or video-based tracking), it is difficult to verify the stated strain levels. The authors should either provide a quantitative method used to estimate these strain values or soften the claim by describing these motions as qualitative large deformations with approximate strain estimates.*

**Our response:**

We thank you very much for this helpful comment. In our experiment, the strain values indicated for the real-world motion demonstrations were estimated using a simple gauge-length method. Specifically, the dimension of the radiating patch along the corresponding strain direction was measured with a ruler before the motion and again after the motion was completed, corresponding to an approximately maximum deformation state. The strain was then estimated using the engineering-strain definition $\varepsilon = (L - L_0)/L_0$, where $L_0$ is the initial patch dimension and $L$ is the patch dimension after deformation.

Based on this method, the hand-raising motion and chest-opening motion were estimated to produce approximate strains of $\varepsilon_x \approx 30\%$ and $\varepsilon_y \approx 30\%$, respectively. The patch dimension along the principal strain axis was used as the representative metric because the antenna resonance is primarily governed by the effective dimension of the radiating patch, whereas dimensional changes in other regions, such as the feed line or ground, are expected to have a significantly smaller influence on the resonance behavior.

In the revised manuscript, we have clarified that these values are approximate strain estimates obtained from direct ruler-based measurement of the patch dimension along the relevant strain axis during human motion in the **Experiment Section**.

**Our modification to the manuscript:**

**"*Demonstrations of end-to-end far field wireless communication of SP-MSiA:***

…… The transmitting antenna was deformed by manually stretching the smart T-shirt to control the deformation magnitude and direction. Moreover, the strain values were approximately estimated using a simple gauge-length method based on the patch dimension measured before and after deformation. The received signals were then recorded by placing the receiver tablet at various distances from the transmitter to measure the efficient communication distances (ECD) and link-failure strain."

*7. The Abstract states that the DP-MSiA exhibits a resonance shift of ≤20 MHz at 2.45 GHz under up to 45% strain across multiple in-plane directions. However, other sections of the manuscript describe the strain-invariant resonance behavior using percentage-based values (e.g., approximately 1.8%), which may exceed 20 MHz depending on how the resonance is defined. The authors should clarify the method used to extract the resonance frequency and provide a consistent definition across the manuscript. A summary table or plot showing the extracted resonance shift versus strain and direction would help ensure that the headline claim is fully supported by the presented data.*

**Our response:**

Thank you very much for this careful observation. We agree that the method used to define and report the resonance shift should be clarified and presented consistently throughout the manuscript. After re-examining the extracted data, we found that the previous description was not sufficiently precise and could lead to confusion. In this work, the resonance frequency was extracted from the measured $S_{11}$ response as the frequency at which $S_{11}$ reaches its minimum value. To ensure consistency, the resonance variation is now defined in the revised manuscript in terms of the absolute frequency shift in MHz, rather than as a percentage. Specifically, for each stretching direction, the reported resonance shift, $\Delta f$, is defined as the difference between the extracted resonance frequency under strain and that in the unstrained state, i.e.,

$$\Delta f = f_{\text{strained}} - f_0$$

where $f_0$ is the resonance frequency in the unstrained state and $f_{\text{strained}}$ is the extracted resonance frequency at the corresponding strain level.

Based on this consistent definition, the previously stated ≤ 20 MHz value corresponds only to the *x*- and *y*-direction stretching cases, whereas the 45° and 135° diagonal stretching cases give a maximum resonance shift of 40 MHz (across the four representative in-plane directions as well). We thank the reviewer for identifying this inconsistency. In the revised manuscript, we have corrected the Abstract and related discussion accordingly, and we now use the absolute resonance-frequency shift in MHz consistently throughout. In addition, we have also added a summary plot **(Figure S23)** and a summary table **(Table S2)** showing the extracted resonance frequencies and corresponding $\Delta f$ values versus strain level and stretching direction for both proposed DP-MSiA and conventional antenna.

**Our modification to the manuscript:**

“**Abstract:** …… Here, we establish a systematic directional mechano-electromagnetic analysis framework for resonant planar antennas and introduce a dual-port multidirectional strain-insensitive antenna (DP-MSiA), whereby strain-insensitive resonance (shift ≤ 40 MHz at 2.45 GHz) is achieved under up to 45% strain across diverse in-plane directions. Based on the stable resonance and high realized gain of the DP-MSiA, we demonstrate strain-insensitive wireless energy harvesting with rectifiers under in-plane strain of varying direction and magnitude, as well as a strain-robust on-body communication system that sustains stable multimodal health-data transmission during natural motion. ……”

“…… When the tensile strain of 30% is applied along 45 and 135° (**Figures 3d-ii**, **iii**), the optimal initial slot length increases slightly from 18 to ~ 19 mm. However, the frequency shift $\Delta f$ is only 20 MHz at 18 mm to cause a negligible difference for practical applications. ……”

“…… This introduces a finite current component along that axis and therefore increases inter-port coupling. In addition, the $S_{11}$ of Port 1 exhibits a remarkable strain-insensitive property, with resonance frequency essentially unchanged (a maximum of 12 MHz shift) at 2.45 GHz under tensile strain of up to 45% along the *x*-direction (**Figure 3e-i**), in good agreement with simulation results (**Figure S17**). In contrast, Port 2 shows an almost linear redshift in its resonance with increasing *x*-direction strain (**Figure S18**). Due to symmetry, the S-parameter characteristics of Port 1 and Port 2 switch roles upon stretching in the *y*-direction, with Port 2 exhibiting a maximum frequency shift of only 20 MHz (**Figure S19**), consistent with the port-mode selectivity discussed above. Under diagonal stretching along the 45° or 135° directions, each port can couple to both modes and the $S_{11}/S_{22}$ response exhibits two resonances as strain increases. For Port 1 excitation, one resonance (Mode 1) redshifts with increasing strain, while the other (Mode 2) remains strain-insensitive (maximum of 40 MHz shift) near 2.45 GHz (**Figures 3e-ii**, **iii**). The same behavior is observed for Port 2 excitation (**Figure S20**) as diagonal stretching is along the symmetric axis of the antenna. Overall, the DP-MSiA exhibits robust strain insensitivity for all four stretching directions, whereas conventional dual-port counterparts without slots suffer from substantial frequency detuning upon stretching (**Figures S21** and **S22**). A summary plot showing the extracted frequency shift ($\Delta f$) versus strain level and stretching direction for both the proposed DP-MSiA and the conventional reference antenna is provided in **Figure S23** and **Table S2**.

……”

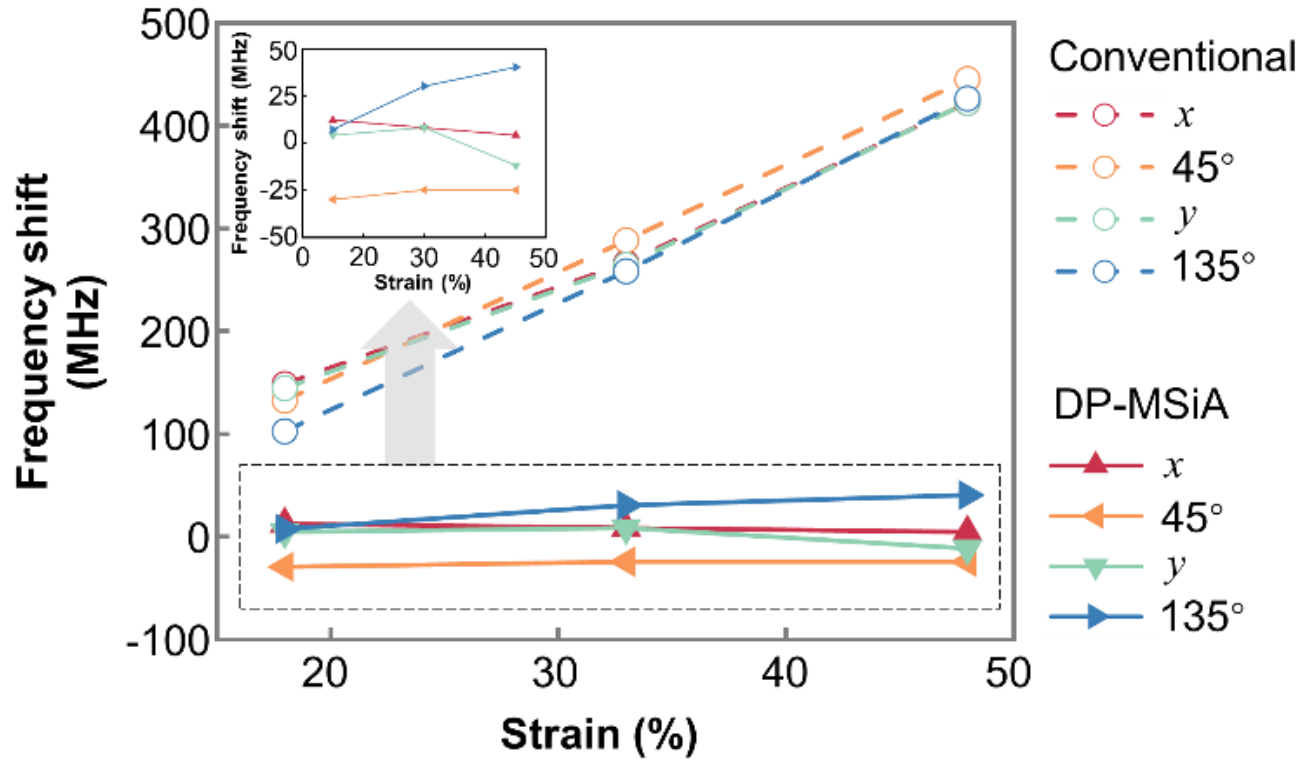


**Figure S23.** Extracted frequency shift (Δ*f*) of the proposed DP-MSiA and the conventional antenna as a function of strain level under four representative in-plane stretching directions (*x*, 45°, *y*, and 135°).

**Table S2. Resonance frequency shifts of the DP-MSiA under different levels and orientations of uniaxial strain.**

| Orientation / Strain | *x* direction | 45° direction | *y* direction | 135° direction |
|---|---|---|---|---|
| 15% | 12 MHz | -30 MHz | 4 MHz | 7 MHz |
| 30% | 8 MHz | -25 MHz | 8 MHz | 30 MHz |
| 45% | 4 MHz | -25 MHz | -12 MHz | 40 MHz |

***Minor comments:***

*1. In the Supplementary Information, Fig. S28 refers to a "two-port patch antenna with the power divider," which may be confusing because a power-divider-fed antenna effectively operates as a single-input system even if the radiator has two feeds. The authors should clarify the terminology to distinguish between the radiator feed configuration and the overall system input configuration.*

**Our response:**

Thank you for pointing out this terminology issue. We agree that the phrase "two-port patch antenna with the power divider" may be confusing, because although the radiator contains two feed branches, the incorporation of the power divider makes the overall antenna system a single-input configuration rather than a true two-port input system. We apologize

for this imprecise wording. In response, we have revised the terminology in the Supplementary Information for clarity, and now refer to this reference antenna as “a no-slot single-input antenna system with a power-divider-fed two-branch patch radiator.”

**Our modification to the manuscript:**

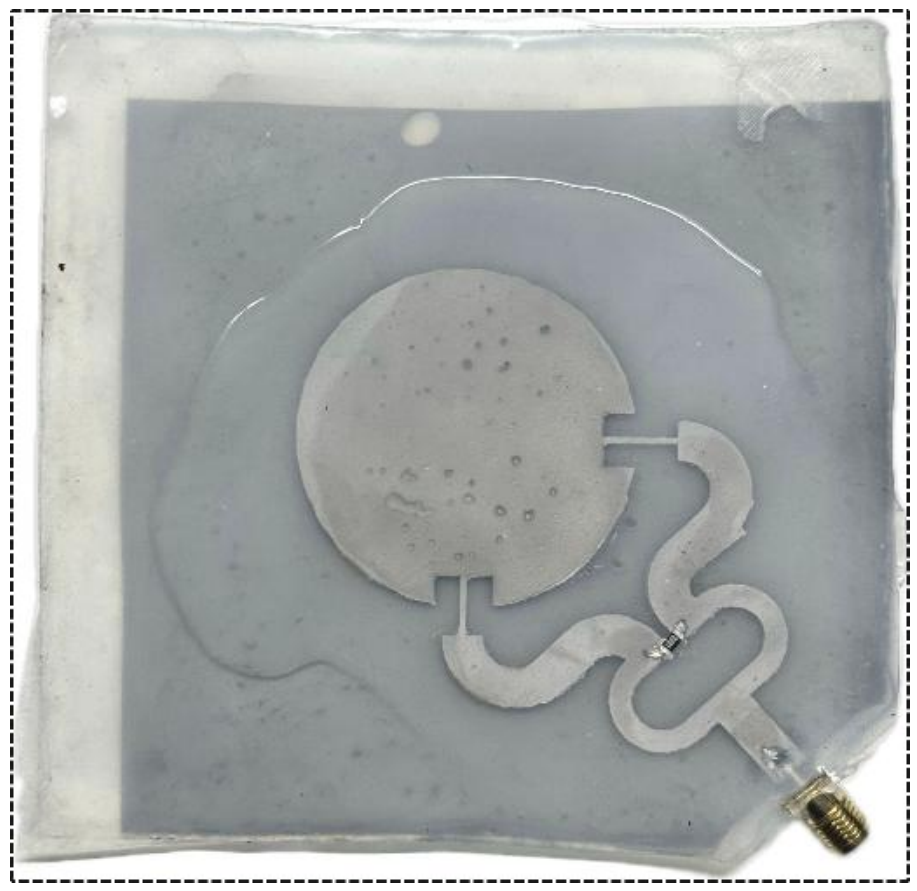

**Figure S33.** Photograph of a no-slot single-input antenna system with a power-divider-fed two-branch patch radiator.

*2. The manuscript and Supplementary Information appear to use the terms “SP-MSiA” and “IP-MSiA” interchangeably in several locations. To avoid confusion for readers, the authors should ensure that the terminology is consistent throughout the manuscript and that each acronym is clearly defined.*

**Our response:**

Thank you for pointing out this inconsistency. We have revised the manuscript and Supplementary Information throughout and now use the acronym S**P-MSiA** consistently.

**Our modification to the manuscript:**

**Figure 6. End-to-end wireless communication performance evaluation of SP-MSiA.** **(a)** (i) Experimental setup of the SP-MSiA integrated with a BLE-based wireless ECG monitoring system (BioBox M2) through an IPEX–SMA coaxial feedline.

**Figure 7. Real-world applications of SP-MSiA-based wearable electronics. (a)** (i) On-body experimental setup of the SP-MSiA integrated with a wireless ECG monitoring system on the right pectoral region. Photographs showing the antenna under (ii) hand-raising ($\varepsilon_y \approx 30\%$) and (iii) chest-opening ($\varepsilon_x \approx 30\%$) motions. **(b)** Comparison of the wireless ECG signals recorded by the systems based on SP-MSiA, single-port slot-loaded antenna, and reference antenna before and during hand-raising and chest-opening movements.

**Figure S26.** Simulated surface current distribution of SP-MSiA at **(a)** 2.40 GHz (Mode 1, major-axis-dominant) and **(b)** 2.45 GHz (Mode 2, minor-axis-dominant).

*3. Several figures would benefit from improved visual clarity, particularly in terms of color contrast, line thickness, and consistency between legend labels and plotted curves. Improving these graphical elements would make the results easier to interpret and better highlight the key comparisons presented in the manuscript.*

**Our response:**

We thank you for this helpful suggestion. We agree that improved visual clarity would enhance the readability of the figures. In the revised manuscript, we have improved the relevant figures by refining the color contrast, increasing line thickness where appropriate, and ensuring consistency between legend labels and plotted curves.

*4. The description of prior work in the Introduction appears to have inconsistencies in the explanations of References 16 and 17. The current text suggests that the roles or characteristics of these references may have been interchanged or inaccurately summarized. The authors should carefully re-check the descriptions of Refs. 16 and 17 and revise the corresponding sentences to ensure that each reference is accurately represented.*

**Our response:**

Thank you for pointing out this issue. We have corrected the order of Refs. 16 and 17 in the revised manuscript and also checked the other references in this section to ensure accurate citation and description.

**Our modification to the manuscript:**

Refences:

[16] O'Neill, M. R., et al. Dielectric Elastomer Architectures with Strain-Tunable Permittivity. ***Advanced Materials Technologies***, (2022), *7*, 11: 2200296. 10.1002/admt.202200296

[17] Kim, S. H., et al. (2024). Strain-invariant stretchable radio-frequency electronics. ***Nature***, (2024), 629, 8014: 1047-1054. 10.1038/s41586-024-07383-3

***Reviewer 2's comments and authors' responses***

*This manuscript reports a multidirectional strain-insensitive stretchable RF antenna system based on a symmetry-guided slot design and dual-port excitation strategy. By leveraging a characteristic-mode-based mechano-electromagnetic framework, the authors achieve near-constant resonance under large in-plane tensile strains across various orientations. The proposed DP-MSiA maintains a frequency shift of ≤20 MHz at 2.45 GHz under up to 45% strain, and is further integrated into wireless energy harvesting and on-body communication systems. The antenna demonstrates stable power transfer and ECG data transmission during body motions for wearable RF electronics. Here are a few concerns listed below.*

**Our response:**

We sincerely thank you for the helpful and insightful comments. We appreciate the reviewer's recognition of the novelty and significance of our multidirectional strain-insensitive stretchable RF antenna system, including the symmetry-guided slot design, dual-port excitation strategy, and characteristic-mode-based mechano-electromagnetic framework. We have carefully considered all of the concerns raised and revised the manuscript accordingly. Detailed point-by-point responses are provided below.

*1. The study claims "multidirectional" or "360° strain-insensitive" behavior. Does this strictly apply to uniaxial strain only, or to all deformation states including biaxial stretching?*

**Our response**:

Thanks for your valuable comments and for the opportunity to further clarify the scope of the term "multidirectional" or "360° strain-insensitive" behavior used in our manuscript. We agree that this point should be stated more explicitly. In our work, this description primarily refers to the antenna response under uniaxial stretching applied along arbitrary in-plane directions, rather than implying complete invariance under all possible deformation states.

We evaluated the device under biaxial strain. Under 30% biaxial stretching with intersection angles of 180°, 150°, 120°, and 90° between the two stretching directions, the SP-MSiA exhibits resonance shifts of 7.5, 27.5, 46.9, and 221.1 MHz, respectively. These values are all smaller than those of the conventional antenna under the same conditions (270.6, 88.0, 136.4, and 244.2 MHz). The biaxial-strain comparison is illustrated in **Figure S39**. As the intersection angle decreases to 90°, the deformation approaches a near-isotropic

stretching condition, under which the slot-patch compensation becomes less effective because the overall patch size increases and the resonance frequency decreases. However, such a condition is more stringent than most practical wearable deformation states, where biaxial strains are generally unequal in magnitude and not orthogonal. Therefore, we have further clarified this point in the revised manuscript.

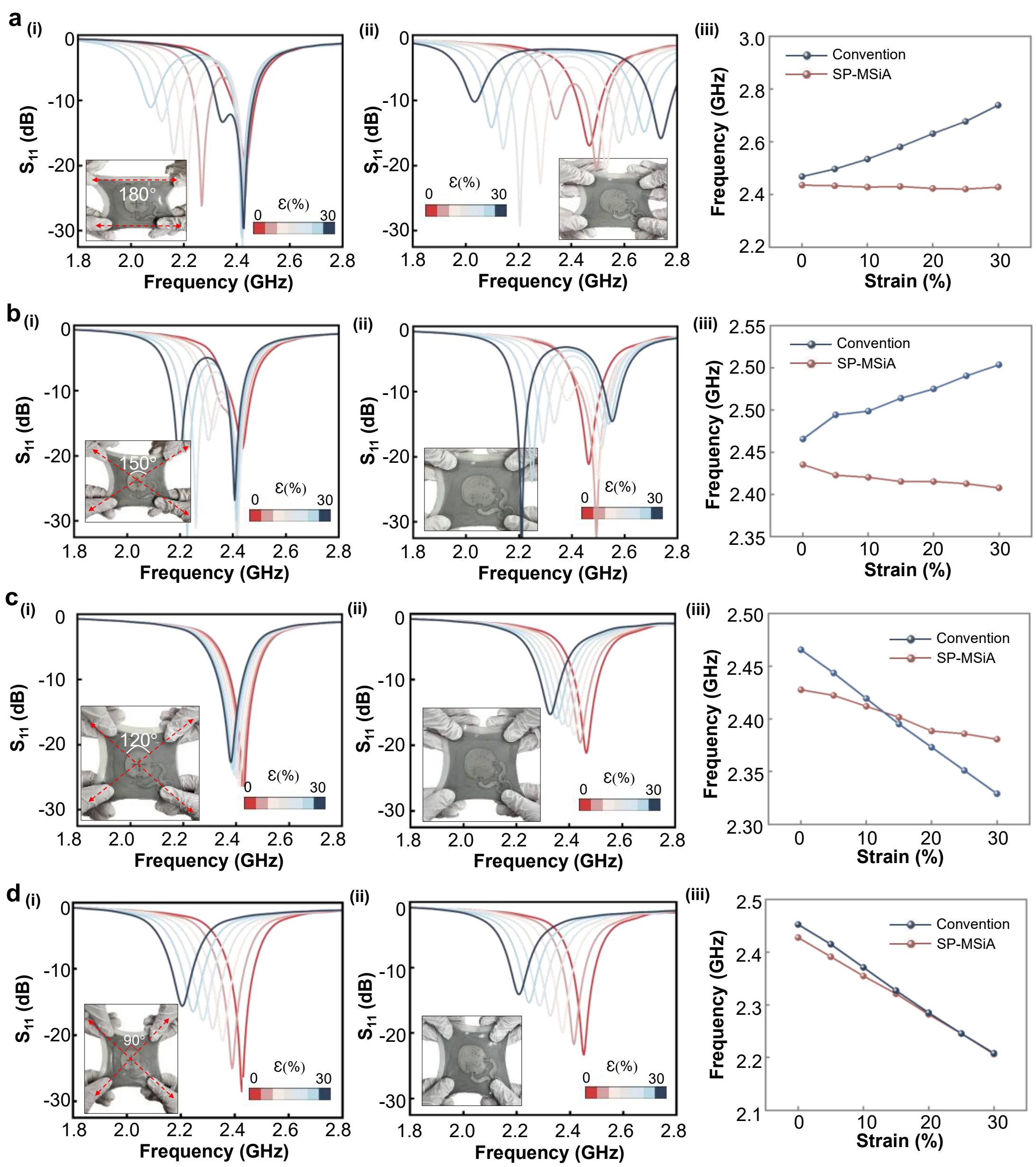


**Figure S39.** Measured $S_{11}$ of the (i) SP-MSiA and (ii) reference conventional antenna under dual biaxial tensile strain of 0, 5, 10, 15, 20, 25, and 30% with four representative inter-axis angles of **(a)** 180° (parallel), **(b)** 150°, **(c)** 120°, and **(d)** 90°, along with (iii) the corresponding extracted resonance frequency. The insets show photographs of the SP-MSiA and the reference conventional antenna

under biaxial strain.

**Our modification to the manuscript:**

"…… Nonetheless, the substantially enhanced communication stability of the SP-MSiA under multidirectional stretching paves the way for the practical implementation of stretchable antennas in wearable electronics.

To further evaluate practical wearable conditions, we also examine biaxial stretching, out-of-plane bending, and on-body loading. Under 30% biaxial strain with intersection angles (i.e., the angle between the two applied biaxial stretching directions) of 180°, 150°, 120°, and 90°, the SP-MSiA exhibits resonance shifts of 7.5, 27.5, 46.9, and 221.1 MHz, respectively, which are smaller than those of the reference antenna (270.6, 88.0, 136.4, and 244.2 MHz) (**Figure S39**). However, as the intersection angle decreases to 90°, the frequency-stability advantage of the SP-MSiA diminishes because the slot-patch compensation becomes less effective as the patch is stretched near isotropically, increasing the overall patch size and decreasing the resonance frequency. Such a condition is more stringent than most practical wearable deformation states, where biaxial strains are generally unequal in magnitude and not orthogonal. Moreover, measurements under out-of-plane bending conditions show only slight changes in resonance frequency and $S_{11}$ (**Figure S40**). These results indicate that the proposed antenna maintains stable impedance behavior under representative wearable deformation scenarios. ……"

*2. The slot-enabled compensation appears optimized at a specific strain level (e.g., 30%). Is the strain-invariant condition theoretically valid across the entire strain range (0–45%), or only near the design point?*

**Our response**:

Thank you for this important comment. We agree that the applicable strain range of the strain-invariant condition should be clarified more quantitatively. Although the strain-invariant condition was derived based on a first-order approximation, the second-order analysis in **Note S3** indicates that the residual error remains small over the strain range considered. To make this point clearer, we have added representative numerical validation in the revised **Supplementary Information** by directly evaluating the approximation error at selected strain levels (10%, 20%, 30%, 40%, and 45%).

To assess the validity range of the strain-invariant condition, we further considered the second-order geometric expansion under uniaxial stretching. Under uniaxial stretching along the $x$-direction, the second-order expansion gives:

$$\frac{|\Delta L|}{L_{eff}(0)} \approx \varepsilon_x^2 \frac{|k_D D_0 + \alpha k_L L_{slot,0}|}{D_0 + \alpha L_{slot,0}}.$$

For an incompressible elastomer, $\nu = 1/2$, $k_D = 3/8$, and $k_L = 0$. Using the first-order strain-invariant condition $L_{slot,0} = \nu D_0/\alpha$ (derived in the undeformed state $\varepsilon_x = 0$), this simplifies to

$$\frac{|\Delta L|}{L_{eff}(0)} \approx \frac{1}{4}\varepsilon_x^2.$$

Accordingly, we now explicitly quantify the approximation error at representative strain levels. By substituting $\varepsilon_x$=0.10, 0.20, 0.30, 0.40, and 0.45, the estimated second-order error is approximately 0.25%, 1.0%, 2.25%, 4.0%, and 5.1%, respectively. Since the resonant frequency scales approximately as $f \propto L_{eff}^{-1}$, these results indicate that the deviation from the first-order strain-invariant condition remains small throughout the experimentally studied range. We have revised this part accordingly and added these representative validations in **Note S3**.

**Our modification to the manuscript:**

**"Note S3: Validity of the linear approximation: second-order effects:**

The derivations above are based on small-strain linearization. As the applied uniaxial strain increases, geometric nonlinearity becomes more significant. To assess the validity of the linear approximation, general second-order expansions of the geometric change are introduced [12]:

$$D(\varepsilon_x) \approx D_0(1 - \nu\varepsilon_x + k_D\varepsilon_x^2), \tag{3.10}$$

$$L_{slot}(\varepsilon_x) \approx L_{slot,0}(1 + \varepsilon_x + k_L\varepsilon_x^2), \tag{3.11}$$

where $k_D$ and $k_L$ are dimensionless coefficients capturing geometric and material nonlinearity. Substituting into Eq. (3.4) gives:

$$L_{eff}^{(2)}(\varepsilon_x) \approx \frac{\pi}{2X_{11}}\left[D_0 + \alpha L_{slot,0} + \varepsilon_x\left(\alpha L_{slot,0} - \nu D_0\right) + \varepsilon_x^2\left(k_D D_0 + \alpha k_L L_{slot,0}\right)\right]. \tag{3.12}$$

Thus, the relative error in the total electrical length when using the linear approximation as in Eq. (3.4) is then:

$$\frac{|\Delta L|}{L_{\mathrm{eff}}(0)} \approx \frac{L_{\mathrm{eff}}^{(2)}(\varepsilon_x)\text{-}L_{\mathrm{eff}}^{(1)}(\varepsilon_x)}{L(0)} = \varepsilon_x^2 \frac{|k_{\mathrm{D}}D_0+\alpha k_{\mathrm{L}}L_{\mathrm{slot,0}}|}{D_0+\alpha L_{\mathrm{slot,0}}}. \tag{3.13}$$

For an ideal incompressible isotropic block under uniaxial tension, the standard kinematics give $v = 1/2$ and $k_{\mathrm{D}} = 3/8$ for the lateral contraction [12][15], while the axial stretching is exactly $1+\varepsilon_x$, so $k_{\mathrm{L}}=0$ [12][15]. For a tensile strain of 30%, $\varepsilon_x=0.3$ and thus $\varepsilon_x^2=0.09$, and the resulting relative error is on the order of 3% when $D_0$ and $L_{\mathrm{slot,0}}$ are of similar magnitude. Thus, the second-order correction remains only a few percent even at 30% stretching, indicating that the linear approximation is sufficiently accurate for the strain range considered in this work. By substituting $\varepsilon_x$=0.10, 0.20, 0.30, 0.40, and 0.45, the estimated second-order error is approximately 0.25%, 1.0%, 2.25%, 4.0%, and 5.1%, respectively. Since the resonant frequency scales approximately as $f \propto \mathrm{L}_{\mathrm{eff}}^{-1}$, these results indicate that the deviation from the first-order strain-invariant condition remains small throughout the experimentally studied range. Thus, although the strain-invariant condition is formally derived at the undeformed state through first-order linearization, the second-order correction remains only a few percent even at 45% stretching, indicating that this condition remains a reliable approximation across the full experimental strain range considered in this work.

*3. The study emphasizes preserved radiation performance. Could the authors provide absolute radiation efficiency values under representative strain levels to quantitatively support this claim?*

**Our response**:

Thank you for this important comment. We agree that providing absolute radiation efficiency values at representative strain levels would more quantitatively support our statement regarding preserved radiation performance. Accordingly, we have added the absolute radiation efficiency values in the revised manuscript as a complementary metric to realized gain.

We emphasize preserved radiation performance because the present slot-loaded design maintains strain insensitivity without strongly compromising the radiating aperture, unlike many previously reported strain-insensitive antenna designs based on excessive serpentine or meandered conductor layouts[1], which push the antenna into an electrically small regime and substantially degrade radiation efficiency due to excessive miniaturization[2]. For example, previous approach in Ref.1 showed a substantial reduction in radiation efficiency compared

with conventional patch-type designs, with values decreasing from approximately −-7 dB to -13 dB even in the undeformed state in order to achieve strain insensitivity. In contrast, in the present work, the proposed SP-MSiA achieves multidirectional strain insensitivity while maintaining an absolute radiation efficiency of approximately -4.55 dB in the undeformed state, which is close to that of the corresponding conventional-size solid no-slot patch antenna (approximately -4.3 dB).

Specifically, the absolute radiation efficiency of the proposed SP-MSiA is -4.55 dB in the undeformed state, -4.78 dB at 15% strain, -5.05 dB at 30% strain, and -5.2 dB at 45% for the *x*-axis strain. These results show that the absolute radiation efficiency remains relatively stable under representative strain levels, thereby quantitatively supporting our claim of preserved radiation performance. Thus, we have added this clarification to the revised manuscript, with the corresponding radiation-efficiency values at representative strain levels provided in **Figures S37** and **S38**.

References:

[1] Kim S H, Basir A, Avila R, et al. Strain-invariant stretchable radio-frequency electronics[J]. ***Nature***, 2024, 629(8014): 1047-1054.

[2] Zhang S, Yang F, Song C, Cheng H. Material strategy for strain-invariant radio-frequency electronics[J]. ***International Journal of Extreme Manufacturing***, 2026, 8:041503

**Our modification to the manuscript:**

"……Moreover, the realized gain of -5.6 dBi from the SP-MSiA under stretching along the strain-sensitive 135° direction is also higher than that of -9.6 dBi from the conventional counterpart due to its smaller resonance-frequency shift. As a complementary metric to realized gain, the absolute radiation efficiency of the SP-MSiA in the undeformed state is -4.55 dB, which is close to that of the conventional no-slot patch antenna (-4.3 dB), and remains stable under representative strain levels (**Figure S37**). A similar trend is also observed for the circular-hole strain-invariant design (**Figure S38**). Specifically, the circular-hole antenna maintains good strain-insensitive resonance behavior under stretching along the *x*-, 45°-, and *y*-directions, but still exhibits a pronounced frequency shift under stretching along the 135° direction. However, despite this partial directional strain tolerance, its maximum realized gain remains much lower than that of the cross-slot SP-MSiA in all cases (**Figure 5f**), owing to its lower radiation efficiency. Therefore, the cross-slot design provides a more favorable overall trade-off between directional strain robustness and radiation performance.

Nonetheless, the substantially enhanced communication stability of the SP-MSiA under multidirectional stretching paves the way for the practical implementation of stretchable antennas in wearable electronics. ……”

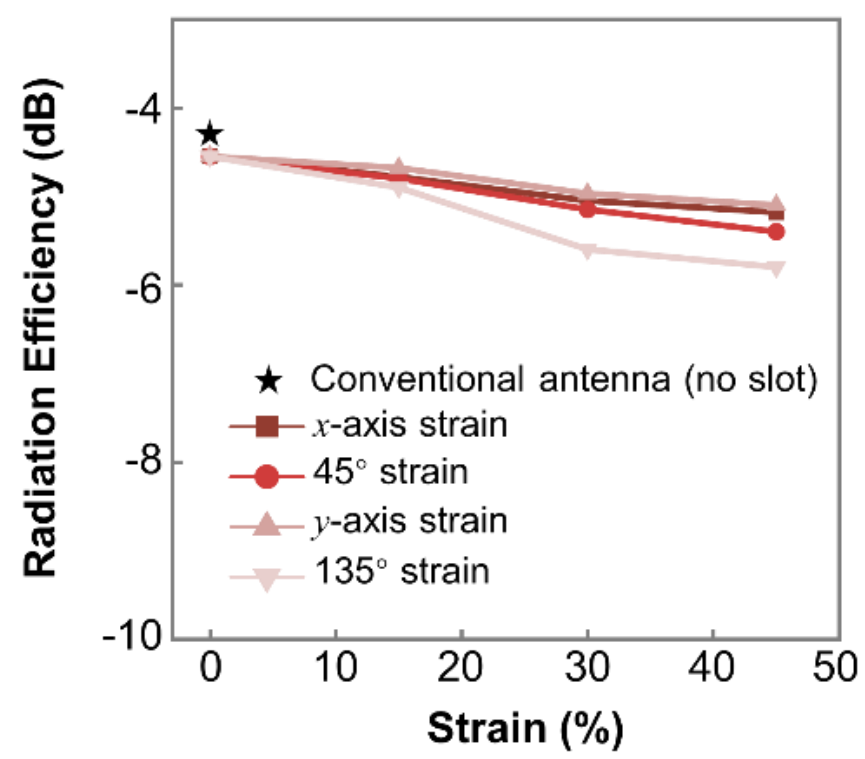


**Figure S37.** Absolute radiation efficiency of the SP-MSiA as a function of strain level under four representative in-plane stretching directions (*x*, 45°, *y*, and 135°).

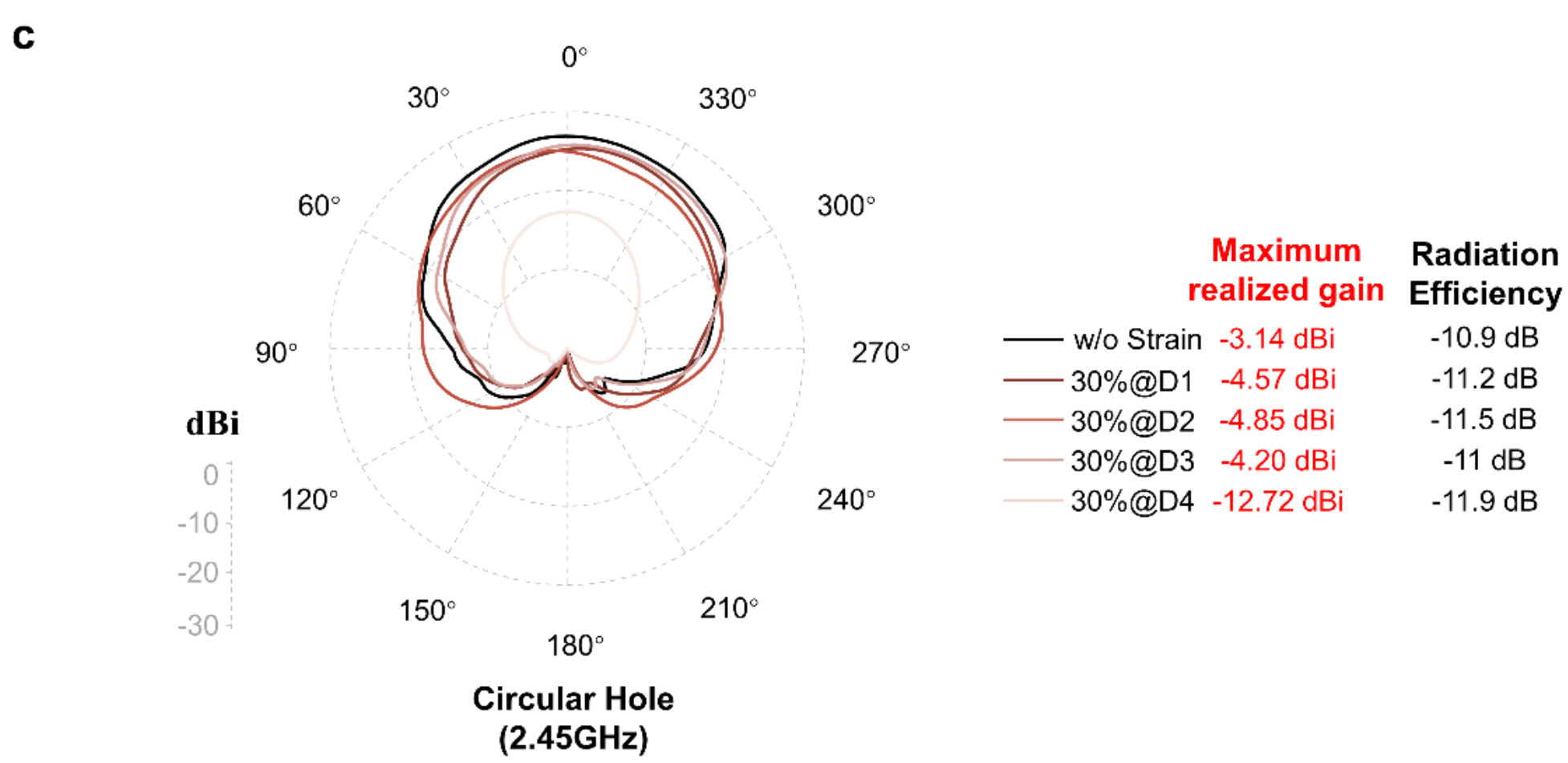


**Figure S38. (c)** Measured radiation patterns and maximum realized gain of the circular-hole strain-invariant antenna in the unstrained state and under 30% strain applied along the *x*, 45°, *y*, and 135° directions.

*4. Is the dual-port architecture fundamentally required for multidirectional strain insensitivity, or could a properly designed single-feed antenna achieve comparable robustness?*

**Our response**:

Thanks for your valueble comment. We agree that it is important to clarify whether the dual-port architecture is fundamentally required for achieving the multidirectional strain insensitivity reported here.

From the perspective of modal excitation theory, the coupling strength between a feed and a given resonant mode is determined by the overlap between the feed excitation and the modal current distribution. For a single-feed antenna, a mode whose current distribution is orthogonal or nearly orthogonal to the feed direction will naturally exhibit weak coupling and therefore cannot be efficiently excited. In the present problem, multidirectional strain insensitivity requires effective excitation of multiple strain-insensitive modes associated with different stretching directions, which imposes an intrinsic limitation on a single-feed configuration.

We did examine whether a properly designed single-feed antenna could provide comparable robustness, including different inset- and offset-feed microstrip arrangements, since such a design would considerably simplify the structure. As shown in **Figure S10a**, the microstrip feed was laterally shifted by an offset $W_{offset}$ to improve coupling to the strain-insensitive mode under $y$-axis stretching. However, as illustrated schematically in **Figure S10b**, this mode remains orthogonal or nearly orthogonal to the single-feed excitation, so the coupling remains intrinsically weak.

This limitation is confirmed by the simulated responses in **Figure S10cd**. For no offset or a small offset ($W_{offset}$ = 4 mm), the strain-insensitive mode is not effectively excited, and almost no distinct resonance associated with this mode appears (**Figure S10c**). When the offset is increased to a larger value ($W_{offset}$ = 10 mm), a weak resonance begins to emerge (**Figure S10d**), indicating partial improvement in coupling. However, the coupling remains too weak for robust excitation. More importantly, robust excitation is obtained only when the strain angle is reduced from 90° to 45°, as shown in **Figure S10ef**. This indicates that even a feed offset that improves coupling to more modes can access only a limited angular range, rather than providing true multidirectional robustness.

Therefore, although introducing a finite feed offset can improve access to the strain-insensitive mode over a limited range of strain angles, a single-feed antenna still cannot efficiently excite all of the relevant strain-insensitive modal responses, particularly when the

strain direction lies approximately between 45° and 135°. In other words, the limitation does not simply arise from insufficient geometric or feed optimization, but from the intrinsic modal-coupling constraint of single-feed excitation. For this reason, the dual-port architecture is fundamentally required in the present design to efficiently excite the relevant strain-insensitive modes and thereby achieve broad multidirectional strain insensitivity.

Alternative feeding strategies (e.g., coaxial back-feeding) could, in principle, provide another excitation pathway. However, they are less suitable for wearable and stretchable applications because they introduce rigid out-of-plane interconnects and mechanical constraints that are unfavorable for soft, conformal, and deformable systems.

In summary, we have clarified this point in the revised manuscript and added the corresponding single-feed comparison.

**Our modification to the manuscript:**

**"……**

**2.2 Design and mechanical-electromagnetic characterization of DP-MSiA.**

To achieve multidirectional strain-insensitivity, the strain-insensitive mode (Mode 2) needs to be effectively excited as the strain direction varies. A single feed can efficiently excite only a subset of the desired strain-insensitive modal current distributions as the desired resonance becomes weak when the modal current distribution has a large angular mismatch with the feed line (**Figure S10**). This limitation is addressed by implementing a dual-port multidirectional strain-insensitive antenna (DP-MSiA) with two orthogonal ports (**Figure 3**), forming the minimum set of in-plane coupling channels needed to excite Mode 2 as the applied tensile strain direction varies.

**……"**

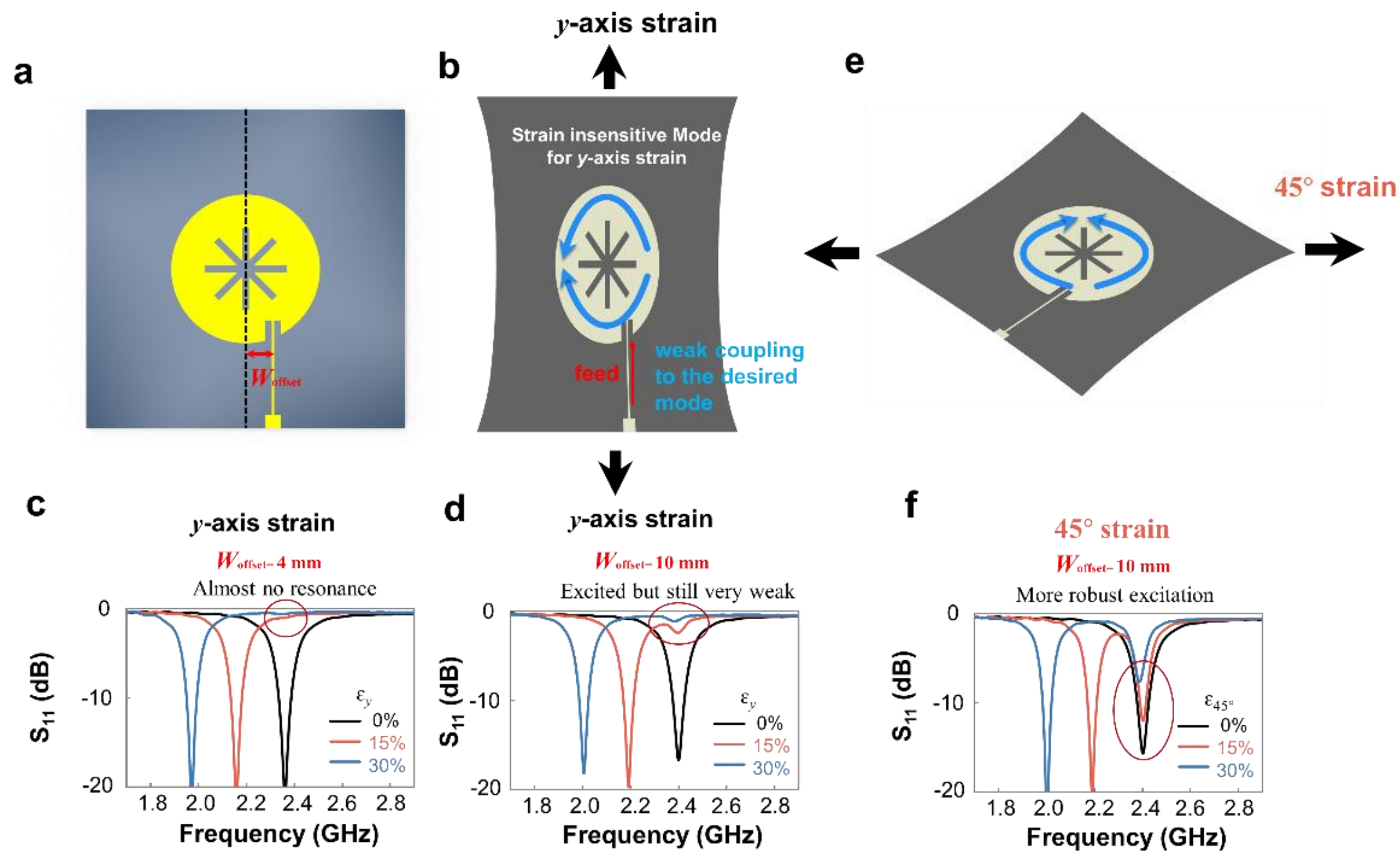


**Figure S10. Limitation of single-feed excitation for multidirectional strain-insensitive operation.** **(a)** Offset-fed single-port antenna design with feed offset ($W_{offset}$). **(b)** Schematic showing weak coupling of the strain-insensitive mode under *y*-axis stretching because it is orthogonal or nearly orthogonal to the feed direction. Simulated reflection responses showing that the desired mode is absent for **(c)** a small offset, only weakly excited for **(d)** a large offset under *y*-direction strain. **(e)** Schematic of the 45° stretching direction. **(f)** Simulated reflection responses for $W_{offset}$ =10 mm under 45°-direction strain, where the desired mode becomes more clearly excited.

*5. The improved communication stability is demonstrated experimentally. Could the authors clarify whether this improvement mainly results from reduced frequency detuning, or radiation-pattern stability?*

**Our response**:

Thank you for this constructive comment. We agree that the physical origin of the improved communication stability should be clarified more explicitly. In the present case, the improvement arises primarily from the reduced strain-induced frequency detuning of the proposed antenna, rather than from a major stabilization of the radiation-pattern shape itself.

To clarify this point, we normalized the original radiation-pattern data in **Figure S36** under representative strain conditions and compared them with those of the reference antenna.

As shown in **Figure R2.2** (derived from the absolute realized gain data in **Figure S36**), the normalized radiation patterns of both the SP-MSiA and the conventional antenna remain broadly similar under strain, indicating that the radiation-pattern shape does not change significantly and is not the main factor responsible for the difference in communication stability.

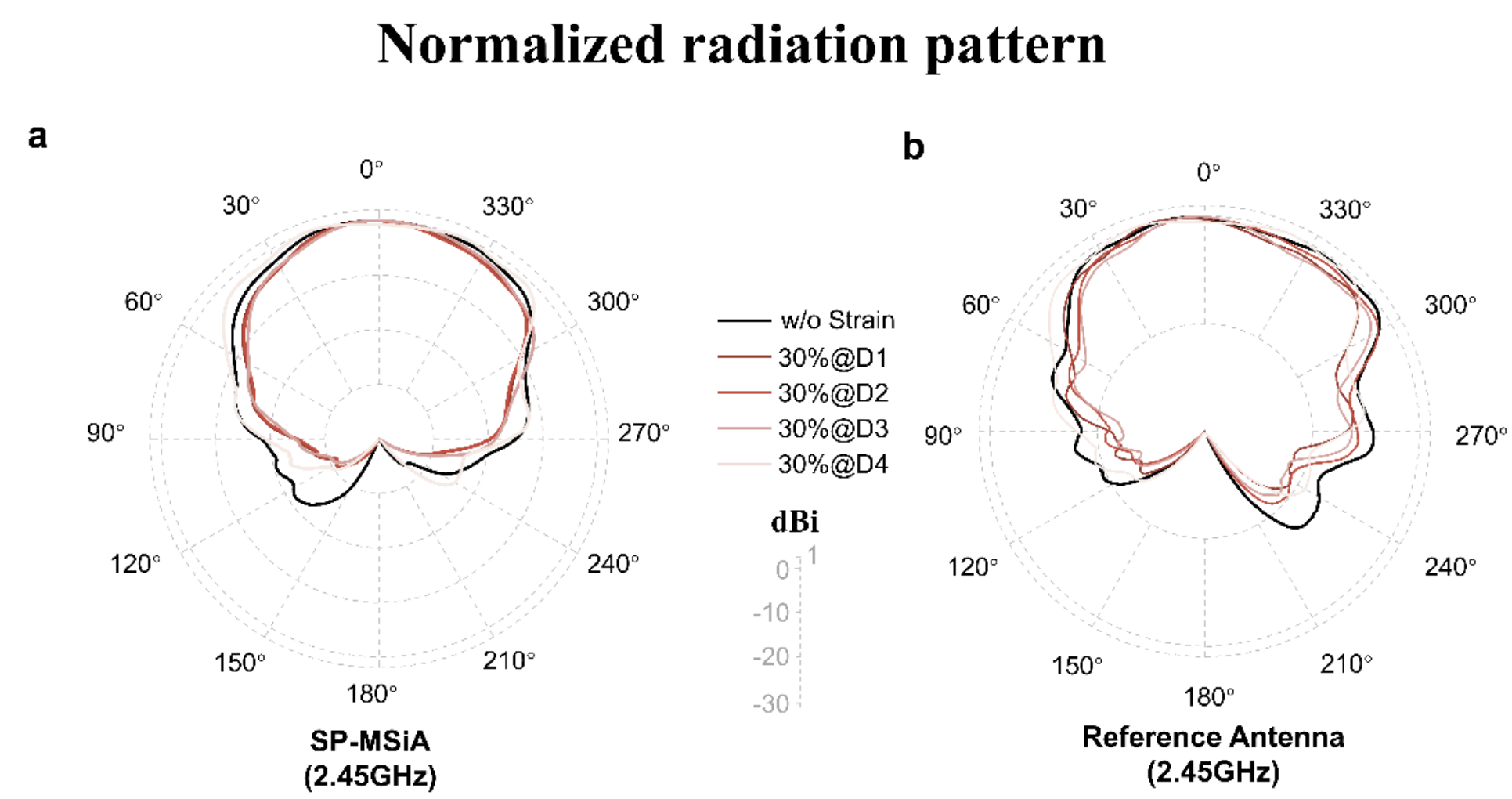


**Figure R2.2**. Normalized radiation patterns of the **(a)** SP-MSiA and **(b)** reference antenna under strain (generated by normalizing the original radiation-pattern data in **Figure S36**)

Instead, the improved communication stability mainly originates from the smaller strain-induced frequency detuning of the SP-MSiA. Because the SP-MSiA maintains its resonance closer to the original operating frequency under deformation, it preserves better impedance matching and higher total efficiency, which in turn maintains a more stable maximum realized gain at boresight. Thus, we have clarified this point in the revised manuscript.

**Our modification to the manuscript:**

"······Although the SP-MSiA remains strain-sensitive under 40% stretching along the 135° direction, its resonance shift of ~0.50 GHz is significantly smaller than that of ~0.65 GHz from the unslotted reference, due to the geometric self-compensation discussed in the previous sections. Both the SP-MSiA and the unslotted reference antenna maintain broadly similar radiation patterns under strain. However, the SP-MSiA exhibits much higher maximum realized gains of 2.8, 1.46, and 2.89 dBi under 30% stretching along the $x$, 45°, and $y$ directions, which are significantly higher than those (-6.4, -7.6, and -6.3 dBi) provided by the conventional counterpart (**Figures 5f** and **S36**). ······"

***<u>Reviewer 3's comments and authors' responses</u>***

*This manuscript presents a DP-MSiA and SP-MSiA designed to minimize the frequency shift and maximize the radiation performance under multidirectional mechanical in-plane strain. The authors comprehensively analyzed the effect of antenna geometry on the signal performance under the mechanical deformation in multi-directions, such as the presence of slots, the number of slots, slot geometry and antenna shapes. Additionally, wireless communication and ECG monitoring were demonstrated as proof-of-concept, showing the outstanding performance compared to the conventional antenna. While the overall originality and experimental validations are outstanding, additional discussions are needed before publication.*

**Our response:**

We sincerely thank the reviewer for the positive and encouraging comments on the originality, systematic analysis, and experimental validation of this work. We also appreciate the reviewer's constructive suggestion regarding the need for additional discussion. In response, we have further revised the manuscript to include additional clarifications and discussions to improve the completeness and clarity of the presentation.

*1. This antenna is intended for wearable devices mounted onto the human body. The authors should show the effect of human body tissue on the antenna's performance even though there is a ground layer.*

**Our response**:

Thank you very much for your valuable comment. We agree that the effect of the human body should be clarified. To address this point, we further evaluated the proposed antenna both by simulation using a human-tissue-material phantom and by measurement when mounted on human tissue.

The simulated reflection coefficient results in **Figures S42a-i** and **ii** show that the antenna performance under on-tissue conditions is very close to that in free space. The simulated on-body $S_{11}$ curves exhibit only a slight shift compared with the free-space case, while the impedance matching remains well preserved around the operating band. The main reason is that the proposed antenna employs a ground plane, which effectively isolates the radiating element from the lossy biological tissues and suppresses strong near-field coupling to the body. As a result, the body loading effect on the antenna impedance is significantly reduced. Furthermore, the simulated realized gain patterns shown in **Figure S42a-iii** demonstrate that

the proposed antenna maintains a radiation behavior on the body that is very similar to that in free space. The free-space and on-body patterns have comparable shapes, and the gain degradation is small. This confirms that the presence of the human body does not significantly distort the antenna radiation characteristics.

In addition, to provide a more realistic assessment, the antenna was further evaluated using the "Gustav" human voxel model available in CST Studio Suite, as shown in **Figure S42b-i**. In this case, the antenna was placed on the chest region of the phantom to emulate a practical wearable scenario. The CST phantom is an anatomically realistic heterogeneous body model, which provides a more representative evaluation of body-loading effects than the simplified layered tissue model. As shown in **Figure S42b-ii**, the simulated reflection coefficient under on-phantom conditions also remains very close to that in free space, with only a slight shift in resonance frequency. Moreover, the realized gain pattern in **Figure S42b-iii** is only slightly affected. These results indicate that body proximity introduces only a minimal influence on the proposed antenna performance.

Finally, we have added additional measurements of the reflection coefficient ($S_{11}$) of the communication antenna under several representative wearable conditions (e.g., placed on the forearm, calf, abdomen, and garment, with photographs shown in **Figure S42c-i**), with the corresponding $S_{11}$ responses provided in **Figure S42c-ii**. The measured $S_{11}$ responses are very similar to those in air, with nearly unchanged resonance frequencies and no obvious deterioration in impedance matching. These results indicate that body proximity introduces minimal degradation to the antenna performance, which is attributable to the sufficiently large ground plane that provides effective isolation from the lossy biological tissue.

**Our modifications to the manuscript:**

"……

**2.5 End-to-end wireless communication performance through SP-MSiA**

Unlike the free-space WEH validation, the BLE communication study is conducted in a wearable configuration by mounting the SP-MSiA on a smart T-shirt, thereby enabling direct evaluation of communication robustness under practical on-body deformation. To assess the on-body safety of the proposed antenna for wearable operation, SAR analysis is conducted to confirm low SAR values, consistent with the effective shielding provided by the ground plane (**Figure S41**). Furthermore, both simulations (**Figure S42ab**) and wearable measurements (**Figure S42c**) confirm that the proposed antenna is minimally affected by body loading,

because the ground plane effectively shields the radiator from lossy biological tissue, suppresses near-field coupling, and thereby preserves the resonance and impedance matching under practical wearable conditions. With a thin stretchable nanogel adhesive applied to the antenna ground plane, the SP-MSiA is bonded to the right pectoral region of a smart T-shirt with embedded electrocardiogram (ECG) electrodes and stretchable interconnects. **……**"

"***Characterization of mechanical-electromagnetic properties:***

**……** Polarization alignment between the antenna under test and the horn was maintained for all measurements. The received signal was recorded at each angular position to obtain the radiation patterns under different strain conditions. The strain-dependent measurements of the proposed antenna are conducted in free space due to the small performance difference between free-space and wearable conditions. As confirmed by the additional on-tissue, on-phantom, and representative on-body/on-garment results **(Figure S42)**, body loading causes only slight resonance shifts and negligible degradation in impedance matching, mainly due to the shielding effect of the ground plane. Therefore, the free-space stretching measurements provide a reasonable and controlled approach for evaluating the intrinsic effect of mechanical deformation on the antenna response. **……**"

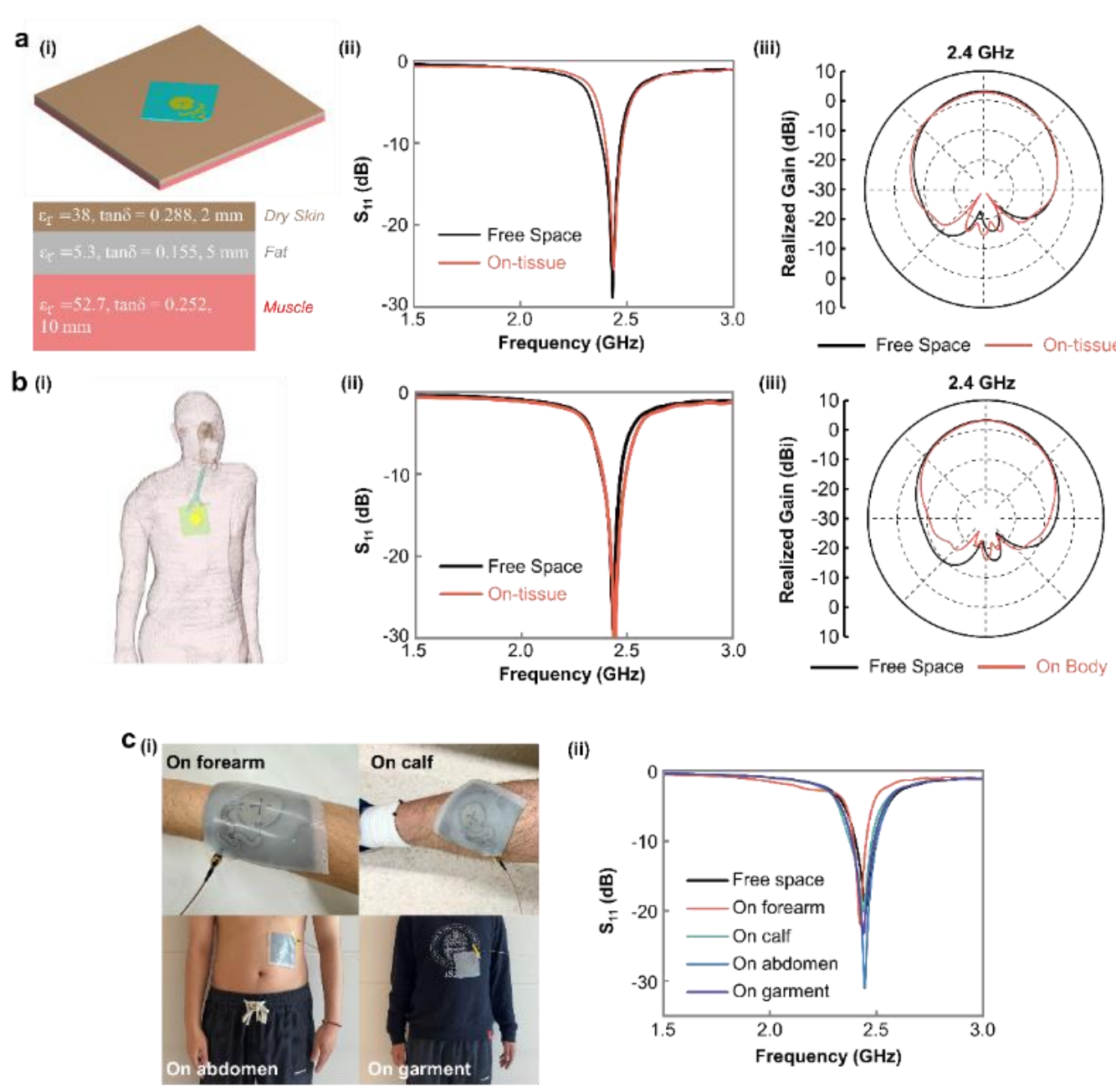


**Figure S15. Simulated and measured body-loading effects of the DP-MSiA. (a)** (i) Three-layer tissue model and simulated (ii) $S_{11}$ and (iii) realized gain patterns in free space and on tissue. **(b)** (i)

“Gustav” human voxel model and simulated (ii) $S_{11}$ and (iii) realized gain patterns in free space and on the body. **(c)** (i) Wearable configurations on the forearm, calf, abdomen, and garment, and (ii) corresponding measured $S_{11}$ responses.

*2. Since the antenna operates in RF, specific absorption rate (SAR) should be studied to evaluate human tissue safety.*

**Our response**:

Thank you very much for this important comment. We agree that the specific absorption rate (SAR) should be evaluated to assess the safety of the proposed antenna for on-body operation. In response, we have now conducted SAR analysis.

Firstly, as shown in **Figure S41a**, the SAR evaluation was carried out using a multilayer planar human-tissue phantom placed underneath the antenna. The phantom consists of 2 mm skin, 5 mm fat, and 10 mm muscle. The corresponding dielectric properties at 2.4 GHz were assigned using the standard human tissue parameters (**Figure S41a-i**), with the simulated SAR distributions shown in **Figure S41a-ii.** The simulated results show that the maximum 10 g averaged SAR is 0.252 W/kg, which is well below the commonly accepted safety limit of 2 W/kg averaged over 10 g of tissue.

Furthermore, to provide a more realistic safety assessment, we also performed SAR simulation using the “Gustav” human voxel model available in CST Studio Suite (**Figure S41b-i**). The antenna was placed on the right chest, consistent with the experimental configuration in **Figure 7a**. The voxel model represents an adult male with a height of 176 cm, a weight of 69 kg, and an age of 38 years. The simulated results (**Figure S41b-ii**) show a maximum 10 g averaged SAR of 0.774 W/kg, with absorption mainly concentrated in the tissue directly beneath and around the antenna. Although this value is higher than that obtained from the simplified multilayer phantom, it is still well below the safety limit of 2 W/kg. This increase is expected because the voxel model accounts for realistic body curvature, heterogeneous tissue distribution, and practical on-body placement, all of which can enhance local field concentration compared with the idealized planar phantom.

In summary, both the phantom-based and voxel-based simulations satisfy the 10 g averaged SAR safety requirement, with maximum values of **0.252 W/kg** and **0.774 W/kg**, respectively. This low SAR can be mainly attributed to the sufficiently large ground plane

in the proposed design, which effectively shields the human tissue from the radiating element and suppresses backward radiation toward the body. Consequently, only a limited portion of the electromagnetic energy penetrates into the tissue, resulting in low power absorption overall. It is also observed that the highest SAR value appears near the region where the ground plane is cut to accommodate the antenna feed, because the shielding effect is locally weakened there. Even so, the peak SAR remains very low and safely below the standard safety limit. Thus, this SAR safety information has been added to the revised manuscript to further support the safety of the proposed antenna for practical on-body applications.

**Our modifications to the manuscript:**

**"……**

**2.5 End-to-end wireless communication performance through SP-MSiA**

Unlike the free-space WEH validation, the BLE communication study is conducted in a wearable configuration by mounting the SP-MSiA on a smart T-shirt, thereby enabling direct evaluation of communication robustness under practical on-body deformation. To assess the on-body safety of the proposed antenna for wearable operation, SAR analysis is conducted to confirm low SAR values, consistent with the effective shielding provided by the ground plane **(Figure S41)**. Furthermore, both simulations (**Figure S42ab**) and wearable measurements (**Figure S42c**) confirm that the proposed antenna is minimally affected by body loading, because the ground plane effectively shields the radiator from lossy biological tissue, suppresses near-field coupling, and thereby preserves the resonance and impedance matching under practical wearable conditions. With a thin stretchable nanogel adhesive applied to the antenna ground plane, the SP-MSiA is bonded to the right pectoral region of a smart T-shirt with embedded electrocardiogram (ECG) electrodes and stretchable interconnects. **……"**

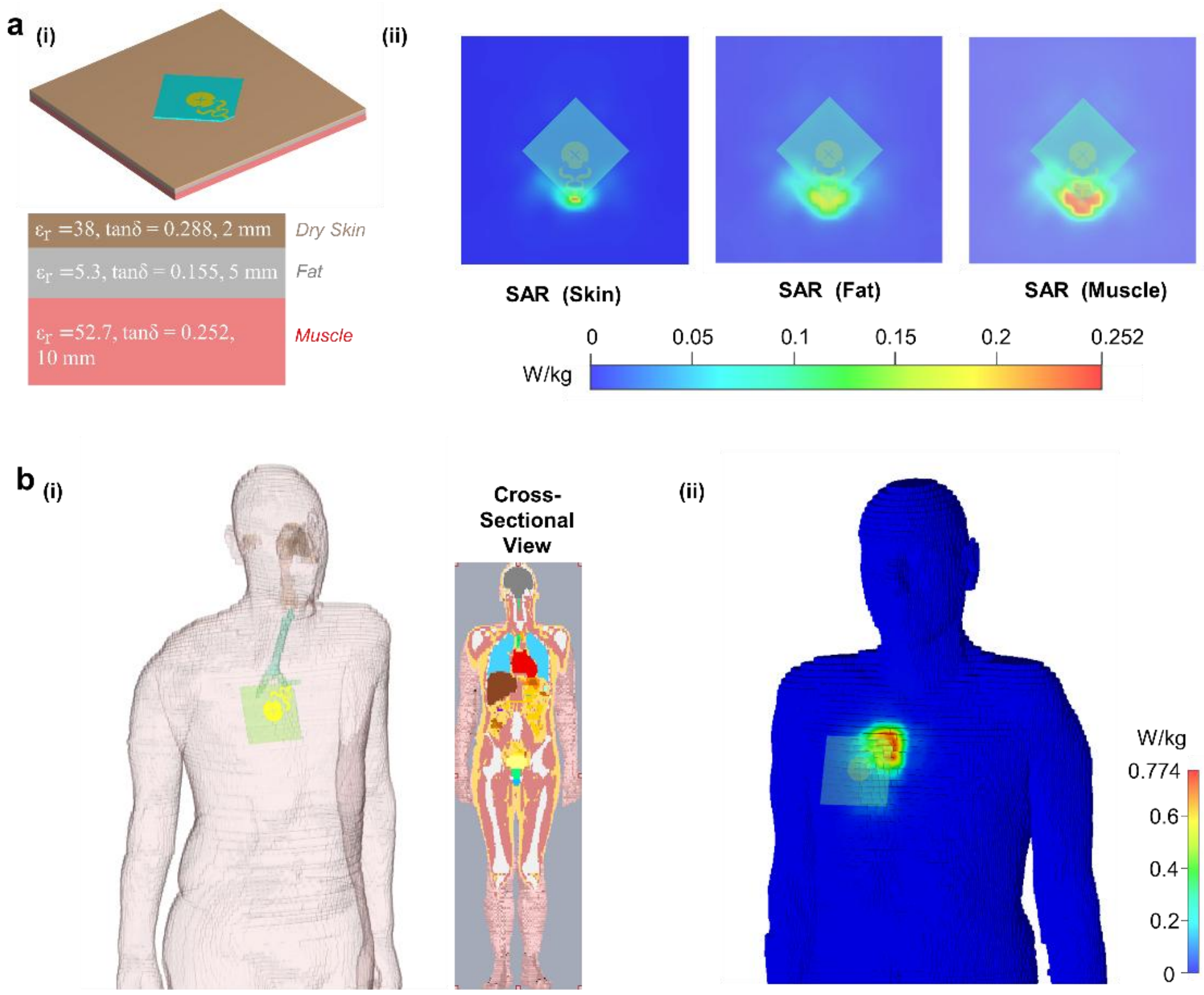


**Figure S41.** (i) Schematic model and (ii) simulated SAR distribution of the proposed SP-MSiA on **(a)** a simplified three-layer tissue model (consisting of dry skin, fat, and muscle) and **(b)** a realistic "Gustav" human voxel model.

*3. The authors should clearly show the microstructure of LMP layer with liquid metal particle size.*

**Our Response:**

Thank you for your helpful suggestion. In response, we have added a detailed characterization of the microstructure of the LMPs layer in the revised manuscript. Specifically, SEM images (**Figure S12a-ii**) are now included to clearly reveal the morphology and distribution of the liquid metal particles. In addition, a quantitative particle size distribution analysis has been provided (**Figure S12a-iii**), showing that the majority of particles fall within the 1-4 μm range. The corresponding description has been added in the revised manuscript to improve clarity and to provide deeper insight into the structural features of the LMP layer.

**Our modification to the manuscript:**

"**……** By combining Ecoflex cured in a mold and liquid metal particles (LMPs) via air spraying (**Figure S10**), the DP-MSiA features a thin Ecoflex substrate layer (~2 mm) sandwiched between the LMPs-based patch with a cross-shaped slot and a square ground layer (**Figure 3a**). It should be noted that, after ultrasonication, the liquid metal particles exhibit a size distribution predominantly in the range of 1~4 μm (**Figure S12a**). However, the transfer process induces partial rupture of the oxide shells, facilitating particle coalescence at the Ecoflex surface and the formation of electrically percolated networks within the LMPs-Ecoflex composites **(Figure S12b**). Moreover, the intrinsically stretchable LMPs patterned on Ecoflex also maintain stable high conductivity upon stretching of 300% (**Figure S11**) to serve as an effective near-perfect conductor for efficient electromagnetic radiation. **……**"

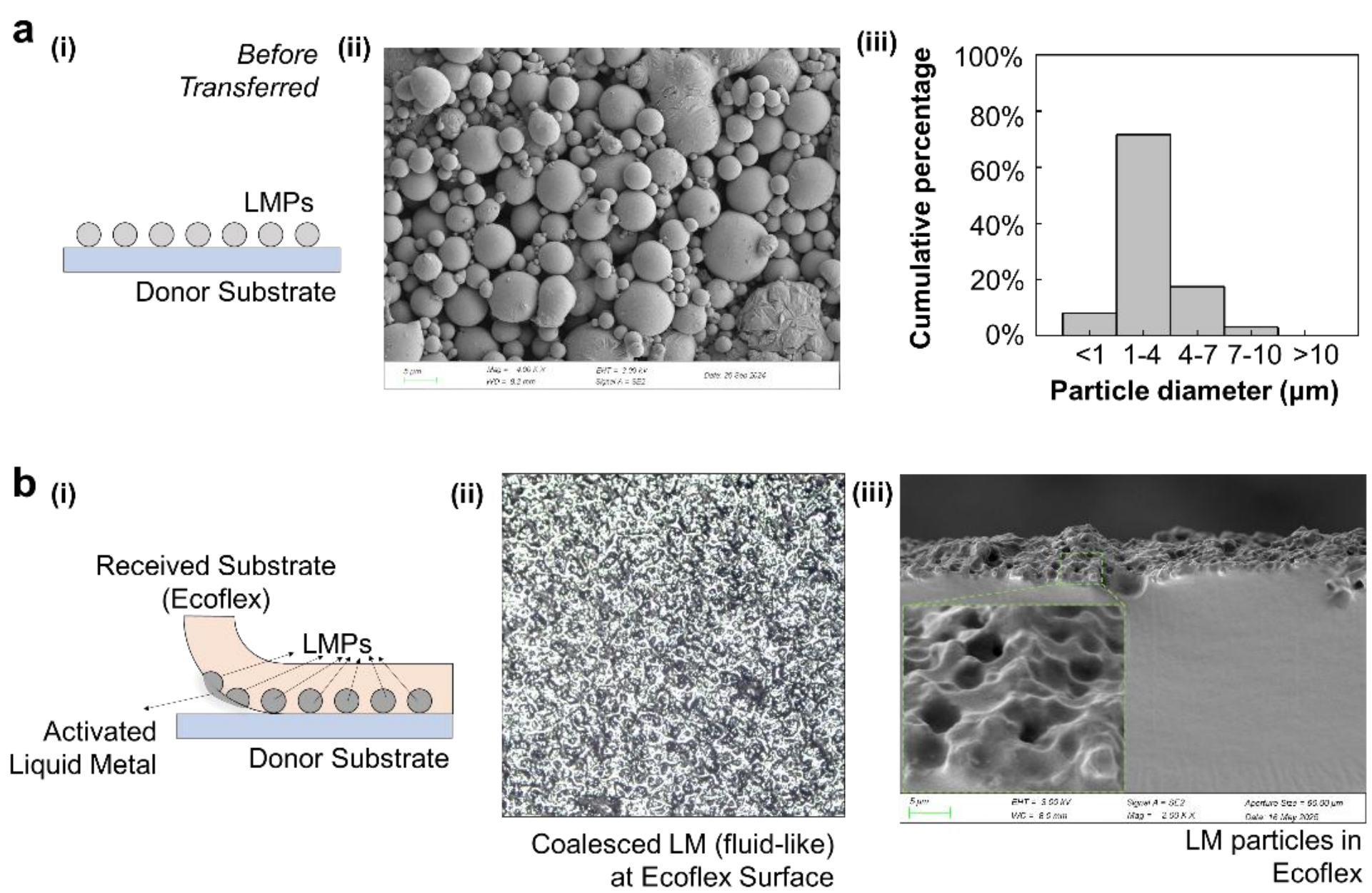


**Figure S12. Microstructure evolution of liquid metal particles (LMPs) before and after transfer. (a)** (i) Schematic illustration of LMPs on the donor substrate prior to transfer. (ii) SEM image and (iii) particle size distribution of LMPs after ultrasonication.

*4. The authors assume the electrical conductivity of the patch and ground layers as $\sigma = 3.6\times10^6$ S/m which is for bulk EGaIn, not for particles. Therefore, a proper conductivity value must be*

*considered in their simulations. Plus, please provide the unit "S/m" on the manuscript.*

**Our Response:**

Thank you for this important comment. We agree that the intrinsic electrical conductivity of isolated liquid metal particles is lower than that of bulk EGaIn due to the presence of the surface oxide layer. However, in our system, after the transfer process, the oxide shells of the liquid metal particles are mechanically disrupted, leading to particle coalescence and the formation of continuous conductive pathways at the elastomer surface. As shown in the optical image (**Figure S12b-ii**), the liquid metal exhibits a coalesced, fluid-like morphology. Furthermore, the SEM image of the Ecoflex surface after acid treatment (**Figure S12b-iii**) reveals interconnected liquid metal domains embedded within the substrate, confirming the formation of percolated conductive networks. This behavior is in good agreement with the prior report [1], which has demonstrated that oxide shell rupture and subsequent particle coalescence can lead to the formation of electrically percolated networks in LMPs-Ecoflex composites.

Therefore, the effective conductivity of the transferred LMP layer is substantially higher than that of isolated oxide-shell particles, because oxide-shell rupture and particle coalescence form percolated conductive pathways. Based on this physical mechanism, the conductivity value of $\sigma = 1\times10^5$ S/m was adopted as an approximation in the simulations. Previously, a conductivity of $\sigma = 3.6\times10^6$ S/m was used in the simulations. We have now updated this value to ~$10^5$ S/m based on experimental estimation (**Figure R3.1**). The revised simulations show negligible differences in the results, and the corresponding change has been updated in the Methods section. Meanwhile, we have revised the manuscript to explicitly include the unit "S/m" for clarity (**Figure S13**).

Reference:

[1] Lixue Tang et al., Printable Metal-Polymer Conductors for Highly Stretchable Bio-Devices [J]. ***iScience*** 4, 302-311 (2018). 10.1016/j.isci.2018.05.013

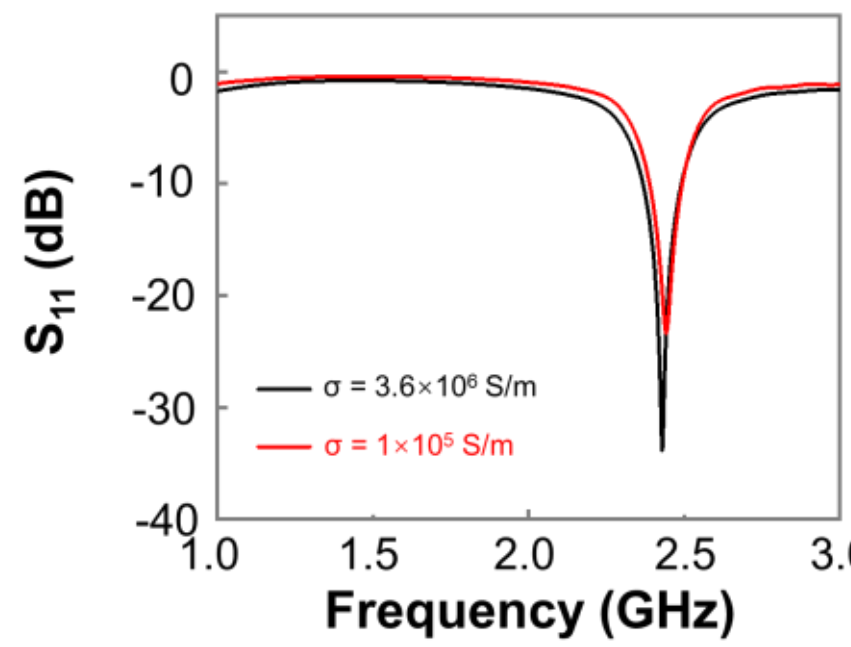


**Figure R3.1**. Simulated reflection coefficient ($S_{11}$) of the antenna using different conductivity values.

**Our modification to the manuscript:**

"······ By combining Ecoflex cured in a mold and liquid metal particles (LMPs) via air spraying (**Figure S11**), the DP-MSiA features a thin Ecoflex substrate layer (~2 mm) sandwiched between the LMPs-based patch with a cross-shaped slot and a square ground layer (**Figure 3a**). It should be noted that, after ultrasonication, the liquid metal particles exhibit a size distribution predominantly in the range of 1~4 μm (**Figure S12a**). However, the transfer process induces partial rupture of the oxide shells, facilitating particle coalescence at the Ecoflex surface and the formation of electrically percolated networks within the LMPs-Ecoflex composites **(Figure S12b**). Moreover, the intrinsically stretchable LMPs patterned on Ecoflex also maintain stable high conductivity upon stretching of 300% (**Figure S13**) to serve as an effective near-perfect conductor for efficient electromagnetic radiation. ······"

"······ Full-wave frequency-domain simulations were then conducted on the corresponding driven antenna models with the inset feed and feedlines. The patch and ground were assigned EGaIn conductivity ($\sigma = 1\times10^5$ S/m) and Ecoflex was modeled with $\varepsilon_r = 3.06$ and tan $\delta = 0.02$. Radiation (open and added space) boundaries were applied, with 50-ohm ports exciting the structure. Adaptive meshing with local refinement at feed/slot edges was used. A 1-4 GHz sweep was performed to obtain S-parameters, Z-parameters, and surface-current distributions. The power divider was simulated similarly. ······"

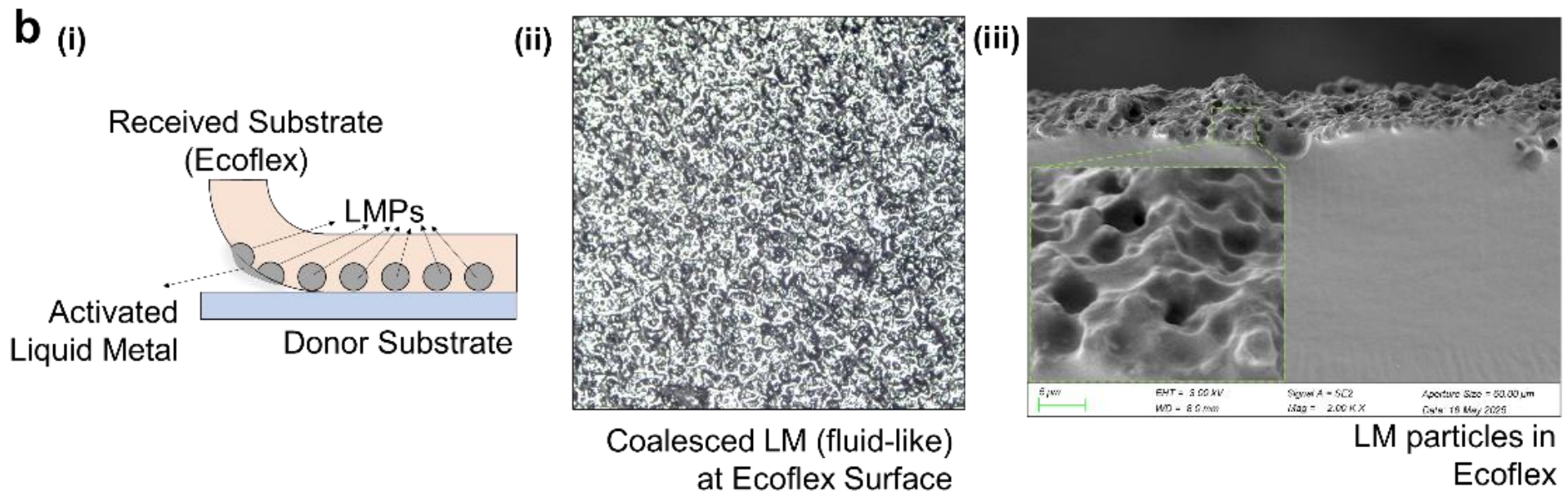


**Figure S12. Microstructure evolution of liquid metal particles (LMPs) before and after transfer. ······ (b)** (i) Schematic illustration of the transfer process from the donor substrate to the Ecoflex substrate. (ii) Optical image showing the coalesced, fluid-like morphology of liquid metal at the Ecoflex surface. (iii) Cross-sectional SEM image revealing liquid metal particles embedded near the Ecoflex surface, forming interconnected conductive pathways.

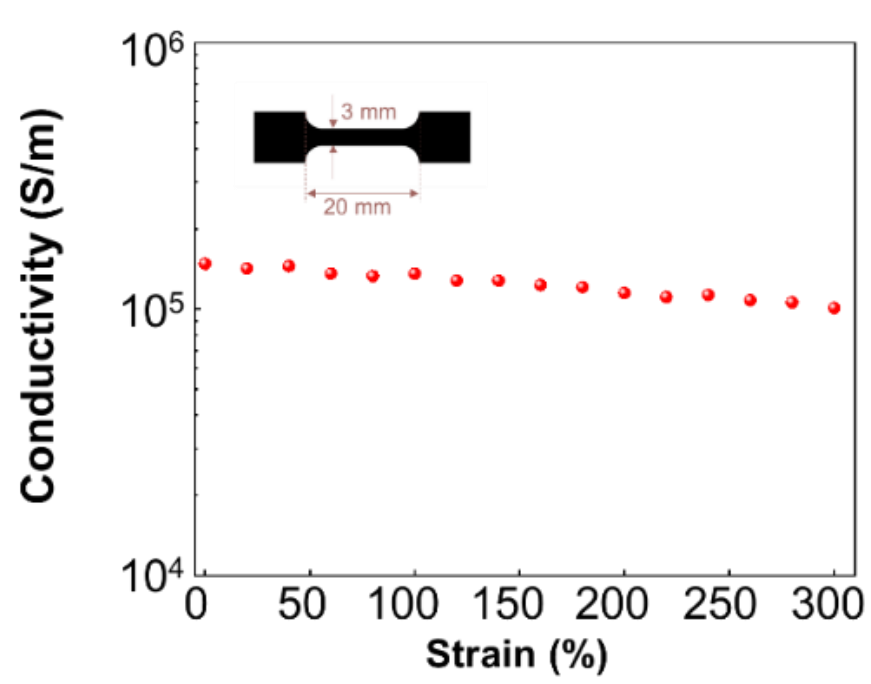


**Figure S13**. Conductivity–strain curve of the LMPs in a standard dumbbell shape (width of 3 mm in the middle) printed on the Ecoflex00-30 substrate.

*5. Although liquid metal and Ecoflex are naturally very soft and stretchable materials, the mechanical reliability under cyclic loading should be presented to guarantee the long term use.*

**Our response**:

Thank you for this constructive comment. We agree that the mechanical reliability under cyclic loading should be evaluated to support long-term use. To address this point, we performed cyclic stretching tests on the proposed antenna and monitored its electrical performance after repeated deformation.

In response, we performed cyclic stretching fatigue tests to evaluate the stability of both the communication platform and the WEH platform under repeated mechanical loading.

For the communication platform, a cyclic stretching test was carried out using 45° diagonal stretching. During the test, the antenna was manually stretched and released repeatedly, so that the evaluation simultaneously probed the cyclic durability of the stretchable antenna structure and the mechanical reliability of the rigid-soft connection, including the SMA/antenna interface. In each cycle, the antenna was stretched to approximately 50% tensile strain and then released to the undeformed state. The $S_{11}$ response was measured in the undeformed state after 0, 200, 400, and 600 cycles. In addition, the communication performance was evaluated in terms of the maximum communication distance after cyclic loading. For each cycle condition, the communication-distance measurement was repeated four times, and the results are presented as mean ± standard deviation with error bars. As shown in **Figure S45a,** only a minor variation in the $S_{11}$ response was observed after repeated deformation. Moreover, as shown in **Figure S45b**, the maximum communication distance remained above 100 m after 600 cycles, with only a small change from the initial state, confirming stable operation of the communication platform under repeated mechanical deformation.

For the WEH platform, we further examined the fatigue tolerance of the integrated rectifying system using two separate evaluations: (i) LED illumination and (ii) output voltage measured across a 160-Ω load. In each cycle, the device was stretched to approximately 50% tensile strain and then released to the undeformed state. As a direct demonstration of practical functionality, the same WEH device was first evaluated by LED illumination under the same RF exposure conditions after cyclic loading. The stretching tests were performed sequentially along the $x$-axis and then the $y$-axis, since these deformation modes load the main interconnect regions containing the rigid components and their joints. The device remained capable of illuminating the LED after 600 stretching cycles along the $x$-axis and after 600 additional stretching cycles along the $y$-axis, as shown in **Figure S30a**, indicating preserved operational capability after repeated deformation. For the 160-ohm-load demonstration, the output voltage was measured after the cyclic stretching tests were completed. After stretching cycles, under the same RF exposure conditions, the output voltage was then measured across a 160-ohm load under representative strain levels applied along four directions ($x$, $y$, 45°, and 135°). It can be found that only a minor drop in output voltage was observed relative to the initial state, as shown in **Figure S30b**. For example, at the highest measured strain, the output voltage decreased from about 1.7 V to about 1.5 V, indicating that the WEH platform maintained stable functionality after repeated deformation. This confirms that the integrated rectifier and its rigid-soft interconnections remained mechanically and electrically stable after repeated deformation.

**Our modification to the manuscript:**

"……The results confirm the importance of the cross-shaped slot structure and dual-port configuration in achieving multidirectional, strain-insensitive RF energy harvesting. In addition, cyclic stretching tests show that the WEH platform retains stable functionality after repeated deformation to illuminate the LED (**Figure S30a**). It also exhibits only a minor decrease in output voltage across a 160-ohm load after 600 stretching cycles along the *x*-axis followed by 600 additional cycles along the *y*-axis (**Figure S30b**). ……"

"……The sustained stable wireless transmission from the SP-MSiA provides the integrated system with uninterrupted ECG recordings even under 30% stretching along the *x*- and *y*-directions (**Figures 6c-ii, iii**), in direct contrast to the abrupt signal experienced by the system with the reference antenna. Furthermore, the cyclic mechanical reliability of the communication platform is also evaluated under repeated stretching to ~50% strain, showing only minor changes in $S_{11}$ (**Figure S45a**) and maintaining a maximum communication distance above 100 m even after 600 cycles (**Figure S45b**).

**2.6 Real-world applications of SP-MSiA-based wearable electronics**

……"

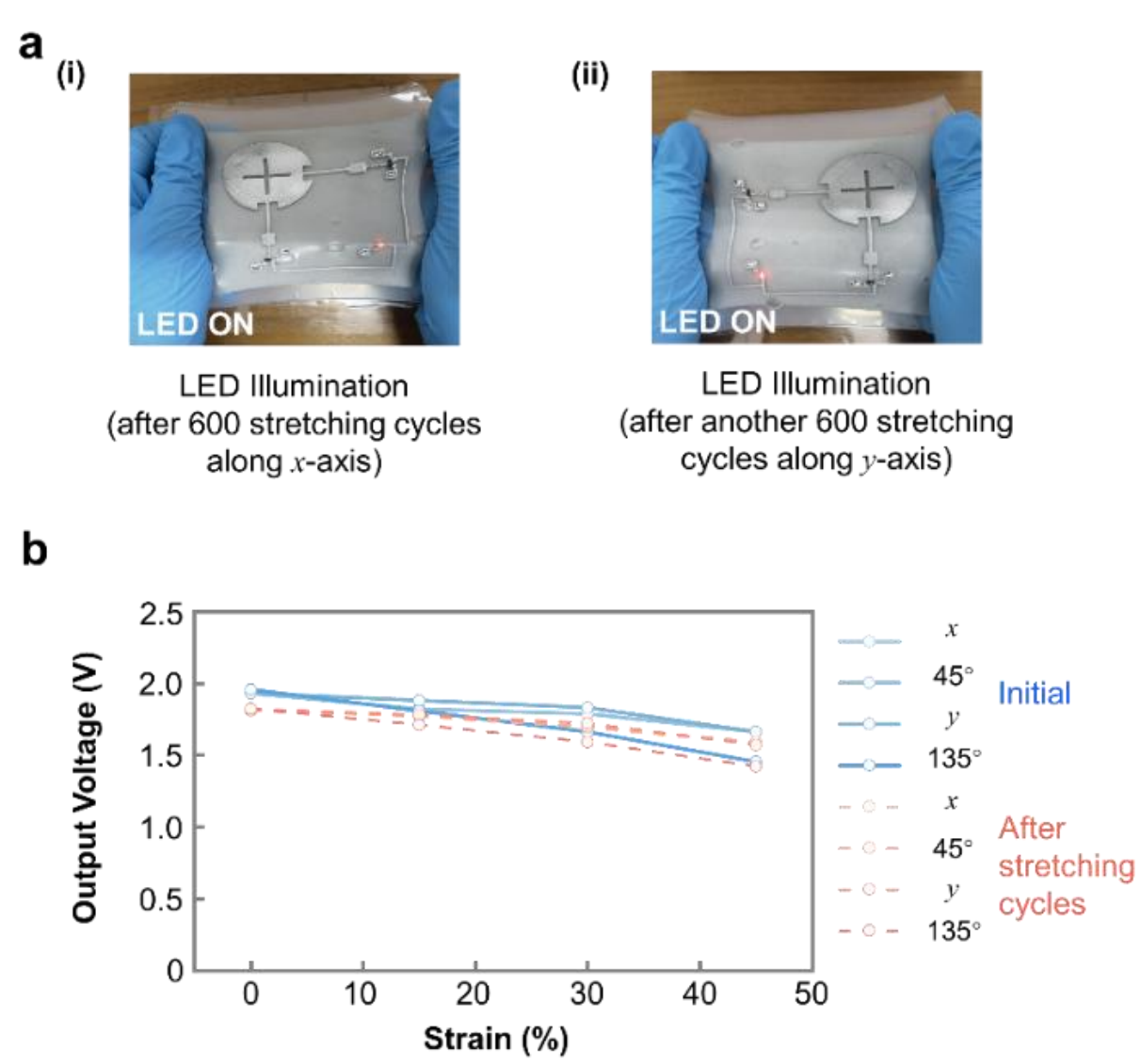


**Figure S30. Cyclic stretching characterization of the DP-MSiA-based WEH system. (a)** Photographs of LED illumination after cyclic stretching along the (i) *x*-axis and (ii) *y*-axis. **(b)** Output voltage versus strain measured along the *x*, 45°, *y*, and 135° directions before and after cyclic stretching.

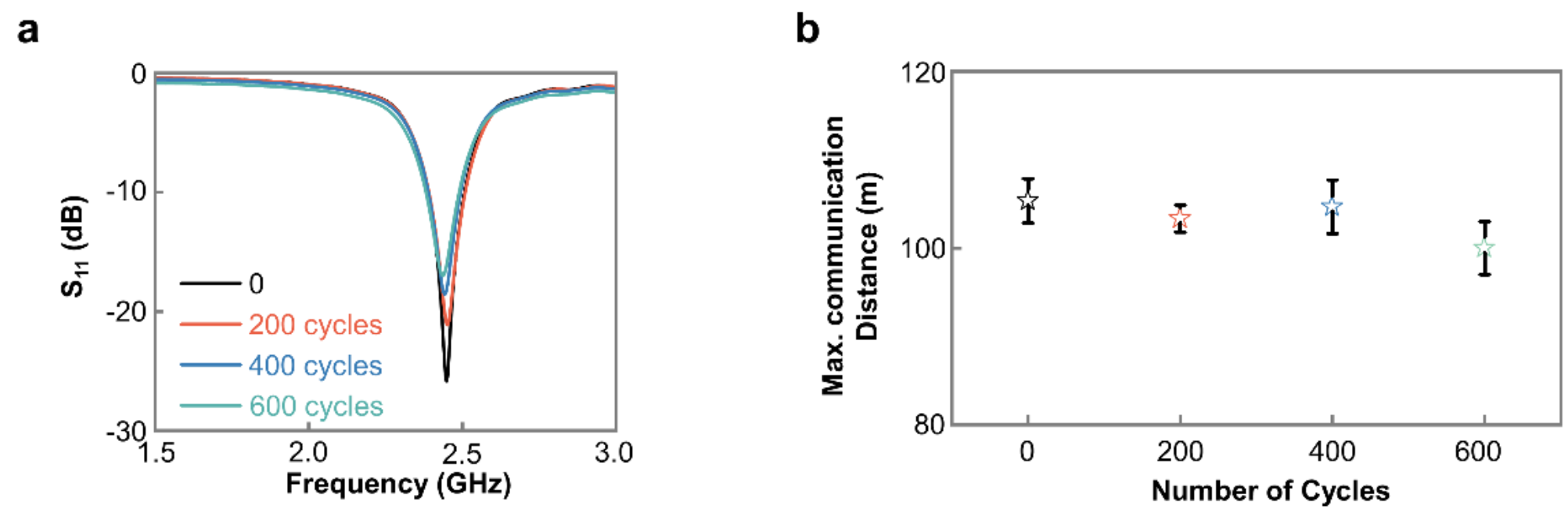


**Figure S45. Cyclic stretching characterization of the SP-MSiA.** **(a)** Measured $S_{11}$ of the SP-MSiA after 200, 400, and 600 stretching cycles. **(b)** Maximum communication distance of the SP-MSiA as a function of stretching cycle number. Error bars represent the standard deviation from four repeated measurements.

*6. Considering the signal weakness at 135 deg, people may still think a circular hole rather than the cross-slot is more beneficial even though the overall efficiency is lower. The authors should clearly show the performance comparison between the circular hole and cross-slot in figure 5f.*

**Our response**:

Thank you for this insightful comment. To address this concern more thoroughly, we fabricated the circular-hole strain-invariant design and experimentally measured its performance under applied strain along different directions.

Specifically, **Figure S38a** shows the photographs of the fabricated circular-hole sample in the unstrained state and under applied strain along the *x*, 45°, *y*, and 135° directions. **Figure S38b** presents the measured reflection coefficients for these cases. From the measured results, it can be seen that the resonance mode of interest remains nearly strain-insensitive under strain applied along the *x*, 45°, and *y* directions, showing a stable resonance frequency in these loading conditions. However, when strain is applied along 135°, the circular-hole design still exhibits a pronounced frequency shift. This measured behavior is fully consistent with our analysis and discussion in the manuscript. In addition, **Figure S38c** shows the measured radiation performance comparison between the circular-hole design and the cross-slot design. The results show that, for the circular-hole case, the realized gain remains relatively stable under strain applied along the *x*, 45°, and *y* directions, which is

consistent with the strain-insensitive resonance behavior. Nevertheless, the absolute maximum realized gain of the circular-hole design (around -4dBi) is significantly lower than that of the cross-slot design (around 3 dBi) in all cases. This is because the circular-hole structure has lower radiation efficiency (below -10 dB).

Based on these added measured results, the revised manuscript now more clearly demonstrates the difference between the two structures in terms of maximum realized gain in **Figure 5f**.

**Our modification to the manuscript:**

**"2.4 Design and characterization of SP-MSiA for stable communication**

…… Moreover, the realized gain of -5.6 dBi from the SP-MSiA under stretching along the strain-sensitive 135° direction is also higher than that of -9.6 dBi from the conventional counterpart due to its smaller resonance-frequency shift. As a complementary metric to realized gain, the absolute radiation efficiency of the SP-MSiA in the undeformed state is -4.55 dB, which is close to that of the conventional no-slot patch antenna (-4.3 dB), and remains stable under representative strain levels (**Figure S37**). A similar trend is also observed for the circular-hole strain-invariant design (**Figure S38**). Specifically, the circular-hole antenna maintains good strain-insensitive resonance behavior under stretching along the *x*-, 45°-, and *y*-directions, but still exhibits a pronounced frequency shift under stretching along the 135° direction. However, despite this partial directional strain tolerance, its maximum realized gain remains much lower than that of the cross-slot SP-MSiA in all cases (**Figure 5f**), owing to its lower radiation efficiency. Therefore, the cross-slot design provides a more favorable overall trade-off between directional strain robustness and radiation performance. Nonetheless, the substantially enhanced communication stability of the SP-MSiA under multidirectional stretching paves the way for the practical implementation of stretchable antennas in wearable electronics.

……"

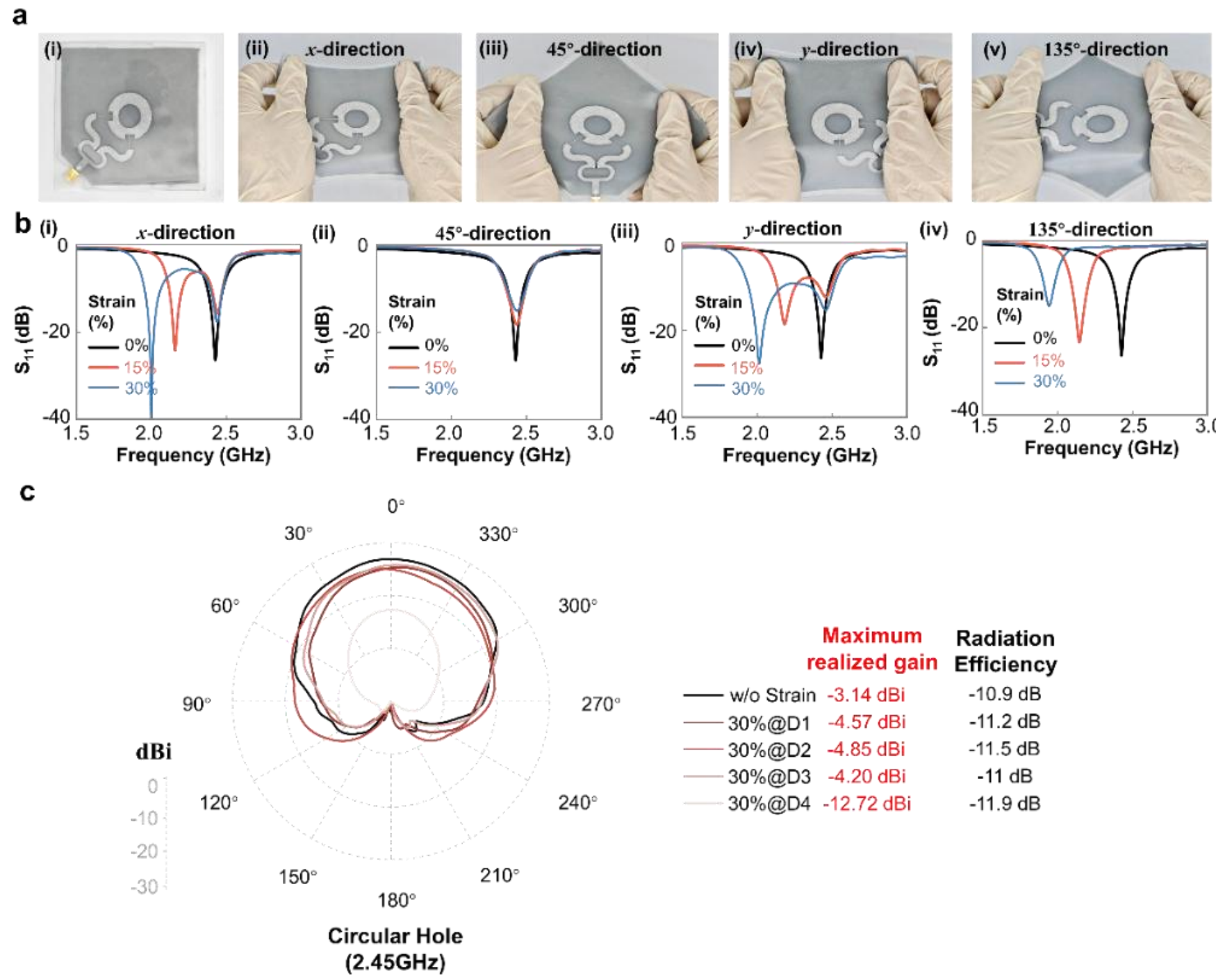


**Figure S38.** **(a)** Photographs of the circular-hole strain-invariant antenna (i) before and under stretching along the (ii) *x*, (iii) 45°, (iv) *y*, and (v) 135° directions. **(b)** Measured S-parameters ($S_{11}$) of the circular-hole strain-invariant antenna under applied strain (15% and 30%) along the (i) *x*, (ii) 45°, (iii) *y*, and (iv) 135° directions. **(c)** Measured radiation patterns and maximum realized gain of the circular-hole strain-invariant antenna before and after 30% strain applied along the *x*, 45°, *y*, and 135° directions.

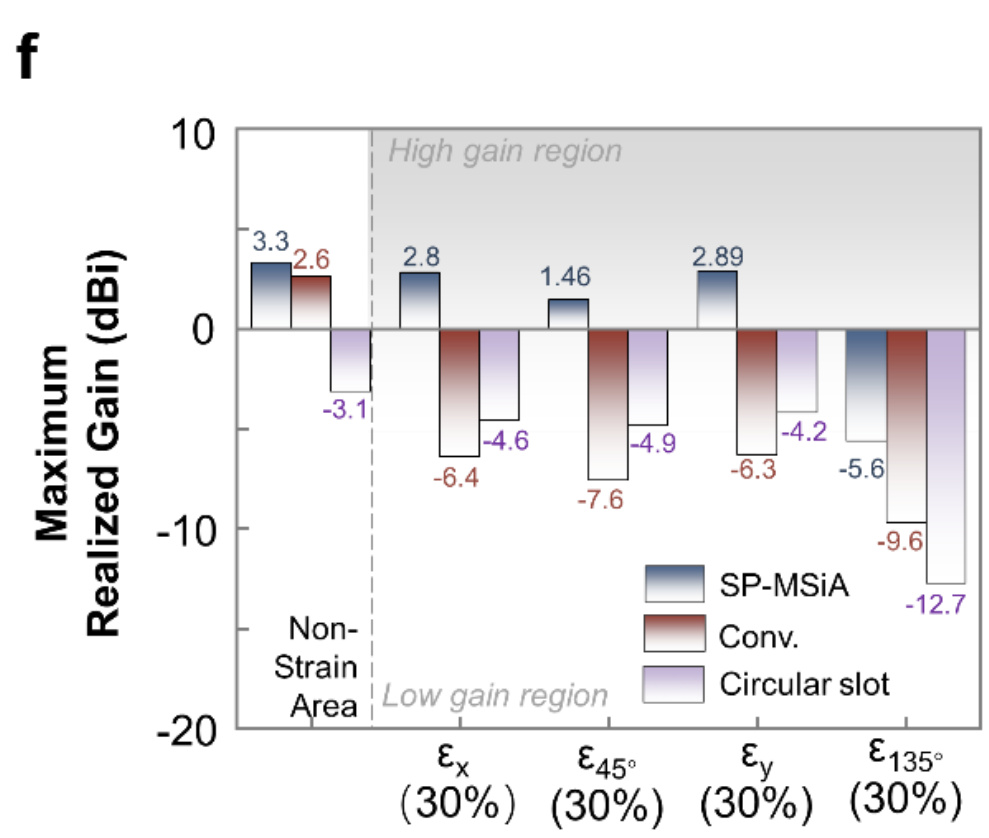


**Figure 5.** **(f)** Measured maximum realized gain of the SP-MSiA, the conventional stretchable antenna, and circular-slot stretchable antenna under tensile strain of 30% applied along (i) *x*, (ii) 45°, (iii) *y*, and (iv) 135° directions.

*7. The authors assume that the device is mechanically deformed only in-plane. However, the device is subject to bending and bi-axial stretching when it is located on the textile or human body. The effect of those mechanical deformations on the antenna performance would further improve the completeness of the manuscript.*

**Our response**:

Thank you for this important comment. We agree that, in practical wearable use, the antenna may experience not only in-plane uniaxial deformation but also bending and biaxial stretching, and we appreciate the opportunity to clarify these effects.

For biaxial stretching, we also evaluated the antenna under representative dual-axis deformation states, as shown in **Figure S39.** Under 30% biaxial stretching with intersection angles of 180°, 150°, 120°, and 90° between the two applied stretching directions, the SP-MSiA exhibits resonance shifts of 7.5, 27.5, 46.9, and 221.1 MHz, respectively. These values are all smaller than those of the reference antenna under the same conditions, namely 270.6, 88.0, 136.4, and 244.2 MHz, respectively, as shown in **Figure S39 a–d(iii)**. This further demonstrates an important strength of the proposed design, namely that its strain-robust behavior extends beyond uniaxial stretching to representative biaxial deformation states relevant to wearable applications. As the intersection angle decreases to 90°, the deformation approaches a near-isotropic stretching condition, under which the slot-patch compensation becomes less effective because the overall patch size increases and the resonance frequency decreases. However, such a condition is more stringent than most practical wearable deformation states, where biaxial strains are generally unequal in magnitude and not orthogonal.

For bending, we evaluated the antenna under three representative conditions, namely flat, bent on the forearm with a smaller curvature, and bent on the calf with a larger curvature, as shown in **Figure S40**. Relative to the flat state, the measured $S_{11}$ responses in both bent cases exhibit only a slight resonance shift, while the impedance matching remains largely unchanged. This indicates that out-of-plane bending has only a minor effect on the resonance frequency and impedance performance of the proposed antenna. Thus, we have clarified this point in the revised manuscript.

**Our modification to the manuscript:**

**"2.4 Design and characterization of SP-MSiA for stable communication**

……Nonetheless, the substantially enhanced communication stability of the SP-MSiA under

multidirectional stretching paves the way for the practical implementation of stretchable antennas in wearable electronics.

To further evaluate practical wearable conditions, we also examine biaxial stretching, out-of-plane bending, and on-body loading. Under 30% biaxial strain with intersection angles (i.e., the angle between the two applied biaxial stretching directions) of 180°, 150°, 120°, and 90°, the SP-MSiA exhibits resonance shifts of 7.5, 27.5, 46.9, and 221.1 MHz, respectively, which are smaller than those of the reference antenna (270.6, 88.0, 136.4, and 244.2 MHz) (**Figure S39**). However, as the intersection angle decreases to 90°, the frequency-stability advantage of the SP-MSiA diminishes because the slot-patch compensation becomes less effective as the patch is stretched near isotropically, increasing the overall patch size and decreasing the resonance frequency. Such a condition is more stringent than most practical wearable deformation states, where biaxial strains are generally unequal in magnitude and not orthogonal. Moreover, measurements under out-of-plane bending conditions show only slight changes in resonance frequency and $S_{11}$ **(Figure S40**). These results indicate that the proposed antenna maintains stable impedance behavior under representative wearable deformation scenarios.

……”

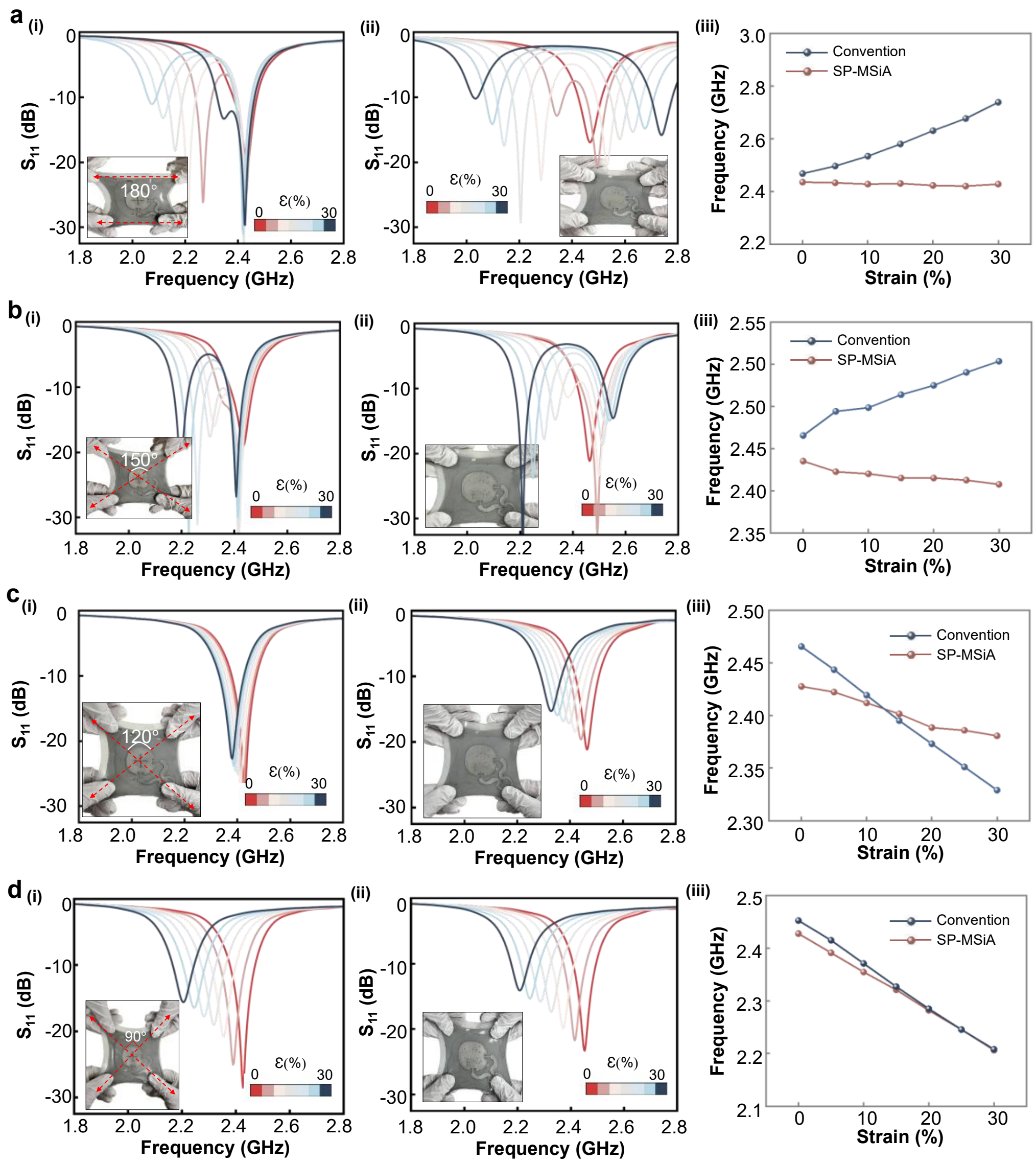


**Figure S39.** Measured $S_{11}$ of the (i) SP-MSiA and (ii) reference conventional antenna under dual biaxial tensile strain of 0, 5, 10, 15, 20, 25, and 30% with four representative inter-axis angles of **(a)** 180° (parallel), **(b)** 150°, **(c)** 120°, and **(d)** 90°, along with (iii) the corresponding extracted resonance frequency. The insets show photographs of the SP-MSiA and the reference conventional antenna under biaxial strain.

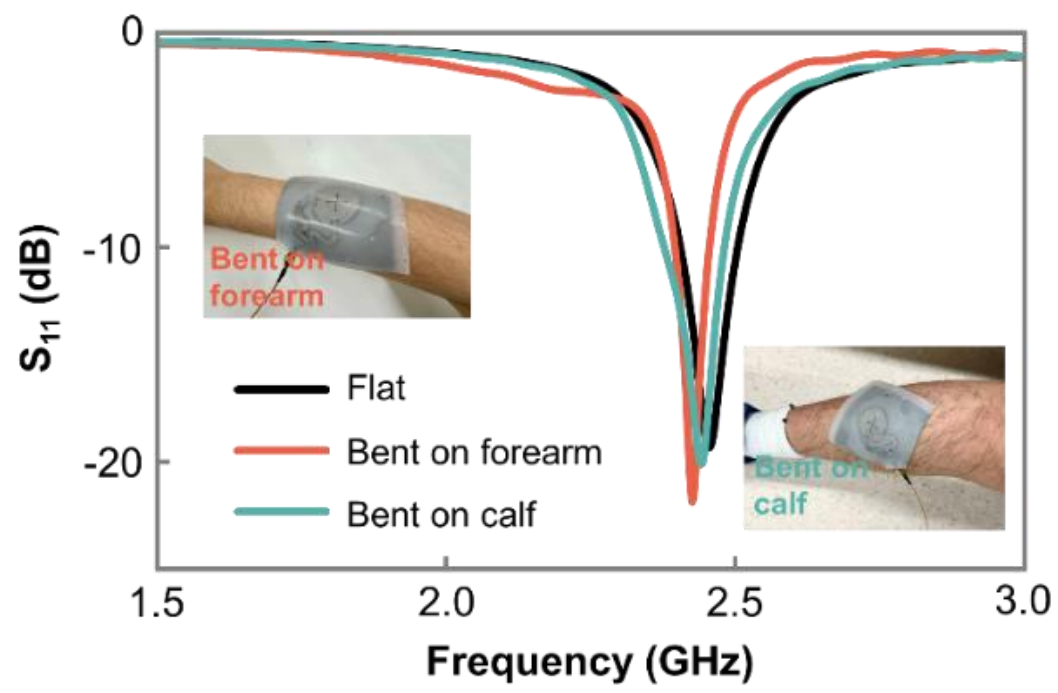


**Figure S40.** Measured $S_{11}$ of the SP-MSiA in the flat state and under bending on the forearm and calf.

*8. In Fig. 2f (i), slot number 8 is invisible.*

**Our response**:

Thank you for pointing out this issue. We apologize that slot number 8 in **Figure 2f-i** was not clearly visible in the previous version. In the revised manuscript, we have increased the font size and improved the labeling in this Figure to make slot number 8 clearly visible.

**Our modification to the manuscript:**

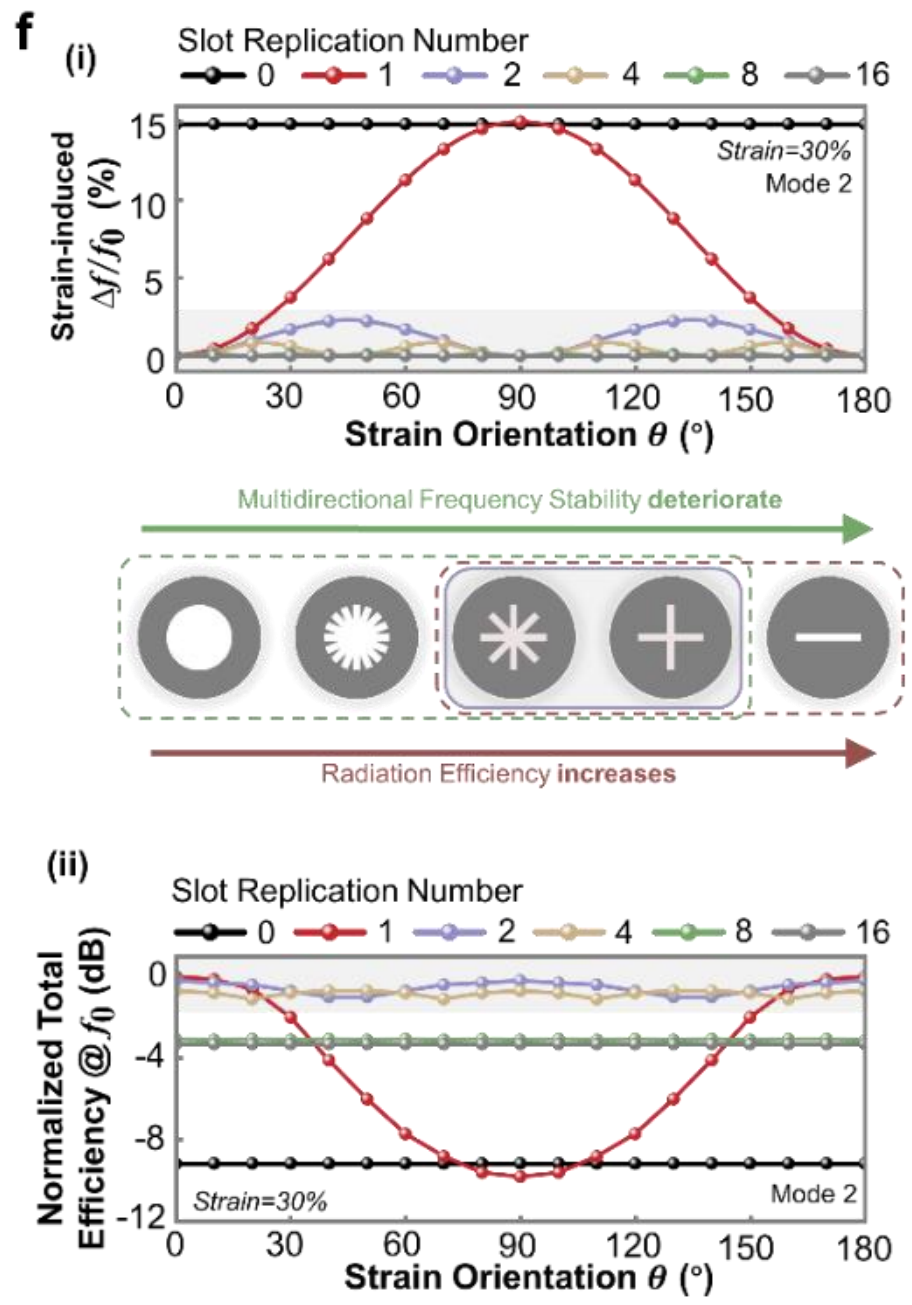


**Figure 2. Symmetry-based mode–mechanical coupling and modal compensation for multidirectional strain insensitivity.** **(f) (i)** Mode 2 multidirectional frequency stability under strain from a sweep of slot replication number, and **(ii)** corresponding total efficiency under the same strain.
